# Human and mouse adrenal glands are characterized by species-specific steroidogenic states and tissue turnover

**DOI:** 10.1038/s41588-026-02737-1

**Published:** 2026-09-08

**Authors:** Maria E. Kastriti, Denis Maksimov, Julia Krupinova, Marina Utkina, Dmitry Beltsevich, Anna Roslyakova, Olga Glazova, Samira Kaziakhmedova, Alina Ryabova, Anna Kuznetsova, Anastasia Shcherbakova, Ekaterina Bondarenko, Zoia Antysheva, Eugene Albert, Lilia Urusova, Anastasia Lapshina, Anastassia Chevais, Ekaterina Avsievich, Valentin Trofimov, Galina Melnichenko, Natalya Mokrysheva, Ivan Dedov, Lukas Englmaier, Julian Petersen, Oleg Gusev, Andreas Heinzel, Rainer Oberbauer, Pavel Volchkov, Peter V. Kharchenko, Igor Adameyko

**Affiliations:** 1 Medical University of Vienna, Center for Brain Research, Department of Neuroimmunology, Vienna, Austria; 2 Lomonosov Moscow State University, Moscow, Russia; 3 Moscow Clinical Scientific Center named after A.S. Loginov, Moscow, Russia; 4 Endocrinology Research Centre, Moscow, Russia; 5 CeMM Research Center for Molecular Medicine of the Austrian Academy of Sciences, Vienna, Austria; 6 Department of Orthodontics, University of Leipzig Medical Center, Leipzig, Germany; 7 Intractable Disease Research Center, Graduate School of Medicine, Juntendo University, Tokyo, Japan; 8 Life Improvement by Future Technologies (LIFT) Center, Moscow, Russia; 9 Federal Center of Brain Research and Neurotechnologies, Моscow, Russia; 10 Department of Nephrology, Internal Medicine III, Medical University of Vienna, Vienna, Austria; 11 National Medical Research Centre of Cardiology named after Academician E.I. Chazov, Moscow, Russia; 12 Almazov National Medical Research Centre, St Petersburg, Russia; 13 Department of Biomedical Informatics, Harvard Medical School, Boston, MA USA; 14 Department of Physiology and Pharmacology, Karolinska Institutet, Solna, Sweden

**Keywords:** Transcriptomics, Stem cells, Stem cells

## Abstract

The adult adrenal cortex undergoes constant renewal, yet underlying human-specific mechanisms remain poorly understood. Here we generated single-cell and spatial transcriptomic atlases of adult human and mouse adrenal glands, leveraging single-cell-resolution spatial data and a rare clonal mosaic case for lineage inference. In humans, we identified age-associated zona glomerulosa (ZG) cell states with direct cortisol synthesis capacity and sex-specific differences in inferred cholesterol balance. Cross-species comparison revealed conserved aldosterone-producing ZG but notable divergence in zona fasciculata markers, absence of zona reticularis homologs in mice and differential SHH–WNT4 signaling in proliferating cells. We uncovered human *WT1*^−^ capsule-to-ZG transition and vascular smooth muscle cell-to-steroidogenic transitions supported by mosaic lineage evidence. We revealed dispersed proliferating cortical *SF1*^+^*EZH2*^*+*^ cells throughout the human cortex in contrast with ZG restriction in mice. Taken together, our data expand the centripetal renewal model and establish a comparative framework for human adrenocortical biology.

## Abstract (web summary)

The authors present a comprehensive comparative resource for adult human and mouse adrenal glands through single-cell and spatial transcriptomic analyses.

## Main

The adrenal gland is involved in maintaining homeostasis and mediating the stress response^1–3^. The mesoderm-derived cortex synthesizes steroid hormones, including glucocorticoids regulating metabolism and immune responses, mineralocorticoids controlling blood pressure and electrolyte balance, and biologically weaker androgens^1^. Corresponding cellular populations are organized in three zones: zona glomerulosa (ZG, HSD3B2^+^ aldosterone-secreting cells through CYP11B2 enzymatic activity), zona fasciculata (ZF, HSD3B2^+^CYP11B1^+^CYP17A1^+^ cells synthesizing glucocorticoids) and zona reticularis (ZR, CYP17A1^+^SULT2A1^+^CYB5A^+^ androgen-producing cells)^4^, with the last absent in rodents due to epigenetic silencing of *Cyp17a1*^5,6^. Chromaffin cells of the medulla drive acute stress response by secreting epinephrine and norepinephrine^3,7^.

Rodent studies established a centripetal model of cortical cell generation: mesenchymal capsular cells release the WNT agonist RSPO3, stimulating outer ZG stem cells that differentiate toward ZG and subsequently ZF cells^8^. Whether this model applies to humans remains unclear: the cellular sources, signaling centers and progenitor identities governing human adrenocortical renewal have not been characterized at molecular resolution and the degree to which rodent mechanisms are conserved is unknown.

**Fig. 1:**
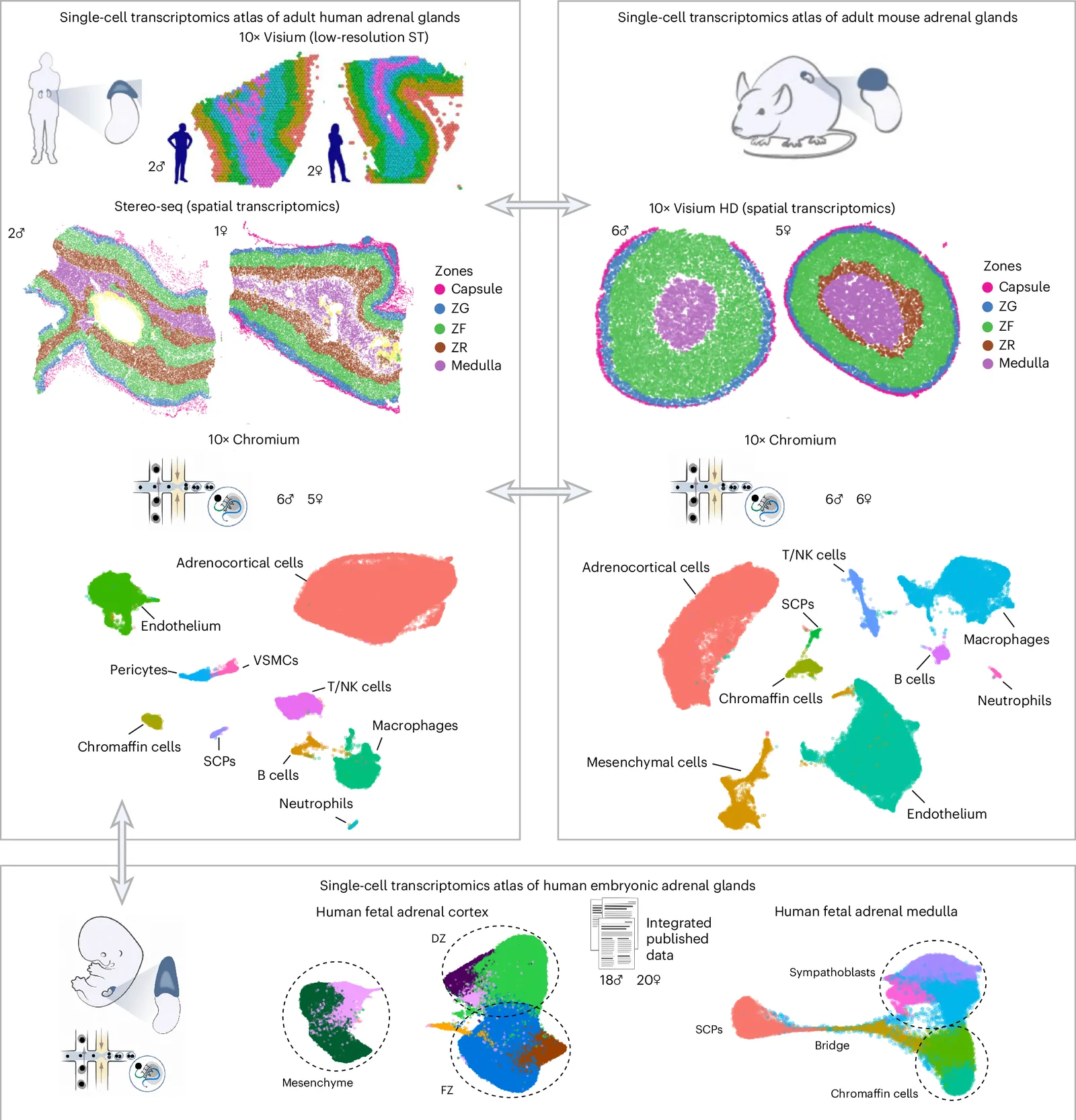
Overview of the study. Comprehensive scRNA-seq and high-resolution spatial transcriptomics atlas of adult human and mouse adrenal glands and human fetal adrenal development. NK, natural killer; ST, spatial transciptomics; VSMCs, vascular smooth muscle cells; SCPs, Schwann cell precursors; FZ, fetal zone; DZ, definitive zone.

Revealing the human-specific aspects of adrenocortical cell-type composition and renewal is crucial for understanding endocrine homeostasis and developing therapies for adrenal insufficiency, hyperplasia and cortical malignancies. To address this, we generated single-cell and spatial transcriptomic atlases of adult human and mouse adrenal glands, enabling direct cross-species comparison of progenitors, renewal mechanisms, sexual dimorphism and steroidogenic cell-state diversity (Fig. 1).

## Results

### Steroidogenic cell states within the human adrenal cortex

**Extended Data Fig. 1:**
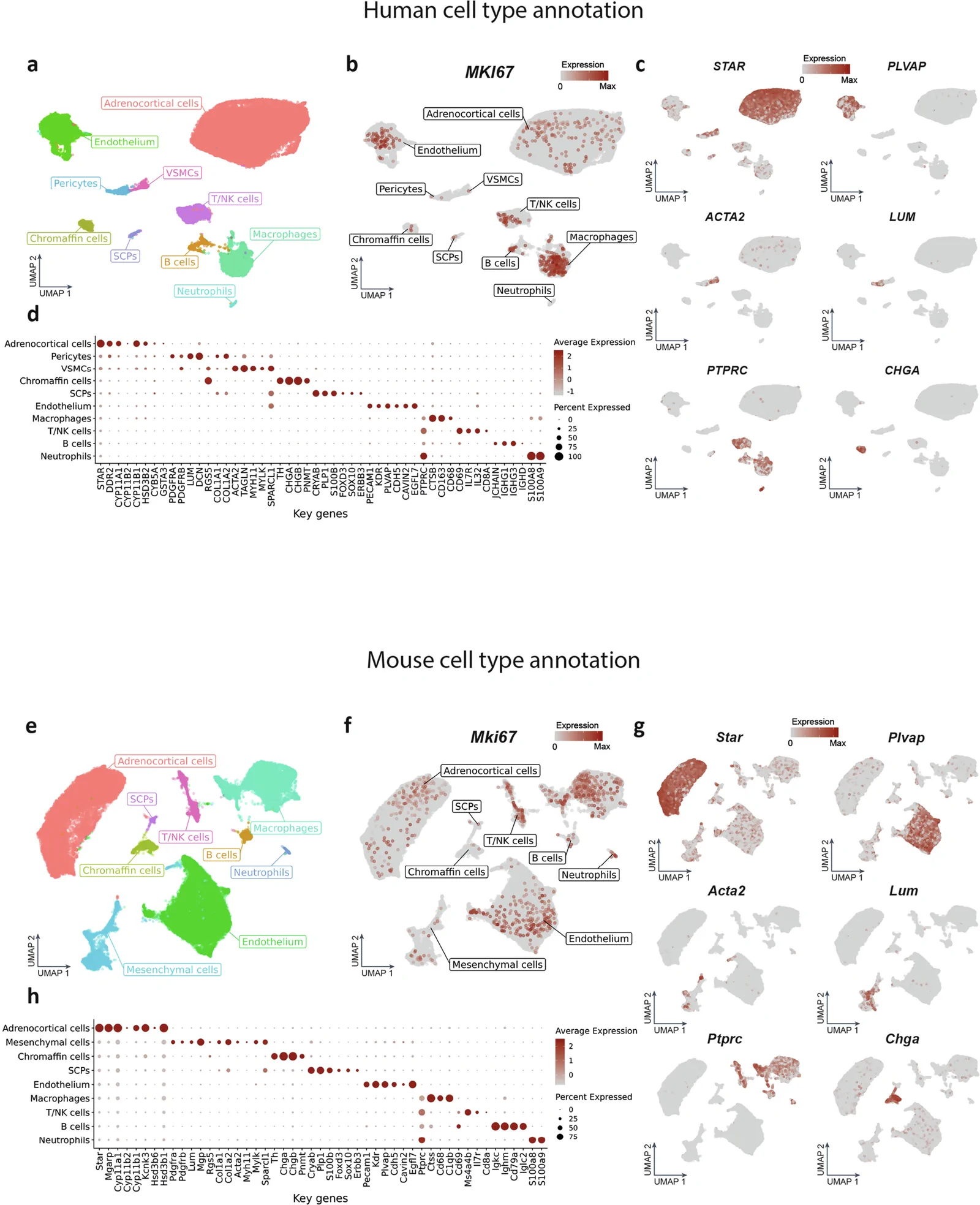
Cell type heterogeneity of human and mouse adult adrenal gland. **a-d**, Human data, **e-h**, mouse data. **a**, UMAP of human adult adrenal gland cells, color coded by cell type (60,146 cells). **b**, Proliferating cells defined by MKI67 expression. **c**, Key cell type markers on the combined UMAP. *STAR* - adrenocortical cells, *PLVAP* - endothelium, *ACTA2* - vascular smooth muscle cells (VSMCs), *LUM* - Pericytes (Fibroblasts), *PTPRC* - immune cells, *CHGA* - chromaffin cells. **d**, DotPlot of cell type marker genes. **e**, UMAP of mouse adult adrenal gland cells, color coded by cell type (67,665 cells). **f**, Proliferating cells defined by *Mki67* expression. **g**, Key cell type markers on the combined UMAP. *Star* - adrenocortical cells, *Plvap* - endothelium, *Acta2*, *Lum* - mesenchymal, *Ptprc* - immune cells, *Chga* - chromaffin cells. **h**, DotPlot displaying genes defining each cell type. VSMCs: vascular smooth muscle cells; SCPs: Schwann cell precursors.

**Extended Data Fig. 2:**
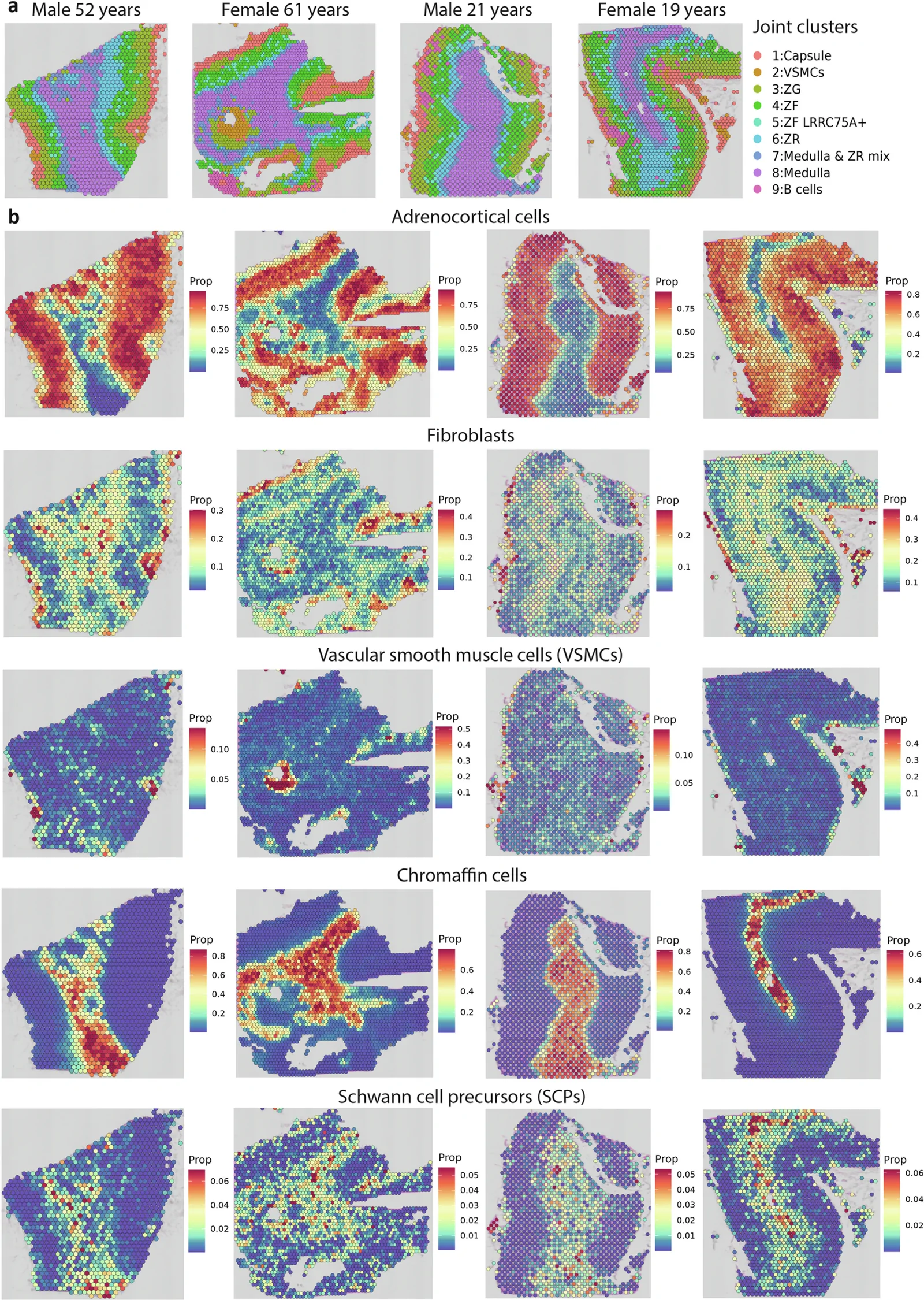
Visium cell type deconvolution for adrenocortical, mesenchymal and medullary cell types. **a**, Joint clusters from Seurat integration of four Visium samples shown on each tissue slide. **b**, Proportion of adrenocortical, mesenchymal (fibroblast or vascular smooth muscle cell-VSMC), medullary (chromaffin or Schwann cell precursor-SCP) cell types per Visium spot, obtained by RCTD cell type deconvolution using the human adult adrenal single cell RNA-sequencing reference. VSMCs: vascular smooth muscle cells; ZG: zona glomerulosa; ZF: zona fasciculata; ZR: zona reticularis.

We first performed single-cell transcriptomics (single-cell RNA sequencing (scRNA-seq)) to characterize human adrenocortical cell populations on the adrenals of six male and five female donors, aged 18–73 years (Table 1 and Supplementary Figs. 1 and 2), recovering *STAR*^+^ steroidogenic cells, immune, chromaffin and sustentacular glial cells, mesenchyme, endothelial and perivascular cells (Extended Data Fig. 1a–d and Supplementary Figs. 3a and 4). Orthogonal Visium spatial transcriptomics (two male and two female donors, aged 19–62 years) confirmed cell types and revealed immune cell distribution suggestive of tertiary lymphoid tissue (Extended Data Fig. 2 and Supplementary Figs. 5 and 6).

**Fig. 2:**
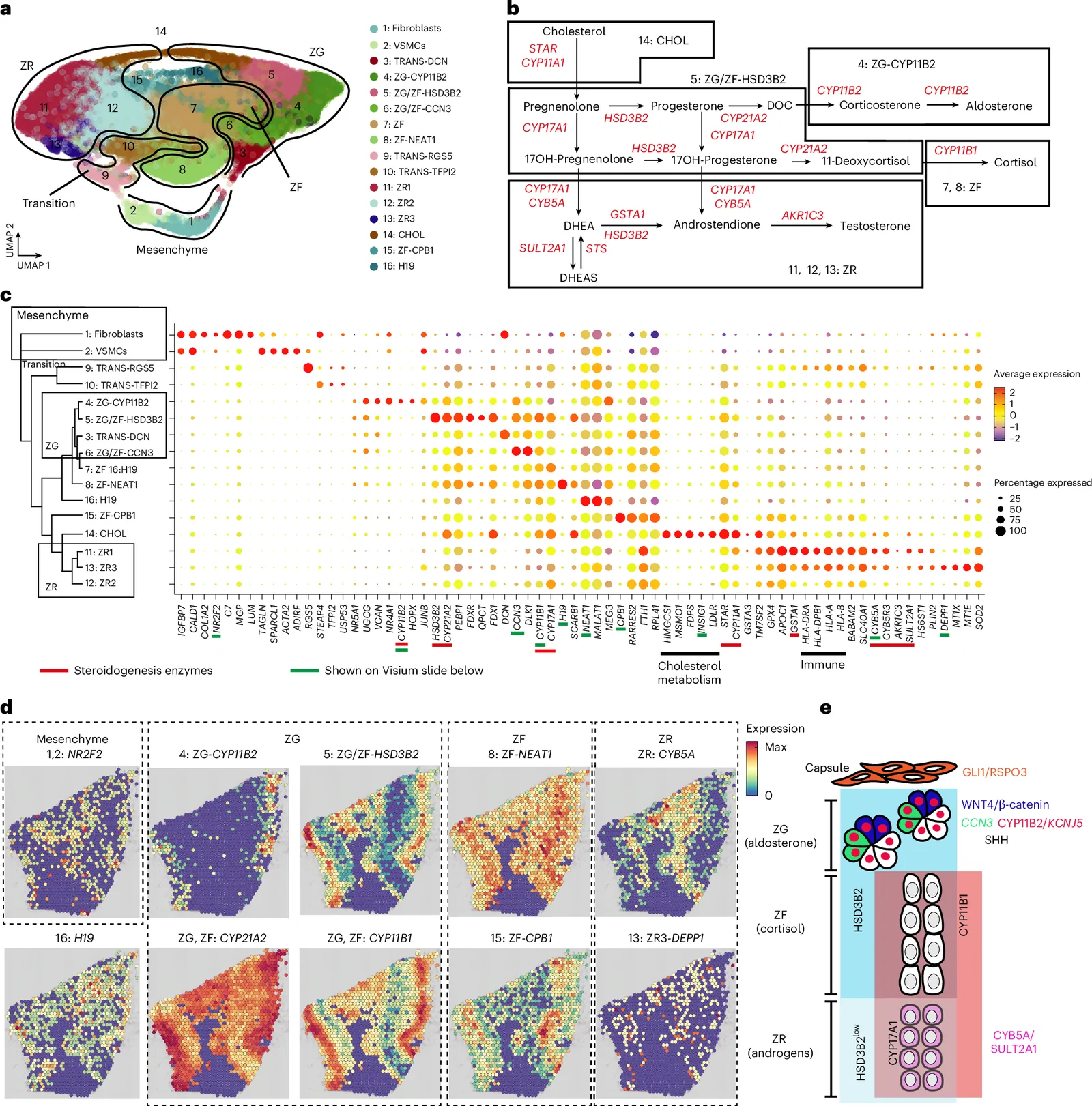
General transcriptional and spatial heterogeneity of the human adult adrenal cortex. **a**, UMAP of integrated adrenocortical and mesenchymal cells with zonation indicated (39,985 cells). **b**, Schematic of steroidogenesis in the human adrenal gland, with cluster-specific pathway specializations highlighted. **c**, DotPlot of cluster-specific marker genes and key steroidogenic enzymes (underlined in red). Genes are shown on Visium slides (**d** and **e**) underlined in green. Cluster hierarchy is shown on the left. **d**, Spatial expression of key marker genes by Visium, with each cluster localizing predominantly to the ZG, ZF or ZR. **e**, Schematic of the normal human adult adrenal cortex zones, markers, steroidogenic roles and ZG internal heterogeneity. DHEA, dehydroepiandrosterone; DHEAS, dehydroepiandrosterone sulfate.

Subselection of mesenchymal (including perivascular) and adrenocortical cells revealed continuous transcriptional states (Fig. 2a and Supplementary Table 1) annotated using known markers and steroidogenic enzymes^1,9^ (Fig. 2b,e): cells expressing *CYP11B2*, *KCNJ5*, *LGR5*, *WNT4*, *CCN3* and *VSNL1* represented aldosterone-producing ZG; *CYB5A*, *SLC13A5*, *SLC27A2* and *HSD3B2*^low^ expressing cells androgenic ZR and the remaining *STAR*^+^ cells expressing *CYP11B1*, *CYP17A1* and/or *HSD3B2* represented glucocorticoid-producing ZF, confirmed by spatial transcriptomics with zonation annotations confirmed by published ZG and ZR signatures^9^ (Fig. 2b–d and Supplementary Fig. 7a).

A population intercalated between ZG and ZR showed high expression of *H19* (cluster 16; Fig. 2a,c), a long noncoding RNA induced in adrenocortical cells upon adrenocorticotropic hormone stimulation^10^, co-expressing steroidogenic genes of mixed identity (*CYP11B1*: ZF or ZR marker; *CYP17A1*: ZF marker; *HSD3B2*: ZG or ZF (ZG/ZF) marker; and *CYP21A2*: pancortical). Spatially, the *H19*^high^ population was enriched near the capsule and ZR–medulla interface and scattered across other cortical regions (Fig. 2d).

**Fig. 3:**
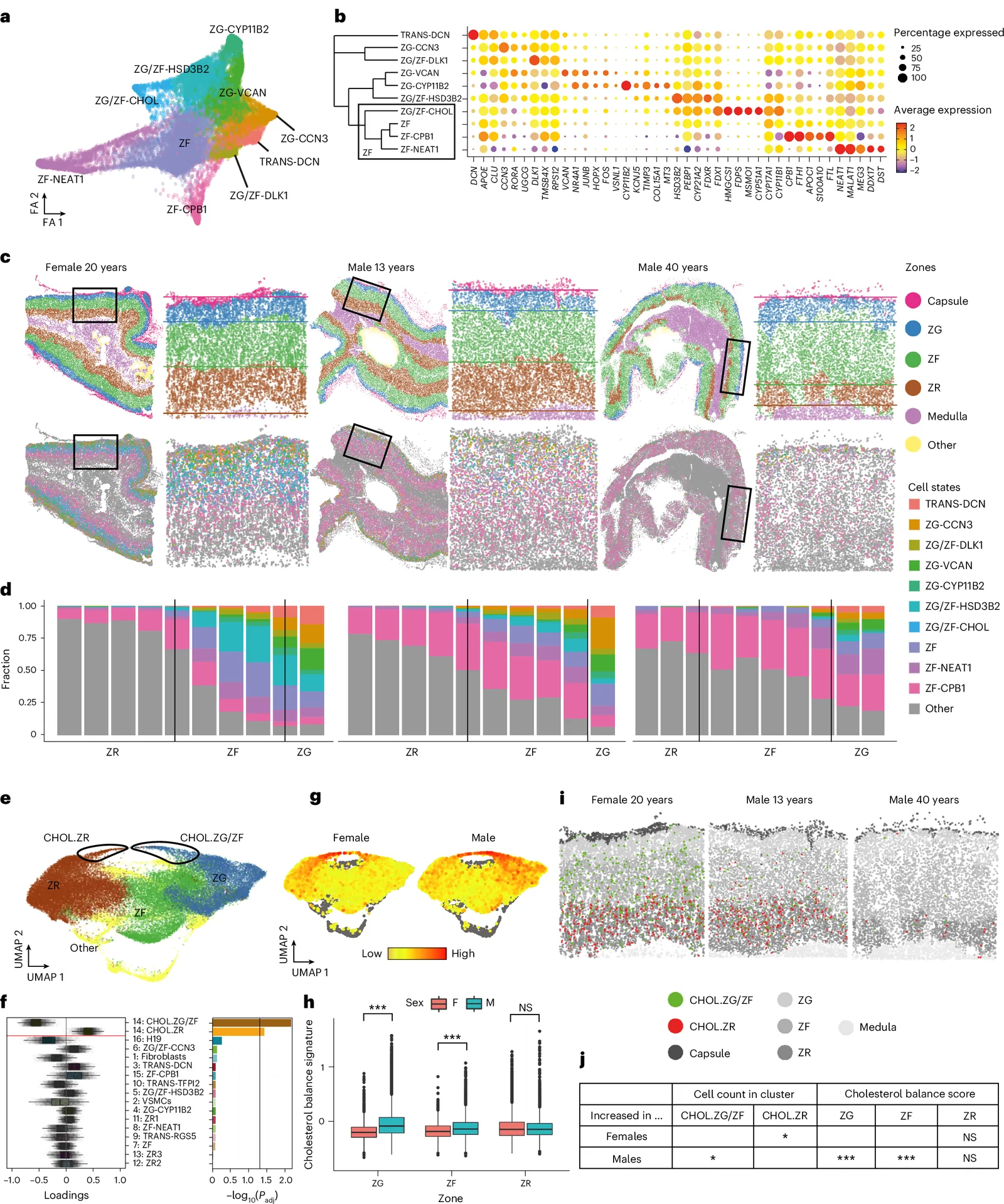
Resolved cell states and sexual dimorphism of the human adult cortex. **a**, ForceAtlas2 embedding of reclustered ZG and ZF (20,255 cells). **b**, DotPlot of cluster-specific genes with cluster hierarchy. **c**, Stereo-seq spatial transcriptomics of three samples with unsupervised zone identification, cell segmentation and scRNA-seq reference-based annotation. Whole slides (85,276, 64,499 and 75,402 cells) and representative cortical areas (8,420, 7,076 and 5,536 cells) are shown; each dot represents one annotated cell colored by zone. **d**, Bar plots showing fractional ZG or ZF cluster composition from ZR–medulla to capsule. The vertical lines mark zone boundaries. **e–j**, Sexual dimorphism in adrenocortical cholesterol (CHOL) biosynthesis. **e**, CHOL.ZG/ZF and CHOL.ZR subpopulations with predominantly ZG or ZR signatures. **f**, Compositional differences between sexes: loadings from 5,000-bootstrap permutation (left) and adjusted *P* values (right). CHOL.ZG/ZF is male overrepresented (*P* = 0.007) and CHOL.ZR is female overrepresented (*P* = 0.037), using a one-sided bootstrap test and Benjamini–Hochberg (BH) adjusted. **g**, Cholesterol balance score on male (right) and female (left) UMAP subsets. **h**, Cholesterol balance score by zone and sex. Cell counts are: female ZG: 4,610; male ZG: 5,456; female ZF: 3,946; male ZF: 5,894; female ZR: 6,322; and male ZR: 8,205. A one-sided bootstrap test was used, with BH-adjusted *P* values: 0.0001 (ZG), 0.0075 (ZF) and 0.4410 (ZR). **i**, Spatial distribution of CHOL.ZG/ZF and CHOL.ZR. **j**, Summary of sex differences in cholesterol biosynthesis. For all box plots, the center is the median, the bounds the quartiles and the whiskers 1.5× the interquartile range (IQR). ^*^P_adj_ < 0.05; ^**^P < 0.01; ^***^P < 0.001; NS, nonsignificant: *P* > 0.05. FA, ForceAtlas dimension; F, female; M, male.

ZG comprises cells with distinct transcriptional features based on established markers such as *CYP11B2* and *HSD3B2* (Fig. 2c), informed by previous studies^4,9^. To examine differentiation toward ZF according to the centripetal model, we reclustered ZG and ZF (Fig. 3a,b and Supplementary Table 2). ZG cells were divided in (1) canonical aldosterone-producing *VCAN*^+^*CYP11B2*^+^ cells (ZG-CYP11B2); (2) *VCAN*^high^*CYP11B2*^−^ cells (ZG-VCAN) corresponding to immature *CYP11B2*^−^*CYP17A1*^−^*CYP11B1*^−^ cells in ZG; (3) a *HSD3B2*^high^ cluster (named ZG/ZF based on *HSD3B2* expression and partial expression of ZF markers *CYP11B1* and *CYP17A1*); (4) a *DLK1*^high^ cluster; (5) a *CCN3*^high^ cluster; and (6) a cluster enriched in cholesterol metabolism genes (ZG-CHOL) (Fig. 3a,b, Supplementary Table 3 and Supplementary Fig. 8). These subclusters partially persisted when reclustering individuals separately (Supplementary Fig. 9).

**Extended Data Fig. 3:**
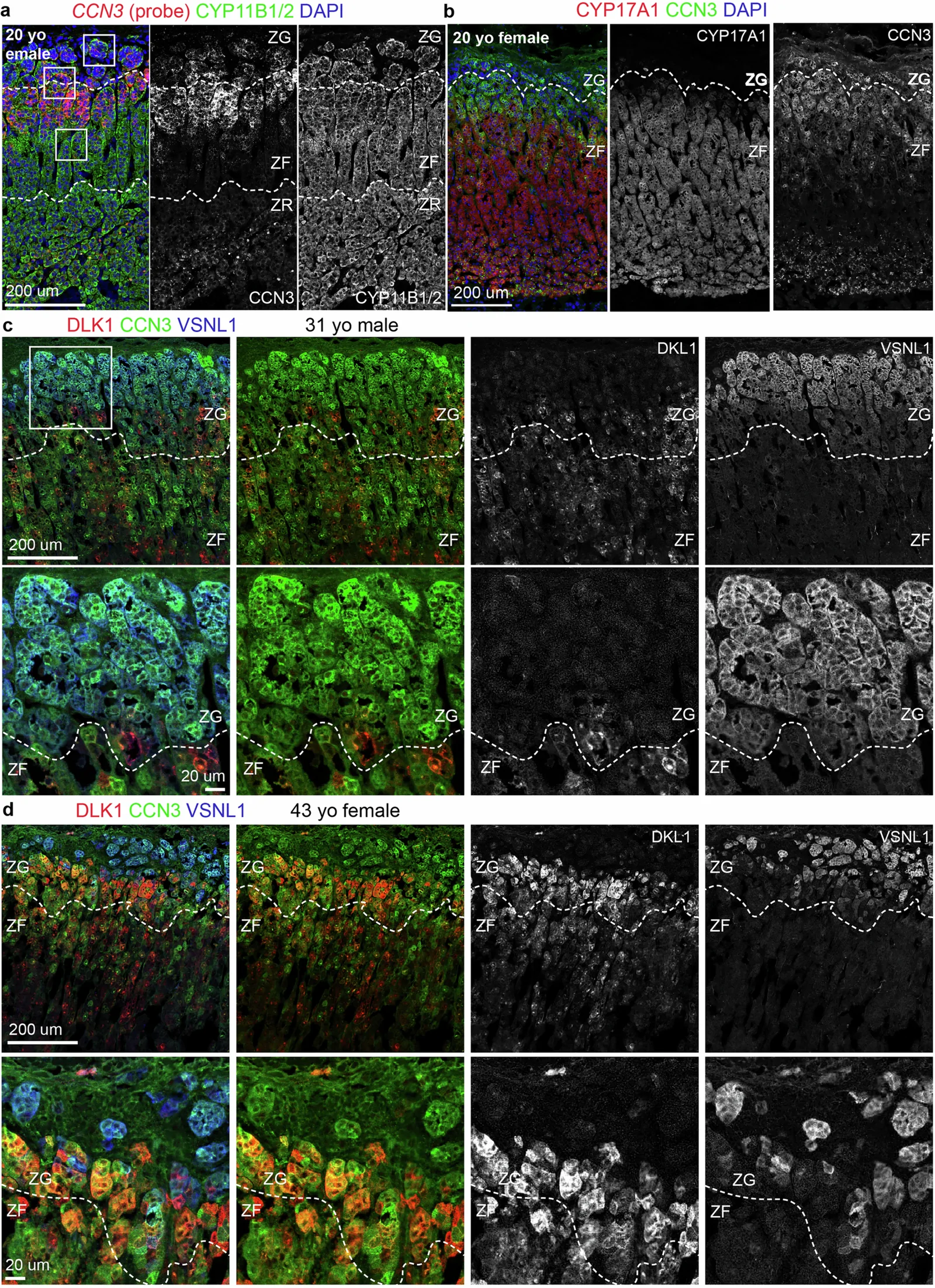
Validation of CCN3 and DLK1 in healthy human adult adrenal cortex. **a**. RNAscope *in situ* hybridization for *CCN3* combined with immunofluorescence for CYP11B1/2 showing *CCN3* enrichment in zona glomerulosa (ZG) and apical zona fasciculata (ZF) in the adrenal cortex from a 20-year-old female. **b**. Immunofluorescence against CYP17A1 (zona fasciculata/reticularis marker) and CCN3 shows partial overlap in apical zona fasciculata additionally to the CYP17A1^−^ zona glomerulosa. **c, d**. Immunofluorescence against DLK1, CCN3 and zona glomerulosa marker VSNL1 on adrenal cortex biopsies of a 31-year-old male and 43-year-old female respectively reveals CCN3 in ZG, CCN3 and DLK1 co-expression in ZG and apical ZF. Note CCN3^+^/VSNL1^+^ rosettes in apical ZG in 43 year old, and stronger DLK1 expression in basal ZG. Experiments repeated independently twice with similar staining patterns. ZG: zona glomeruolosa; ZF: zona fasciculata; ZR: zona reticularis.

*VCAN* histology revealed expression in *KCNJ5*^+^*CYP11B2*^+^ steroidogenic rosettes of ZG and negligible levels in upper *HSD3B2*^+^ ZF (Supplementary Fig. 10). *DLK1*^high^ and *CCN3*^high^ clusters were characterized by a *CYP11B2*^−^*CYP17A1*^low^*CYP11B1*^low^ signature at lower levels than confidently ZF-assigned clusters, suggesting that they may represent ZG-to-ZF transition, supported by *CCN3*^high^ cell detection within the ZG and ZF (Fig. 3a,b, Supplementary Fig. 8 and Extended Data Fig. 3a–c). DLK1 immunostaining was enriched within ZF in young donors but shifted toward ZG in older (>40 years old) individuals, with several cells co-expressing DLK1 and CCN3, supporting their classification as a ZG–ZF hybrid cluster (Extended Data Fig. 3d,e).

A *DCN*^high^ cluster with low *CYP17A1*, *CYP11B1*, *CYP21A2* and *HSD3B2* levels linked the capsule (cluster 1, mesenchyme) to ZG (Fig. 2a,c). Due to placement adjacent to *DLK1*^high^ and *CCN3*^high^ clusters of ZG, we annotated these cells as a transitional population (TRANS-DCN, Fig. 3a). ZF cells were connected to ZG smoothly, with gradual gene expression changes, separated into a central *CYP11B2*^−^*CYP17A1*^high^*CYP11B1*^+^ population and two populations of *NEAT*^high^ and *CPB1*^high^ cells (Fig. 3a,b).

**Extended Data Fig. 4:**
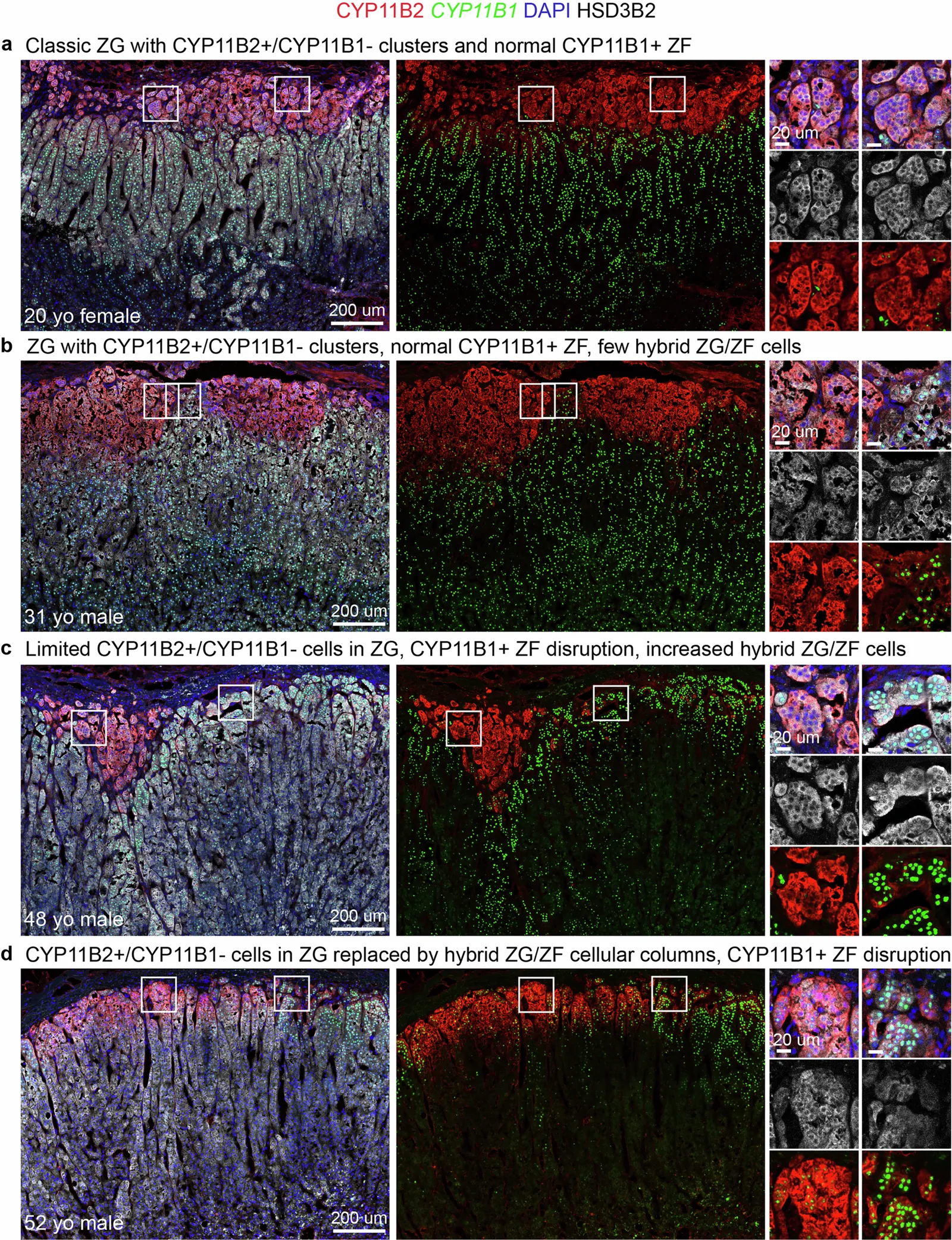
Validation of HSD3B2^+^/*CYP11B1*^+^/CYP11B2^−^ steroidogenic cells within human adult zona glomerulosa increasing with age. **a**-**d**. Immunodetection for CYP11B2 and HSD3B2 combined with RNAscope *in situ* hybridization for *CYP11B1* mRNA on adrenal cortex biopsies of a 20-year-old female, 31-year-old, 48-year-old and 52-year-old male respectively reveals several HSD3B2^+^/*CYP11B1*^+^/CYP11B2^−^ cellular clusters in zona glomerulosa, increasingly with age, corresponding to plausible involvement in glucocorticoid synthesis. Stainings for CYP11B2 and HSD3B2 repeated independently three times with reproducible findings. ZG: zona glomerulosa; ZF: zona fasciculata.

We identified a steroidogenic population with higher *HSD3B2* expression levels than other ZG and ZF cells^4,9,11^ (cluster ZG-HSD3B2, Fig. 3a,b), co-expressing *HSD3B2*, *CYP21A2*, *CYP17A1* and *CYP11B1* but lacking *CYP11B2*. This suggests their involvement in direct cortisol production within the ZG, supported by a previous study^12^, and expanded ZF marker expression in ZG linked to aging^13^. Histological confirmation of coexpression of *HSD3B2* and *CYP21A2* and *HSD3B2*^+^*CYP11B1*^+^CYP11B2^−^ cells within ZG rosettes in older donors (Extended Data Fig. 4 and Supplementary Fig. 11) challenged the assumption that *CYP11B1* expression is exclusive to ZF (Fig. 2e).

Using Stereo sequencing (Stereo-seq)^14^ on three donors (aged 13–41 years), we reconstructed zonal subclusters on tissue sections (Fig. 3c,d and Supplementary Figs. 12–15). Apical ZG was dominated by *VCAN*^high^, *CCN3*^high^ and *CYP11B2*^high^ populations; *DLK1*^high^ cells expanded basally in younger donors and were restricted to the ZG in the older individual and *HSD3B2*^high^ cells occupied expanded subcapsular domains (Fig. 3d and Supplementary Figs. 13 and 14). Within the ZF, *NEAT1*^high^ and *CPB1*^high^ populations were found in apical and basal positions, respectively (Supplementary Figs. 13 and 14).

The ZG-CHOL cluster, marked by endogenous cholesterol biosynthesis genes, bridging the ZG and ZR, showed compositional differences between sexes (Fig. 3e,f,i,j and Supplementary Fig. 16d–f), with higher cholesterol biosynthesis pathway activity in male ZG and ZF compared to female (Supplementary Fig. 17). Quantification of cholesterol acquisition, usage and esterification (Supplementary Fig. 18) across cortical zones and sexes extended to a cohort of 274 individuals^15^ and modeled using a mixed linear model (Supplementary Figs. 19 and 20) revealed increased inferred cholesterol balance activity in male ZG or ZF (Fig. 3g,h and Supplementary Figs. 19e, 20c and 21–25), suggesting a surplus exceeding steroidogenesis demand.

### Mechanisms of human adult adrenal cortex cell renewal

**Fig. 4:**
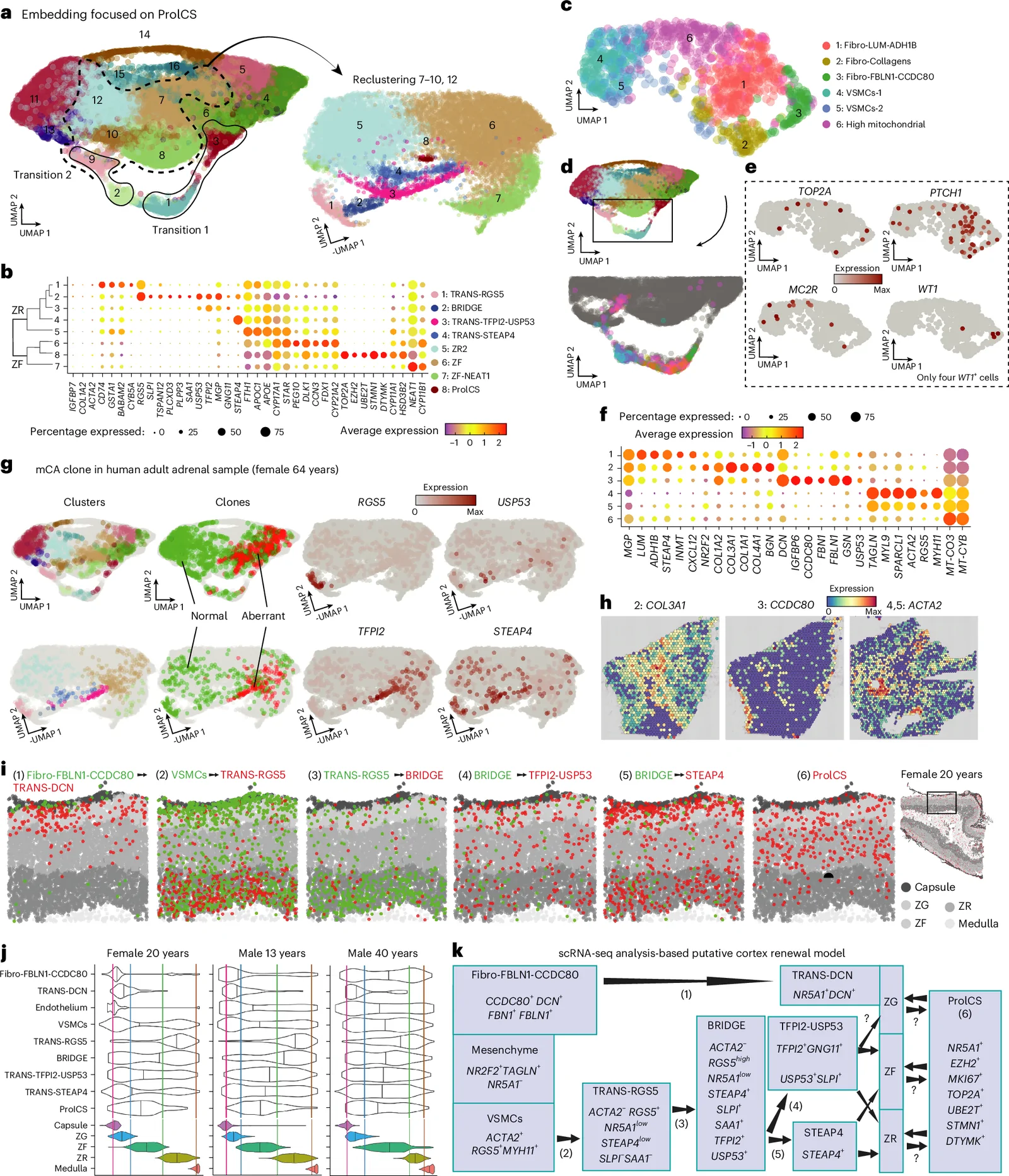
Fine-cell populations and transitions in adult adrenal renewal. **a**, Focused re-analysis of clusters 7–10 and 12 from the main embedding (left, 39,985 cells) at higher resolution (right, 16,926 cells). Original clusters 9, 12, 7 and 8 retain their colors; original cluster 10 is resolved into new clusters 2–4. **b**, DotPlot of cluster-specific marker genes and steroidogenic enzymes, with cluster hierarchy. **c**, UMAP of focused human adult mesenchymal cell reintegration (1,177 cells). **d**, Mapping of mesenchymal subclusters on to the main adrenocortical–mesenchymal UMAP. **e**, Expression of *TOP2A*, *PTCH1*, *MC2R* and *WT1* in adult mesenchymal cells. **f**, DotPlot of mesenchymal subpopulation marker genes. **g**, The mCA clonal analysis in one female donor (aged 64 years) identifying an aberrant clone with chr. 13 or chr. 22 deletions and chr. 8 or chr. 9 amplifications (Supplementary Fig. 31). Normal and aberrant cells are shown on the main and focused UMAPs; the *TFPI2*^+^ intermediate state expression supports VSMC-to-ZF or VSMC-to-ZG transition. **h**, Visium expression of mesenchymal subpopulation markers. **i**, Renewal-associated cell types in Stereo-seq data (8,420-cell representative area). The source and target cell types are indicated above each panel. **j**, Violin plots of renewal cell-type spatial distribution from capsule to medulla, with zone boundaries indicated. **k**, Scheme of human adrenocortical renewal depicting transitions from mesenchymal subpopulations and ProlCS cells. Max, maximum.

**Extended Data Fig. 5:**
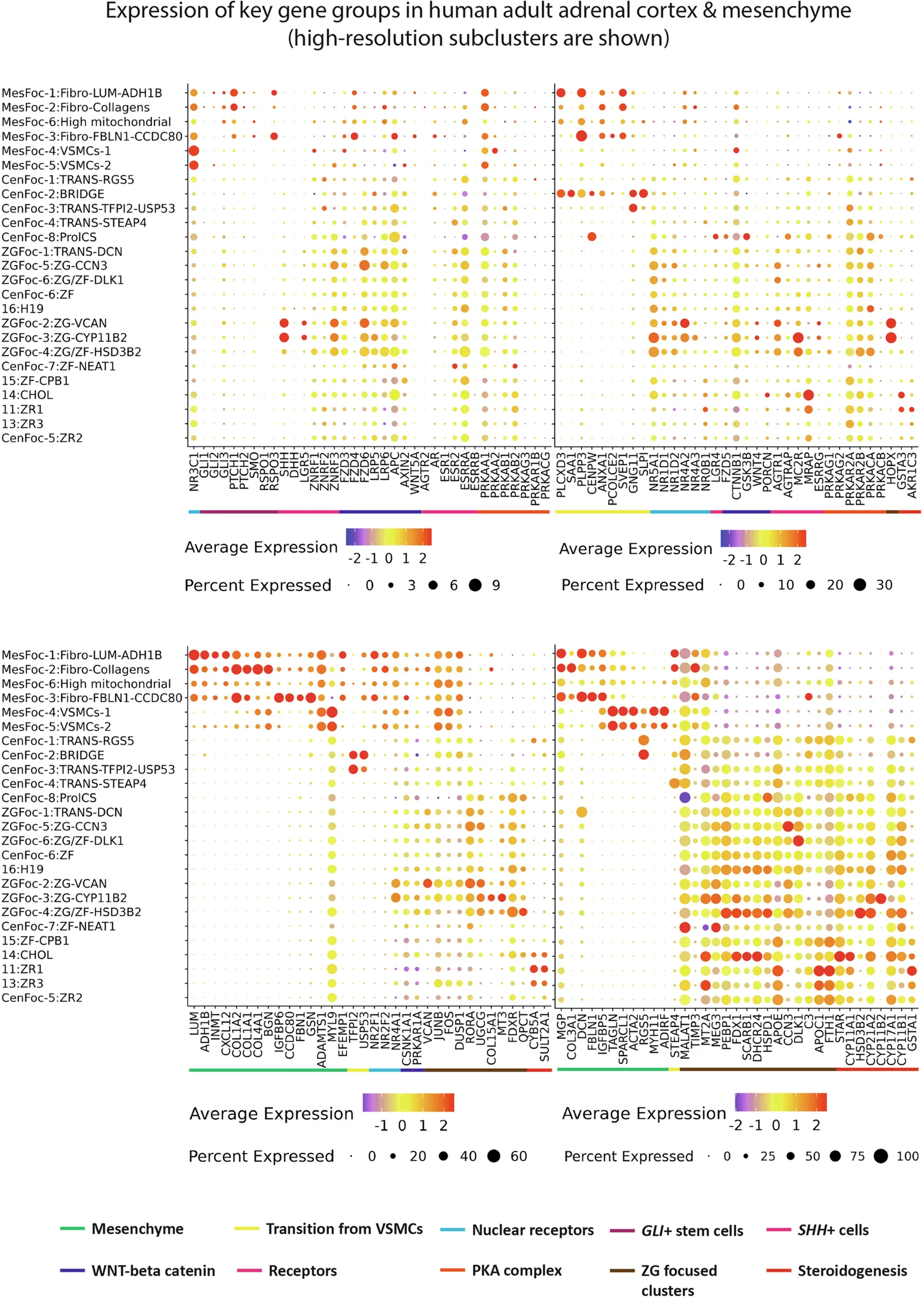
Expression of key gene groups in human adult adrenal cortex and mesenchyme. DotPlots of genes involved in putative pathways of adult adrenal renewal regulation, divided into four groups by maximum percentage of expressing cells in any cluster (e.c. for genes on the first DotPlot maximal percent positive cells in any cluster is 10%). Clusters include subpopulations from the adrenocortical and mesenchymal integration (Fig. 2a,c), focused zona glomerulosa (ZG) reclustering (prefix ZGFoc-, Supplementary Fig. 8), central part of the embedding and proliferating cortical state (ProlCS) cells (prefix CenFoc-, Fig. 4a), and mesenchyme (prefix MesFoc-, Fig. 4c). VSMCs: vascular smooth muscle cells; ZG: zona glomerulosa; ZF: zona fasciculata; ZR: zona reticularis; CHOL: cholesterol.

Next, we focused on cells involved in renewal and mesenchymal cells including the capsule (Fig. 4a,b and Supplementary Figs. 26 and 27), to construct a data-driven hypothesis on cortical dynamics. We subclustered adjacent clusters of *NR5A1*^+^ cells relevant to transitions from mesenchyme (clusters 7–10 and 12; Fig. 4a–e and Supplementary Table 4). Analysis of cell states and proliferation signatures suggests three potential sources of steroidogenic cells: (1) transition 1 (from fibroblasts or capsule); (2) transition 2 (from vascular smooth muscle cells (VSMCs)); and (3) proliferating cortical *NR5A1*^+^*EZH2*^+^ cells that lack mesenchymal features (proliferating cortical state (ProlCS) cells) hinted by bromodeoxyuridine labeling in mouse adrenals^16^ (Fig. 4a, Supplementary Figs. 26 and 27 and Extended Data Fig. 5).

Inferred transition 1 corresponds to *GLI1*^+^*PTCH1*^+^ capsular mesenchymal stem cells differentiating toward ZG^17–19^ (Extended Data Fig. 5), characterized by *Wt1* expression in rodents. Of note, *WT1*^+^ cells are found in low numbers in the adult human cortex (Fig. 4c–f). We examined the transition from cluster 1 (capsule, *CCDC80*^+^*FBN1*^+^*FBLN1*^+^*DCN*^+^*NR5A1*^−^ cells mapped through spatial transcriptomics to the outer surface of the gland (Fig. 4h)) toward cells of cluster 3 (ZG, *NR5A1*^+^*DCN*^+^ cells), but failed to find intermediate states with exclusive markers, suggesting that this mechanism is not extensively utilized in the healthy human adult gland (Supplementary Fig. 28).

Inferred transition 2 (Supplementary Fig. 29) from *ACTA2*^+^*RGS5*^+^*MYH11*^+^ VSMCs contributes through an *RGS5*^+^*ACTA2*^−^ state to the ZF and ZR (according to the Uniform Manifold Approximation and Projection (UMAP) representation of single-cell transcriptomes) and possibly to the ZG. We depicted this intermediate step from *RGS5*^+^*ACTA2*^−^ cells (cluster 9) to *STEAP4*^+^*TFPI2*^+^ cells (cluster 10) and then to the ZG as inferred transition 3 (Supplementary Fig. 30). VSMCs are present in the capsule and the upper ZG and between the ZR and medulla. According to observed *ACTA2*, *RGS5*, *PLCXD3* and *STEAP4* expression in single cells and Visium in VSMCs and steroidogenic cells (and other genes shown in Supplementary Figs. 26 and 27), this transition may drive renewal of the ZR and the ZG or ZF. We previously identified an individual carrying mosaic chromosomal alterations (mCAs), which can serve for cellular lineage reconstruction^20^ (Fig. 4g and Supplementary Fig. 31). The pattern of mCAs in this sample, largely composed of genetically normal ZR cells (only three aberrant cells within the ZR), and aberrant VSMCs, ZG and ZF cells supports the inferred transition from VSMCs through *TFPI2*^+^*GNG11*^+^ cells differentiating toward cells of the ZF and ZG. The VSMC-to-intermediate *USP53*^+^ state predicted to give rise to the ZR does not contain genetically aberrant cells, thus it does not provide evidence for this transition. Cluster 3:TRANS-DCN contained a high fraction of aberrant cells, indicating the potential presence of aberrant fibroblasts and supporting the transition from 1:fibroblasts toward transient cluster 3:TRANS-DCN. Finally, predominant contribution of the aberrant clone to the ZG and ZF supports the centripetal model of differentiation from the ZG toward the ZF^8,16,21^.

As a third potential renewal source, we observed a cycling *NR5A1*^+^ population (ProlCS—cluster 8 on focused UMAP; Fig. 4a), expressing genes associated with chromatin remodeling, DNA repair, chromosome and cytoskeletal rearrangements (*EZH2*, *UBE2T*, *STMN1* and *DTYMK*) and proliferation (*TOP2A*, *MKI67* and *CENPM*). Based on cluster location in the center of the UMAP, we hypothesized its contribution to all cortical zones. RNA velocity analysis (Supplementary Fig. 32) produced variable outcomes across samples, despite a conserved pattern of directional differentiation from the center of the UMAP (position of dividing cortical cells) to the periphery (right, ZG and ZF; left, ZR).

**Extended Data Fig. 6:**
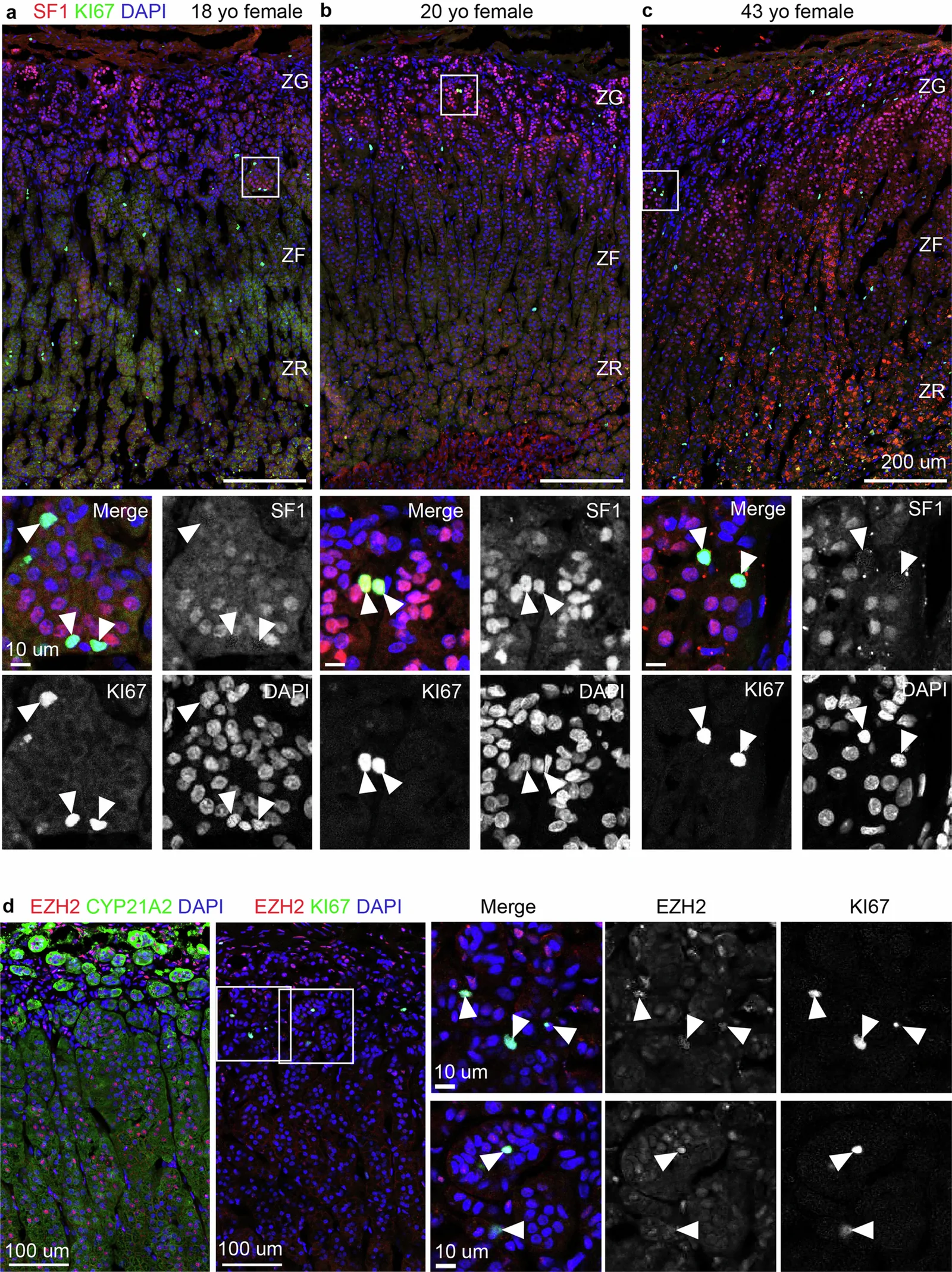
Proliferating cells throughout the human adult adrenal cortex. **a, b, c**. SF1 (encoded by *NR5A1*) and KI67 immunofluorescence showing steroidogenic cells (evident by autofluorescence in characteristic rosette-like and columnar-like structures) in adrenal cortex biopsies from a 18-year-old (**a**), 20-year-old (**b**) and 43-year-old (**c**) female respectively. Arrowheads in insets show KI67^+^ proliferating cells. Note that SF1 is not detected in the majority of cycling cells within the cortex, apart from a rare case in the biopsy of the 20-year-old female. **d**. Immunofluorescence against EZH2 and either pan-steroidogenic marker CYP21A2 (left panel) or KI67 (right panel) on an adrenal cortex biopsy of a 20-year-old female reveals co-expression in numerous cells within zona glomerulosa and extensive nuclear EZH2 immunoreactivity. Stainings performed on multiple donors once. ZG: zona glomerulosa; ZF: zona fasciculata; ZR: zona reticularis.

To validate these transitions and their spatial enrichment, we questioned our high-resolution spatial transcriptomics data (Stereo-seq). Cycling *NR5A1*^+^ cells (referred to as ProlCS cells) were distributed throughout the cortex (Supplementary Fig. 33), supported by KI67, SF1(*NR5A1*) immunofluorescence (Extended Data Fig. 6). The transition from capsular mesenchyme (fibro-FBLN1-CCDC80 cells) to the ZG with transient *DCN* expression (TRAN-DCN) was enriched in the ZG and the *TFPI2* or *STEAP4* transient state hypothesized to contribute to the ZF was enriched in the ZF (Fig. 4i,j and Supplementary Fig. 15). The transition from VSMCs to the ZF or ZR through the transient *RGS5* state was enriched in the ZR, decreasing in the ZF (Fig. 4i,j and Supplementary Fig. 15). Our findings suggest a revised model of human adrenocortical turnover that goes beyond the classic centripetal model from the ZG to the ZF and subsequently the ZR (Fig. 4k).

### Comparison of adult and fetal human adrenal cortex

To examine adult adrenocortical renewal compared to developmental renewal, we reanalyzed published data from human adrenocortical primordium^22–25^ (7–21 weeks after fertilization, Carnegie stages 16 or 17 onward and 6 days postnatally (Supplementary Figs. 34 and 35)), reproducing described transition from Schwann cell precursors (SCPs) to chromaffin cells and SCPs to sympathoblasts^22,26^ (Supplementary Fig. 36a–c).

**Fig. 5:**
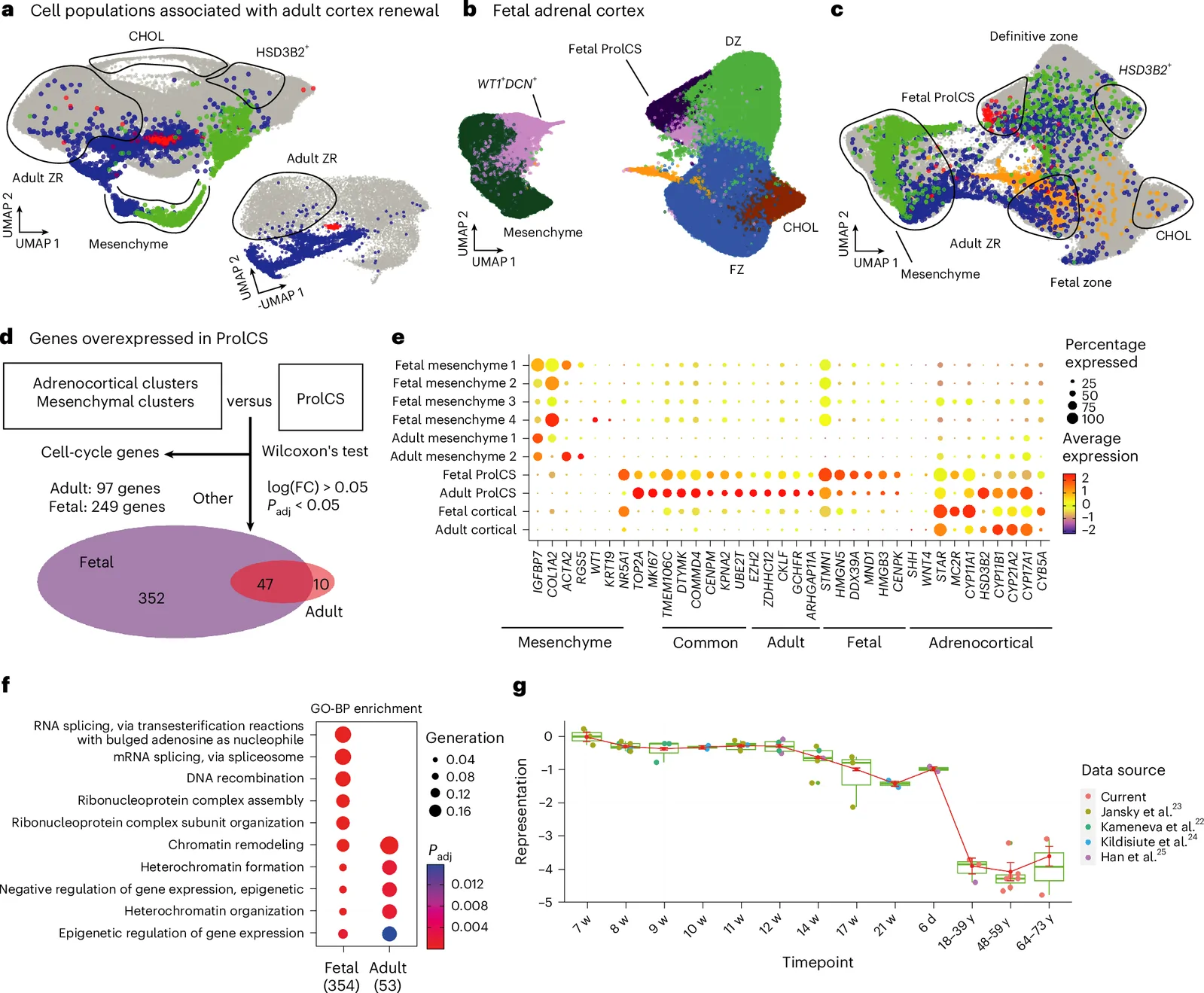
Comparative analysis of adult human adrenocortical renewal and fetal development. **a**, Key renewal populations on the main UMAP (left, 39,985 cells) and central region (right): transition 1 (fibroblasts, green), transition 2 (VSMCs, blue) and ProlCS (red). **b**, UMAP of integrated fetal and newborn adrenocortical and mesenchymal cells from four published datasets^22–25^ (130,911 cells; Supplementary Fig. 34). **c**, Adult renewal populations overlaid on fetal–newborn–adult integration: ProlCS (red), transition 1 (green), transition 2 (blue) and fetal mesenchyme-to-cortex transition (orange). **d**, Venn diagram of ProlCS marker genes in adult versus fetal datasets after exclusion of cell-cycle genes (differential expression by two-sided Wilcoxon’s test, with BH adjustment, log(fold-change) (log(FC)) > 0.05, *P* < 0.05). **e**, DotPlot of ProlCS-specific markers and transcriptional differences between adult and fetal ProlCSs, including mesenchymal and adrenocortical reference genes. **f**, Gene set overrepresentation analysis (gene ontology (GO) biological process (BP)) of adult and fetal ProlCS marker genes from **d**. One-sided hypergeometric test with the BH adjustment. **g**, Temporal dynamics of ProlCS representation across fetal (7–21 weeks after conception, *n* = 35), newborn (6 days, *n* = 2), and adult (18–73 years, *n* = 12) samples^22–25^. For each box plot, the center line is the median, the box limits the upper and lower quartiles and the whiskers 1.5× the IQR. The red dots and error bars are the mean ± s.e.m. from Poisson’s linear mixture model. w, week; y, year; d, day.

**Extended Data Fig. 7:**
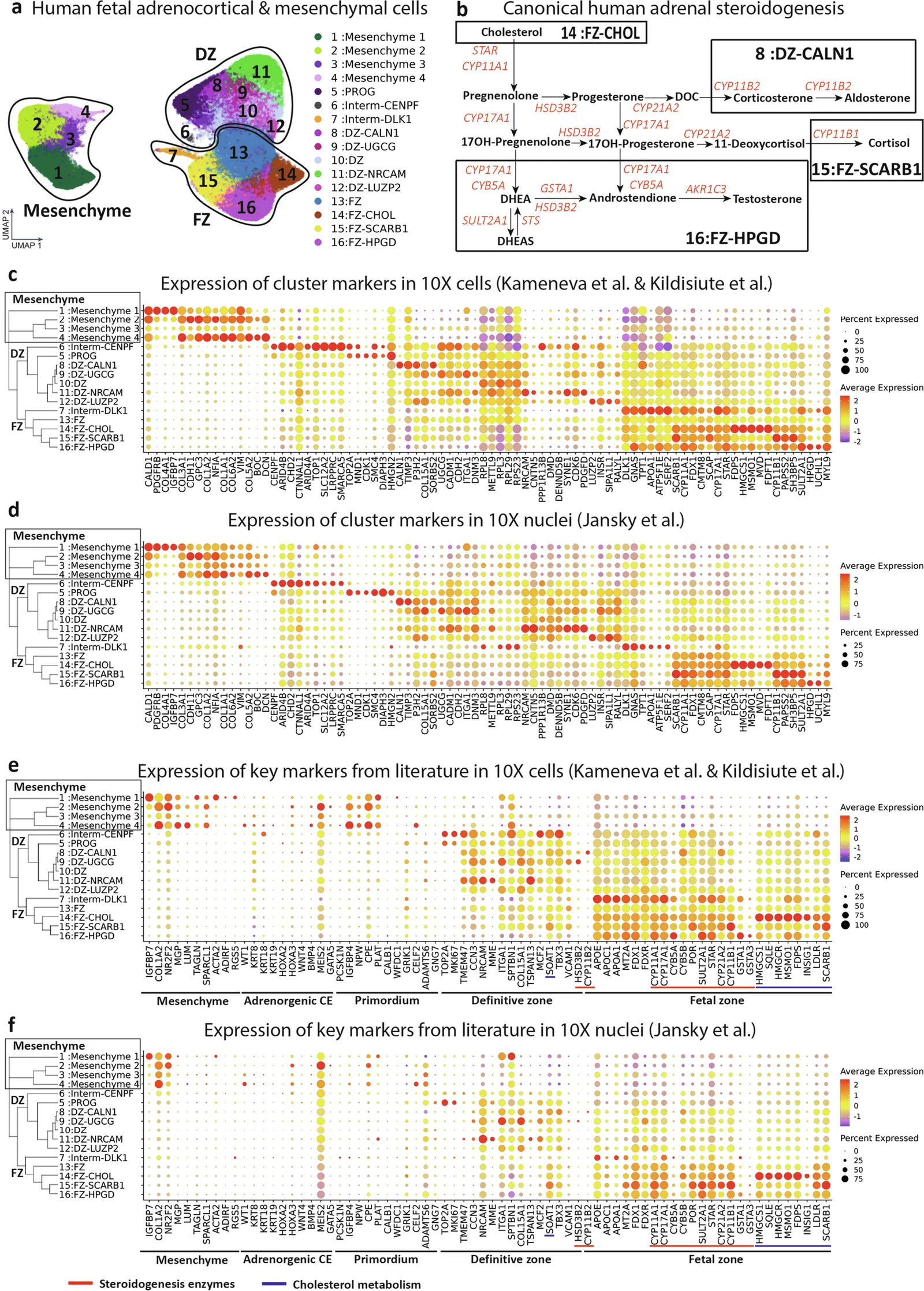
Transcriptional heterogeneity of the human fetal adrenal cortex. **a**, UMAP of integrated fetal adrenocortical and mesenchymal cells with zonation indicated (130,911 cells). **b**, Schematic of steroidogenesis in the human adrenal gland with cluster-specific pathway specializations. **c, d**, DotPlots of cluster-specific marker genes with cluster hierarchy based on transcriptional similarity. **c**, 10x single cell data (53,140 cells). **d**, 10x single nuclei data (69,723 cells). **e-f**, DotPlots displaying literature markers for key fetal adrenal compartments, steroidogenic enzymes (underlined red) and cholesterol metabolism genes (underlined blue). **e**, single cell data. **f**, single nuclei data. CE: coelomic epithelium; DZ: definitive zone; FZ: fetal zone; CHOL: cholesterol.

**Extended Data Fig. 8:**
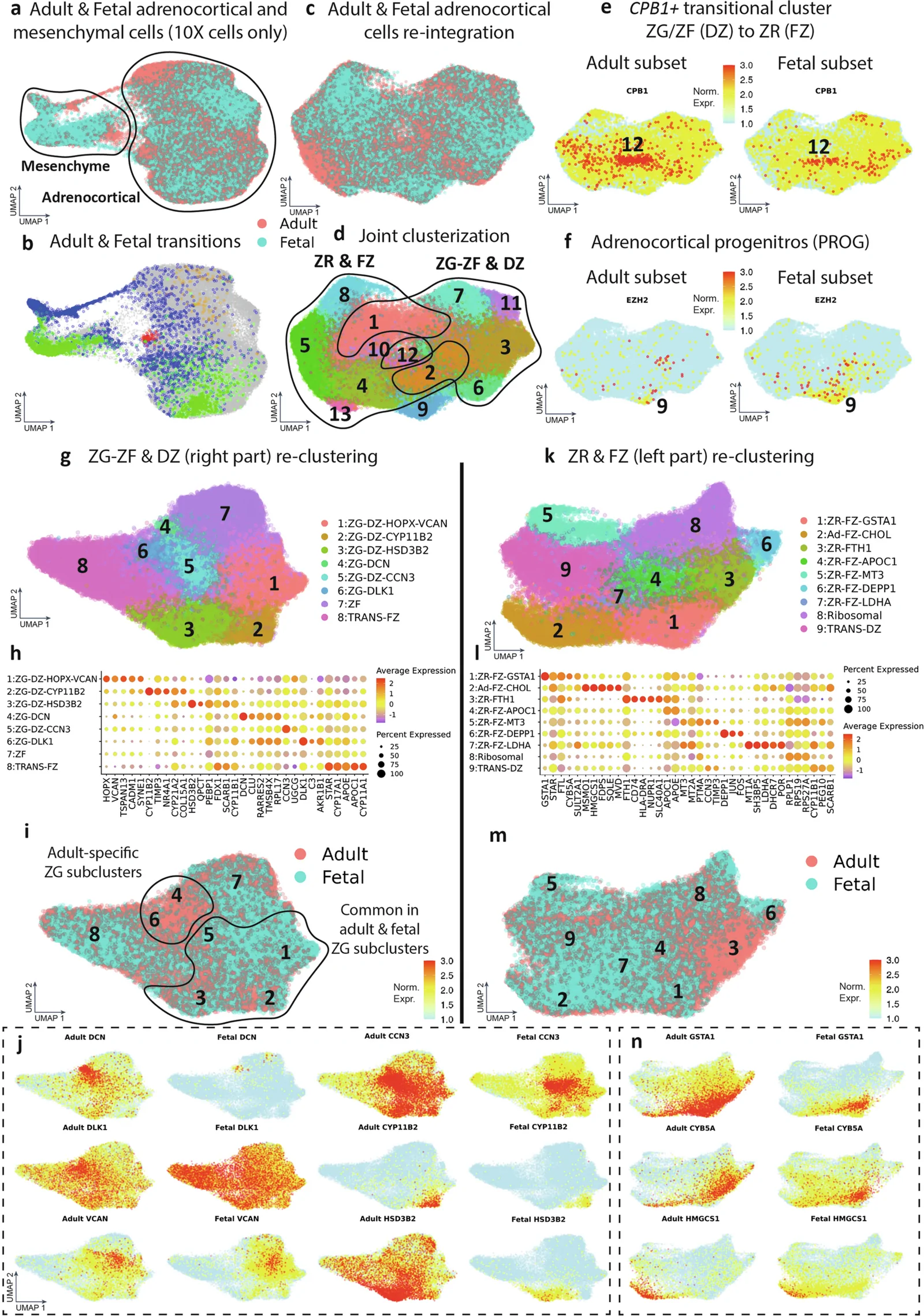
Human adult and fetal adrenocortical cell integration. **a,b**, UMAP embedding of integrated adult (39,985 cells) and fetal (53,140 cells) adrenocortical and mesenchymal cells (93,125 total). **a**, Adult (red) versus fetal (blue) compositional contribution. **b**, Adult renewal populations and transitions overlaid on adult-fetal integration: proliferating cortical state (ProlCS, red), transition 1 (from Fibroblasts) in green, transition 2 (from VSMCs) in blue, fetal mesenchyme-to-cortex transition in orange. c-f, UMAP of re-integrated adult (36,924 cells) and fetal (43,466 cells) adrenocortical cells (80,390 total). **c**, Adult/fetal compositional contribution. **d**, Joint clustering with areas selected for reclustering. **e**, *CPB1* expression in adult (left) and fetal (right) subsets. **f**, *EZH2* expression in adult and fetal subsets. **g–j**, Re-integration of adult ZG/ZF (22,043 cells) and fetal DZ (26,906 cells) cells. **g**, Joint clustering. **h**, DotPlot of cluster marker genes. **i**, Adult/fetal compositional contribution. **j**, Expression of adult ZG markers (*DCN, CCN3, DLK1, VCAN, CYP11B2, HSD3B2*) in adult (left) and fetal (right) subsets. **k–n**, Re-integration of adult ZR (18,770 cells) and fetal FZ (21,160 cells) cells (39,930 total). **k**, Joint clustering **l**, DotPlot of cluster marker genes. **m**, Adult/fetal compositional contribution. **n**, Expression of adult ZR markers (*GSTA1, CYB5A*) and cholesterol biosynthesis marker (*HMGCS1*) in adult and fetal subsets. ZG, zona glomerulosa; ZF, zona fasciculata; ZR, zona reticularis; DZ, definitive zone; FZ, fetal zone; ProlCS, proliferating cortical state.

We observed established fetal cortical zones: the definitive zone (DZ) composed of *HSD3B2*^+^*CYP11B1*^+^*CCN3*^+^ cells, *CYP17A1*^+^*SULT2A1*^+^ fetal zone (FZ) with high levels of *LDLR*^27,28^ and a population of *HSD3B2*^high^ cells reflecting a transitional zone^27,29^ (Fig. 5b, Extended Data Fig. 7a–d and Supplementary Table 5). We combined fetal and adult cells focusing on the populations or transitions relevant to adrenocortical turnover in the adult and confirmed partial homology (all data: Fig. 5a,c and Supplementary Figs. 37 and 38; only 10× cells: Extended Data Fig. 8). Fetal DZ included proliferating cells (5:ProlCS) and showed similarity to adult ZG, whereas underrepresented *CYP11B2*^+^ and *HSD3B2*^+^ fetal clusters resembled corresponding adult ZG clusters (Extended Data Figs. 7c–f and 8j and Supplementary Table 6). We observed the absence of *DCN* and lower expression and numbers of *CCN3*^+^ cells in the human fetal cortex compared to the adult and increased *DLK1*^+^ cell numbers in the fetal cortex versus the adult (Extended Data Fig. 8g–j). ZR marker genes *GSTA1* and *CYB5A* were also less prevalent in the fetal cortex (Extended Data Fig. 8n).

The FZ in our compiled dataset consists of clusters (15: FZ-SCARB1 and 16:FZ-HPGD) with either increased *CYP11B1* expression (glucocorticoid production) or androgen-related enzymes (that is, *CYP17A1* and *SULT2A1*), respectively (Extended Data Fig. 7) and a cluster enriched in endogenous cholesterol biosynthesis (14: FZ-CHOL) (Extended Data Fig. 7). We detected sexual dimorphism associated with this cluster, overrepresented in females (Supplementary Fig. 39d), consistent with compositional overrepresentation of adult female cholesterol synthesis in the adult ZR.

We compared proliferating and stem cells in human fetal and adult adrenal glands (Fig. 5a,c). Fetal mesenchyme analysis revealed predominant expression of *PTCH1* and *RSPO3* and a *WT1*^+^*DCN*^+^ mesenchyme subpopulation (Supplementary Fig. 40a–d). Compositional dynamics showed a gradual decrease of mesenchymal clusters and adrenocortical mesenchymal progenitors, a gradual increase of FZ cell numbers (Supplementary Fig. 41) and a near absence of *WT1*^+^ cells in adult adrenals (Fig. 4e). Comparisons of the mesenchymal, cortical and mesenchyme-to-cortex transitions between adult and fetal adrenal glands showed that transitions from *ACTA2*^+^*RGS5*^+^ perivascular mesenchyme (shown in blue) and *DCN*^+^ mesenchyme (shown in green) occur only in adult adrenals and transcriptomic differences between *WT1*^+^ fetal and adult mesenchyme (Fig. 5c,e).

Finally, we comparatively examined the top genes of cortical cycling *NR5A1*^+^*EZH2*^+^ cells in fetal and adult human adrenals (ProlCS cells) (Fig. 5d,f). Differentially expressed (DE) genes were numerous, with fetal proliferating cells differentially expressing genes related to messenger RNA splicing and ribosomal assembly, whereas fetal and adult cells shared genes dedicated to chromatin remodeling and gene expression regulation, and fetal ProlCS expressed higher levels of *NR5A1* (gene coding for SF1) and *MC2R* (coding for the adrenocorticotropic hormone receptor) (Fig. 5e,f). The quantification of proliferating *NR5A1*^+^*EZH2*^+^ cell density showed a gradual decrease as development progressed, with especially low numbers in the adult (Fig. 5g).

Adult renewal involves mesenchyme-to-steroidogenic transitions (adrenal capsule-derived progenitors), contrasting with the fetal cortex 6–7 weeks after conception and onwards, a time during which direct mesenchyme-to-steroidogenic transitions are restricted, and a bridging population between the two is less identifiable (Extended Data Fig. 7a). This is in agreement with studies recapitulating human cortex development and subsequent expansion of the fetal cortex through proliferation, rather than mesenchymal-to-steroidogenic transitions^27,30^.

### Comparison of human and mouse adult adrenal cortex

To uncover interspecies differences, we generated scRNA-seq data from 6-week-old to 8-week-old male and female adult mice, combined with existing datasets^25,31^ (Supplementary Figs. 42 and 43). We identified adrenal cortical cells, sustentacular glia, adrenergic *PNMT*^+^ and noradrenergic *PNMT*^−^ chromaffin cells of the medulla, connective tissue, immune and endothelial cells and confirmed their general similarity (Extended Data Fig. 1 and Supplementary Fig. 44). We then carried out high-resolution Visium HD spatial transcriptomics of adult mouse adrenal glands from 8-week-old males and females (Supplementary Figs. 45–48).

**Fig. 6:**
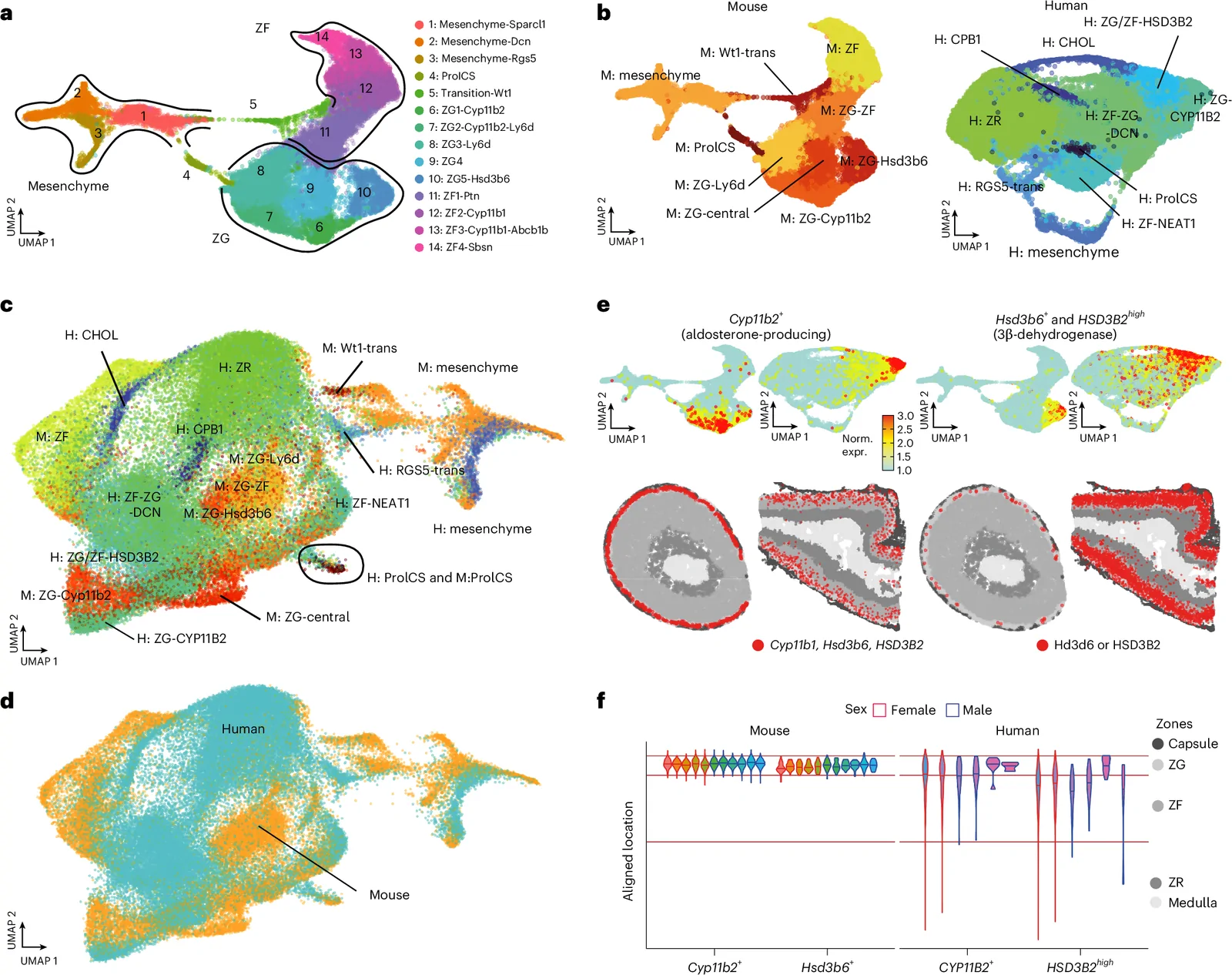
Interspecies comparison of adult adrenal cortex between humans and mice. **a**, UMAP of mouse adrenocortical and mesenchymal cells with zonation indicated (25,932 cells). **b**, Cluster annotation on mouse (left, 25,932 cells) and human (right, 39,985 cells) UMAPs, as mapped on to the integrated embedding in **d**. **c**, Interspecies integration UMAP (65,917 cells): mouse clusters in red–yellow, human clusters in blue–green. Cluster names are prefixed M or H. **d**, Contribution of human and mouse cells to the integrated embedding. **e**, The two transcriptionally conserved cell populations: aldosterone-producing *Cyp11b2*^+^ and *CYP11B2*^+^ cells and *Hsd3b6*^+^ and *HSD3B2*^high^ 3β-hydroxysteroid dehydrogenase-expressing ZG cells. Key marker expression shown in scRNA-seq and spatial data (Stereo-seq for human, Visium HD for mouse). One female slide per species is shown. **f**, Spatial distribution of homologous cell states across all samples after coordinate transformation equalizing ZG and ZF sizes between species. Norm. expr., normalized expression.

Subselection and reclustering of adrenocortical and mesenchymal or perivascular murine populations (25,932 cells) facilitated further analysis of cellular transitions and comparison to human cortex (Fig. 6a–f and Supplementary Table 7).

**Extended Data Fig. 9:**
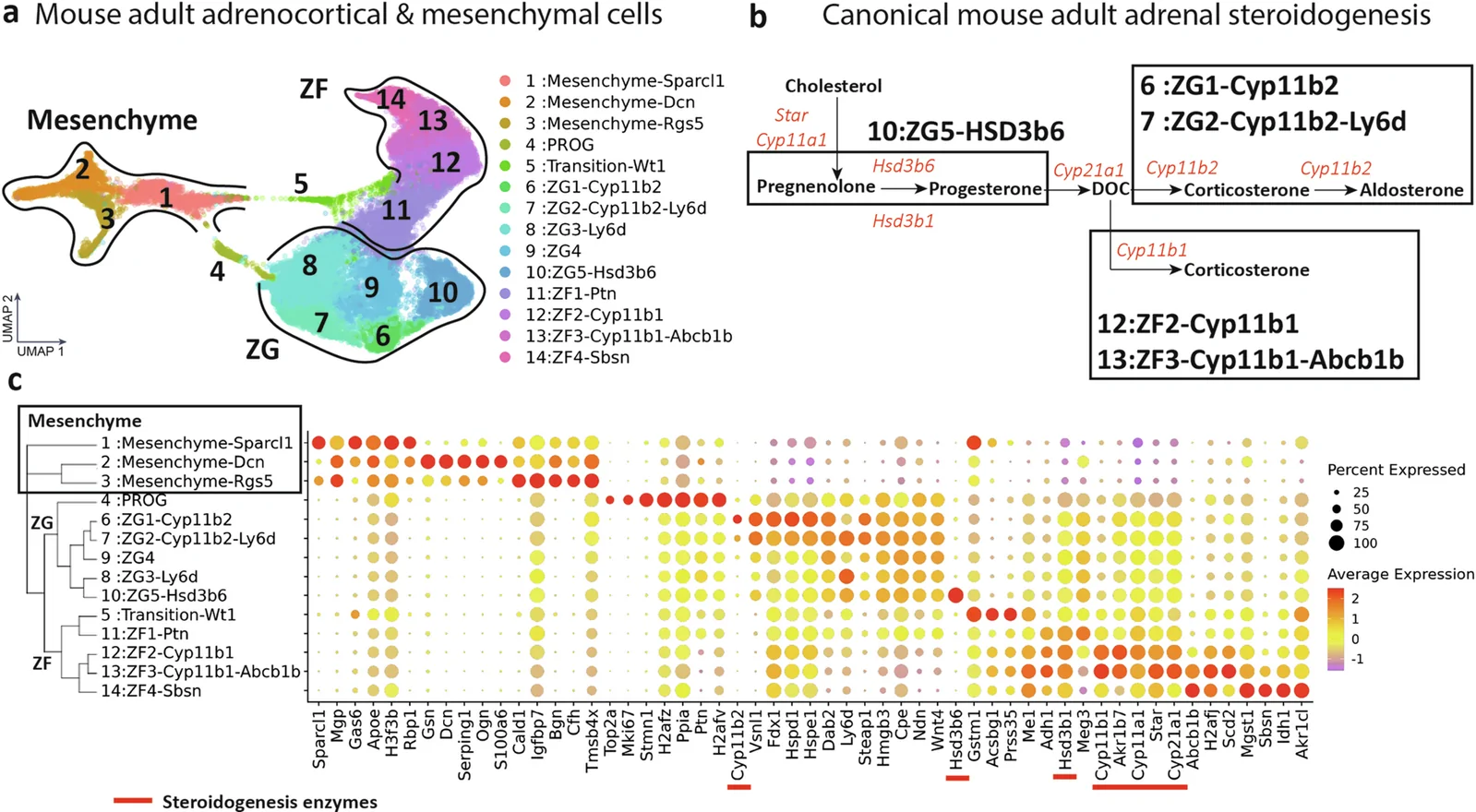
Transcriptional heterogeneity of adult mouse adrenal cortex. **a**, UMAP of reintegrated mouse adrenocortical and mesenchymal cells with zonation indicated (25,932 cells). **b**, Schematic of steroidogenesis in the mouse adrenal gland with cluster-specific pathway specializations. **c**, DotPlot of cluster-specific marker genes and steroidogenic enzymes (underlined red) with cluster hierarchy. ZG: zona glomerulosa; ZF: zona fasciculata; ProlCS: proliferating cortical state cells.

Murine ZG (clusters 6–10) consisted of *Dab2*-expressing or *Wnt4*-expressing clusters characterized by mutually exclusive expression of *Hsd3b6* (cluster 10, glucocorticoid producing) and *Cyp11b2* (clusters 6–9, aldosterone synthesizing), whereas ZF (clusters 11–14) consisted of *Hsd3b1*-expressing or *Cyp11b1*-expressing, glucocorticoid-synthesizing clusters (Extended Data Fig. 9a–c).

Among genes shared between ZG and ZF, we identified *Ptn* (secreted growth factor pleiotrophin), also highly expressed in cycling cortical cells. Sexual dimorphism was observed in murine ZF with higher levels of cholesterol biosynthesis activity in females (cluster 14, WP103: *P*_adj_ = 4.8 × 10^−5^; WP4346: *P*_adj_ = 1.4 × 10^−^^3^; Supplementary Fig. 49a–i). As expected due to *Cyp17a1* repression, coding for the key enzyme in androgen synthesis, 17α-hydroxylase^5,6^, no ZR was identified in the mouse cortex. Adrenocortical subclusters and corresponding marker gene distribution on Visium HD reproduced ZG/ZF zonation and the X zone (XZ) in nulliparous females (Supplementary Figs. 45–48).

**Fig. 7:**
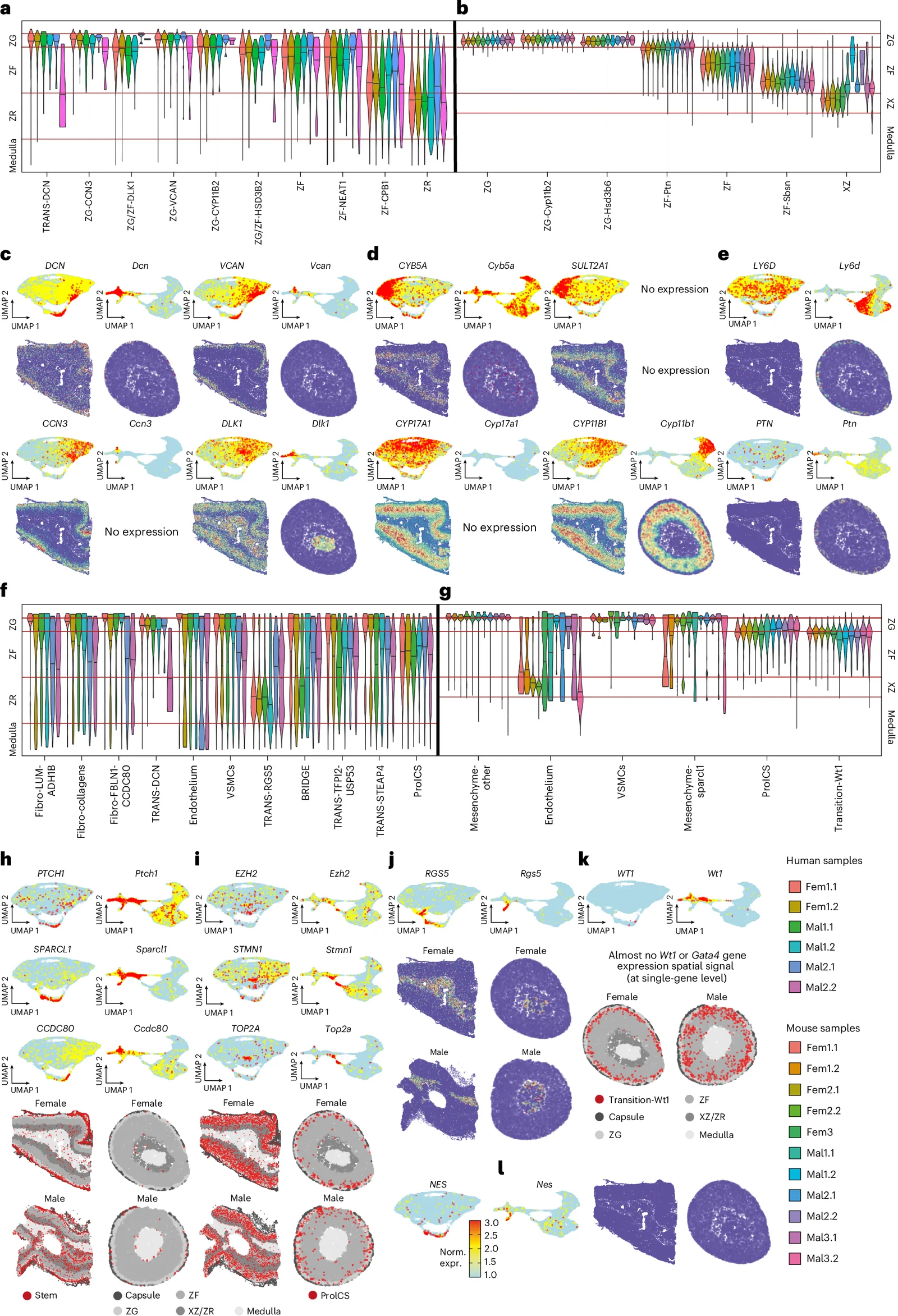
Systematic spatial interspecies comparison of the adult adrenal cortex. **a**,**b**, Spatial distribution of adrenocortical cell states in humans (**a**) and mice (**b**) across all samples after coordinate transformation enabling cross-species comparison. **c**–**e**, Expression of key heterogeneity markers in human (39,985 cells) and mouse (25,932 cells) scRNA-seq (upper) and representative female spatial slides (human 85,276 cells, mouse 11,313 cells, lower). **c**, *DCN*, *VCAN*, *CCN3* and *DLK1* human ZG markers largely absent in mouse cortex. **d**, ZR markers *CYB5A*, *SULT2A1* and *CYP17A1* absent in mouse and *CYP11B1* (ZF glucocorticoid synthesis) conserved. **e**, Mouse ZG markers *Ly6d* and *Ptn* showing no cortical specificity in humans. **f**,**g**, Spatial distribution of renewal-related cell states in humans (**f**) and mice (**g**), including endothelium and mesenchymal clusters of the capsular and perivascular niche. **h**–**k**, Key marker gene expression in scRNA-seq and spatial data (one female and one male slide per species). **h**, Capsule-localized mesenchymal stem cells. **i**, ProlCS cells. **j**, Human-specific *RGS5*^+^ VSMC-to-adrenocortical transition. **k**, Mouse-specific *Wt1*^+^ stem cell transition. The transition–*Wt1* state was detectable in mouse spatial data despite low *Gata4* expression. **l**, *NES* (NESTIN) expression showing no direct association with adrenocortical renewal in either species.

**Extended Data Fig. 10:**
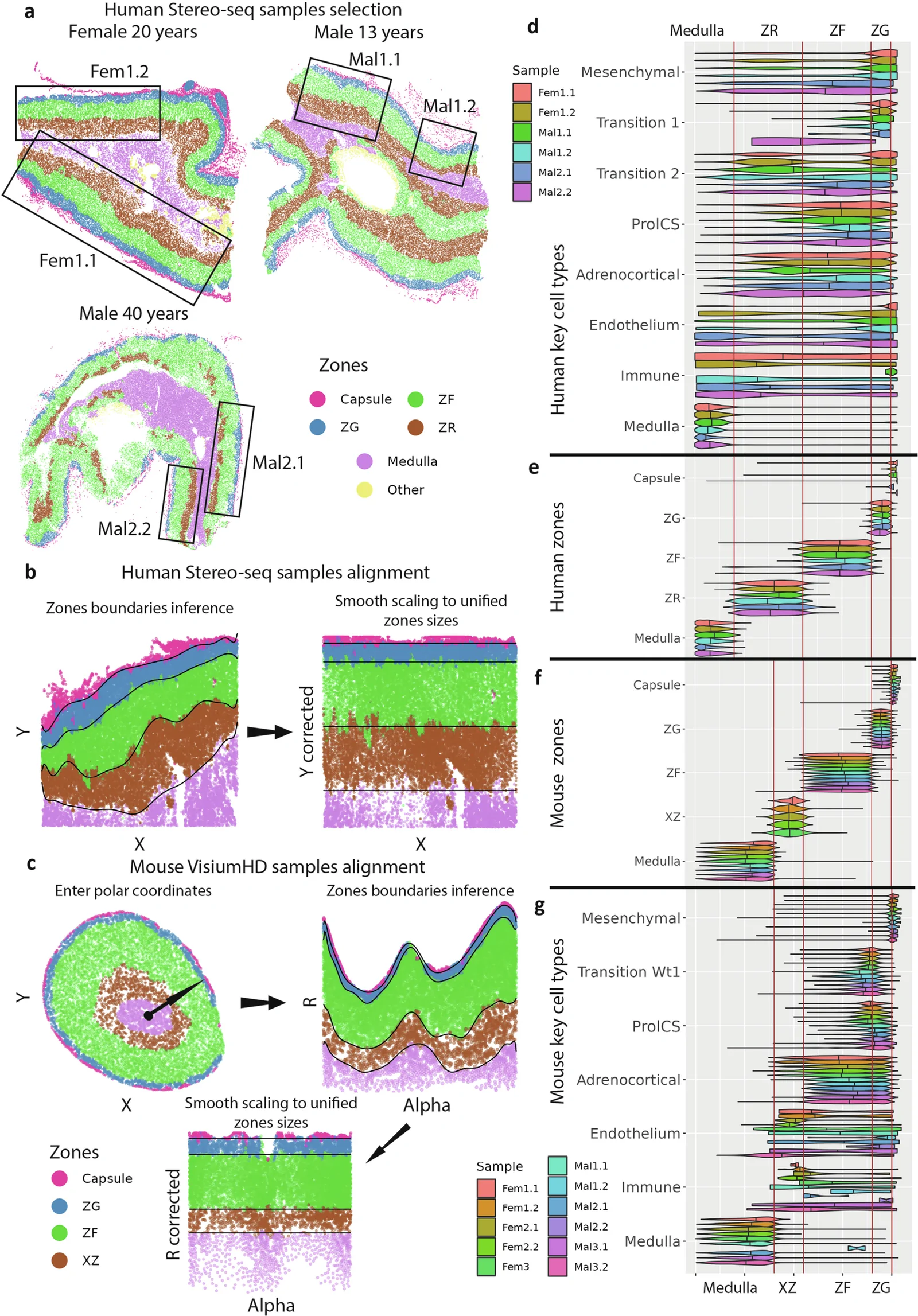
Spatial alignment of human and mouse samples. **a**, Representative areas with intact zonal structure selected for spatial alignment and comparison (highlighted). **b**, Human Stereo-seq spatial alignment procedure (see Methods). **c**, Mouse VisiumHD spatial alignment procedure (see Methods). **d-g**, Spatial distribution of cell clusters across all samples after coordinate transformation equalizing ZG and ZF sizes between species. **d**, Human adrenal cell types. **e**, Human adrenal zones. **f**, Mouse adrenal zones. **g**, Human adrenal cell types. Zone distribution violins confirm successful alignment across all human and mouse samples. ZG: zona glomerulosa; ZF: zona fasciculata; ZR: zona reticularis; XZ: X-zone; ProlCS: proliferating cortical state.

We then combined the scRNA-seq data from mouse and human adrenal cortices (Fig. 6b–d and Supplementary Figs. 50 and 51). We observed co-clustering of (1) mesenchymal and (2) steroidogenic (*NR5A1*^+^*EZH2*^+^) cycling populations, indicating high transcriptional overlap. Aldosterone-producing *Cyp11b2* or *CYP11B2* cells of mouse and human ZG co-clustered and occupied similar subcapsular positions on tissue sections, despite wider *CYP11B2*^+^ domains in humans (Fig. 6c–f and Supplementary Fig. 52). Analysis of the pansteroid enzyme 3β-hydroxysteroid dehydrogenase revealed distribution of human-specific *HSD3B2* (type II isoform) at the ZG and ZF versus inner ZG-specific expression of the murine isoform *Hsd3b6* (type VI isoform) (Fig. 6e,f)^32^. The remaining adrenocortical subclusters occupied species-restricted domains, revealing substantial transcriptional divergence despite general similarity (Fig. 6c,d and Supplementary Fig. 52) and similar spatial distribution of functionally relevant clusters (Fig. 7a,b and Extended Data Fig. 10).

Mesenchymal, ZG and ZF signatures and genes of steroidogenesis revealed conserved *DCN* expression in mesenchyme and *CYP11B1* in ZF whereas cortical *DCN*, *VCAN*, *CCN3*, *CYP17A1* and *SULT2A1* were human specific (Fig. 7c,d). We noted striking disparities in expression of the Notch inhibitor *Dlk1* or *DLK1* in human ZG versus mouse mesenchyme (capsule), suggesting differential regulation of Notch signaling and cell–cell communication (Fig. 7c). Mouse ZG-specific *Ly6d* was a pancortical marker in human adrenals whereas *Ptn*, expressed in a gradient in mouse ZG, is mosaically expressed in humans (Fig. 7e). As a metric of cortical subpopulations, we quantified interspecies heterogeneity of ZG and ZG-to-ZF clusters, revealing increased heterogeneity in humans versus mice (Supplementary Fig. 52g), potentially reflecting transitional states and tissue scaling.

**Fig. 8:**
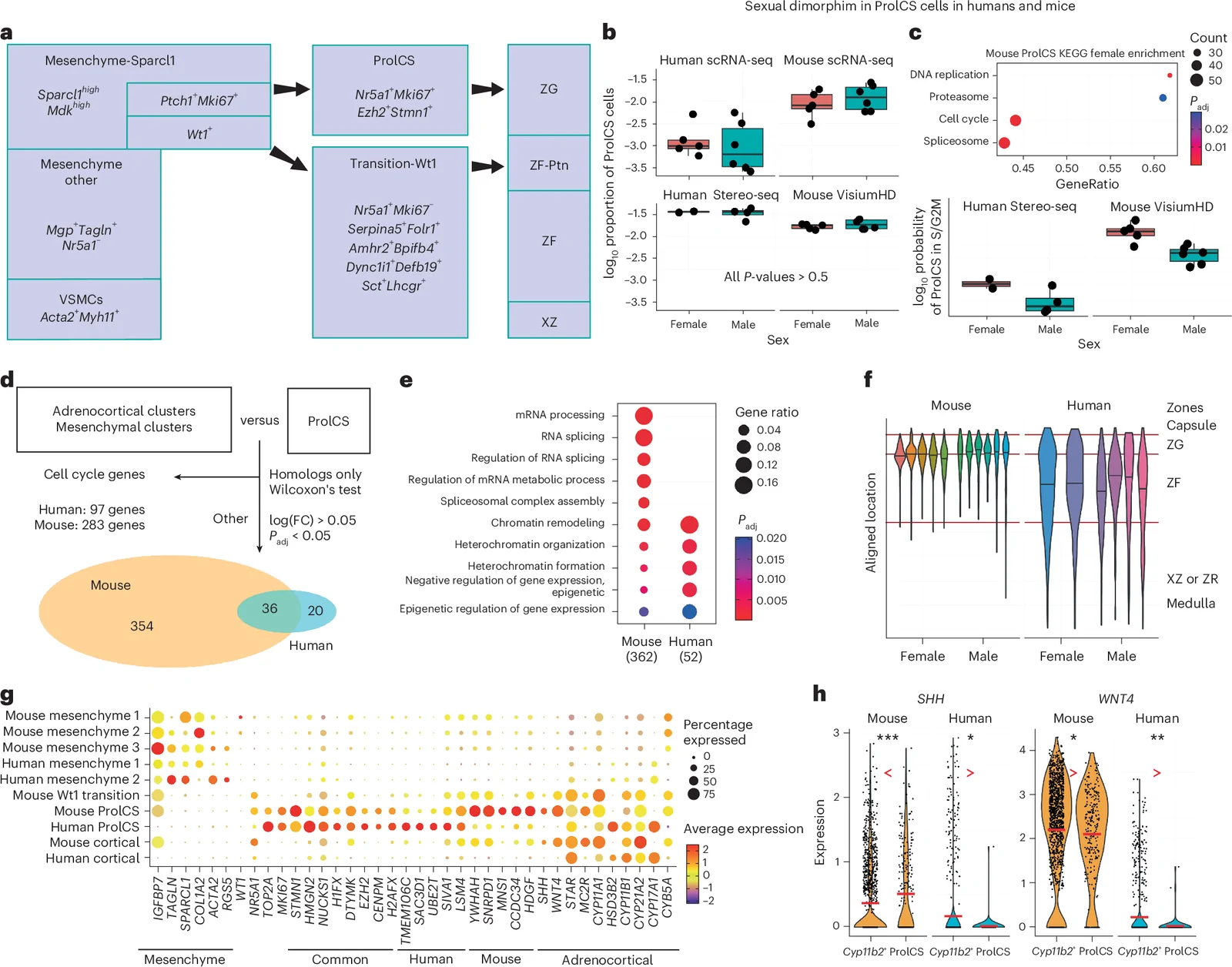
ProlCS cells show partially distinct molecular features between humans and mice. **a**, Scheme of adult mouse adrenocortical renewal depicting transitions from mesenchymal subpopulations and ProlCS cells. **b**, ProlCS cell counts (log_10_(transformed) Laplace-smoothed fractions) by sex—human scRNA-seq: six female and five male; mouse scRNA-seq: five female and six male (10×); human Stereo-seq: one female and two male (two and four slide pieces as independent samples); and mouse Visium HD: five females and six males. No significant sex differences were seen in humans or mice (two-sided Wilcoxon’s test). **c**, Top: DE pathways between male and female mice ProlCS (pseudo-bulk, cacoa; two-sided gene set enrichment analysis (GSEA), BH adjusted). The human cohort was insufficient for an equivalent analysis. Bottom: fraction of ProlCS in S or G2M phase per spatial sample—female mouse ProlCS shows higher cycling fraction (*P*= 0.009, two-sided Wilcoxon’s test). The sample counts are as in **b**. **d**, Venn diagram of ProlCS marker genes, human versus mouse (homolog-filtered, cell-cycle genes excluded; two-sided Wilcoxon’s test, BH adjusted, log(FC) > 0.05, *P* < 0.05). **e**, Gene set overrepresentation analysis (GO BP) of human and mouse ProlCS markers from **d** (one-sided hypergeometric test, BH adjusted). **f**, Spatial distribution of ProlCS across all samples after coordinate transformation. **g**, DotPlot of ProlCS-specific markers in humans and mice with mesenchymal and adrenocortical reference genes. **h**, *SHH* or *Shh* (left) and *WNT4* or *Wnt4* (right) expression in ProlCS versus *CYP11B2*^+^ or *Cyp11b2*^+^ cells. The horizontal lines are means; *P* values: 0.0003, 0.016, 0.020 and 0.009 (two-sided Wilcoxon’s test). For all box plots, the center is the median, the bounds the quartiles and the whiskers 1.5× the IQR. ^*^P < 0.05; ^**^P < 0.01; ^***^P < 0.001; NS, *P* > 0.05. KEGG, Kyoto Encyclopedia of Genes and Genomes.

We then analyzed adrenocortical cell generation in the adult mouse adrenal gland. In agreement with the literature, a subset of capsular *Wt1*^+^ cells co-expresses *Gli1*, although we additionally observed *Gli1*^+^*Ptch1*^+^*Wt1*^−^ cells (Supplementary Figs. 40e–h and 53). Such a composite nature of the capsule was suggested before^18^ and our analysis confirms direct *Gli1*^+^*Ptch1*^+^*Wt1*^−^ cell contribution to dividing *Nr5a1*^+^(SF1^+^)*Shh*^+^*Wnt4*^+^ cortical cells (cluster 4: ProlCS) in the outermost ZG, as shown by lineage-tracing studies in mice^17^ (Supplementary Figs. 53 and 54). Previously described *Wt1*^+^ or *Gata4*^+^ mesenchymal capsular cells giving rise to the ZF^18,33^ bypassing the ZG were linked through a transient state in our data (cluster 5: Transition–Wt1) with numerous unique marker genes (Fig. 7k and Supplementary Fig. 55). RNA velocity analysis supported mesenchyme-to-cortex renewal directions in adult mice (Supplementary Fig. 56), but the transition directions between the ZG subclusters could not be robustly established (Supplementary Fig. 57 and Supplementary Table 8). Overall, we reproduced known mechanisms of cortical renewal in mice^17,18,33^, revealing the transcriptomic signatures of progenitors that contribute directly to the ZG and ZF (Fig. 8a).

Then, we compared the stem cells that we uncovered in human glands to those in mice (Fig. 7f–k). Capsular markers were consistent between humans and mice, including *NESTIN*, a marker of stem cells responsible for stress-response adaptation^34^, expressed by the capsule of both species (Fig. 7l). We observed common Sonic Hedgehog (SHH) signaling mediated through the receptor *Ptch1*, WNT signaling (expression of WNT agonist *Ccdc80*) and conserved *Sparcl* expression between species (Fig. 7h). Similarly, proliferating *NR5A1*^+^ cells shared expression of *STMN1*, *EZH2* and other genes associated with DNA replication (Fig. 7i). A major evolutionary difference was the absence of *WT1*^+^ cells in adult humans (except for negligible numbers), despite their abundance in mice (Fig. 7k). Transitions observed from adult human mesenchymal or perivascular cell populations to adrenocortical cells (*RGS5*^+^ bridge) were not identified in mice (Fig. 7j).

Quantification of proliferating *NR5A1*^+^ cells did not reveal a statistically significant difference between males and females in either species (Fig. 8b). Further exploration of mouse sexual dimorphism proved the enrichment of cell-cycle and DNA replication pathways in female-derived proliferating *NR5A1*^+^ cells (cluster 4: ProlCS) (Fig. 8c and Supplementary Fig. 49g–i). This finding was supported by spatial transcriptomics, whereby analysis of mitotic signature in male cortex versus female cortex as a function of (1) cellular subtype and (2) zone of enrichment confirmed more extensive proliferation within cortical cells in females (Fig. 8c and Supplementary Fig. 58). Thus, the described increased rate of adrenal cortex turnover in female mice^17^ relies not on larger numbers of proliferating cells but on an increased proliferation rate. Similar analysis comparing proliferation activity between human male and female *NR5A1*^+^ cells did not lead to a statistical significance (Fig. 8b,c). To comprehend how cortical proliferation might be controlled in the human adult adrenal, we explored how cholesterol balance influences proliferation of tumor adrenocortical cells (data from The Cancer Genome Atlas (TCGA)), revealing positive correlation of cholesterol levels with adrenocortical tumor growth and lethality (Supplementary Fig. 59), a mechanism potentially at play in healthy adult cortical proliferation.

Analysis of co-integrated proliferating *NR5A1*^+^ cells (Fig. 6c) and spatial examination revealed differences in distribution (in mouse ZG versus uniformly distributed in human cortex) and partially shared genes (such as *EZH2, CENPM, H2AFX* and *STMN1*), accompanied by clear transcriptional differences such as lower *WNT4* and *SHH* levels in humans compared to mice (Fig. 8d–g and Supplementary Fig. 53d), regulating ZG identity and *Ptch1*^+^ capsular progenitor recruitment, respectively^35–37^. Despite widespread *Shh* expression in mice ZG in our data (Supplementary Fig. 53d), the literature states that proliferating cortical cells of mice are nested within *Shh*^+^*Nr5a1*^+^*Cyp11b2*^−^ cells^8^. To address this difference in *WNT4* and *SHH* expression in human versus mouse cortex, we set the putatively homologous and differentiated aldosterone-producing *CYP11B2*^+^ ZG cell state as species-specific background (Fig. 8h). Our analysis resulted in higher *Shh* and lower *Wnt4* expression in mouse ProlCS versus differentiated *Cyp11b2*^+^ ZG, whereas, in human ProlCS, *SHH* and *WNT4* levels were lower than in *CYP11B2*^+^ ZG (Fig. 8h).

## Discussion

Cellular and molecular mechanisms regulating human adrenocortical renewal remain poorly understood, mainly due to the limited sample constraints of previous single-cell transcriptomics and mouse model divergence from human adrenal biology. Our single-cell and high-resolution spatial transcriptomic atlases across human and mouse cohorts provide the resolution needed to address these gaps and are publicly accessible without requiring computational expertise.

Our unbiased analysis of human adult adrenal independently confirms continuous transcriptional transitions across adrenal cortical zones and regional *WNT4* and *SHH* activity, consistent with the centripetal model and known marker genes^8,16,21,35–37^. Expanding on markers of the human cortex, we demonstrated numerous transcriptional states within human ZG beyond canonical CYP11B2^+^ cells. ZG subpopulations characterized by diverse gene programs regulate extracellular matrix composition, cell adhesion, migration, growth, proliferation and differentiation, including the known ZG marker *CCN3*, NOTCH signaling inhibitor *DLK1* or proteoglycan *VCAN*^38–40^, pointing to complex signaling and zonal structure maintenance. Even though previous studies independently uncovered these markers in humans^41–44^, our analysis pinpoints the presence of numerous subpopulations with discrete markers.

When addressing human-specific features, we specifically focused on specialized steroidogenic populations, self-renewal mechanisms and sexual dimorphism, not captured in mouse models. We observed differential expression of cholesterol biosynthesis pathways between men and women, suggesting sex-specific and spatially organized regulation of steroidogenesis in adult human adrenals. We uncovered aging-associated ZG cellular phenotype with high *HSD3B2*, *CYP11B1* and *CYP21A2* expression, likely involved in direct cortisol synthesis. Previously, the enzymatic function of 3β-hydroxysteroid dehydrogenase (encoded by *HSD3B2*) was attributed to all adrenocortical cells in humans^9,45^, whereas our results suggest the presence of a corresponding specialized cell state. These findings demonstrate the unique biological sex-dependent and age-dependent dynamics of the human cortex.

Systematic interspecies comparisons revealed that aldosterone-producing *CYP11B2*^+^ cells of the ZG, capsule and proliferating *NR5A1*^+^(SF1^+^) cells are evolutionarily conserved between mouse and human cortex, whereas most cortical cell types showed transcriptional deviations between the two species. Specifically, *CYP17A1*, *SULT2A1*, *VCAN*, *CCN3* and cortical *DCN* were confirmed as human-specific, and *Ly6d* and *Hsd3b6* as mouse-specific, markers. We also uncovered murine-specific *Hsd3b6* expression in ZG cells as the homolog of *HSD3B2* cells in humans, pointing to the importance of studies on the evolutionary divergence and parallel convergence of genes coding for 3β-hydroxysteroid dehydrogenase. Notably, *DLK1*, encoding a NOTCH inhibitor expressed in human ZG subpopulations, is instead expressed in mouse capsular mesenchyme, suggesting differential interspecies regulation of Notch signaling. *WNT4* and *SHH* expression levels are lower in human ZG and ProlCS cells compared to mice, yet remain the key zonation and progenitor recruitment signals in both species^35–37^, suggesting differential regulation of renewal confounded by vastly different organ size and renewal speed. The absence of ZR in mice due to epigenetic silencing of *Cyp17a1*^5,6^ represents the most functionally notable interspecies divergence and limits the use of rodent models for studying androgen production and adrenarche.

Our comparison between human fetal and adult single-cell data revealed partial similarity but important differences in transitory *DCN*^+^, *CCN3*^+^ and *DLK1*^+^ adrenocortical populations and proliferating *NR5A1*^+^*EZH2*^+^ cells (the latter underrepresented in adult adrenals). We pinpointed plausible additional self-renewal sources unique to adult human adrenal (*ACTA2*^+^*RGS5*^+^ VSMC mesenchymal population and *DCN*^+^ progenitors derived from the capsule). We show similarity between the FZ of the developing cortex and the ZR of the adult based on androgen-producing capacity and fetal DZ with adult ZG based on *CCN3* and *HSD3B2* expression. Adult-specific VSMCs, as well as the *DCN*^+^ state, mediate transitions toward ZG and ZF, whereas the fetal cortex at the analyzed stages of development grows through local cortical proliferation of *NR5A1*^+^ cells as previously suggested^30^.

Integrating our human and mouse data, we proposed a model of adrenocortical self-renewal extending beyond the classic centripetal model. First, two noncortical stem cell sources contributed to cortical renewal in both species. Capsular *Gli1*^+^ or *Gli2*^+^ mesenchymal cells contributed to ZG in both mice and humans, confirmed by RNA velocity and consistent with lineage tracing in mice^17,35^. However, their contribution is minimal in healthy adult human adrenals. The second source differs fundamentally between species: in mice, *Wt1*^+^*Gata4*^+^ capsular cells contribute directly to the ZF bypassing the ZG, validated experimentally through unaffected ZF formation on *Nr5a1* deletion in ZG cells^33^. In adult humans, *WT1*^+^ cells are almost absent and we instead identified *ACTA2*^+^*RGS5*^+^ VSMCs (present in the capsule, outer ZG and ZR–medulla boundary) contributing to ZF and ZR through previously uncharacterized intermediate states. The analysis of mCAs in one donor provides orthogonal lineage support for this VSMC-to-ZG/ZF transition, with aberrant VSMCs, ZG and ZF cells sharing clonal identity. In addition, *NR5A1*^+^*EZH2*^+^ cycling cortical cells, validated by Stereo-seq and immunofluorescence, are distributed throughout the human cortex, in contrast to their restriction to the outer ZG in mice. Their lack of mesenchymal features and wide distribution supports a model of local, zone-specific cortical renewal complementing the centripetal model, with *EZH2* expression linked to both steroidogenic differentiation^46^ and cancer progression risk^47^. In mice, proliferative cortical cells show clear sexual dimorphism: female *Nr5a1*^+^*Shh*^+^ cells exhibit 3× higher proliferative activity than the male^17,19^, a difference that we attribute to proliferation rate rather than proliferating cell numbers although no equivalent sex difference is detected in human cortical proliferation.

### Limitations

The human cortex renewal model proposed is inferred from transcriptomic data, directional gene expression changes and computational trajectory analysis, and has not been validated by lineage tracing in human tissue. Although the mCA case provides support for the VSMC-to-steroidogenic transition, it cannot substitute for functional validation. Therefore, this proposed transition should be interpreted as a working hypothesis, pending confirmation in organoids recapitulating adult tissue.

In addition, transcriptional profiles do not directly reflect cell phenotypes: cell identity is shaped by translation, protein turnover and local signaling. The gradual transcriptional shifts that we observed across cortical zones likely underestimate the sharpness of histological boundaries, because protein regulation establishes discrete functional identities despite smooth mRNA gradients. Importantly, despite consistency of our ZG-producing cortisol hypothesis with our spatial transcriptomics, functional validation of cortisol production from the *HSD3B2*^high^ ZG subpopulation is pending and would require steroid hormone measurements in nonfixed tissue or organoid systems.

Finally, sex-associated and age-associated findings are based on a cohort of 11 human donors. Replication in larger cohorts is needed to establish the generalizability of the cholesterol balance dimorphism and age-related ZG subtype.

## Methods

### Ethics and human samples

All research complies with European regulations on human tissue collection from deceased organ donors approved by the Ethical Committee of the Medical University of Vienna, Austria (ethical agreement no. 1544/2020), in full compliance with the Declaration of Helsinki. One adrenal gland per donor was excised, accompanied by kidney explantation from deceased organ donors. Individual informed consent was not obtained from organ donors or their next of kin, because this is not required under applicable Austrian law for research use of tissue surplus to clinical need, such as adrenal glands, which are removed together with the kidneys before transplantation and discarded as waste. In Austria, removal of organs from deceased individuals is governed by the presumed-consent principle under the Federal Act on Organ Transplantation (Organtransplantationsgesetz). Under this framework, tissue removal is lawful in the absence of a registered objection and hospitals are legally required to consult the national registry before any removal and check that no objection was recorded for any donor included in this study. Supplementary Table 1 provides an overview of the samples included in this study and used for data generation. Fixed frozen adrenal gland biopsies are stored and can be shared following an official request to I. Adameyko and material transfer agreement.

### Ethics and mouse work

All research was conducted in compliance with either European Union regulations and ethical permit approved by the Austrian Ministry of Science, Research and Technology (ethical permit no. BMWFW-66.009/0018-WF/V/3b/2017) or Directive 2010/63/EU and recommendations of the local Bioethical Committee of Lomonosov Moscow State University. Mice were housed in standard, specific pathogen-free facilities at 24 °C, 12-h dark-to-light cycle, normal humidity and free access to food and water. Wild-type mice were obtained from Janvier Labs.

### Human adult adrenal gland histology

Human adrenal glands were cryosectioned and immunostained following standard protocols. RNAscope in situ hybridization was performed using the Multiplex Fluorescent Detection Kit v2 (ACD). Details of antibodies and probe information are provided in Supplementary Information.

### Preparation of adrenal gland single-cell suspension, sequencing and analysis

Single-cell suspensions from human adrenals were prepared by enzymatic dissociation, methanol fixed and loaded on to the 10x Chromium controller for library generation and sequencing. Mouse adrenal cells from four to six pooled glands per sex were dissociated enzymatically, filtered and loaded on to the 10x Chromium controller for library generation and sequencing. Samples were further processed by barcoding, downstream library generation and sequencing (performed by the Biomedical Sequencing Facility, CeMM Research Center for Molecular Medicine of the Austrian Academy of Sciences, Vienna, Austria). For detailed description and downstream sequencing parameters and analysis, see Supplementary Information and Supplementary Tables 1–8 for marker genes enriched in each cluster in corresponding embeddings.

### Statistics and reproducibility

No statistical method was used to predetermine sample size. Human adrenal gland samples were obtained from eleven donors (six male and five female, aged 18–73 years) and sample size reflected donor availability rather than a power calculation. Mouse adrenal glands were collected from six animals for spatial transcriptomics and eleven animals for scRNA-seq (males and females analyzed separately), with sample size determined by the requirement for sufficient cell yield after pooling of four to six glands per sample.

The experiments were not randomized, donors were not assigned to experimental groups and all available samples were included in the atlas, with none excluded. The investigators were not blinded to allocation during experiments and outcome assessment. Given the observational and atlas-based nature of this study, blinding was not applicable to the primary data generation or computational analyses. Statistical analyses were performed as described in Methods. Histological validations by immunofluorescence and RNAscope in situ hybridization were performed on tissue sections from eight donors. Representative images shown in the figures are from single experiments and the findings were confirmed across two to three independent staining rounds with similar results.

### Low-resolution spatial transcriptomics (10x Visium) of human adrenal glands

Fresh-frozen optimal cutting temperature-embedded biopsies of human adrenal glands from four individuals underwent spatial transcriptomics (10x Genomics Visium v1) following an established protocol and pipeline from 10x Genomics. The 10-µm-thick sections were permeabilized for 15 min for mRNA capture. Sequencing data were processed with spaceranger v2.0.0 and refdata-gex-GRCh38-2020-A reference genome.

### High-resolution spatial transcriptomics (Stereo-seq) of human adrenal glands

The 10-µm-thick cryosections were mounted on to a STOmics Stereo-seq chip (1 × 1 cm^2^) and enzymatically permeabilized for 10–15 min, followed by downstream treatment according to a published protocol^14^. Purified and amplified fragmentation products were sequenced using an MGI DNBSEQ-Tx sequencer. Sequencing data were processed with SAW v7.0.0^48^ and the GRCh38 reference genome. The resulting.tissue.gef (bin expression data, bin_size = 40) and .adjusted.cellbin.gef (segmented cells expression data) files were transferred to Seurat.rds using the Stereopy v1.1.0 Python package^49^ (st.io.read_gef, st.io.stereo_to_anndata) and script h5ad2rds.R from Stereopy github.

### High-resolution spatial transcriptomics (10x Visium HD) of mouse adrenal glands

Formalin-fixed paraffin-embedded sections of adult mouse adrenals of mice (age 6 weeks, three female and three male C57Bl/6J mice) were processed according to the validated protocol by 10x Genomics (User Guide CG000685, Rev B). Sequencing data were processed with spaceranger v3.0.1 using refdata-gex-mm10-2020-A and the Visium Mouse Transcriptome Probe Set v2.0. Cell segmentation was performed using bin2cell v0.3.0 Python^50^ (a wrapper for StarDist^51–53^) assigning 2-µm bins to cell IDs. Transcriptome and coordinates of each cell were calculated as the sum and mean of constituent bins, respectively.

### Sexual dimorphism analysis

The package cacoa v0.4.0^54^ was used to analyze sexual dimorphism. The GSEA was extended to include the Kyoto Encyclopedia of Genes and Genomes and WikiPathways using the DOSE v3.24.2^55^ and clusterProfiler v4.6.2^56,57^ packages, whereas mitochondrial, ribosomal and the X or Y chromosome genes were excluded from the analysis (biomaRt). The parameter n.boot=5000 was set for analyzing cluster-based compositional differences. Cluster-free analysis was performed using the embedding cell-density approach (bins = 300, bandwidth = 0.08) with Wilcoxon’s adjustment. For adult mouse sexual dimorphism analysis only, 10× samples without a male–female mix were included (six males and five females). Human fetal 10× cells and 10× nuclei samples were analyzed separately. The analysis included only a subset of samples, ensuring that the number of samples was equal for each sex at each week of embryonic development (three and four samples of each sex for cells and nuclei, respectively).

### Transition and velocity analysis

To manually analyze transitions, part of the embedding associated with the transition (source population, target population and bridge) was subselected. The trajectory was constructed using the learn_graph function from the monocle3 v1.3.3 package^58–60^, with parameters ncenter (130, 140 or 150) and minimal_branch_len (15 or 20) chosen to cover the entire target region without branching; close_loop was set to FALSE. Pseudotime was calculated from one of the terminal nodes to correspond to the assumed direction of the transition.

To identify marker genes specific to the transition, rather than the source (from) or target (to) clusters, the strategy described in ref. ^22^ was used. Briefly, for each gene, the normalized-by-gene variance mean value of the residual from a linear model lm(transition ~ MEAN_from + MEAN_to −1), modeling the expression profile of each cell from transition as a mixture of the mean profiles of the ‘from’ and ‘to’ clusters, was estimated. The obtained values were used to prioritize genes, expressed in at least ten cells of transition.

Heatmaps reflecting changes in gene expression during the transition include all transition cells and subsets of cells (sizes equal to the number of transition cells, but no fewer than 300) from the from and to clusters, sorted by pseudotime. The list of genes includes those that characterize the transition (as described above) and marker genes from area under the curve (AUC)-based DE analysis. DE groups were: from versus (transition + to), from versus to, transition versus to, transition versus (from + to), to versus from and to versus (from + transition). Signatures of from and to clusters were calculated as the average scaled expression of cluster markers, which were then winsorized at 5% and 95% thresholds, scaled to (0, 1) and averaged for each cell using a sliding window of ten neighboring cells (five before and five after) after ordering the cells by pseudotime. The coexpression of two signatures was calculated using the following formula:

$$\begin{array}{l}{\rm{Coexpression}}={\rm{Sign\;from}}/({\rm{Sign\;from}}+{\rm{Sign\;to}})\\\qquad\qquad\qquad\times {\rm{Sign\;to}}/({\rm{Sign\;from}}+{\rm{Sign\;to}})\times 4,\end{array}$$

where Sign from and Sign to are signatures of clusters from and to, respectively. Coexpression achieves maximum value = 1, when Sign from = Sign to, which corresponds to cells with intermediate phenotype.

For RNA velocity analysis, the velocyto.py v0.17.17^61^ command line interface (generation of spliced and unspliced count matrices) and Python package scVelo v0.2.4^62^ (transcriptional dynamics analysis in stochastic mode) were used. To identify putative transitions between focused clusters of mouse ZG we used the PAGA approach from the scVelo package.

### Compositional dynamics

The analysis of changes in cluster representation during embryonic development from published datasets, taking into account batch effects from different data sources, was conducted using generalized linear mixed models (glmer function from package lme4 v1.1-34, family = ‘poisson’). Count data were Laplace smoothed. Regression was performed independently for each cluster using the following formula:

$$\begin{array}{l}{\rm{Cluster\;size}} \sim 1+{\rm{Gestation\;week}}+(1{\rm{|Data\;source}})\\\qquad\qquad\qquad\quad+{\rm{Offset}}(\log ({\rm{Sample\;size}})),\end{array}$$

where Cluster size is the number of cells in cluster + 1, Gestation week is the week of development (categorical variable), Data source is the source of data (one each from Kameneva et al.^22^, Jansky et al.^23^, Kildisiute et al.^24^ and Han et al.^25^) and Sample size is the number of cells in sample + number of clusters.

### Cholesterol balance analysis in bulk scRNA-seq and Visium data

First, expression of the cholesterol-consumption signature (steroidogenic enzymes *STAR*, *CYP11A1*, *HSD3B2*, *CYP21A2*, *CYP11B2*, *CYP17A1*, *CYP11B1*, *CYB5A*, *SULT2A1*, *GSTA1* and *AKR1C3*, cholesterol esterification gene *SOAT1* and cholesterol-obtaining negative regulator *MYLIP*) and cholesterol-obtaining signature (WikiPathways pathway WP4718—cholesterol metabolism with Bloch and Kandutsch–Russell pathways, all genes involved in cholesterol synthesis from acetyl-CoA and its uptake from blood and lipid esters, *LDLR*, *SCARB1* and *LIPE*) were calculated using the AddModuleScore function from Seurat. Comparisons of signature activity in adrenocortical zones of male and female adrenals were performed using linear mixture regression (lmer function from package lme4 v.1.1-34) with the following formula:

$${\rm{Signature\;expression}} \sim {\rm{Zone\;sex}}+(1{\rm{|Sample}})-1,$$

where Signature expression is the cholesterol-consumption signature or cholesterol-obtaining signature expression, Zone sex is a composite feature of sex and zone (one each of male ZG, male ZF, male ZR, female ZG, female ZF and female ZR) and Sample is scRNA-seq (or Visium) sample. To test statistical significance of differences in Zone sex coefficients, the samples and cells (or spots) in each sample were resampled with replacement 1,000× and regression for each resampling iteration was independently performed. Empirical *P* values were set to min(st, 1-st), where st is the fraction of iteration with Female zone > Male zone (zone is one of ZG, ZF or ZR), then *P* values were adjusted using the Benjamini–Hochberg procedure.

To test whether increased activity of steroidogenesis explains the increased cholesterol production we used a similar procedure with the formula:

$$\begin{array}{l}{\rm{Cholesterol\;obtaining}} \sim {\rm{Cholesterol\;consumption}}\\\qquad\qquad\qquad\quad\qquad+{\rm{Zone\;sex}}+(1{\rm{|Sample}})-1.\end{array}$$

The cholesterol-balance signature expression was defined as a cholesterol-obtaining signature expression after regressing out cholesterol-consumption signature expression and sample-specific random effects.

Activities of gene signatures in adrenocortical carcinoma TCGA-ACC bulk RNA-seq data (79 samples with survival data) were estimated using the ssgsea function from package corto v1.2.2^63^. The proliferating cortical state gene signature was obtained as markers of cluster ProlCS with AUC > 0.6 in the fetal dataset (which contains numerous proliferating cells), cell-cycle-associated genes I(CC) (see Supplementary Notes) were excluded here and used as a separate signature. Cholesterol balance was treated as residuals of model lm(Cholesterol obtaining ~ 1 + Cholesterol consumption). Statistical significance of difference between cholesterol-obtaining and cholesterol-balance Pearson’s correlations with ProlCS signature was estimated via bootstrap (boot v1.3-28). For each signature, the fraction of the explained variance in the model lm(ProlCS ~ 1 + Signature) was also estimated. Confidence intervals and significance of difference between cholesterol-obtaining-explained and cholesterol-balance-explained variances were estimated via bootstrap. Survival analysis was performed using R package survival v3.4-0.

Normal adrenal gland bulk RNA-seq data (106 females and 168 males) from the Genotype-Tissue Expression cohort^15^ were obtained via the recount3 v1.0.7 R package^64^. Raw counts were transcript per million normalized via the recount v1.28.0 package^65^. Gene IDs were mapped on the HUGO Gene Nomenclature Committee gene names (biomaRt v2.58.8^66,67^), then the cholesterol-obtaining signature and cholesterol-usage signature were estimated using the ssgsea function from package corto v1.2.4^63^. The cholesterol-balance signature expression was defined as the cholesterol-obtaining signature expression after regressing out the cholesterol-consumption signature expression in model lm(Cholsource ~ Cholconsum).

### Somatic CNV analysis

To infer clonal relationships among adrenal gland cells, we analyzed somatic copy number variations (CNVs) using the Numbat (2.6) package^68^, following the developer-provided vignette. The scRNA-seq count matrices were input without prior CNV preselection. The method leverages allele-based and expression-based inference to estimate CNVs and assign clonal identities at single-cell resolution. Clones were visualized across UMAP embeddings and anatomical compartments to explore potential lineage structure.

### Analysis of low-resolution Visium spatial transcriptomics data

The R package Seurat was used to work with spatial transcriptomics data. To obtain joint clustering and zone annotation, four Visium samples were Seurat integrated with parameters (nfeatures = 3,000, npcs = 30, resolution = 0.5) and after excluding I(MT, RB, HB, CC). For cell-type deconvolution R package spacexr v2.2.1^69^ (RCTD) was used with doublet_mode = ‘full’, as recommended for low-resolution Visium data. Expected cell-type-specific gene expression for each spot was estimated as proportional to the proportion of the cell type on the spot and the probability of observing the gene in each cell type (according to the scRNA-seq reference), as suggested in the original RCTD publication. For spatial visualization of gene expression in adrenocortical and mesenchymal cells, to exclude the impact of other cell types (especially relevant for cell-cycle markers), SCTransform-normalized, cell-type-specific gene expression was used. First, cell-type deconvolution was performed based on low resolution (usually more robust than high resolution) cell-type annotation, which included adrenocortical, chromaffin, endothelial, immune, mesenchymal and SCP cells. Next, cell-type-specific gene expressions were estimated. Finally, matrices of adrenocortical and mesenchymal cells were summed and spots with <1,500 counts were excluded (this threshold allowed avoidance of visualization of adrenocortical ambient RNA in the medulla) and the resulting count matrix was SCTransform normalized and visualized. For visualization of marker genes of adrenocortical zones or clusters, adrenocortical-specific matrix (without mesenchymal matrix summation) was used.

### Analysis of high-resolution (Stereo-seq and Visium HD) spatial transcriptomics data

The R package Seurat v5.1.0 was used to work with spatial transcriptomics data. The initial analysis included two orthogonal procedures. First, identification of key zones in adrenal glands (capsule, ZG, ZF, ZR or XZ and medulla) was performed via an unsupervised clustering approach on binned spatial data. This meant that no tissue-imaging information and no scRNA-seq reference, only expression data from bins (not segmented cells) and their coordinates, were used to make homogeneous layers. This analysis, which referred to tissue-pattern identification, allowed us afterwards to get unbiased statistics of how each cell type is distributed by cortical zone and perform spatial alignment.

Second, when it comes to cell type, subtype or state localization and validation, there was a need to be sure that we worked with real cells, not some complicated mixture of them. So, this analysis was based on segmented cells (their positions and transcriptomes) and scRNA-seq reference-based annotation and deconvolution.

All three human samples were analyzed individually in both tissue-pattern identification and cell-type annotation. Each slide containing mouse adrenals (Visium HD) was manually split into individual adrenal sections for the analysis and visualization purposes and to remove empty areas between sections. Empty areas were identified using a tissue-pattern-finding procedure.

For the visualization purposes and cell-type co-occurrence analysis, the Giotto v1.1.2R package^70^ was used. To compute cell–cell interaction enrichment: first, a spatial *k*-nearest neigbors (KNN) graph was calculated using function createSpatialNetwork (*k* = 4, method = ‘kNN’), then proximity enrichment was estimated via cellProximityEnrichment function (adjust_method = ‘fdr’, number_of_simulations = 1,000)

### Tissue-pattern identification in high-resolution spatial transcriptomics data

For the tissue-pattern identification, the Banksy v0.99.13 package^71^ and SeuratWrappers v0.4.0 were used according to the Seurat vignette.

For human samples, low-quality bins (bin_size = 40) with nFeatures <80, nCounts <80, percent.mt >20, percent.rb >20 or percent.hb >5 were filtered out. Then, each sample was normalized via SCTransform (Seurat v5.0.1) and the top 2,000 variable features after exclusion of I(MT, HB, RB) were selected. On these features a BANKSY assay was generated via the RunBanksy function (λ = 0.2, assay = ‘SCT’, slot = ‘data’, k_geom = 20) and dimensional reduction on this assay was generated using function RunPCA (npcs = 20), FindNeighbors, FindClusters (resolution = 0.2 or 0.3 for the young male sample) and RunUMAP. For the aged male sample (due to reported aging-associated disruption of adrenocortical zonation^13,72^), the medulla was removed and all analyses repeated (npcs = 10, resolution = 0.2) to obtain more accurate zone patterns. The resulting bin-level tissue patterns were transferred on the cells by the nearest neighbors on spatial coordinates using class::knn v7.3-20 (*k* = 4, *l* = 0, probability = FALSE, use.all = TRUE).

For mouse samples, BANKSY assays were generated on 8-μm bins independently across samples and variable features were selected using the Seurat function SelectIntegrationFeatures, but kept common within male and female groups. Then, samples were merged, scaled (ScaleData) and integrated in each sex group using Harmony v1.2.1. Finally, embedding (on 15 harmony-corrected principal component analysis components) and clustering (resolution = 0.2) were generated. The resulting clusters (tissue patterns) were transferred on high-resolution (2-μm) bins using the nearest neighbors on spatial coordinates with class::knn v7.3-20 (*k* = 4, *l* = 0, probability = FALSE, use.all = TRUE). Tissue-pattern identities of cells were set to the most common identity of the corresponding 2-μm bins.

### Cell-type annotation in high-resolution spatial transcriptomics data

Segmented cells with >100 RNA counts (unique molecular identifiers) were annotated using spacexr v2.2.1^69^ (RCTD) doublet_mode = ‘doublet’, as recommended for single-cell resolution spatial transcriptomics. Annotation was performed using an scRNA-seq reference in an iterative manner with a gradual increase in annotation resolution. Briefly, we initially annotated mesenchymal and adrenocortical, immune, endothelial and medullary cells. Then, adrenocortical cells were re-annotated to separate cells of different zones and intermediate states (that is, putatively transitional cell states or ProlCSs). Finally, ZG internal heterogeneity was inferred and, independently, mesenchymal cells were re-annotated to finer subpopulations. All three human samples were annotated independently (because each sample was processed on an independent Stereo-seq slide), whereas mouse samples on the same slide were annotated jointly, because this provided more data for RCTD’s internal platform-effect parameter inference and was expected to result in better annotation of rare cell types (a few cells per section).

### Cycling cell identification in high-resolution spatial transcriptomics data

Cells in G2M or S-cell cycle phases (that is, cycling cells) were identified using AUCell v1.24.0^73^ functions AUCell_buildRankings, AUCell_calcAUC (aucMaxRank = 2600 or aucMaxRank = 850 for Stereo-seq or Visium HD data, respectively) and AUCell_exploreThresholds (thrP = 0.01, assignCells = TRUE). I(MT, RB, HB) genes were excluded from the count matrix before calculation of rankings. An extended cell-cycle signature was calculated on the whole adrenal scRNA-seq dataset as an intersection of I(CC) and II(CC = 0.05) gene sets, which resulted in 214 and 202 genes for humans and mice, respectively. In other words, those signatures are GO-annotated cell-cycle genes, which are positively correlated with Seurat-estimated G2M or S-cell cycle scores (to exclude mitotic negative regulators).

To estimate mitotic activity of cortical cells in each spatial sample, we utilized a generalized linear model (glm function from package stats v4.0.5) with family = ‘binomial’ and model cycling ~ annotation, where cycling binomial variable is AUCell-identified cells in the G2M or S phase. An annotation categorical variable is a cell-type annotation as ProlCS or other adrenocortical cell type (generally not cycling, served as a background here). The resulting per-sample coefficients were used for male versus female comparison via a two-sided Wilcoxon’s test.

### Spatial alignment of high-resolution spatial transcriptomics samples

This analysis was used to achieve a visual representation of zonal structures in the spatial data with consistency among different samples while adjusting local tissue morphology deviations and capturing the distribution of cell types. Radial coordinates were introduced for mouse adrenals (concentric around the middle of the medulla, if present, or ZF) to obtain a layered structure, whereas small outlier pieces were manually removed. For human adrenal sections with more complex morphology, two pieces per sample with representative layered structure and relatively intact capsule were manually selected.

Boundaries between each pair of connected zones (medulla and ZR, ZR and ZF, ZF and ZG, ZG and capsule) were independently inferred using logistic regression (glm, family = ‘binomial’) as a polynomial function of the horizontal axis with models Zones ~ poly(α, 20) + R or Zones ~ poly(X, 14) + Y in mice or humans, respectively. This provides the ability for each α or X to obtain specific coordinates of all boundaries. After that, for each α or X a function that transforms original coordinates to aligned coordinates can be constructed as a smooth monotonic spline (R function splinefun from package stats v4.0.5, method = ‘monoH.FC’) with *x* values as original zonal boundaries (calculated for each α or X) and *y* values as targets for zonal boundaries (constant for all α or X). Constant (target) vertical coordinates for boundaries were set to 1 (between the ZG and capsule), 0.9 (between the ZG and ZF), 0.55 (between the ZF and ZR or XZ), 0.4 (between the XZ and medulla) and 0.2 (between the ZR and medulla) to be roughly comparable with average zone sizes. For each cell on spatial slide, we obtained corresponding zone boundaries, constructed a spline function and calculated new (aligned) vertical coordinates.

### Reporting summary

Further information on research design is available in the Nature Portfolio Reporting Summary linked to this article.

## Online content

Any methods, additional references, Nature Portfolio reporting summaries, source data, extended data, supplementary information, acknowledgements, peer review information; details of author contributions and competing interests; and statements of data and code availability are available at https://doi.org/10.1038/s41588-026-02737-1.

## Supplementary information

Supplementary Information Supplementary Methods, Description and links to data available on cell × gene, Table 1, Information on primary and secondary antibodies used for immunofluorescence, Information on RNAscope probes, Figs. 1–59 and References.

Reporting Summary

Peer Review File

Supplementary Tables 1–8 Genes enriched in analysis.

## Data Availability

Sequenced reads of mouse scRNA-seq and Visium HD samples, h5 files with gene counts in individual cells or spots and all data related to Visium, Visium HD or Stereo-Seq spatial transcriptomics (coordinate matrices, slide images and so on) were deposited under the Gene Expression Omnibus (GEO) accession no. GSE253852. Sequenced reads of human scRNA-seq, Visium and Stereo-seq spatial transcriptomics were deposited under European Genome–phenome Archive (EGA) controllable accession no. EGAC00001003383. The EGA provides a managed access framework in which human data are archived under controlled conditions and released only to approved applicants through a Data Access Committee (DAC) review process. Researchers wishing to access the raw data may submit a data access request to the DAC via the EGA portal (https://ega-archive.org/datasets/EGAC00001003383), describing their intended use. Requests are evaluated only against the original ethical framework and, if approved according to the ethical criteria, access is granted subject to a Data Use Agreement that legally binds the recipient to the approved terms of use. Controlled access via the EGA is not intended to restrict scientific use, but to ensure that human raw sequencing data cannot be repurposed in ways that would violate donor privacy or applicable ethical regulations. The contact details for the DAC and expected response timelines are available on the EGA dataset page. We provide interactive access to the data via CellxGene^74–76^ (detailed list and links in Supplementary Information) and PDF versions of all Extended Data figures and Supplementary figures are available via figshare at https://adameykolab.hifo.meduniwien.ac.at/cellxgene_public/filecrawl/2026_NatGen_Kastriti_Maksimov (ref. ^77^). Human fetal scRNA-seq data were obtained from the following sources: Kameneva et al.^22^ processed Seurat object via the link from github: https://github.com/artem-artemov/adrenal (http://pklab.med.harvard.edu/artem/adrenal/data/Seurat/adrenal.human.seurat.scrublet.rds); Jansky et al.^23^ counted matrix and annotation from: https://adrenal.kitz-heidelberg.de/developmental_programs_NB_viz/; Kildisiute et al.^24^ processed Seurat object from: https://www.neuroblastomacellatlas.org/, Han et al.^25^ samples counted matrices from GEO accession no. GSE134355. Previously published mouse adult scRNA-seq sample count matrices were downloaded from GEO accession nos. GSE134355 and GSE161751 (refs. ^25,31^). Normal adrenal gland bulk RNA-seq data from the Genotype-Tissue Expression cohort^15^ were obtained via the recount3 v1.0.7R package^65^. Adrenocortical carcinoma bulk RNA-seq and metadata were downloaded from the TCGA portal: https://portal.gdc.cancer.gov/projects/TCGA-ACC.

## Code availability

Source code is available via GitLab at https://gitlab.com/MaksimovDenis/adrenalglandscatlas and via Zenodo at https://doi.org/10.5281/zenodo.20258071(ref. ^78^).
