# Supplementary material for: Human and mouse adrenal glands are characterized by species-specific steroidogenic states and tissue turnover
Source: Nat Genet. 2026 Sep 8;58(9):2270–83. doi: 10.1038/s41588-026-02737-1 (PMC13553300; doi:10.1038/s41588-026-02737-1)
Supplement: Supplementary file 1 [file 41588_2026_2737_MOESM1_ESM.pdf]

nature genetics

Supplementary information

https://doi.org/10.1038/s41588-026-02737-1

Human and mouse adrenal glands are characterized by species-specific steroidogenic states and tissue turnover

In the format provided by the authors and unedited

Supplementary Information File

Contents:

Supplementary Methods:

Detailed human adult adrenal gland preparation and histology

Preparation of human adrenal gland single-cell suspension

Preparation of mouse adrenal gland single-cell suspension

Single cell RNA-sequencing, library preparation and preprocessing

Analysis of single-cell RNA-sequencing data and quality control (QC)

Removing unwanted effects during single cell RNA-sequencing data integration

Marker gene identification and representation

Tissue-level integration and cell type annotation

Target integrations of adrenocortical and mesenchymal cells

Target integrations of medullary cells

Target integrations of mesenchymal cells

Target integrations of endothelial cells

Target integrations of immune cells

Description and links to data available on cell x gene

Table 1 - adrenal donor information

Supplementary information on primary and secondary antibodies used for immunofluorescence

Supplementary information on RNAscope probes

Supplementary Figures 1-59

Supplementary References

Supplementary Methods

Detailed human adult adrenal gland preparation and histology

Excised human adrenal glands were cut into 1-cm-wide strips and fixed in 4% PFA for 12-18 hrs at 4°C. Following 3 x washes with 1xDPBS, biopsies were transferred to 30% sucrose in 1xDPBS for cryoprotection for 36 hrs minimum before cryoembedding in O.C.T. Cryosectioning took place at -20°C using a cryostat (Leica CM3050 S) and sections mounted on SuperFrost Plus slides before storing at -20°C until further use.

For immunofluorescent stainings, sections were subjected to heat-mediated antigen retrieval using a steam cooker in prewarmed 1xAntigen Retrieval Solution (S1699, Dako) for 10 min. Following 3 washes in 1xPBST (0.1% Tween 20 in 1xPBS), antibodies (please see Supplementary Notes for information on antibodies) were applied in blocking solution (5% normal donkey serum, 0.1% Tween 20 in 1xDPBS) overnight at 4°C. After three washes in 1xPBST, Alexa fluorophore-conjugated secondary antibodies combined with DAPI were applied to sections for 45 min at room temperature, followed by 3 washes in 1xPBST. Slides were mounted using Mowiöl until imaging took place.

RNAscope in situ hybridization on fixed cryosections was performed according to the manufacturer's protocol using the RNAscope Multiplex Fluorescent Detection Kit v2 (Advanced Cell Diagnostics, Cat No 323110). Please see Supplementary Notes for probe information.

Stained tissue was imaged using a LSM780 Leica confocal microscope equipped with 10x and 20x objectives. Images were exported as high resolution jpegs using Fiji (ImageJ 1.53t) and assembled using Adobe Photoshop (26.4.1) and Adobe Illustrator (29.3.1).

Preparation of human adrenal gland single-cell suspension

Human adrenal glands, kept in sterile 1xPBS on ice were cleaned from surrounding fat, cut into strips and then into 5 mm x 5 mm pieces. Enzymatic digestion took place in dispase II (2 mg/ml) (Roche, Cat No 04942078001)/collagenase P (2 mg/ml) (Roche Cat No 11213865001) mix in 1xPBS solution containing 2 mM CaCl2 at 37°C, with mild shaking for 30-40 min, followed by mechanical dissociation with a wide-bore pipette tip. Single cell suspension was transferred to a clean falcon through a 100-µm-pore size cell culture filter. Cells were precipitated by centrifugation at 300 x g, 5 min, 4°C and washed three times with 10% FBS (Fetal Bovine Serum) 1xPBS followed by 90% methanol fixation according to the demonstrated protocol by 10x Genomics and stored at -80°C. Rehydration of methanol-fixed single cells was performed just prior to cell loadind onto the 10x Genomics Chromium controller. Briefly, fixed cell suspension was equilibrated on ice and spun down at 3000 g, 10 min, 4°C. Supernatant was removed and replaced by 1 mL rehydration buffer. Spinning down, removal of supernatant and resuspension in rehydration buffer (1% Bovine Serum Albumin, or BSA, 0.5 U/µL RNAse out (ThermoFisher Scientific, Cat No 10777019) in 1xPBS) was repeated two more times, cells were counted and resuspended to a concentration of 700-1,500 cells/µL. Subsequently, cells were loaded onto the 10x Chromium controller and samples were further processed by

barcoding, downstream library generation and sequencing (performed by the Biomedical Sequencing Facility, CeMM Research Center for Molecular Medicine of the Austrian Academy of Sciences, Vienna, Austria).

Preparation of mouse adrenal gland single-cell suspension

Mouse adrenal glands were collected from 5-week-old C57BL-CD-1/10SnSlc-H-2aw18/H-2b mice and 6-to-8-week-old C57BL/6J mice. Cells from 4–6 adrenals (collected from 2 or 3 animals) were pooled into one sample, separated according to biological sex. Glands were cut in small pieces and kept in 1xPBS/10% FBS solution on ice. Then organs were cleared from fat and further shredded using a scalpel. Enzymatic digestion took place in a dissociation solution (HBSS Ca^{2+}, 1.26 mM; collagenase type I, 20 mg/mL; DNaseI; BSA 0.3%) for 30 minutes at 37°C by shaking at 100 rpm and gentle pipetting every 10 minutes. After dissociation, 1xPBS containing 10% FBS was added to the cell suspension and centrifuged at 500 x g for 5 minutes at 4°C. The cell pellet was resuspended in 10% FBS in 1xPBS and filtered using a 70 μM filter, centrifuged at 500 x g for 5 minutes at 4°C. Cells were resuspended in 1xPBS/0.03% BSA to a concentration of 1000 cells/μL prior to loading onto the 10x Chromium controller, barcoding and downstream library generation and sequencing.

Single cell RNA-sequencing, library preparation and preprocessing

Single-cell RNA-seq libraries were generated with the Next GEM Single Cell 3' Reagent Kit (v3.1, 10x Genomics) according to the manufacturer's instructions aiming for a maximum cell recovery of 10,000 cells. Library concentrations were quantified with the Qubit 2.0 Fluorometric Quantitation system (Life Technologies, Carlsbad, CA, USA) and size distribution was assessed using the Experion Automated Electrophoresis System (Bio-Rad, Hercules, CA, USA). Both human and mouse scRNA-seq libraries were sequenced using either Illumina HiSeq 4000 or NovaSeq 6000 platform. Raw sequencing data were demultiplexed using bcl2fastq v2.20.0.422. The data were further processed with the CellRanger v7.0.0 count pipeline[1] and refdata-gex-GRCh38-2020-A or refdata-gex-mm10-2020-A reference genomes for human and mouse respectively, downloaded from 10x Genomics web site (https://www.10xgenomics.com/).

Analysis of single-cell RNA-sequencing data and quality control (QC)

The R package Seurat v.4.3.0 was used to calculate the quality control metrics and perform filtering with adjusted parameters as described below[2,3].

Human adult adrenal data

Cells with < 250 genes or 500 counts (UMIs) were manually removed. Function gene.vs.molecule.cell.filter (min.cell.size = 500, max.cell.size = 50,000) from pagoda2 v.1.0.10 package[4] was used to filter out cells with underrepresented gene diversity for a given number of counts. Cells with high doublet score estimates (python package Scrublet v.0.2.3)[5], percent of mitochondrial genes >25% and hemoglobin counts >20% were filtered out. Following testing of various doublet filtering thresholds (scrublet score 0.2, 0.25, 0.3, 0.35, 0.4) were tested in order to find one that, on the one hand, preserves as many cells as possible in the analysis, and on the other hand, does not allow clusters with markers of endothelial or immune cells to appear in the focused

integration of adrenocortical and mesenchymal cells. Briefly, for each scrublet score threshold we performed ambient RNA correction (R package SoupX v.1.6.2, details below)[6], single cell RNA sequenced sample integration (R package Harmony v.0.1.1, details below)[7] and marker based cell type annotation (details below). For each scrublet score threshold adrenocortical and mesenchymal cells were extracted and re-integrated (R package Seurat, details below). When the same procedure (integration, cell type annotation, adrenocortical & mesenchymal cells re-integration) was performed without ambient RNA correction it always resulted in the appearance of clusters with immune or endothelial markers (even with a hard scrublet score threshold 0.2). After visualization of immune and endothelial gene expression and distribution of cells from ambient-rich clusters on ambient-corrected embeddings, the scrublet score threshold of 0.35 was selected.

Ambient RNA correction was performed using SoupX package functions estimateSoup (soupRange = c(2, 10)), autoEstCont and adjustCounts (method = "subtraction", roundToInt = T) for each sample individually, based on Seurat clusters. Clustering was obtained via standard Seurat pipeline (SCTransform, RunPCA (n.pcs = 30), FindNeighbours (dims = 1:30), FindClusters (n.iter = 500)). Cells with <500 counts after ambient RNA correction were removed.

Mouse adult adrenal data

For mouse data cell gene content was the same as for adult human samples, with the threshold for mitochondrial and hemoglobin gene fraction at >25%. For general (all cell types) integration and focused endothelial, immune integrations cells with scrublet scores <0.2 were used. For medullary and adrenocortical & mesenchymal integrations cells with scrublet scores >0.4 were filtered out and other doublet/high ambient cells were removed manually (see below).

Human fetal adrenal data

For datasets from Jansky et al.[8] and Kildisiute et al.[9] no additional QC filtering was performed and original annotation was used. For Kameneva et al.[10] already annotated dataset cells with >5% fraction of hemoglobin reads were additionally filtered out. Han et al.[11] QC was performed manuallya as described for adult human samples, with >5% threshold for hemoglobin fraction and 0.2 threshold for scrublet score.

Removing unwanted effects during single cell RNA-sequencing data integration

Two strategies were employed to remove unwanted effects (cell cycle, variability in quality after dissociation) presented by a set of genes X during integration. Strategy I involved simply excluding the set X genes from the variable features (VFs) for integration. Strategy II involved excluding genes that correlated with the X signature in addition to genes from X. The X signature was calculated using the Seurat function AddModuleScore (features = X) or CellCycleScoring (only for cell cycle with default parameters) on the merged and normalized (NormalizeData function) Seurat object - several scRNA-seq samples before batch correction. The X signature for mitochondrial or ribosomal RNA was the percent of corresponding counts in each cell (PercentageFeatureSet function). Next, scaling was performed using the ScaleData

(features = rownames) function and correlation was computed using the R function rowCors between the calculated signature and each of the genes in the scale.data slot of the Seurat object. When removing the cell cycle effect, the correlation was calculated for each gene with the S.Score and G2M.Score signatures, and the maximum value was taken. Then, the distribution of correlations was plotted and the threshold was manually selected so that it covered outliers, most of the genes in the X, and did not include biologically informative genes related to other processes. Finally, genes with an absolute (except mitochondrial RNA and adrenocortical ambient RNA signatures) correlation value above the threshold were combined with genes from X for subsequent exclusion from the list of VFs.

Gene sets X used are mitochondrial genes (MT), ribosomal genes (RB), hemoglobin genes (HB), stress genes *FOS, FOSB, JUN, JUNB* (ST), adrenocortical genes *STAR, CYP11B1, CYP11A1, RARRES2* (CORT) or cell cycle (CC). The CC set includes genes annotated in cell cycle-relevant GO categories (GO:0007049, GO:0006260, GO:0000278, GO:0051726) obtained via R package biomaRt v.2.54.0[12,13] in addition to standard Seurat cc.genes set.

For integration of data of any of the methods below, the method of gene removal will be indicated in abbreviated code. For example, I(MT, RB, HB) II(CC=0.1, ST=0.25) means that gene sets MT, RB and HB were removed according to the Strategy I and gene sets CC and ST according to the Strategy II with thresholds 0.1 and 0.25 respectively. For each integration (including focused) correlations were recalculated on the subset of cells involved in integration.

Marker gene identification and representation

To identify marker genes that defined specific clusters, the Seurat function FindAllMarkers was used with ranking by AUC (test.use = "roc"). The other filters logfc.threshold and min.pct were relaxed for working with small integrations and clusters. For the focused clusters of human ZG & ZF all clusters had at least one marker gene with AUC > 0.7, which guided their following interpretation. In most dot plots, biologically informative or more specific genes were manually added. In dot plots dedicated to comparing markers of proliferating cortical state (ProlCS) cells (adult vs embryonic and human vs mouse), markers common to ProlCS cells of both groups were identified, as well as those that distinguish each group, provided that the gene remains a marker of proliferating cortical cells.

For the formal intersection of ProlCS marker genes in adult vs fetal humans or mouse vs human an independent analysis was performed. Firstly, differentially expressed genes between ProlCS and all cortical and mesenchymal clusters were identified separately for each dataset. For DE analysis the two-sided Wilcoxon test was employed, followed by adjustment for multiple hypothesis testing with Benjamini-Hochberg method. For that, Seurat function FindMarkers function with parameters only.pos = T, logfc.threshold = 0.05 was runned multiple times to compare ProlCS with any other cluster. For the interspecies analysis only genes with properly identified homologues were included in analysis. Genes with a LogFC > 0.05 and adjusted p-value < 0.05 for each comparison of ProlCS vs any adrenocortical or mesenchymal cluster (intersection) were selected. Secondly, cell cycle genes I(CC) were manually

excluded. Gene set enrichment analysis of adult and fetal (human and mouse) ProlCS marker genes via Gene ontology (GO) - Biological Process (BP) database was performed using compareCluster (fun = enrichGO, OrgDb = org.Hs.eg.db, ont = "BP") function from the clusterProfiler v.4.6.2 R package[14,15].

To construct a cluster hierarchy tree based on the batch-corrected expression matrix of VFs, the expression within each cluster was averaged (AverageExpression function in the Seurat package). Then, a pairwise distance matrix between clusters was obtained using Pearson correlation (cor.dist function), followed by hierarchical clustering using hclust (method = "ave"). For adrenocortical and mesenchymal cell integrations, the tree was rooted at the branch of mesenchymal cells.

Tissue-level integration and cell type annotation

<u>Human adult adrenal data</u>

Eleven human adult samples (for donor information see Table 1) were integrated via Harmony (v.0.1.1)[7] and with exclusion from VFs of genes I(MT, RB, HB) II(CC=0.1). For each scrublet score threshold (see QC section) number of VFs, principal components (PCs) and clustering resolution (parameter resolution of FindClusters function) were chosen to ensure consistency with UMAP and the absence of clusters containing cells of different types based on cell cycle activity. For selected threshold 0.35 they were (nfeatures = 4000, npcs = 55, resolution = 1.5), for strong threshold 0.2 (nfeatures = 2500, npcs = 30, resolution = 1). The parameter max.iter.harmony was set to 20 in the RunHarmony function, while the remaining parameters were left at their default values. UMAP embeddings here and below were generated using RunUMAP with the same number of PCs as used for batch correction.

Each of the clusters was manually annotated based on marker gene expression to one of the following cell types: adrenocortical (*STAR, CYP11A1, CYP11B1*), pericytes or fibroblasts (*LUM*, *PDGFRA*), VSMCs - vascular smooth muscle cells (*ACTA2, TAGLN*), chromaffin cells (*CHGA, PNMT, TH*), SCPs - Schwann cell precursors (*CRYAB, SOX10, FOXD3*), endothelial cells (*PECAM1, KDR, PLVAP*), macrophages (*CTSB, CD136, CD68*), T/Natural killer cells (*CD69, IL7R, CD4*), B cells (*JCHAIN, IGHG1, IGHG3*) or neutrophils (*S100A8, S100A9*).

<u>Mouse adult adrenal data</u>

Sixteen mouse samples were similarly integrated via Harmony with exclusion from VFs of genes I(MT, RB, HB) II(CC=0.1, ST=0.25) and parameters (nfeatures = 2500, npcs = 30, resolution=0.5). Data with weak 0.4 scrublet threshold were integrated using Seurat with parameters (nfeatures = 3000, npcs = 30, resolution = 0.5) and after excluding I(MT, RB, HB) II(CC=0.1) (details below).

Each of the clusters was manually annotated based on marker gene expression to one of the following cell types: adrenocortical (*Star, Cyp11a1, Cyp11b1*), mesenchymal cells (*Sparcl1, Col1a1, Col1a2*), chromaffin cells (*Chga, Pnmt, Th*), SCPs - Schwann cell precursors (*Cryab, Sox10, Foxd3*), endothelial cells (*Pecam1, Kdr, Plvap*), macrophages (*Ctss, Cd68, C1qb*), T/Natural killer cells (*Cd69, Il7r, Cd8a*), B cells (*Igkc, Ighm*) or lastly, neutrophils (*S100a8, S100a9*).

Human fetal adrenal data

6 human fetal samples from Han et al. were similarly integrated via Harmony with exclusion from VFs of genes I(MT, RB, HB, CC) and parameters (nfeatures = 2500, npcs = 35, resolution=0.5). Cell types were annotated by standart Seurat label transfer procedure with 30 PCs and Kameneva et al.[10] dataset as a reference.

A UMAP embedding for Jansky et al.[8] dataset was generated based on Harmony integration with exclusion from VFs of genes I(MT, RB, HB, CC) and parameters (nfeatures = 2500, npcs = 20).

Target integrations of adrenocortical and mesenchymal cells

Human adult adrenal data

Adrenocortical and mesenchymal cells were re-normalised (SCTransform) and re-integrated using Seurat CCA (ncomps = 35) according to the vignette (SelectIntegrationFeatures, PrepSCTIntegration, FindIntegrationAnchors, IntegrateData), with the only difference being that a larger number of genes were pre-selected for integration in order to obtain the required number (nfeatures = 3500) after excluding I(MT, RB, HB) II(CC=0.1, ST=0.25). For subsequent steps (RunPCA, RunUMAP, FindNeighbors) here and below the same number of components was used as for integration (npcs = 35). FindClusters resolution parameter was 0.5. For all other integrations described, Seurat integration specifically denotes CCA approach with SCTransform normalization, unless otherwise stated.

Following integration, a small outlier cluster of 91 cells lacking specific markers and represented by only 2 of 11 samples emerged. It was manually removed (subset function), and the RunPCA, RunUMAP, FindNeighbors, FindClusters steps were repeated with npcs = 30.

Embeddings focused on the central part or zona glomerulosa of the embedding of adrenocortical and mesenchymal cells were obtained by selecting cells from the relevant clusters (see corresponding figures) and performing subsequent centering ScaleData (do.center = T, do.scale = F), PCA, UMAP and clustering steps. The previous batch-corrected matrix (adrenocortical and mesenchymal cells) was used, and full re-integration was not performed. After cell subsetting VFs were ordered by standard deviation and top nfeatures VFs were selected. For the focused on central part embedding (nfeatures = 2500, npcs = 30, resolution = 0.5) a fraction of cells in one of clusters expressed immune marker genes (*PTPRC, CD163, CD69*), these cells were manually removed and embedding recalculated. Original adrenocortical cluster 7:ZF was represented by two nearly identical clusters without clear discriminative features, so they were manually joined. BRIDGE cells were part of the TRANS-RGS5 cluster and were manually selected as an additional cluster. The focused on zona glomerulosa embedding was calculated with parameters nfeatures = 2500, npcs = 30, resolution = 0.5.

The same re-clustering procedure was used to split the cluster with increased cholesterol biosynthesis (14:CHOL) into two parts with expression of ZG or ZR signature (nfeatures = 3000, npcs = 20, resolution = 0.5).

ForceAtlas2[16] (FA2) embedding of human ZG & ZF was calculated via the ReductionWrappers v.2.5.4 package[17], which utilises reticulate v.1.28 to use fa2 v.0.3.5 package[18] with Python v.3.8.20. Firstly, focused t-SNE embedding (RunTSNE Seurat function) of ZG and ZF clusters (nfeatures = 3000, npcs = 20) was calculated in a similar manner to two previous focused objects. Secondly, function DoForceAtlas2 was runned on the resulting Seurat object with parameter reduction_use = "tsne".

Gene expression of zona glomerulosa signature (*SHOC1, CABP7, CYP11B2, KCNJ5, APCDD1, GABBR2, LGR5, WNT4, CCN3, VSNL1*) and zona reticularis signature (*CYB5A, FGG, ORM1, SLC13A5, SLC27A2*) were calculated using the AddModuleScore function from Seurat, and the above mentioned gene sets were obtained from Baker et al.[19].

Mouse adult adrenal data

Mouse adrenocortical and mesenchymal cells (obtained after filtering with weak 0.4 scrublet threshold) integration was performed similarly using Seurat with parameters (nfeatures = 3000, npcs = 30, resolution = 0.5) and after excluding I(MT, RB, HB) II(CC=0.1, ST=0.25). Next, three clusters with expression of immune marker genes (*Ptprc, C1qa, C1qb*), endothelial marker genes (*Kdr, Pecam1, Plvap, Cdh5*) or cells expressing < 500 distinct genes were manually removed. The remaining cells were re-integrated using Seurat with parameters (nfeatures = 3000, npcs = 30, resolution = 0.5) and after excluding newly identified I(MT, RB, HB) II(CC=0.1, ST=0.25).

Mouse focused zona glomerulosa FA2 embedding was obtained similarly to the human one with the same parameters. To detect X zone cells on the tail of 14:ZF4-Sbsn cluster of adrenocortical & mesenchymal cells integration, cells of this cluster were re-integrated using the Harmony approach with parameters (nfeatures = 800, npcs = 8, resolution = 0.5) and same genes exclusion as for the original integration. *Pik3c2g+Akr1c18+* cluster was defined as X zone cells.

Human fetal adrenal data

As datasets from Kameneva et al.[10] and Han et al.[20] contained *PAX2+* liver cells that were closely related to the adrenocortical cells on the embedding, it was necessary to perform two rounds of Harmony integration and *PAX2+* cluster removal for each dataset. For Kameneva et al. Harmony parameters were (nfeatures = 4000, npcs = 40) and for Han et al. (nfeatures = 2500, npcs = 35), excluded from VFs genes were I(MT, RB, HB, CC). Before integrating datasets, gene names in all count matrices were updated using HGNC website https://www.genenames.org/tools/multi-symbol-checker/.

37 fetal and neonatal samples with > 800 target cells/nuclei were integrated using Seurat with parameters (nfeatures = 3000, npcs = 40, resolution = 0.5) and after excluding I(MT, RB, HB) II(CC=0.25, ST=0.4). To avoid the error that arises in Seurat when integrating with a large number of anchors (sparse matrix with more than 2^31 non-zero elements), the samples were divided into four groups and integration was performed in two stages - first within each group, and then between the resulting four

objects. The grouping was done according to the hierarchy for Seurat integrations through pairwise integration of samples.

Human adult and fetal data integration - all data

49 fetal, neonatal and adult samples were integrated similarly to fetal integration, but five groups for integration, I(MT, RB, HB) II(CC=0.25, ST=0.3) genes were excluded and VFs were ranked accordingly to average between adult and fetal variability ranks.

Some additional adult and fetal integrations of 10x cells only data (without 10x nuclei and Microwell-seq samples, only Kameneva et al.[10] and Kildisiute et al.[9] datasets were included) has been performed to decrease batch effects and obtain more interpretable integration with ability to accurately resolve cortical heterogeneity. Initially, adrenocortical and mesenchymal cells form 27 fetal and adult samples were integrated using SCTransform (min_cells = 2) and Seurat (nfeatures = 3000, npcs = 40, resolution = 0.5) and after excluding I(MT, RB, HB) II(MT=0.45, RB=0.45, CC=0.35, ST=0.25). Then, only adrenocortical cells (without mesenchymal and transitional bridges) were extracted from the resulting integration, re-normalized and re-integrated with the same parameters. Finally, focused embeddings for the left and right parts of the resulting adrenocortical integration were re-calculated with parameters nfeatures = 3000, npcs = 40, resolution = 0.5.

Adult human and mouse (cross-species) data integration

Human and mouse samples were integrated using Seurat (nfeatures = 4000, npcs = 35, resolution = 0.5, k.filter = 600). Samples were integrated in two consecutive steps - firstly within and then between species. Gene homologues were identified using biomaRt and by uppercase gene name, VFs were ranked according to average between human and mouse variability ranks. To remove undesirable effects from VFs, all genes that were earlier excluded in mouse or human integrations were excluded.

Since zona reticularis is absent in mouse adrenal cortex and late ZF also has *CYP17A1*-mediated 17-alpha-hydroxylase human-specific activity, we selected ZG and intermediate between ZG and ZF (early ZF) clusters for additional focused interspecies integration. Only samples with >200 target cells were included. Genes *HSD3B2* and *Hsd3b6* were considered as homologues. Finally, 9 human and 13 mouse samples were integrated using SCTransform (min_cells = 2) and Seurat (nfeatures = 2500, npcs = 20, resolution = 0.5). To remove undesirable effects from VFs, all genes that were earlier excluded in mouse or human integrations were excluded.

For ZG and early ZF cells, a formal cross-species heterogeneity comparison was performed via ROGUE entropy approach[21]. Firstly, we selected a common set of variable genes (76 genes in total) for all human and mouse samples with at least 100 cells, each gene in this set was expressed at least in 10 cells of each sample and formally identified as variable with adjusted p-value < 0.05 in at least two samples. Secondly, the ROGUE entropy metric was estimated using this set of genes in each selected sample. Entropies in human and mouse groups were compared via two-sided Wilcoxon test.

Target integrations of medullary cells

Human adult data

8 human adult samples with > 20 medullary cells (SCPs and chromaffin cells) were integrated using conos v.1.5.0 R package[22] (n.odgenes = 800, space = "PCA", ncomps = 20, k = 30, k.self = 5). Before integration, individual samples were normalized using basicP2proc (n.odgenes = 800, nPcs = 10) function from pagoda2 package. Before normalization I(MT, RB, HB, CC) genes were excluded from count matrices.

6 human adult samples with > 20 chromaffin cells were similarly normalized with parameters (n.odgenes = 100, nPcs = 2) after exclusion of genes I(MT, RB, HB, CC) II(ST=0.2, CORT=0.25) and integrated (n.odgenes = 100, ncomps = 4, space = "CPCA", k = 30, k.self = 5).

Mouse adult data

15 mouse samples with > 20 medullary cells (SCPs and chromaffin cells) (obtained after filtering with weak 0.4 scrublet threshold) integration was performed similarly using conos with parameters (n.odgenes = 1000, space = "CPCA", ncomps = 5, k = 10, k.self = 5) and after excluding I(MT, RB, HB, CC) II(ST=0.25) and basicP2proc default normalization. Next, two clusters with expression of adrenocortical marker genes (*Star, Cyp11a1, Cyp11b1*) or endothelial marker genes (*Kdr, Pecam1, Plvap, Cdh5*) were manually removed. The remaining cells were re-normalized and re-integrated with parameters (n.odgenes = 800, space = "CPCA", ncomps = 5, k = 15, k.self = 5).

Human fetal data

31 human fetal samples with > 100 medullary cells (SCPs, chromaffin cells, sympathoblasts) were integrated using Seurat (nfeatures = 3000, npcs = 30, resolution = 0.5) and after excluding I(MT, RB, HB, CC). Next, a cluster of cells with expression of adrenocortical marker genes (*STAR, CYP11B1, CYP11A1, CYP17A1*) was manually removed. The remaining cells were re-integrated using Seurat with parameters (nfeatures = 2000, npcs = 20, resolution = 0.3).

Target integrations of mesenchymal cells

Human adult adrenal data

10 human adult samples with > 20 mesenchymal cells (Fibroblasts and VSMCs) were integrated using Seurat (nfeatures = 800, npcs = 10, resolution = 0.5) and after excluding I(HB) II(MT=0.15, RB=0.2, CC=0.2, ST=0.2). Due to the small number of cells in the samples, additional parameters were set during SCTransform normalization (min_cells = 2) and Seurat integration (k.score = 20, k.weight = 20).

Mouse adult adrenal data

11 mouse samples with > 20 mesenchymal cells (Perivascular) were integrated using Seurat (nfeatures = 1000, npcs = 10, resolution = 0.5) and after excluding I(HB) II(MT=0.25, RB=0.45, CC=0.2, ST=0.2). Due to the small number of cells in the samples, additional parameters were set during SCTransform normalization

(min_cells = 2) and Seurat integration (k.score = 50, k.weight = 50). Next, a cluster with expression of endothelial marker genes (*Kdr, Pecam1, Plvap*) was manually removed. The remaining cells were re-integrated using Seurat with the same parameters.

Human fetal adrenal data

35 human fetal samples with > 100 mesenchymal cells were integrated using Seurat (nfeatures = 1000, npcs = 15, resolution = 0.5) and after excluding I(RB, HB) II(MT=0.35, CC=0.2, ST=0.6). Parameter (min_cells = 2) was set during SCTransform normalization.

Target integrations of endothelial cells

Human adult adrenal data

11 (all) human adult samples with > 150 endothelial cells were integrated using Seurat (nfeatures = 1500, npcs = 20, resolution = 0.5) and after excluding I(HB) II(MT=0.2, RB=0.3, CC=0.2, ST=0.2). Parameter (min_cells = 2) was set during SCTransform normalization.

Mouse adult adrenal data

16 (all) mouse adult samples with > 100 endothelial cells were integrated using Seurat (nfeatures = 1500, npcs = 20, resolution = 0.5) and after excluding I(HB) II(MT=0.05, RB=0.25, CC=0.2, ST=0.2). Parameter (min_cells = 2) was set during SCTransform normalization. Next, two clusters with expression of adrenocortical marker genes (*Star, Cyp11a1, Hsd3b1*) or macrophage marker genes (*C1qa, C1qb, C1qc*) were manually removed. The remaining cells were re-integrated using Seurat with the same parameters.

Target integrations of immune cells

Human adult adrenal data

10 human adult samples with > 20 immune cells were integrated using Harmony (npcs = 30, theta = 0.5), VFs were set to top 2,500 VFs of single sample "Female 53 years (1)", which had low ambient RNA level and > 1300 immune cells. Next, a cluster with expression of adrenocortical marker gene *STAR* was manually removed and one sample with few target cells was excluded. The remaining 9 samples were re-integrated using Harmony with the same parameters.

Mouse adult adrenal data

16 (all) mouse samples with > 40 immune cells were integrated via Harmony with exclusion from VFs of genes I(HB) II(MT=0.15, RB=0.4, CC=0.2, ST=0.3) and parameters (nfeatures = 3000, npcs = 30, resolution=0.5). Next, three clusters composed of 714 cells characterized by expression of adrenocortical (*Star, Cyp11a1, Hsd3b1*), endothelial (*Kdr, Pecam1, Plvap*) or perivascular (*Igfbp7, Col1a2, Dcn*) marker genes were manually removed. Then, UMAP embedding was recalculated.

Description and links to data available on cell x gene

Human_adult_adrenal_all_cell_types.h5ad - Tissue-level embedding of the human adult adrenal gland, focused embeddings of medullary, chromaffin, endothelial, mesenchymal and immune cell types;

Human_adult_cortex_mesenchyme.h5ad - Human adult adrenocortical & mesenchymal cells embedding, focused parts of cortical embedding - ZG, ZG & ZF, central part;

HumanLowResVisiumAllSamples.h5ad - Human adult Visium low resolution spatial transcriptomics (4 spatial samples and joint UMAP);

HumanStereoSeqAllSamples.h5ad - Human adult Stereo-seq high resolution spatial transcriptomics (3 spatial samples with annotated cells);

Human_fetal_adrenal_main_cell_types.h5ad - Human fetal adrenocortical & mesenchymal cells embedding, fetal medulla cells (SCPs + chromaffin + sympathoblasts) embedding, focused fetal mesenchyme embedding;

Human_fetal_comparison_adrenal.h5ad - Human adult and fetal adrenocortical & mesenchymal cells joint embedding (all cells & nuclei), human adult and fetal adrenocortical & mesenchymal cells joint embedding (only 10X cells), focused cortical, DZ and FZ parts of the 10X embedding;

Mouse_adult_adrenal_all_cell_types.h5ad - Tissue-level embedding of the mouse adult adrenal gland, focused embeddings of medullary, chromaffin, endothelial, mesenchymal and immune cell types. Also includes focused adrenocortical & mesenchymal cells embedding, focused ZG & ZF-Ptn cells embedding;

Human_Mouse_comparison_adult_adrenal.h5ad - Adult mouse and human cross-species adrenocortical & mesenchymal cells joint embedding, focused ZG cells re-integration;

MouseVisiumHDAllSamples.h5ad- Mouse adult VisiumHD high resolution spatial transcriptomics (11 spatial samples with annotated cells).

Table 1. Information on human adrenal tissue donors. Cause of death among the 19 donors included cerebral ischemia (n = 4), intracerebral bleeding (n = 5), subarachnoidal bleeding (n = 3), suicide (n = 2), accident (n = 1) and unknown/unspecified (n = 4).

Sex / age	Method of analysis
Female / 18 yo	Single cell transcriptomics, histological validations
Female / 19 yo	Spatial transcriptomics (10x Genomics Visium)
Female / 20 yo	Spatial transcriptomics (StereoSeq), histological validations
Female / 43 yo	Histological validations
Female / 53 yo No 1	Single cell transcriptomics, histological validations
Female / 53 yo No 2	Single cell transcriptomics
Female / 59 yo	Single cell transcriptomics
Female / 61 yo	Spatial transcriptomics (10x Genomics Visium)
Female / 64 yo	Single cell transcriptomics
Male / 13 yo	Spatial transcriptomics (StereoSeq)
Male / 21 yo	Spatial transcriptomics (10x Genomics Visium)
Male / 31 yo	Histological validations
Male / 39 yo	Single cell transcriptomics
Male / 40 yo	Spatial transcriptomics (StereoSeq), histological validations
Male / 48 yo	Single cell transcriptomics, histological validations
Male / 52 yo	Single cell transcriptomics<br>Spatial transcriptomics (10x Genomics Visium), histological validations
Male / 54 yo	Single cell transcriptomics
Male / 57 yo	Single cell transcriptomics
Male / 73 yo	Single cell transcriptomics

Supplementary information on primary and secondary antibodies used for immunofluorescence

Primary antibodies used: anti-VSNL1 (1: 500, Sigma-Aldrich, #MABN762, LOT 3959519), anti-KI67 (1:200, Zytomed, #RBK027-05, LOT V223), anti-SF1 (1:50, R&D Systems, #PP-N1665-0C, LOT B-4), anti-CYP11B1/2 (1:500, Sigma-Aldrich, #HPA049171, LOT 000020197), anti-EZH2 (1:50, Fisher Scientific, #MA5-18108, clone 144CT2.1.1.5, LOT 79751985), monoclonal anti-CYP11B2 (1:50, Millipore, MABS1251, clone 41-17B, LOT4206942), anti-HSD3B2 (1:200, Sigma-Aldrich, #HPA043261, LOT R39655), anti-CYP21A2 (1:1000, Sigma-Aldrich, #HPA053371, LOT 000013564), anti-NOV/CCN3 (1:100, R&D Systems, #AF1640, LOT JKB022410A), anti-DLK1 (1:500, Sigma-Aldrich, #HPA062262, LOT 000017636), anti-CYP17A1 (1:250, Abcam, #ab134910, LOT 1018866-22), anti-KCNJ5 (1:500, Fisher Scientific, #PA5-36967, LOT 79748411).

Secondary antibodies used: donkey anti-mouse, rabbit or goat-Alexa-488/546 or 647-conjugated secondary antibodies (1:1000, Invitrogen).

Supplementary information on RNAscope probes used

Human-specific probes with proprietary sequences were used according to manufacturer's instructions (ACD Bio): *HSD3B2* (Cat No 467681, 467681-C3), *WNT4* (Cat No 429441), *CYP17A1* (Cat No 467251-C2), *CCDC80* (Cat No 1153161-C2), *CCN3* (Cat No 412051-C2), *CYP11B1* (Cat No 882221), *VCAN* (Cat No 430071-C3).

Supplementary References

1. Nunes, J. B. *et al.* Integration of mass cytometry and mass spectrometry imaging for spatially resolved single-cell metabolic profiling. *Nat Methods* 1–5 (2024) doi:10.1038/s41592-024-02392-6.
2. Stuart, T. *et al.* Comprehensive Integration of Single-Cell Data. *Cell* **177**, 1888-1902.e21 (2019).
3. Hao, Y. *et al.* Integrated analysis of multimodal single-cell data. *Cell* **184**, 3573-3587.e29 (2021).
4. kharchenkolab/pagoda2. Kharchenko Lab (2024).
5. Wolock, S. L., Lopez, R. & Klein, A. M. Scrublet: Computational Identification of Cell Doublets in Single-Cell Transcriptomic Data. *Cell Systems* **8**, 281-291.e9 (2019).
6. Young, M. D. & Behjati, S. SoupX removes ambient RNA contamination from droplet-based single-cell RNA sequencing data. *GigaScience* **9**, giaa151 (2020).
7. Korsunsky, I. *et al.* Fast, sensitive and accurate integration of single-cell data with Harmony. *Nat Methods* **16**, 1289–1296 (2019).
8. Jansky, S. *et al.* Single-cell transcriptomic analyses provide insights into the developmental origins of neuroblastoma. *Nat Genet* **53**, 683–693 (2021).
9. Kildisiute, G. *et al.* Tumor to normal single-cell mRNA comparisons reveal a pan-neuroblastoma cancer cell. *Science Advances* **7**, eabd3311 (2021).
10. Kameneva, P. *et al.* Single-cell transcriptomics of human embryos identifies multiple sympathoblast lineages with potential implications for neuroblastoma origin. *Nat Genet* **53**, 694–706 (2021).
11. Han, X. *et al.* Construction of a human cell landscape at single-cell level. *Nature* **581**, 303–309 (2020).
12. Durinck, S. *et al.* BioMart and Bioconductor: a powerful link between biological databases and microarray data analysis. *Bioinformatics* **21**, 3439–3440 (2005).
13. Durinck, S., Spellman, P. T., Birney, E. & Huber, W. Mapping identifiers for the integration of genomic datasets with the R/Bioconductor package biomaRt. *Nat Protoc* **4**, 1184–1191 (2009).
14. Xu, S. *et al.* Using clusterProfiler to characterize multiomics data. *Nat Protoc* **19**, 3292–3320 (2024).
15. Yu, G. Thirteen years of clusterProfiler. *The Innovation* **5**, 100722 (2024).
16. Jacomy, M., Venturini, T., Heymann, S. & Bastian, M. ForceAtlas2, a Continuous Graph Layout Algorithm for Handy Network Visualization Designed for the Gephi Software. *PLOS ONE* **9**, e98679 (2014).
17. Smith, M. milescsmith/ReductionWrappers. (2025).
18. Chippada, B. bhargavchippada/forceatlas2. (2025).
19. Baker, J. E. *et al.* Targeted RNA sequencing of adrenal zones using immunohistochemistry-guided capture of formalin-fixed paraffin-embedded tissue. *Molecular and Cellular Endocrinology* **530**, 111296 (2021).
20. Han, X. *et al.* Construction of a human cell landscape at single-cell level. *Nature* **581**, 303–309 (2020).
21. Liu, B. *et al.* An entropy-based metric for assessing the purity of single cell populations. *Nat Commun* **11**, 3155 (2020).

22. Barkas, N. *et al.* Joint analysis of heterogeneous single-cell RNA-seq dataset collections. *Nat Methods* **16**, 695–698 (2019).

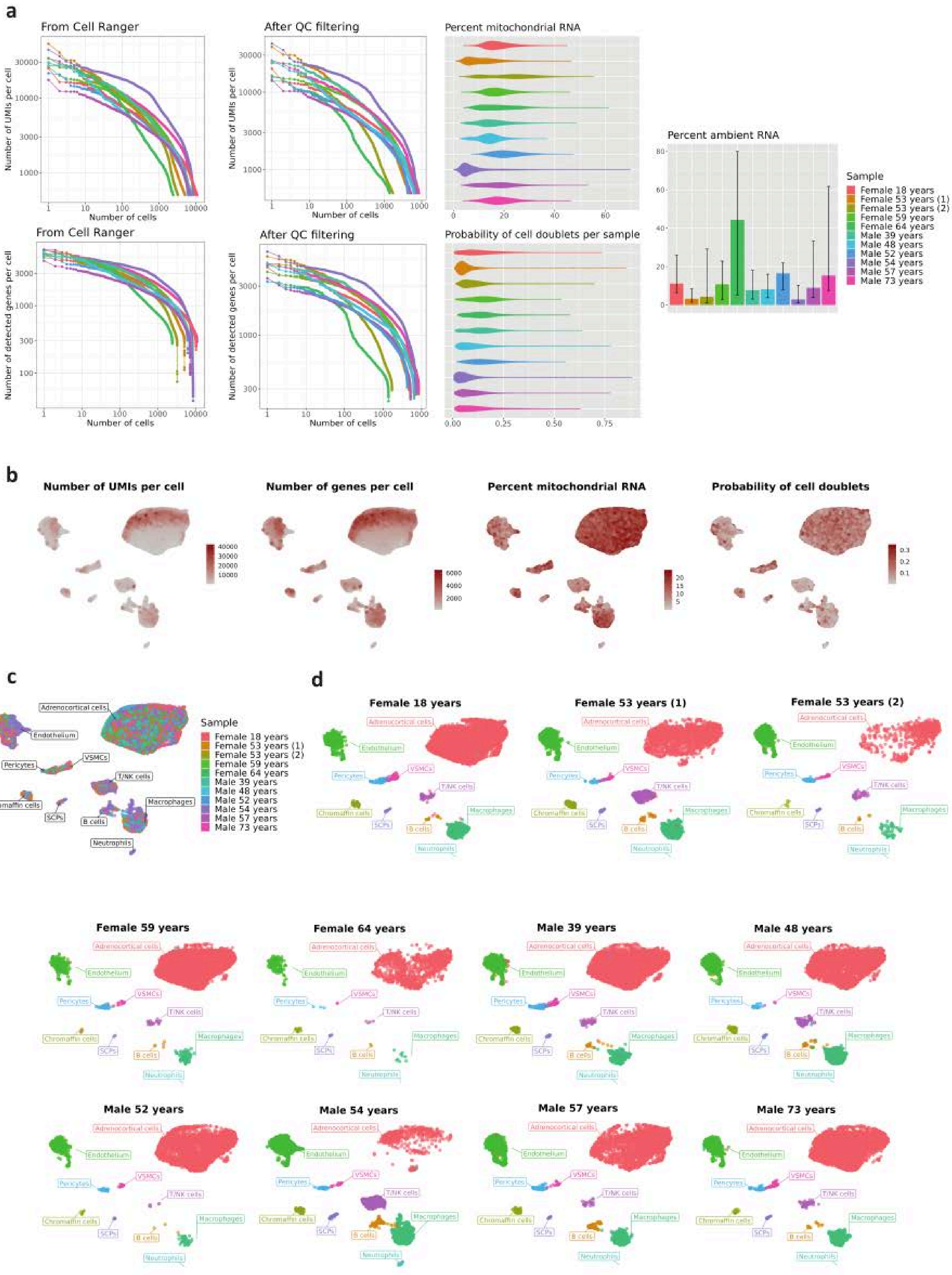

a
From Cell Ranger
After QC filtering
Percent mitochondrial RNA
Percent ambient RNA
Probability of cell doublets per sample
Number of UMIs per cell
Number of detected genes per cell
Number of cells
Sample
Female 18 years
Female 53 years (1)
Female 53 years (2)
Female 59 years
Female 64 years
Male 39 years
Male 48 years
Male 52 years
Male 54 years
Male 57 years
Male 73 years
b
Number of UMIs per cell
Number of genes per cell
Percent mitochondrial RNA
Probability of cell doublets
c
d
Adrenocortical cells
Endothelium
Pericytes
VSMCs
T/NK cells
Chromaffin cells
SCPs
B cells
Macrophages
Neutrophils

Supplementary Fig. 1 | Technical quality controls for scRNA-seq data of human adult adrenal gland. a, Numbers of detected genes per cell (upper panel) and numbers of UMIs per cell (lower panel) in each sample before (From Cell Ranger) and after QC filtering (After QC filtering). Fraction of mitochondrial RNA (Percent mitochondrial RNA) and Scrublet score (Probability of cell doublets per sample) distributions in each sample. SoupX estimated median fraction of ambient RNA (Percent ambient RNA) in each sample, error bars represent 95% confidence interval. The color legend is consistent across all plots. **b**, Number of UMIs per cell, Number of genes per cell, Percent mitochondrial RNA and Probability of cell doublets (Scrublet scores) after all QC filtrations and ambient RNA correction on the general UMAP embedding (60,146 cells). **c**, Per sample compositional impact on the general UMAP embedding (60,146 cells). **d**, UMAP embedding subset of cells from each individual human sample.

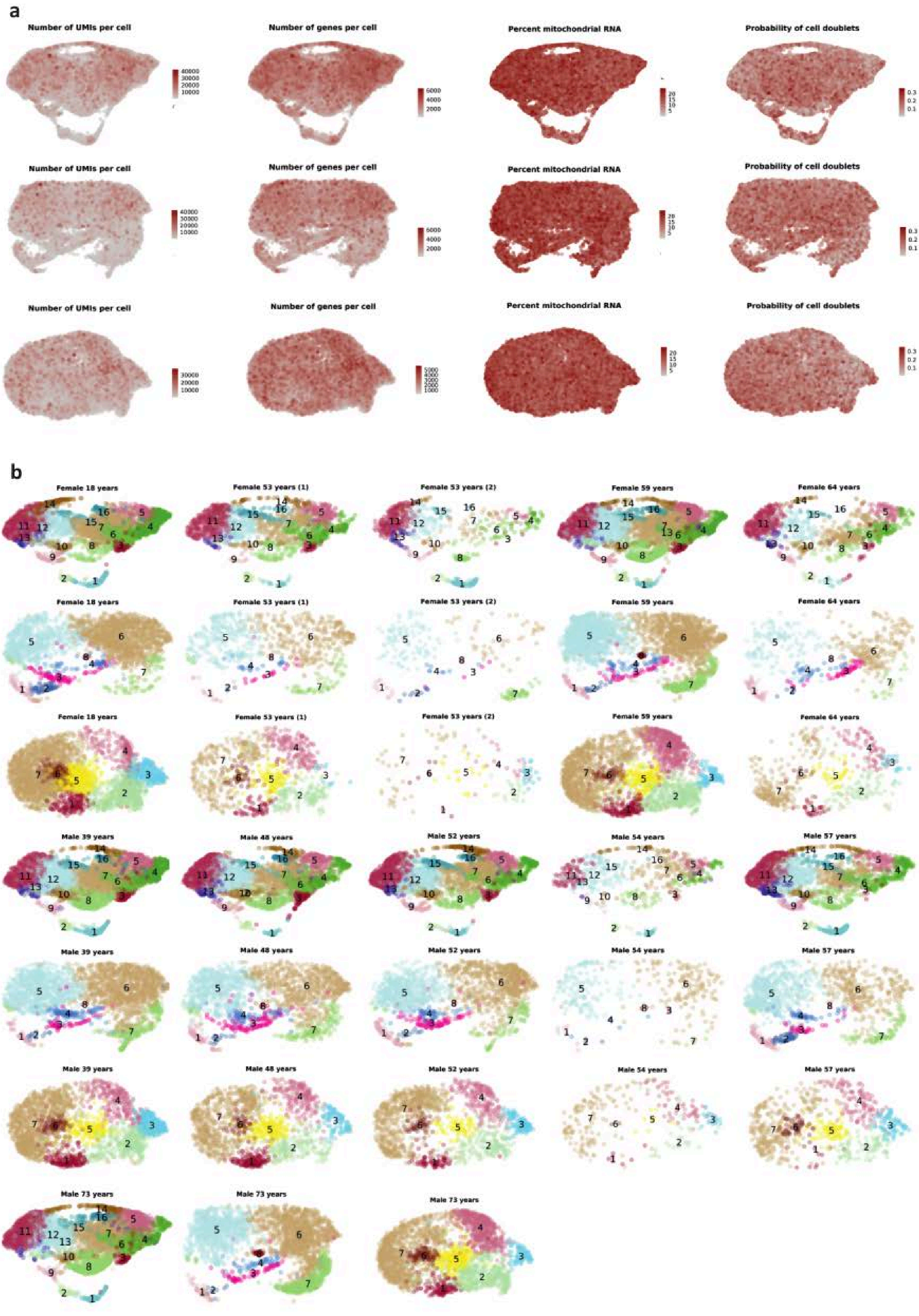
a
Number of UMIs per cell
Number of genes per cell
Percent mitochondrial RNA
Probability of cell doublets
40000
30000
20000
10000
6000
4000
2000
20
15
10
5
0.3
0.2
0.1
Number of UMIs per cell
Number of genes per cell
Percent mitochondrial RNA
Probability of cell doublets
Number of UMIs per cell
Number of genes per cell
Percent mitochondrial RNA
Probability of cell doublets
30000
20000
10000
5000
4000
3000
2000
1000
b
Female 18 years
Female 53 years (1)
Female 53 years (2)
Female 59 years
Female 64 years
Male 39 years
Male 48 years
Male 52 years
Male 54 years
Male 57 years
Male 73 years

Supplementary Fig. 2 | QC metrics and per sample compositional impact of human adult adrenal cortex. a, Number of UMIs per cell, Number of genes per cell, Percent mitochondrial RNA and Probability of cell doublets (Scrublet scores) on the adrenocortical and mesenchymal cells embedding (39,985 cells), focused on central part embedding and focused (16,926 cells) on zona glomerulosa embedding (17,260 cells). **b**, Per sample compositional impact - UMAP embedding subset of cells from each individual human sample. Subsets are shown for general adrenocortical and mesenchymal cells embedding, focused on central part embedding and focused on zona glomerulosa embedding with related clusterization.

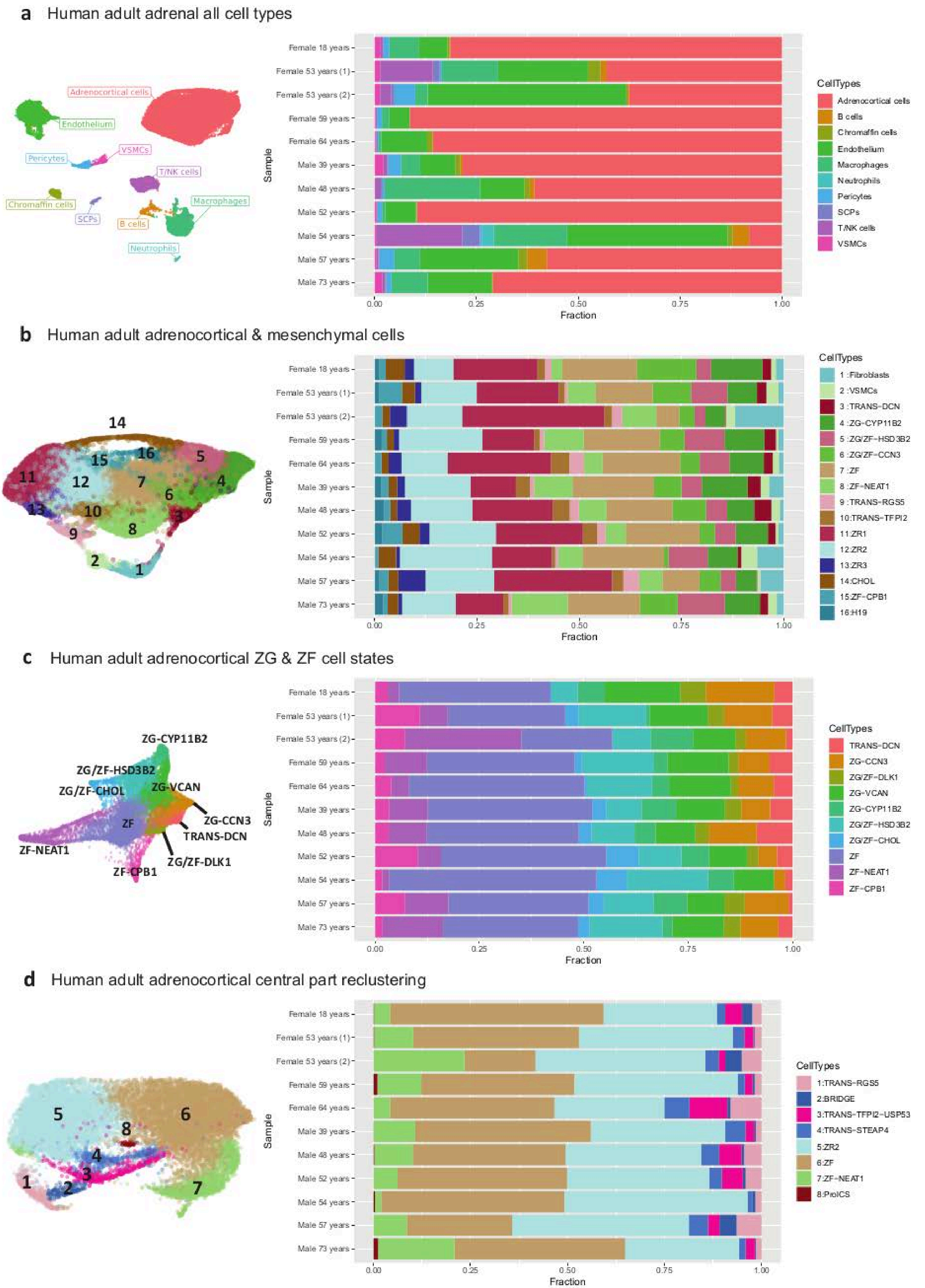

a Human adult adrenal all cell types
Adrenocortical cells
Endothelium
Pericytes
VSMCs
T/NK cells
Chromaffin cells
SCPs
B cells
Macrophages
Neutrophils
Female 18 years
Female 53 years (1)
Female 53 years (2)
Female 59 years
Female 64 years
Male 39 years
Male 48 years
Male 52 years
Male 54 years
Male 57 years
Male 73 years
Sample
0.00
0.25
0.50
0.75
1.00
Fraction
CellTypes
Adrenocortical cells
B cells
Chromaffin cells
Endothelium
Macrophages
Neutrophils
Pericytes
SCPs
T/NK cells
VSMCs
b Human adult adrenocortical & mesenchymal cells
14
15
16
5
11
12
7
4
6
13
10
3
9
8
2
1
CellTypes
1 :Fibroblasts
2 :VSMCs
3 :TRANS-DCN
4 :ZG-CYP11B2
5 :ZG/ZF-HSD3B2
6 :ZG/ZF-CCN3
7 :ZF
8 :ZF-NEAT1
9 :TRANS-RGS5
10:TRANS-TFPI2
11:ZR1
12:ZR2
13:ZR3
14:CHOL
15:ZF-CPB1
16:H19
c Human adult adrenocortical ZG & ZF cell states
ZG-CYP11B2
ZG/ZF-HSD3B2
ZG/ZF-CHOL
ZG-VCAN
ZF
ZG-CCN3
TRANS-DCN
ZF-NEAT1
ZG/ZF-DLK1
ZF-CPB1
CellTypes
TRANS-DCN
ZG-CCN3
ZG/ZF-DLK1
ZG-VCAN
ZG-CYP11B2
ZG/ZF-HSD3B2
ZG/ZF-CHOL
ZF
ZF-NEAT1
ZF-CPB1
d Human adult adrenocortical central part reclustering
5
6
8
4
3
1
2
7
CellTypes
1:TRANS-RGS5
2:BRIDGE
3:TRANS-TFPI2-USP53
4:TRANS-STEAP4
5:ZR2
6:ZF
7:ZF-NEAT1
8:ProICS

Supplementary Fig. 3 | Compositional contribution of each sample in human adult scRNA-seq embeddings. a-d, For each embedding (left) the fractions of cell types or states in each sample are shown as a bar plot (right). **a**, The UMAP embedding of the human adult adrenal gland cells, color coded by cell type (60,146 cells). **b**, The UMAP embedding resulting from integration of adrenocortical and mesenchymal cells (39,985 cells). **c**, The ForceAtlas2 (FA2) embedding illustrates zona glomerulosa (ZG) & zona fasciculata (ZF) (20,255 cells). **d**, The UMAP embedding with focused central part of the common embedding and proliferating cortical cells (16,926 cells).

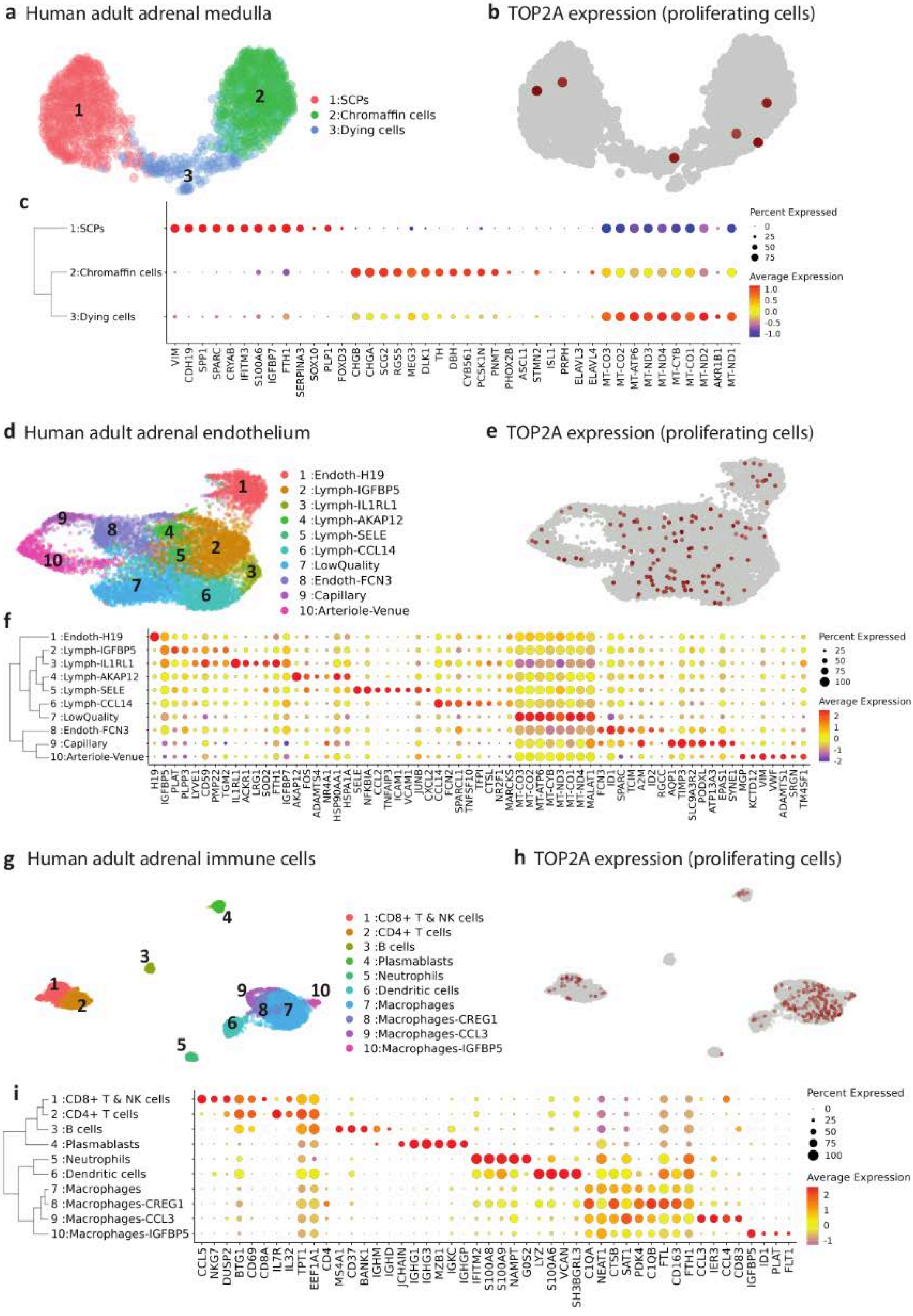
a Human adult adrenal medulla
1:SCPs
2:Chromaffin cells
3:Dying cells
b TOP2A expression (proliferating cells)
c
Percent Expressed
0
25
50
75
Average Expression
1.0
0.5
0.0
-0.5
-1.0
VIM CDH19 SPP1 SPARC CRYAB IFITM3 S100A6 IGFBP7 FTH1 SERPINA3 SOX10 PLP1 FOXD3 CHGB CHGA SCG2 RGS5 MEG3 DLK1 TH DBH CYB561 PCSK1N PNMT PHOX2B ASCL1 STMN2 ISL1 PRPH ELAVL3 ELAVL4 MT-CO3 MT-CO2 MT-ATP6 MT-ND3 MT-ND4 MT-CYB MT-CO1 MT-ND2 AKR1B1 MT-ND1
d Human adult adrenal endothelium
1 :Endoth-H19
2 :Lymph-IGFBP5
3 :Lymph-IL1RL1
4 :Lymph-AKAP12
5 :Lymph-SELE
6 :Lymph-CCL14
7 :LowQuality
8 :Endoth-FCN3
9 :Capillary
10:Arteriole-Venue
e TOP2A expression (proliferating cells)
f
Percent Expressed
25
50
75
100
Average Expression
2
1
0
-1
-2
H19 IGFBP5 PLAT PLPP3 LYVE1 CD59 PMP22 TGM2 IL1RL1 ACKR1 LRG1 SOD2 FTH1 IGFBP7 AKAP12 FOS ADAMTS4 NR4A1 HSP90AA1 HSPA1A SELE NFKBIA CCL2 TNFAIP3 ICAM1 VCAM1 JUNB CXCL2 CCL14 FCN2 SPARCL1 TNFSF10 TFPI CTSL NR2F1 MARCKS MT-CO3 MT-CO2 MT-ATP6 MT-CYB MT-ND3 MT-CO1 MT-ND4 MALAT1 FCN3 ID1 SPARC TCIM A2M ID2 RGCC AQP1 TIMP3 SLC9A3R2 PODXL ATP13A3 EPAS1 SYNE1 MGP KCTD12 VIM VWF ADAMTS1 SRGN TM4SF1
g Human adult adrenal immune cells
1 :CD8+ T & NK cells
2 :CD4+ T cells
3 :B cells
4 :Plasmablasts
5 :Neutrophils
6 :Dendritic cells
7 :Macrophages
8 :Macrophages-CREG1
9 :Macrophages-CCL3
10:Macrophages-IGFBP5
h TOP2A expression (proliferating cells)
i
Percent Expressed
0
25
50
75
100
Average Expression
2
1
0
-1
CCL5 NKG7 DUSP2 BTG1 CD69 CD8A IL7R IL32 TPT1 EEF1A1 CD4 MS4A1 CD37 BANK1 IGHM IGHD JCHAIN IGHG1 IGHG3 MZB1 IGKC IGHGP IFITM2 S100A8 S100A9 NAMPT G0S2 LYZ S100A6 VCAN SH3BGRL3 C1QA NEAT1 CTSB SAT1 PDK4 C1QB FTL CD163 FTH1 CCL3 IER3 CCL4 CD83 IGFBP5 ID1 PLAT FLT1

Supplementary Fig. 4 | Transcriptional heterogeneity of human adult adrenal medulla, endothelium and immune cells. a-c, Human adult adrenal medulla (1,082 cells). **d-f**, Human adult adrenal endothelium (9,865 cells). **g-i**, Human adult adrenal immune cells (8,233 cells). **a,d,g**, A UMAP embedding resulting from the focused reintegration of target human adrenal cell population. **b,e,h**, *TOP2A* gene expression indicates location of proliferating cells on UMAP. **c,f,i**, DotPlot representation of marker genes for subpopulations of target human adrenal cell population. The hierarchy of clusters, based on transcriptional similarity, is shown on the left.

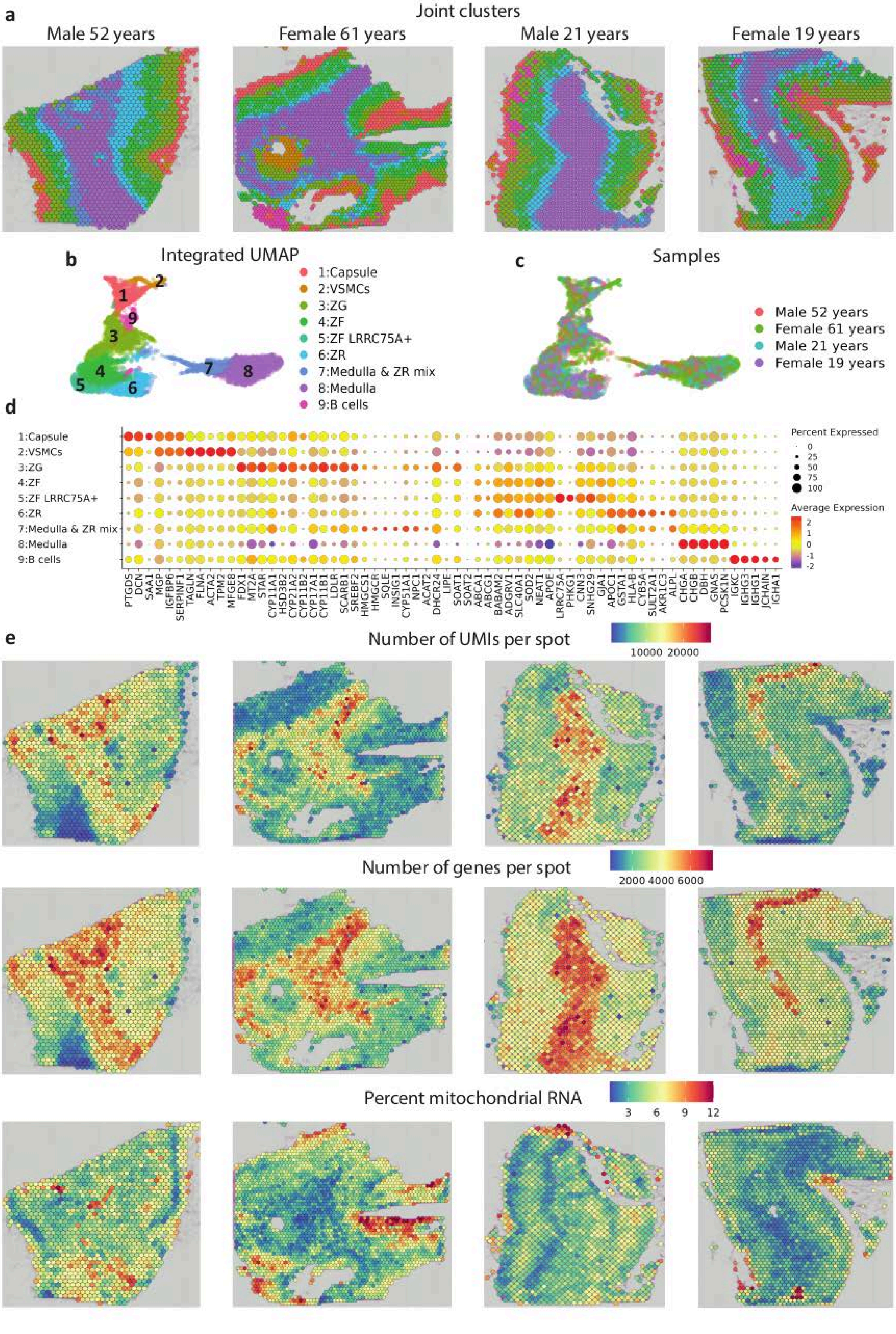

a
Joint clusters
Male 52 years
Female 61 years
Male 21 years
Female 19 years
b
Integrated UMAP
1:Capsule
2:VSMCs
3:ZG
4:ZF
5:ZF LRRC75A+
6:ZR
7:Medulla & ZR mix
8:Medulla
9:B cells
c
Samples
Male 52 years
Female 61 years
Male 21 years
Female 19 years
d
Percent Expressed
0
25
50
75
100
Average Expression
2
1
0
-1
-2
PTGDS DCN SAA1 MGP IGFBP6 SERPINF1 TAGLN FLNA ACTA2 TPM2 MFGE8 FDX1 MT2A STAR CYP11A1 HSD3B2 CYP21A2 CYP11B2 CYP17A1 CYP11B1 LDLR SCARB1 SREBF2 HMGCS1 HMGCR SQLE INSIG1 CYP51A1 NPC1 ACAT2 DHCR24 LIPE SOAT1 SOAT2 ABCA1 ABCG1 BABAM2 ADGRV1 SLC40A1 SOD2 NEAT1 APOE LRRC75A PHKG1 CNN3 SNHG29 GJA1 APOC1 GSTA1 HLA-B CYB5A SULT2A1 AKR1C3 ALPL CHGA CHGB DBH GNAS PCSK1N IGKC IGHG3 IGHG1 JCHAIN IGHA1
e
Number of UMIs per spot
10000 20000
Number of genes per spot
2000 4000 6000
Percent mitochondrial RNA
3 6 9 12

Supplementary Fig. 5 | Visium spatial transcriptomics data of human adult adrenal gland. a-b, Joint clusters were obtained through Seurat integration and clustering of four Visium samples. **a**, Clustering results on tissue slide for each sample. **b**, Clustering results on integrated UMAP embedding (6,984 spots). **c**, Per sample compositional impact on integrated UMAP embedding. **d**, DotPlot displaying cluster-specific marker genes and key steroidogenic enzymes. **e**, Number of UMIs per spot, Number of genes per spot and Percent mitochondrial RNA on tissue slide for each sample.

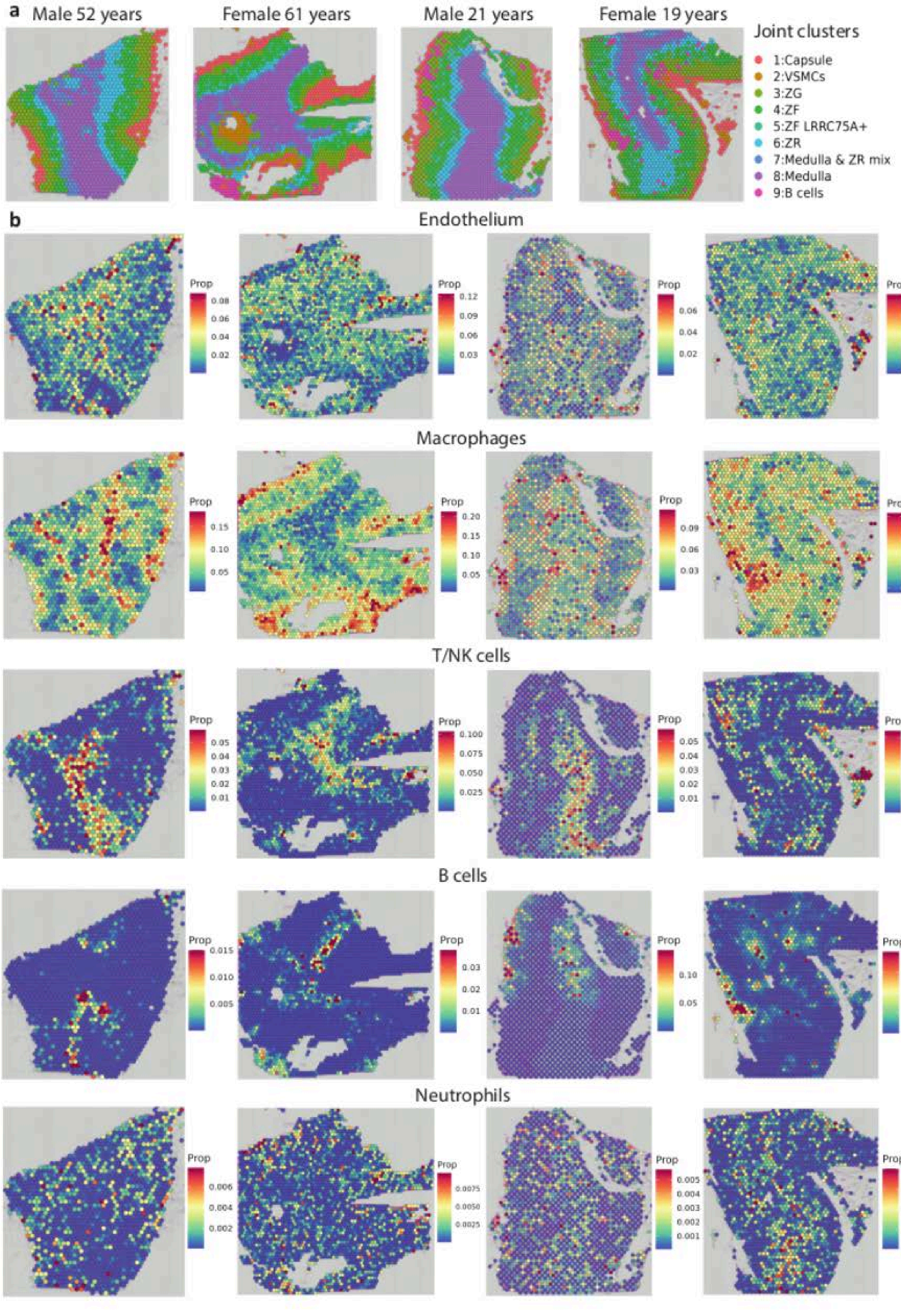

a
Male 52 years
Female 61 years
Male 21 years
Female 19 years
Joint clusters
1:Capsule
2:VSMCs
3:ZG
4:ZF
5:ZF LRRC75A+
6:ZR
7:Medulla & ZR mix
8:Medulla
9:B cells
b
Endothelium
Macrophages
T/NK cells
B cells
Neutrophils
Prop

Supplementary Fig. 6 | Visium cell type deconvolution results for endothelial and immune cell types. **a**, Joint clusters were obtained through Seurat integration and clustering of four Visium samples and are shown on tissue slide for each sample. **b**, The proportion of endothelial and immune (Macrophages, T/NK cells, B cells, Neutrophils) cell types in each spot on tissue slide for each sample, obtained via RCTD cell type deconvolution on the human adult adrenal scRNA-seq reference.

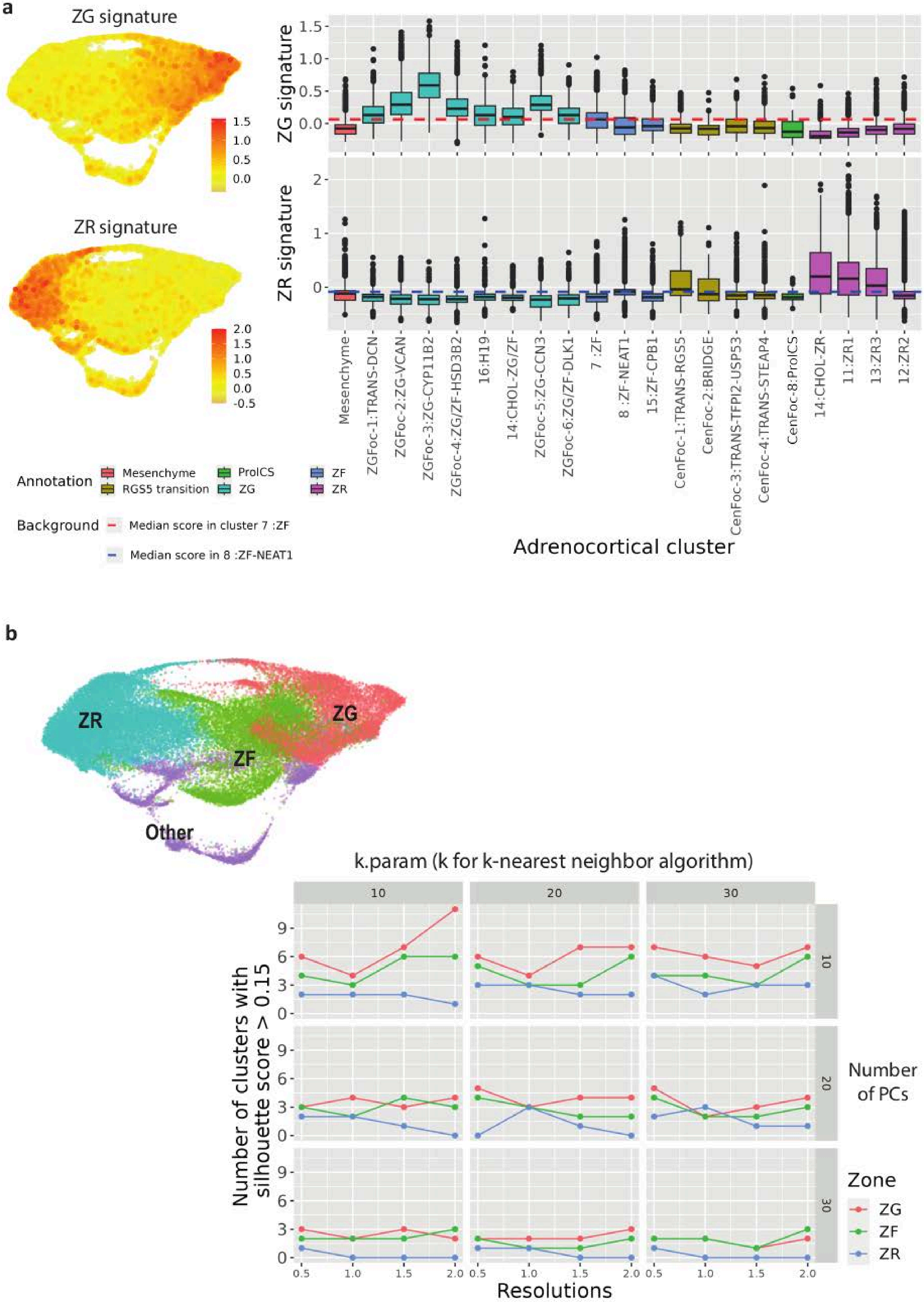

a
ZG signature
ZR signature
ZG signature
ZR signature
Annotation
Mesenchyme
RGS5 transition
ProlCS
ZG
ZF
ZR
Background
Median score in cluster 7 :ZF
Median score in 8 :ZF-NEAT1
Adrenocortical cluster
Mesenchyme
ZGFoc-1:TRANS-DCN
ZGFoc-2:ZG-VCAN
ZGFoc-3:ZG-CYP11B2
ZGFoc-4:ZG/ZF-HSD3B2
16:H19
14:CHOL-ZG/ZF
ZGFoc-5:ZG-CCN3
ZGFoc-6:ZG/ZF-DLK1
7 :ZF
8 :ZF-NEAT1
15:ZF-CPB1
CenFoc-1:TRANS-RGS5
CenFoc-2:BRIDGE
CenFoc-3:TRANS-TFPI2-USP53
CenFoc-4:TRANS-STEAP4
CenFoc-8:ProlCS
14:CHOL-ZR
11:ZR1
13:ZR3
12:ZR2
b
ZR
ZG
ZF
Other
k.param (k for k-nearest neighbor algorithm)
Number of clusters with silhouette score > 0.15
Resolutions
Number of PCs
Zone
ZG
ZF
ZR

Supplementary Fig. 7 | Zona glomerulosa heterogeneity systematic validation. a, Zona glomerulosa (ZG) and zona reticularis (ZR) gene signatures expression distribution on UMAP (39,985 cells, left) and in each cluster (right). The clusters shown include subpopulations derived from focused reintegration/reclustering of the zona glomerulosa (cluster name prefix ZGFoc-), the central part of the common embedding and proliferating cortical cells (cluster name prefix CenFoc-). Mesenchymal cells are grouped in the Mesenchyme cluster. For each box-plot, center line, median; box limits, upper and lower quartiles; whiskers, 1.5x interquartile range; points, outliers. **b**, We confirmed higher heterogeneity of ZG relative to other zones (p.adj < 4.4e-5, two-sided paired Wilcoxon test) by analyzing silhouette scores at different clustering resolutions and in a wide range of parameters. From each zone 10,000 cells were sampled, for each clusterization the mean silhouette score in each cluster was then calculated.

Human adrenocortical focused zona glomerulosa reclustering

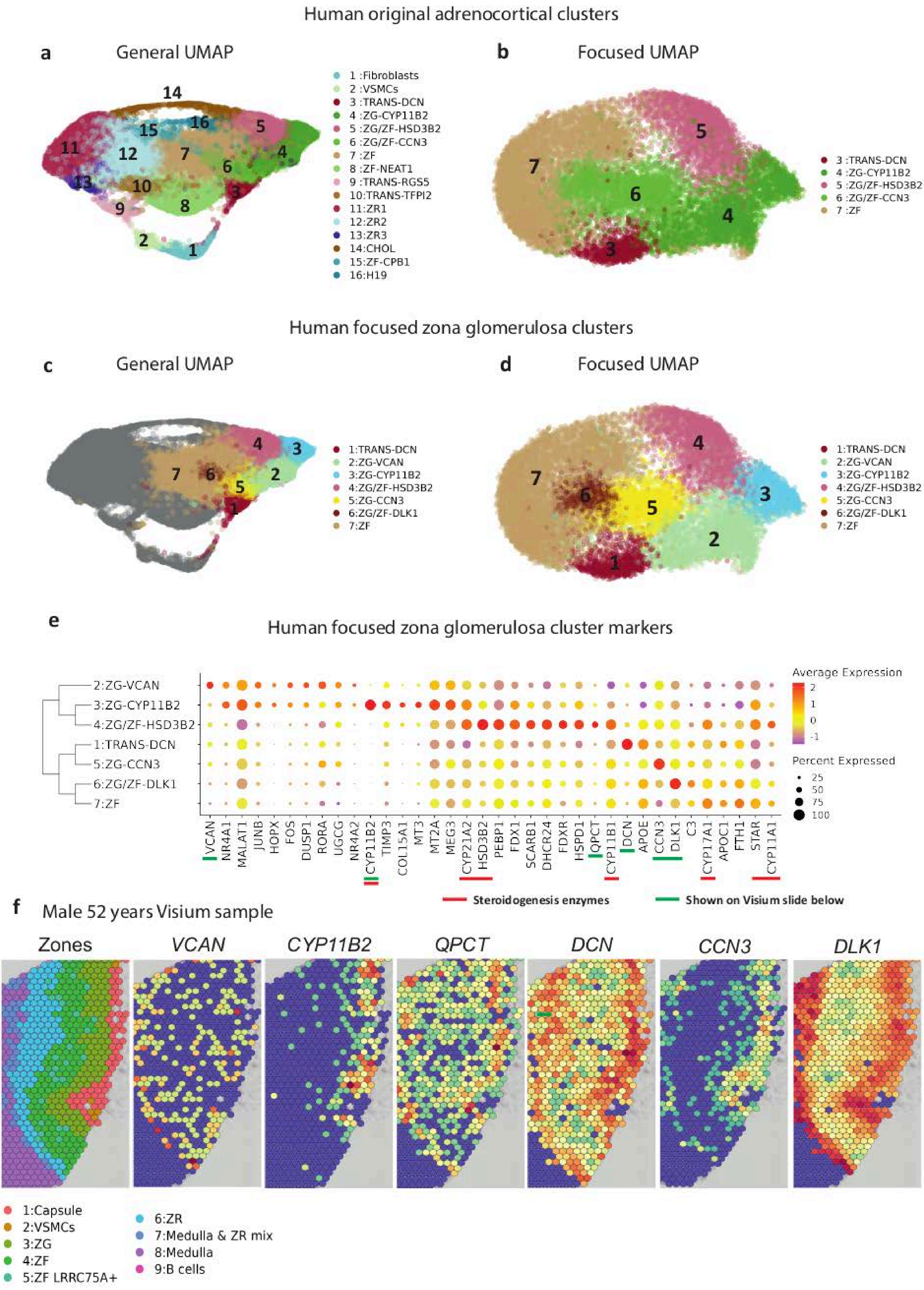

Supplementary Fig. 8 | Focused reclustering of human zona glomerulosa. a-d, Clusters 3-7 of adrenocortical and mesenchymal UMAP were re-analysed separately to increase local embedding and clustering resolution. Clusters 3, 5, 7 from original embedding correspond to clusters 1, 4, 7, respectively, on the new (focused) embedding and have the same colors on both UMAPs. **a**, Original UMAP embedding and original clusterization of adrenocortical and mesenchymal cells (39,985 cells). **b**, Focused UMAP embedding and original clusterization (17,260 cells). **c**, Original UMAP embedding and focused clusterization (39,985 cells). **d**, Focused UMAP embedding and focused clusterization (17,260 cells). **e**, DotPlot displaying focused cluster-specific marker genes and key steroidogenic enzymes. The hierarchy of clusters, based on transcriptional similarity, is shown on the left. **f**, Visualization of key marker gene expression using spatial transcriptomics (Visium).

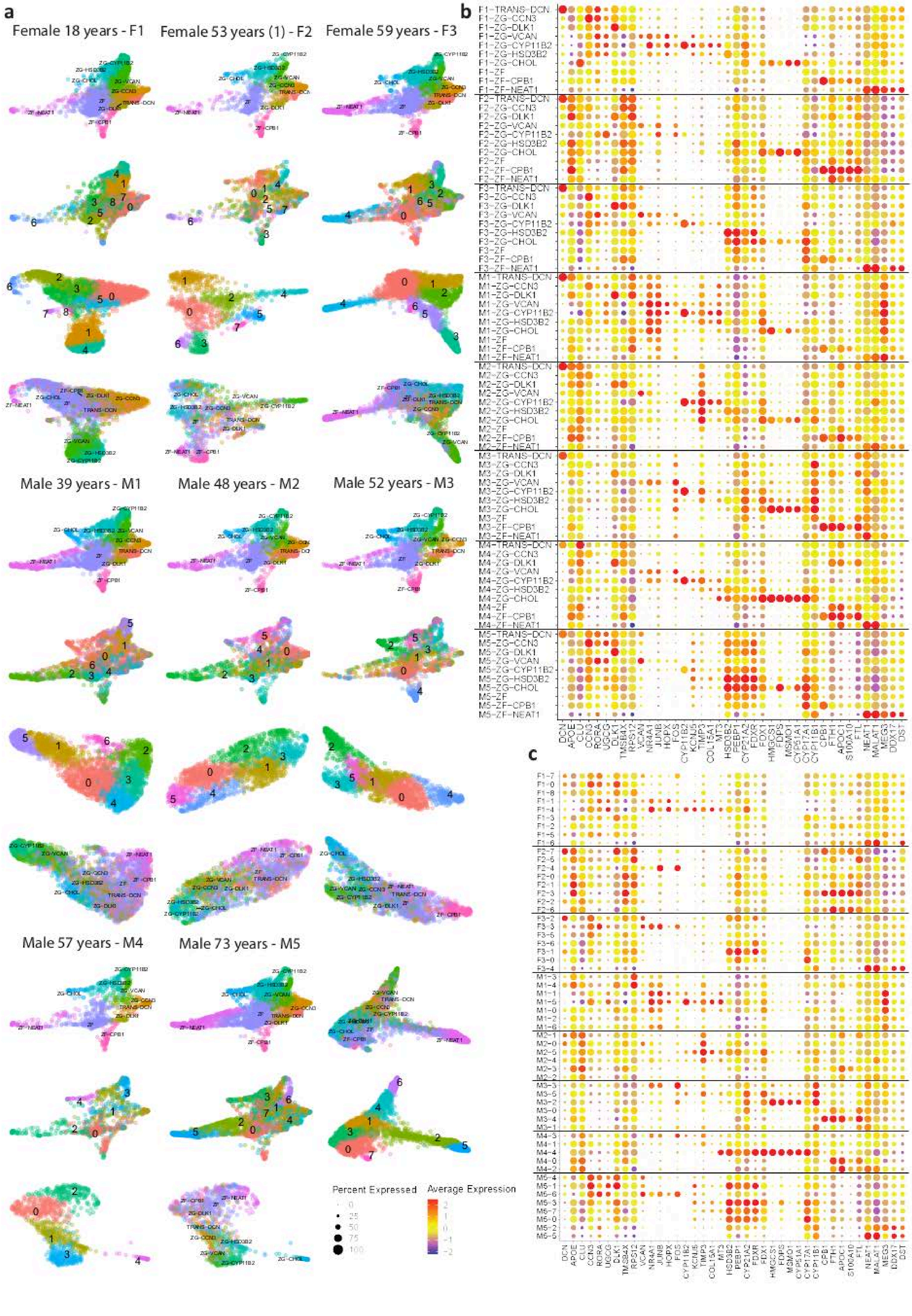

a
Female 18 years - F1
Female 53 years (1) - F2
Female 59 years - F3
Male 39 years - M1
Male 48 years - M2
Male 52 years - M3
Male 57 years - M4
Male 73 years - M5
b
F1-TRANS-DCN
F1-ZG-CCN3
F1-ZG-DLK1
F1-ZG-VCAN
F1-ZG-CYP11B2
F1-ZG-HSD3B2
F1-ZG-CHOL
F1-ZF
F1-ZF-CPB1
F1-ZF-NEAT1
F2-TRANS-DCN
F2-ZG-CCN3
F2-ZG-DLK1
F2-ZG-VCAN
F2-ZG-CYP11B2
F2-ZG-HSD3B2
F2-ZG-CHOL
F2-ZF
F2-ZF-CPB1
F2-ZF-NEAT1
F3-TRANS-DCN
F3-ZG-CCN3
F3-ZG-DLK1
F3-ZG-VCAN
F3-ZG-CYP11B2
F3-ZG-HSD3B2
F3-ZG-CHOL
F3-ZF
F3-ZF-CPB1
F3-ZF-NEAT1
M1-TRANS-DCN
M1-ZG-CCN3
M1-ZG-DLK1
M1-ZG-VCAN
M1-ZG-CYP11B2
M1-ZG-HSD3B2
M1-ZG-CHOL
M1-ZF
M1-ZF-CPB1
M1-ZF-NEAT1
M2-TRANS-DCN
M2-ZG-CCN3
M2-ZG-DLK1
M2-ZG-VCAN
M2-ZG-CYP11B2
M2-ZG-HSD3B2
M2-ZG-CHOL
M2-ZF
M2-ZF-CPB1
M2-ZF-NEAT1
M3-TRANS-DCN
M3-ZG-CCN3
M3-ZG-DLK1
M3-ZG-VCAN
M3-ZG-CYP11B2
M3-ZG-HSD3B2
M3-ZG-CHOL
M3-ZF
M3-ZF-CPB1
M3-ZF-NEAT1
M4-TRANS-DCN
M4-ZG-CCN3
M4-ZG-DLK1
M4-ZG-VCAN
M4-ZG-CYP11B2
M4-ZG-HSD3B2
M4-ZG-CHOL
M4-ZF
M4-ZF-CPB1
M4-ZF-NEAT1
M5-TRANS-DCN
M5-ZG-CCN3
M5-ZG-DLK1
M5-ZG-VCAN
M5-ZG-CYP11B2
M5-ZG-HSD3B2
M5-ZG-CHOL
M5-ZF
M5-ZF-CPB1
M5-ZF-NEAT1
DCN APOE CLU CCN3 RORA UGCG DLK1 TMSB4X RPS12 VCAN NR4A1 JUNB HOPX FOS CYP11B2 KCNJ5 TIMP3 COL15A1 MT3 HSD3B2 PEBP1 CYP21A2 FDXR FDX1 HMGCS1 FDPS MSMO1 CYP51A1 CYP17A1 CYP11B1 CPB1 FTH1 APOC1 S100A10 FTL NEAT1 MALAT1 MEG3 DDX17 DST
c
F1-7 F1-0 F1-8 F1-1 F1-4 F1-3 F1-2 F1-5 F1-6
F2-7 F2-5 F2-4 F2-0 F2-1 F2-3 F2-2 F2-6
F3-2 F3-3 F3-5 F3-6 F3-1 F3-0 F3-4
M1-3 M1-4 M1-1 M1-5 M1-0 M1-2 M1-6
M2-1 M2-0 M2-5 M2-4 M2-3 M2-2
M3-3 M3-5 M3-2 M3-0 M3-4 M3-1
M4-3 M4-1 M4-4 M4-0 M4-2
M5-4 M5-1 M5-6 M5-3 M5-7 M5-0 M5-2 M5-5
DCN APOE CLU CCN3 RORA UGCG DLK1 TMSB4X RPS12 VCAN NR4A1 JUNB HOPX FOS CYP11B2 KCNJ5 TIMP3 COL15A1 MT3 HSD3B2 PEBP1 CYP21A2 FDXR FDX1 HMGCS1 FDPS MSMO1 CYP51A1 CYP17A1 CYP11B1 CPB1 FTH1 APOC1 S100A10 FTL NEAT1 MALAT1 MEG3 DDX17 DST
Percent Expressed
0
25
50
75
100
Average Expression
2
1
0
-1
-2

Supplementary Fig. 9 | Reclustering of human zona glomerulosa and fasciculata cells in individual samples. a, For each sample, which contains at least 800 ZG & ZF cells (8/11 samples in total) four plots are provided. They are subset from joint embedding with joint clusterization, subset from joint embedding with individual sample clusterization, individual sample FA2 embedding with individual sample clusterization, and individual sample FA2 embedding with joint clusterization. Joint embedding and joint clusterization are from Fig. 3a. **b-c**, Dot plots with human adult ZG & ZF marker genes expression in each sample individually. Markers are the same as on Fig. 3b. **b**, For each sample joint clusters are shown. **c**, For each sample individual clusters are shown.

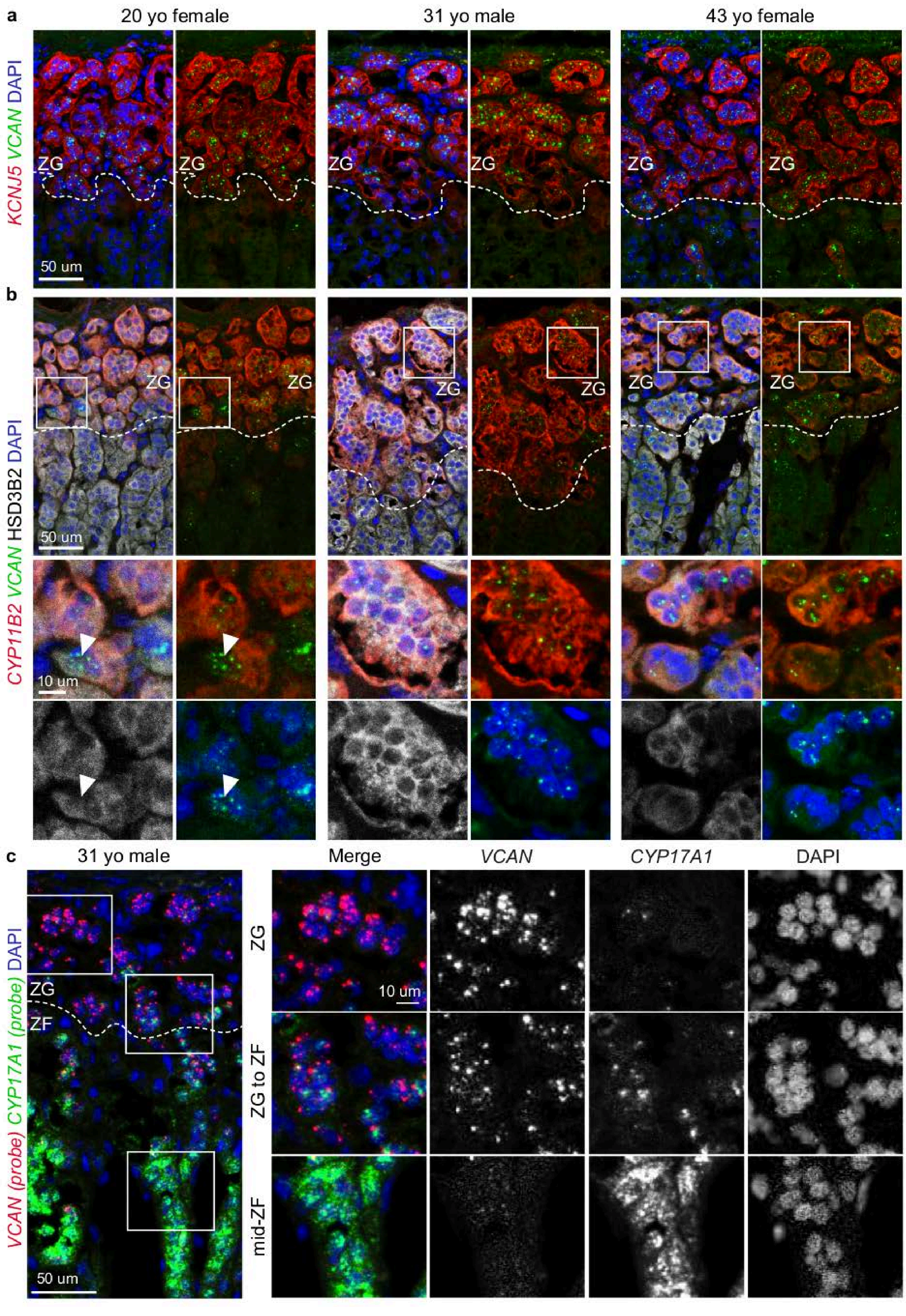

a
20 yo female
31 yo male
43 yo female
KCNJ5 VCAN DAPI
ZG
50 um
b
HSD3B2 DAPI
CYP11B2 VCAN
ZG
50 um
10 um
c
31 yo male
Merge
VCAN
CYP17A1
DAPI
VCAN (probe) CYP17A1 (probe) DAPI
ZG
ZF
ZG to ZF
mid-ZF
10 um
50 um

Supplementary Fig. 10 | Validation of VCAN as a marker gene of zona glomerulosa cells in healthy human adult adrenal cortex. a. RNAscope *in situ* hybridization for *VCAN* (green) combined with immunofluorescence for ZG marker KCNJ5 (red) on adrenal cortex biopsies from 3 donors shows VCAN+ mRNA dots within zona glomerulosa **b**. RNAscope in situ hybridization for *VCAN* (green) combined with immunofluorescence for ZG marker CYP11B2 (red) and ZG/ZF marker HSD3B2 on adrenal cortex biopsies from 3 donors shows variable *VCAN* expression and the occasional VCAN+/CYP11B2- cell (arrow head). **c.** RNAscope in situ hybridization for *VCAN* (red) and *CYP17A1* (green, marker gene of ZF/ZR) on adrenal cortex biopsy from a 31-year-old male reveals high VCAN expression in CYP17A1- zona glomerulosa, much lower in apical and absent in more basal zona fasciculata.

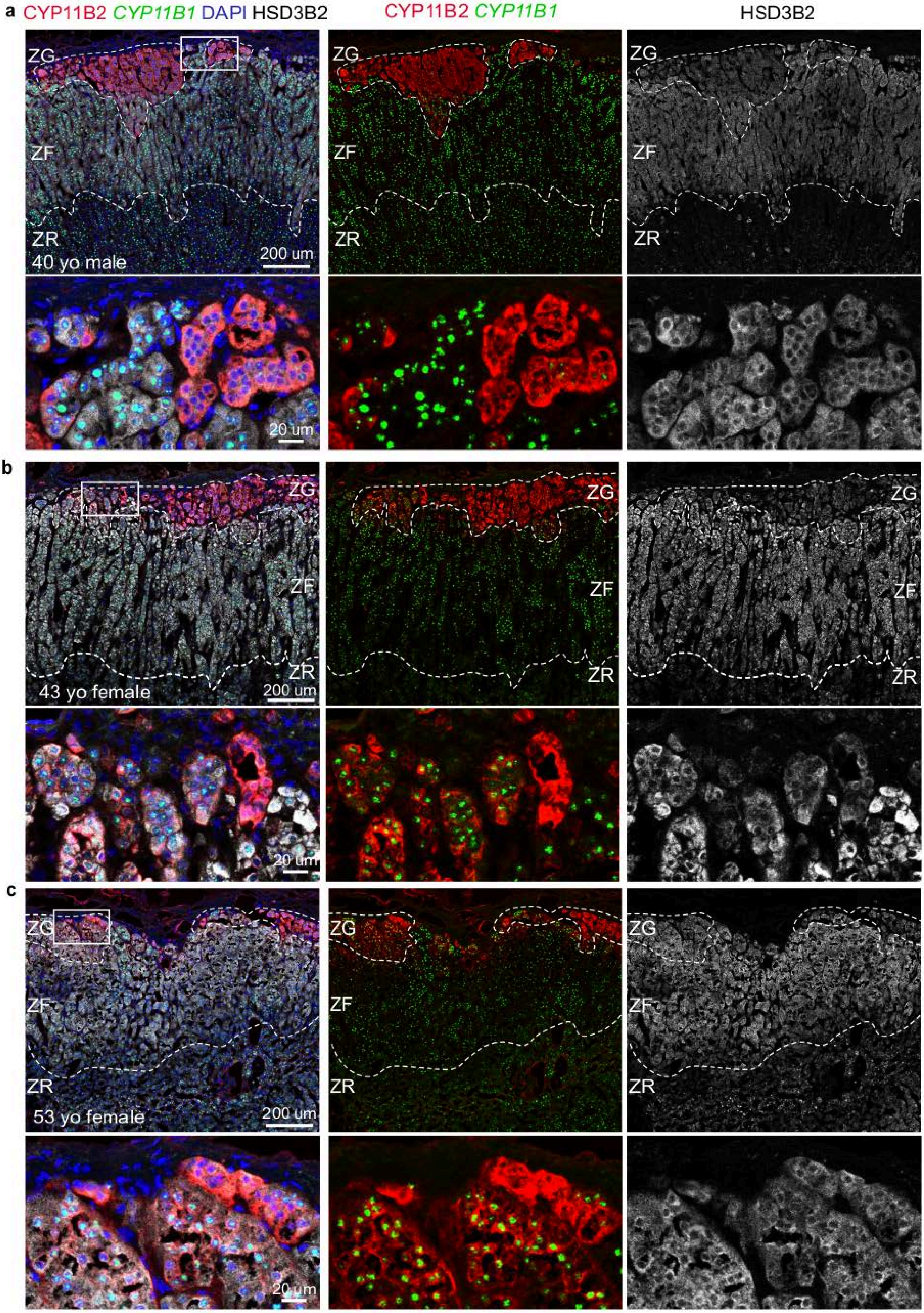

a
CYP11B2 CYP11B1 DAPI HSD3B2
CYP11B2 CYP11B1
HSD3B2
ZG
ZF
ZR
40 yo male
200 um
20 um
b
43 yo female
c
53 yo female

Supplementary Fig. 11 | Increased numbers of HSD3B2+/*CYP11B1*+ cells lacking CYP11B2 expression within aged adult human zona glomerulosa. a-c. RNAscope *in situ* hybridization against *CYP11B1* (green) combined with immunofluorescence against CYP11B2 (red) and HSD3B2 (gray) on adrenal cortex biopsies from 3 donors ages 40 years old and older reveal loss of classical aldosterone-producing CYP11B2+/CYP11B1- cells and onset of CYP11B1 expression in CYP11B2/HSD3B2+ cells within ZG:

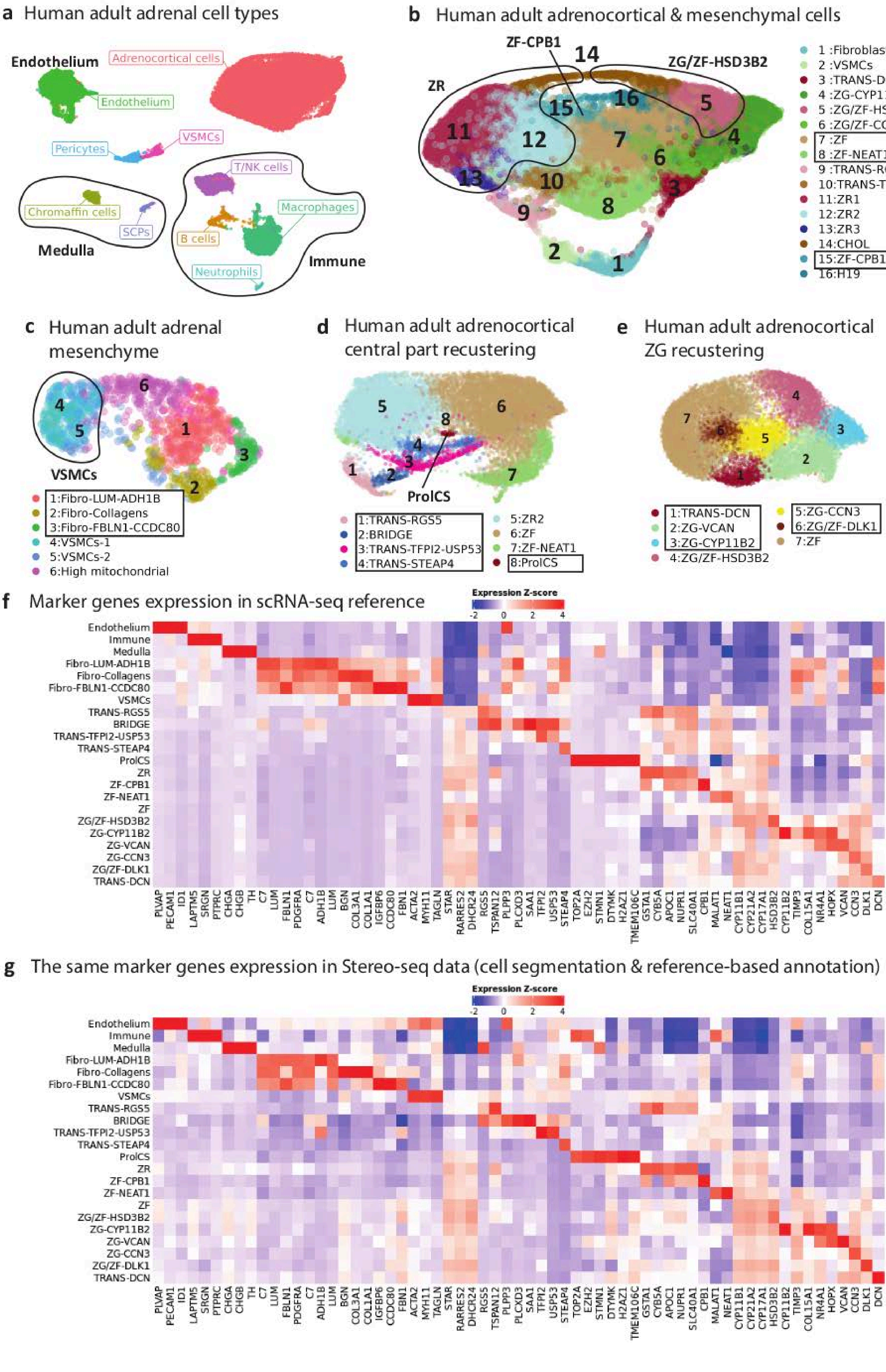

a Human adult adrenal cell types
Endothelium
Adrenocortical cells
Endothelium
Pericytes
VSMCs
T/NK cells
Chromaffin cells
SCPs
Medulla
Macrophages
B cells
Neutrophils
Immune
b Human adult adrenocortical & mesenchymal cells
ZF-CPB1
ZR
ZG/ZF-HSD3B2
1 :Fibroblasts
2 :VSMCs
3 :TRANS-DCN
4 :ZG-CYP11B2
5 :ZG/ZF-HSD3B2
6 :ZG/ZF-CCN3
7 :ZF
8 :ZF-NEAT1
9 :TRANS-RGS5
10:TRANS-TFPI2
11:ZR1
12:ZR2
13:ZR3
14:CHOL
15:ZF-CPB1
16:H19
c Human adult adrenal mesenchyme
VSMCs
1:Fibro-LUM-ADH1B
2:Fibro-Collagens
3:Fibro-FBLN1-CCDC80
4:VSMCs-1
5:VSMCs-2
6:High mitochondrial
d Human adult adrenocortical central part recustering
ProlCS
1:TRANS-RGS5
2:BRIDGE
3:TRANS-TFPI2-USP53
4:TRANS-STEAP4
5:ZR2
6:ZF
7:ZF-NEAT1
8:ProlCS
e Human adult adrenocortical ZG recustering
1:TRANS-DCN
2:ZG-VCAN
3:ZG-CYP11B2
4:ZG/ZF-HSD3B2
5:ZG-CCN3
6:ZG/ZF-DLK1
7:ZF
f Marker genes expression in scRNA-seq reference
Expression Z-score
-2 0 2 4
Endothelium
Immune
Medulla
Fibro-LUM-ADH1B
Fibro-Collagens
Fibro-FBLN1-CCDC80
VSMCs
TRANS-RGS5
BRIDGE
TRANS-TFPI2-USP53
TRANS-STEAP4
ProlCS
ZR
ZF-CPB1
ZF-NEAT1
ZF
ZG/ZF-HSD3B2
ZG-CYP11B2
ZG-VCAN
ZG-CCN3
ZG/ZF-DLK1
TRANS-DCN
PLVAP PECAM1 ID1 LAPTM5 SRGN PTPRC CHGA CHGB TH C7 LUM FBLN1 PDGFRA C7 ADH1B LUM BGN COL3A1 COL1A1 IGFBP6 CCDC80 FBN1 ACTA2 MYH11 TAGLN STAR RARRES2 DHCR24 RGS5 TSPAN12 PLPP3 PLCXD3 SAA1 TFPI2 USP53 STEAP4 TOP2A EZH2 STMN1 DTYMK H2AZ1 TMEM106C GSTA1 CYB5A APOC1 NUPR1 SLC40A1 CPB1 MALAT1 NEAT1 CYP11B1 CYP21A2 CYP17A1 HSD3B2 CYP11B2 TIMP3 COL15A1 NR4A1 HOPX VCAN CCN3 DLK1 DCN
g The same marker genes expression in Stereo-seq data (cell segmentation & reference-based annotation)
Expression Z-score
-2 0 2 4
Endothelium
Immune
Medulla
Fibro-LUM-ADH1B
Fibro-Collagens
Fibro-FBLN1-CCDC80
VSMCs
TRANS-RGS5
BRIDGE
TRANS-TFPI2-USP53
TRANS-STEAP4
ProlCS
ZR
ZF-CPB1
ZF-NEAT1
ZF
ZG/ZF-HSD3B2
ZG-CYP11B2
ZG-VCAN
ZG-CCN3
ZG/ZF-DLK1
TRANS-DCN
PLVAP PECAM1 ID1 LAPTM5 SRGN PTPRC CHGA CHGB TH C7 LUM FBLN1 PDGFRA C7 ADH1B LUM BGN COL3A1 COL1A1 IGFBP6 CCDC80 FBN1 ACTA2 MYH11 TAGLN STAR RARRES2 DHCR24 RGS5 TSPAN12 PLPP3 PLCXD3 SAA1 TFPI2 USP53 STEAP4 TOP2A EZH2 STMN1 DTYMK H2AZ1 TMEM106C GSTA1 CYB5A APOC1 NUPR1 SLC40A1 CPB1 MALAT1 NEAT1 CYP11B1 CYP21A2 CYP17A1 HSD3B2 CYP11B2 TIMP3 COL15A1 NR4A1 HOPX VCAN CCN3 DLK1 DCN

Supplementary Fig. 12 | Cell type annotation of human high-resolution spatial transcriptomics (Stereo-seq). a-e, Reference scRNA-seq UMAP embeddings with focused view on different aspects of adrenal cell type heterogeneity. On each UMAP or its cluster legend, highlighted cell states were included in reference and resolved in spatial data. **a,** The UMAP embedding of the human adult adrenal gland cells (60,146 cells), color coded by cell type. Medullary, immune or endothelial cells were annotated as single groups. **b,** The UMAP embedding resulting from integration of adrenocortical and mesenchymal cells, including fibroblasts and vascular smooth muscle cells (39,985 cells). Since cluster 14:CHOL consists of ZG and ZR cells with increased cholesterol biosynthesis signature expression, it was splitted into two parts. Because cholesterol biosynthesis also occurs in other ZG/ZR cells in a gradient manner, the left and right parts of the 14:CHOL cluster were joined with ZR and ZG/ZF-HSD3B2 respectively. **c,** The UMAP embedding resulting from the focused reintegration of human adult adrenal mesenchymal cells (1,177 cells). VSMCs subclusters don't have clear discriminative marker genes and were joined. **d,** The UMAP embedding with the focused central part of the common embedding and proliferating cortical cells (16,926 cells). **e,** The UMAP embedding with focused reclustering of ZG uncovering its cell state heterogeneity (17,260 cells). **f-g,** Heatmaps representing the expression of marker genes in all cell states, identified using scRNA-seq reference data. Markers with high expression levels were manually selected based on differential expression analysis on scRNA-seq reference data. **f,** Expression in scRNA-seq reference data. **g,** Expression in spatial transcriptomics Stereo-seq data (in segmented and annotated cells). ProlCS, proliferating cortical state; ZG, zona glomerulosa; ZF, zona fasciculata; ZR, zona reticularis.

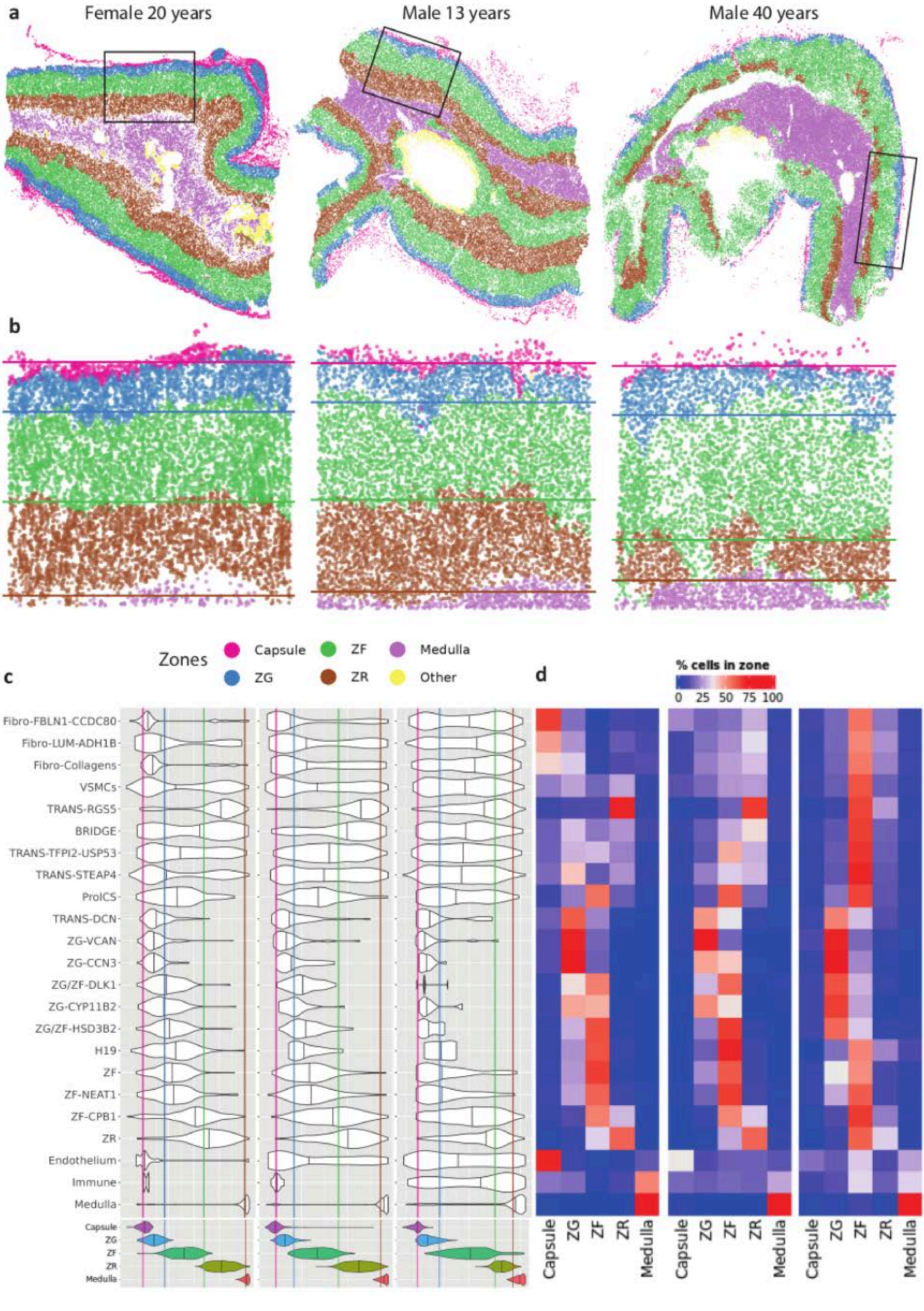

a
Female 20 years
Male 13 years
Male 40 years
b
Zones
Capsule
ZG
ZF
ZR
Medulla
Other
c
Fibro-FBLN1-CCDC80
Fibro-LUM-ADH1B
Fibro-Collagens
VSMCs
TRANS-RGS5
BRIDGE
TRANS-TFPI2-USP53
TRANS-STEAP4
ProlCS
TRANS-DCN
ZG-VCAN
ZG-CCN3
ZG/ZF-DLK1
ZG-CYP11B2
ZG/ZF-HSD3B2
H19
ZF
ZF-NEAT1
ZF-CPB1
ZR
Endothelium
Immune
Medulla
d
% cells in zone
0 25 50 75 100
Capsule
ZG
ZF
ZR
Medulla

Supplementary Fig. 13 | Adrenal zones and cell types spatial distribution across zones in human Stereo-seq data. a-b, Key adrenal zones on tissue sections **a**, Zones on whole tissue sections (85,276, 64,499 and 75,402 cells for Female 20, Male 13 and Male 40 samples, respectively). Selected areas with intact and representative layered structure are highlighted with rectangles. The cluster named Other mainly corresponds to large vessels or to poorly covered areas. **b**, Zones on selected section areas (8,420, 7,076 and 5,536 cells for Female 20, Male 13 and Male 40 samples, respectively). Those selected areas are used as representatives for visualization of cell states in tissue in following figures. **c**, Violin plots representing spatial distribution of various cell groups (above are cell types, below - adrenal zones) in the selected representative areas. Vertical lines depict boundaries between adrenal zones. One column per sample: Female 20 years, Male 13 years, Male 30 years. **b**, Heatmaps (one column per sample) representing how each cell type is split by zones in the whole adrenal section (i.e. which percent of cells of ZG-VCAN cell type belong to ZG). ZG, zona glomerulosa; ZF, zona fasciculata; ZR, zona reticularis.

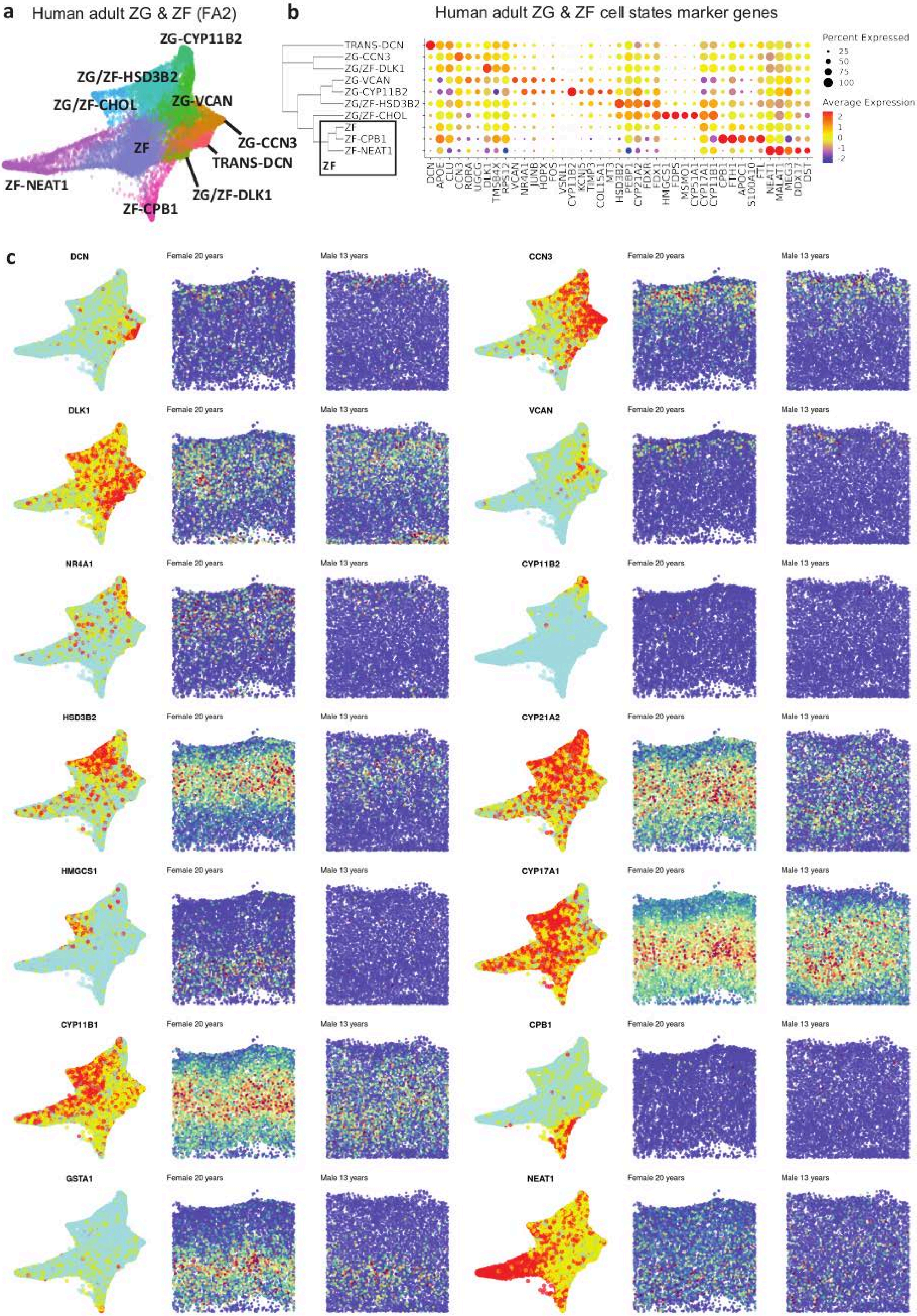
a
Human adult ZG & ZF (FA2)
ZG-CYP11B2
ZG/ZF-HSD3B2
ZG/ZF-CHOL
ZG-VCAN
ZF
ZG-CCN3
TRANS-DCN
ZF-NEAT1
ZG/ZF-DLK1
ZF-CPB1
b
Human adult ZG & ZF cell states marker genes
TRANS-DCN
ZG-CCN3
ZG/ZF-DLK1
ZG-VCAN
ZG-CYP11B2
ZG/ZF-HSD3B2
ZG/ZF-CHOL
ZF
ZF-CPB1
ZF-NEAT1
ZF
Percent Expressed
25
50
75
100
Average Expression
2
1
0
-1
-2
DCN
APOE
CLU
CCN3
RORA
UGCG
DLK1
TMSB4X
RPS12
VCAN
NR4A1
JUNB
HOPX
FOS
VSNL1
CYP11B2
KCNJ5
TIMP3
COL15A1
MT3
HSD3B2
PEBP1
CYP21A2
FDXR
FDX1
HMGCS1
FDPS
MSMO1
CYP51A1
CYP17A1
CYP11B1
CPB1
FTH1
APOC1
S100A10
FTL
NEAT1
MALAT1
MEG3
DDX17
DST
c
DCN
CCN3
DLK1
VCAN
NR4A1
CYP11B2
HSD3B2
CYP21A2
HMGCS1
CYP17A1
CYP11B1
CPB1
GSTA1
NEAT1
Female 20 years
Male 13 years

Supplementary Fig. 14 | Marker genes driving human adult ZG and ZF transcriptomic heterogeneity. a, A ForceAtlas2 (FA2) embedding illustrating zona glomerulosa (ZG) & zona fasciculata (ZF) (20,255 cells), with clusters derived from focused reclustering of ZG. **b,** DotPlot displaying cluster-specific marker genes. The hierarchy of clusters, based on transcriptional similarity, is shown on the left. **c**, The expression of key marker genes from **b** in scRNA-seq data and high-resolution spatial transcriptomics (Stereo-seq) of the two normal human adult adrenal samples. Each sample is represented by a selected rectangular area exhibiting a representative and undisturbed zonal structure (8,420 and 7,076 cells for Female 20 and Male 13 samples, respectively). Each dot corresponds to a segmented cell, with colors indicating gene expression level.

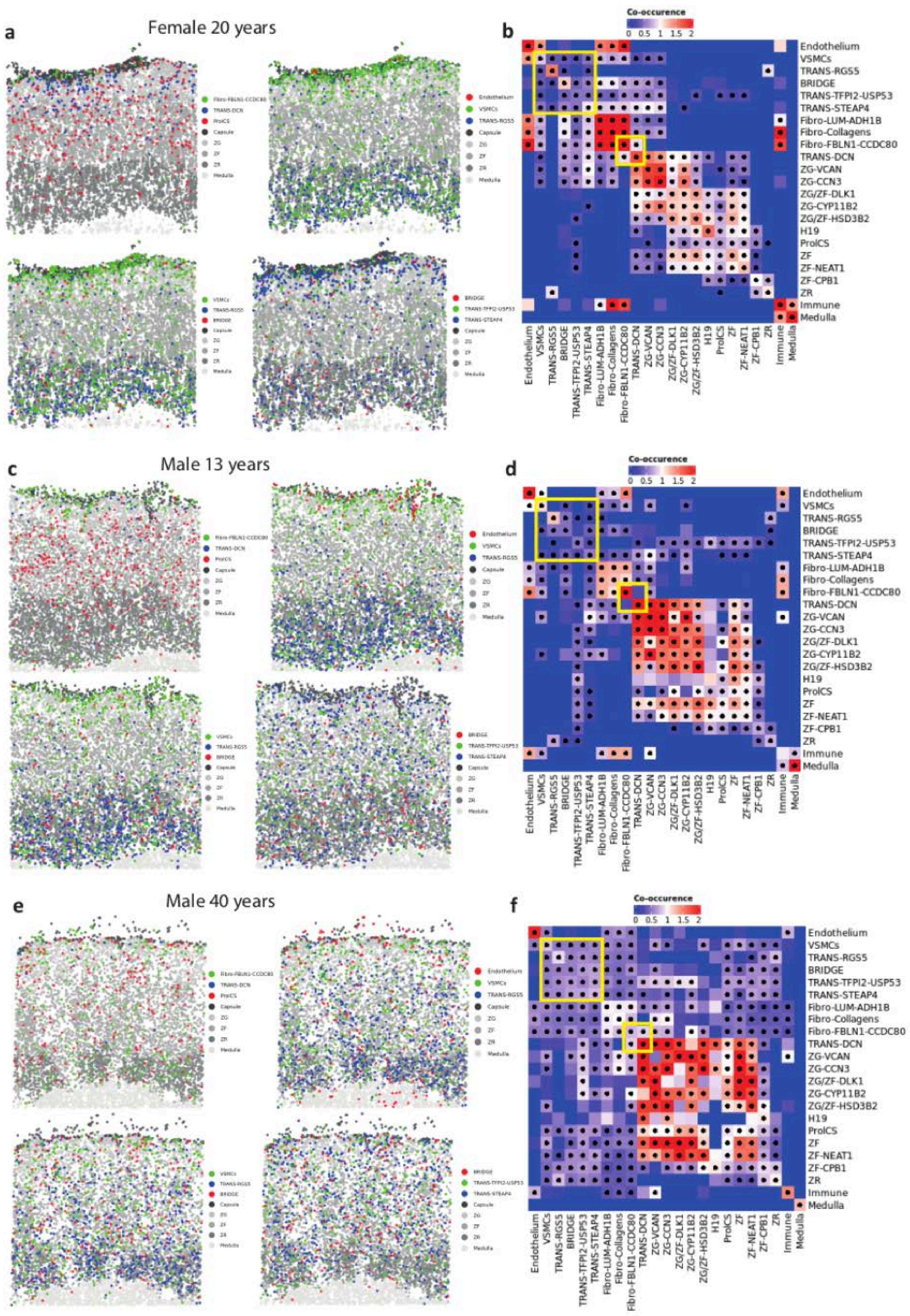

a
Female 20 years
Fibro-FBLN1-CCDC80
TRANS-DCN
ProlCS
Capsule
ZG
ZF
ZR
Medulla
Endothelium
VSMCs
TRANS-RGS5
BRIDGE
TRANS-TFPI2-USP53
TRANS-STEAP4
b
Co-occurence
0 0.5 1 1.5 2
Endothelium
VSMCs
TRANS-RGS5
BRIDGE
TRANS-TFPI2-USP53
TRANS-STEAP4
Fibro-LUM-ADH1B
Fibro-Collagens
Fibro-FBLN1-CCDC80
TRANS-DCN
ZG-VCAN
ZG-CCN3
ZG/ZF-DLK1
ZG-CYP11B2
ZG/ZF-HSD3B2
H19
ProlCS
ZF
ZF-NEAT1
ZF-CPB1
ZR
Immune
Medulla
c
Male 13 years
d
Co-occurence
0 0.5 1 1.5 2
e
Male 40 years
f
Co-occurence
0 0.5 1 1.5 2

Supplementary Fig. 15 | Spatial distribution and co-occurrence of renewal-associated cell types or states in human Stereo-seq data. **a**,**c**,**e**, Renewal-associated cell types (states) were identified in high-resolution spatial transcriptomics (Stereo-seq) data. Each of four subplots represent one of putative transitions (discovered in scRNA-seq dataset). On the first subplot, from Fibro-FBLN1-CCDC80 (green) to TRANS-DCN (blue), ProlCS (red) cells are also shown as possibly also connected with capsular mesenchymal stem cells. On the second subplot, from VSMCs (green) to TRANS-RGS5 (blue), Endothelial (red) cells are also shown as a putative part of the niche. On the third subplot, from TRANS-RGS5 (blue) to BRIDGE (red), VSMCs (green) cells are also shown as a previous step of complex transition. On the fourth subplot, from BRIDGE (red) to TRANS-TFPI2-USP53 (green) or TRANS-STEAP4 (blue). Background gray colors highlight arenal zones. **b**,**d**,**f**, Spatial co-occurrence of cell states in the whole adrenal section. Dots highlight statistically significant enrichment of co-occurrence, adjusted p-value < 0.05 (see Methods section). Yellow squares highlight co-occurrences of states putatively involved in transition. **a-b**, Female 20 years sample (8,420 and 85,276 cells). **c-d**, Male 13 years sample (7,076 and 64,499 cells). **e-f**, Male 40 years sample (5,536 and 75,402 cells). ProlCS, proliferating cortical state; ZG, zona glomerulosa; ZF, zona fasciculata; ZR, zona reticularis.

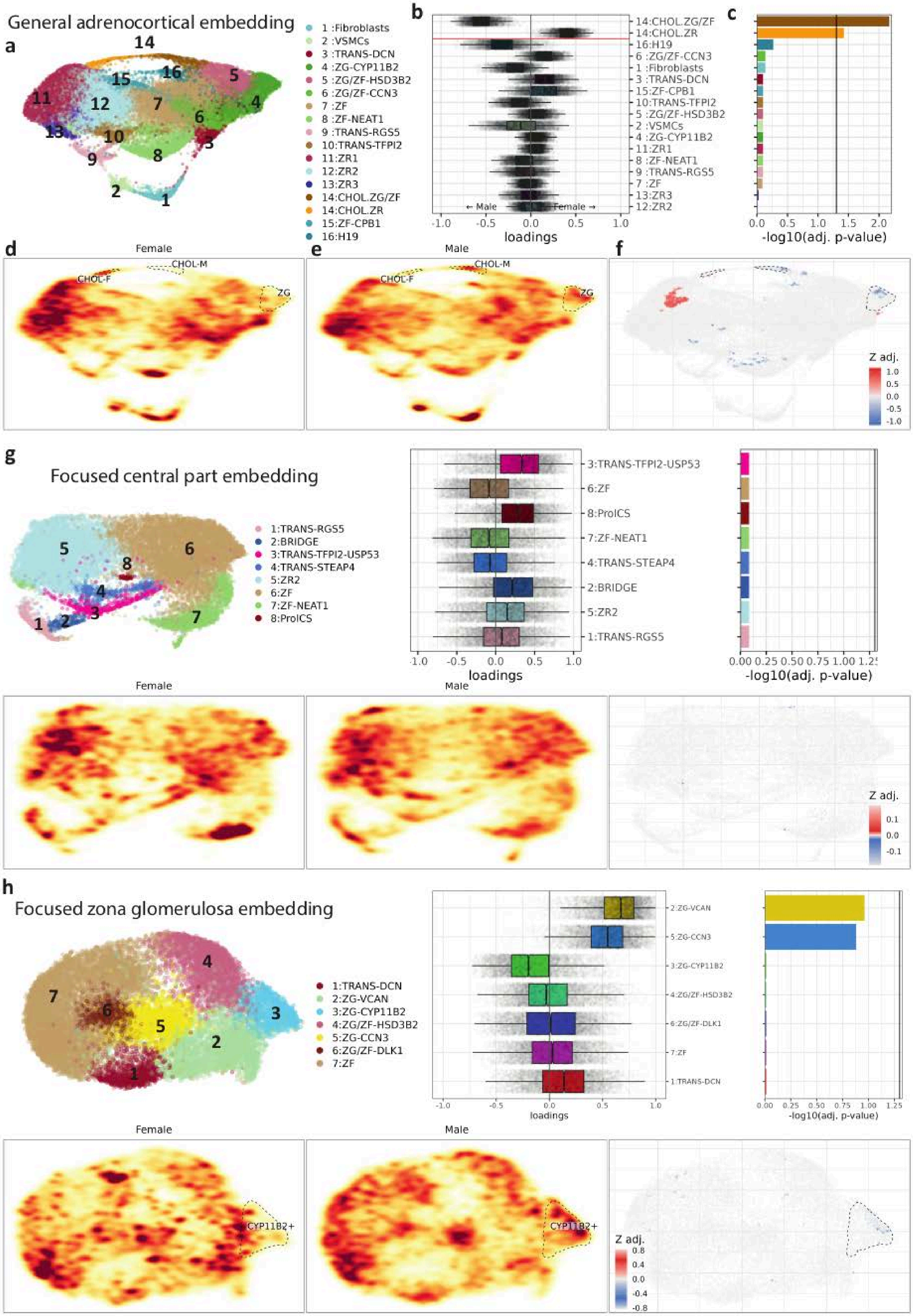
General adrenocortical embedding
a
b
c
1 :Fibroblasts
2 :VSMCs
3 :TRANS-DCN
4 :ZG-CYP11B2
5 :ZG/ZF-HSD3B2
6 :ZG/ZF-CCN3
7 :ZF
8 :ZF-NEAT1
9 :TRANS-RGS5
10:TRANS-TFPI2
11:ZR1
12:ZR2
13:ZR3
14:CHOL.ZG/ZF
14:CHOL.ZR
15:ZF-CPB1
16:H19
14:CHOL.ZG/ZF
14:CHOL.ZR
16:H19
6 :ZG/ZF-CCN3
1 :Fibroblasts
3 :TRANS-DCN
15:ZF-CPB1
10:TRANS-TFPI2
5 :ZG/ZF-HSD3B2
2 :VSMCs
4 :ZG-CYP11B2
11:ZR1
8 :ZF-NEAT1
9 :TRANS-RGS5
7 :ZF
13:ZR3
12:ZR2
← Male
Female →
loadings
-log10(adj. p-value)
d
e
f
Female
Male
CHOL-F
CHOL-M
ZG
Z adj.
g
Focused central part embedding
1:TRANS-RGS5
2:BRIDGE
3:TRANS-TFPI2-USP53
4:TRANS-STEAP4
5:ZR2
6:ZF
7:ZF-NEAT1
8:ProlCS
3:TRANS-TFPI2-USP53
6:ZF
8:ProlCS
7:ZF-NEAT1
4:TRANS-STEAP4
2:BRIDGE
5:ZR2
1:TRANS-RGS5
loadings
-log10(adj. p-value)
Female
Male
Z adj.
h
Focused zona glomerulosa embedding
1:TRANS-DCN
2:ZG-VCAN
3:ZG-CYP11B2
4:ZG/ZF-HSD3B2
5:ZG-CCN3
6:ZG/ZF-DLK1
7:ZF
2:ZG-VCAN
5:ZG-CCN3
3:ZG-CYP11B2
4:ZG/ZF-HSD3B2
6:ZG/ZF-DLK1
7:ZF
1:TRANS-DCN
loadings
-log10(adj. p-value)
Female
Male
CYP11B2+
Z adj.

Supplementary Fig. 16 | Sexual dimorphism of human adult adrenal cortex in terms of cell subtype representation - compostonal effects. a-f, Compositional differences related to the original adrenocortical and mesenchymal cell embedding and clusterization (all results were obtained using cacoa R package, see Methods section). **a**, General (original) UMAP embedding and clusterization (39,985 cells). **b-c**, Cluster based analysis of compositional differences. **b**, Estimated loadings for cluster representation differences. Each dot corresponds to the permutation iteration (n.boot=5000). Positive loading values indicate cluster overrepresentation in females, negative - in males. For each box-plot, center line, median; box limits, upper and lower quartiles; whiskers, 1.5x interquartile range. **c**, Statistical significance of observed cluster representation differences (adjusted with Benjamini-Hochberg method p-values). The vertical line is set at the significance level p-value = 0.05. **d-f**, Cluster-free, embedding cell density based analysis of compositional differences. Dotted lines highlight some areas of significant compositional differences. CHOL-F and CHOL-M are the left (zona reticularis) and right (zona glomerulosa) parts of 14:CHOL cluster with increased density in females and males respectively. ZG are CYP11B2+ zona glomerulosa cells. **d**, Cell density in females. **e**, Cell density in males. **f**, Wilcox test estimation of difference significance in cell density. Red indicates areas of increased density in females, blue - in males. **g**, Compositional differences related to the embedding and clusterization focused on the central part (16,926 cells). **h**, Compositional differences related to the embedding and clusterization focused on zona glomerulosa (17,260 cells).

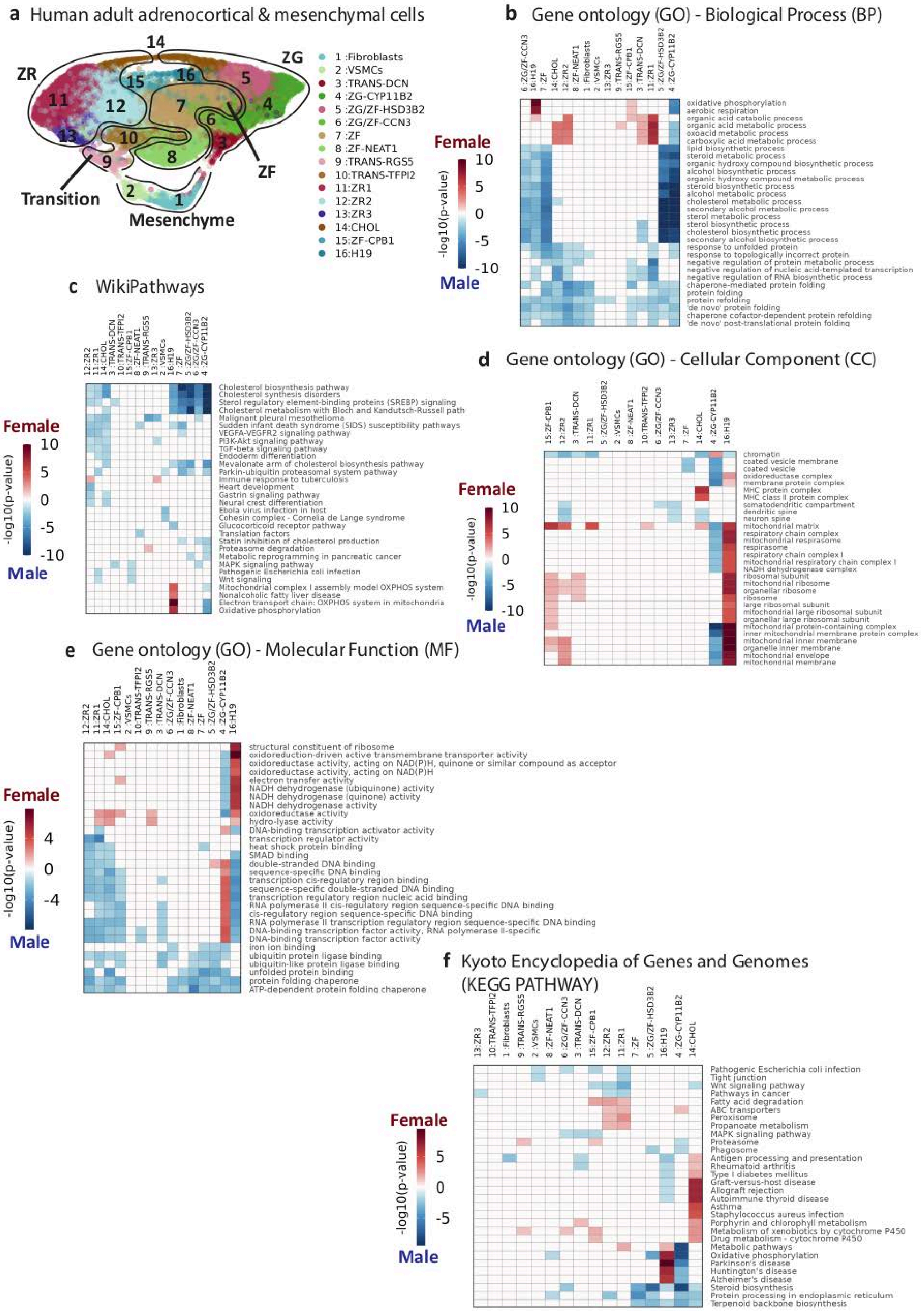
a Human adult adrenocortical & mesenchymal cells
ZR
14
ZG
15
16
5
11
12
7
4
6
13
10
8
3
9
ZF
2
1
Transition
Mesenchyme
1 :Fibroblasts
2 :VSMCs
3 :TRANS-DCN
4 :ZG-CYP11B2
5 :ZG/ZF-HSD3B2
6 :ZG/ZF-CCN3
7 :ZF
8 :ZF-NEAT1
9 :TRANS-RGS5
10:TRANS-TFPI2
11:ZR1
12:ZR2
13:ZR3
14:CHOL
15:ZF-CPB1
16:H19
b Gene ontology (GO) - Biological Process (BP)
Female
Male
-log10(p-value)
oxidative phosphorylation
aerobic respiration
organic acid catabolic process
organic acid metabolic process
oxoacid metabolic process
carboxylic acid metabolic process
lipid biosynthetic process
steroid metabolic process
organic hydroxy compound biosynthetic process
alcohol biosynthetic process
organic hydroxy compound metabolic process
steroid biosynthetic process
alcohol metabolic process
cholesterol metabolic process
secondary alcohol metabolic process
sterol metabolic process
sterol biosynthetic process
cholesterol biosynthetic process
secondary alcohol biosynthetic process
response to unfolded protein
response to topologically incorrect protein
negative regulation of protein metabolic process
negative regulation of nucleic acid-templated transcription
negative regulation of RNA biosynthetic process
chaperone-mediated protein folding
protein folding
protein refolding
'de novo' protein folding
chaperone cofactor-dependent protein refolding
'de novo' post-translational protein folding
c WikiPathways
Cholesterol biosynthesis pathway
Cholesterol synthesis disorders
Sterol regulatory element-binding proteins (SREBP) signaling
Cholesterol metabolism with Bloch and Kandutsch-Russell path
Malignant pleural mesothelioma
Sudden infant death syndrome (SIDS) susceptibility pathways
VEGFA-VEGFR2 signaling pathway
PI3K-Akt signaling pathway
TGF-beta signaling pathway
Endoderm differentiation
Mevalonate arm of cholesterol biosynthesis pathway
Parkin-ubiquitin proteasomal system pathway
Immune response to tuberculosis
Heart development
Gastrin signaling pathway
Neural crest differentiation
Ebola virus infection in host
Cohesin complex - Cornelia de Lange syndrome
Glucocorticoid receptor pathway
Translation factors
Statin inhibition of cholesterol production
Proteasome degradation
Metabolic reprogramming in pancreatic cancer
MAPK signaling pathway
Pathogenic Escherichia coli infection
Wnt signaling
Mitochondrial complex I assembly model OXPHOS system
Nonalcoholic fatty liver disease
Electron transport chain: OXPHOS system in mitochondria
Oxidative phosphorylation
d Gene ontology (GO) - Cellular Component (CC)
chromatin
coated vesicle membrane
coated vesicle
oxidoreductase complex
membrane protein complex
MHC protein complex
MHC class II protein complex
somatodendritic compartment
dendritic spine
neuron spine
mitochondrial matrix
respiratory chain complex
mitochondrial respirasome
respirasome
respiratory chain complex I
mitochondrial respiratory chain complex I
NADH dehydrogenase complex
ribosomal subunit
mitochondrial ribosome
organellar ribosome
ribosome
large ribosomal subunit
mitochondrial large ribosomal subunit
organellar large ribosomal subunit
mitochondrial protein-containing complex
inner mitochondrial membrane protein complex
mitochondrial inner membrane
organelle inner membrane
mitochondrial envelope
mitochondrial membrane
e Gene ontology (GO) - Molecular Function (MF)
structural constituent of ribosome
oxidoreduction-driven active transmembrane transporter activity
oxidoreductase activity, acting on NAD(P)H
oxidoreductase activity, acting on NAD(P)H, quinone or similar compound as acceptor
electron transfer activity
NADH dehydrogenase (ubiquinone) activity
NADH dehydrogenase (quinone) activity
NADH dehydrogenase activity
oxidoreductase activity
hydro-lyase activity
DNA-binding transcription activator activity
transcription regulator activity
heat shock protein binding
SMAD binding
double-stranded DNA binding
sequence-specific DNA binding
transcription cis-regulatory region binding
sequence-specific double-stranded DNA binding
transcription regulatory region nucleic acid binding
RNA polymerase II cis-regulatory region sequence-specific DNA binding
cis-regulatory region sequence-specific DNA binding
RNA polymerase II transcription regulatory region sequence-specific DNA binding
DNA-binding transcription factor activity, RNA polymerase II-specific
DNA-binding transcription factor activity
iron ion binding
ubiquitin protein ligase binding
ubiquitin-like protein ligase binding
unfolded protein binding
protein folding chaperone
ATP-dependent protein folding chaperone
f Kyoto Encyclopedia of Genes and Genomes (KEGG PATHWAY)
Pathogenic Escherichia coli infection
Tight junction
Wnt signaling pathway
Pathways in cancer
Fatty acid degradation
ABC transporters
Peroxisome
Propanoate metabolism
MAPK signaling pathway
Proteasome
Phagosome
Antigen processing and presentation
Rheumatoid arthritis
Type I diabetes mellitus
Graft-versus-host disease
Allograft rejection
Autoimmune thyroid disease
Asthma
Staphylococcus aureus infection
Porphyrin and chlorophyll metabolism
Metabolism of xenobiotics by cytochrome P450
Drug metabolism - cytochrome P450
Metabolic pathways
Oxidative phosphorylation
Parkinson's disease
Huntington's disease
Alzheimer's disease
Steroid biosynthesis
Protein processing in endoplasmic reticulum
Terpenoid backbone biosynthesis

Supplementary Fig. 17 | Sexual dimorphism of human adult adrenal cortex in gene differential expression and transcriptional effects. **a**, Human adult adrenocortical and mesenchymal cells embedding (39,985 cells). **b-f,** Gene set enrichment analysis of differential expression between males and females in each cluster. Columns indicate clusters, rows - pathway names. Colors indicate statistical significance, red - overexpressed in females, blue - in males. **b**, Gene ontology (GO), Biological Process (BP). **c**, WikiPathways. **d**, Gene ontology (GO), Cellular Component (CC). **e**, Gene ontology (GO), Molecular Function (MF). **f**, Kyoto Encyclopedia of Genes and Genomes (KEGG PATHWAY).

a WikiPathways: Cholesterol metabolism with Bloch and Kandush-Russell pathways (WP4718)

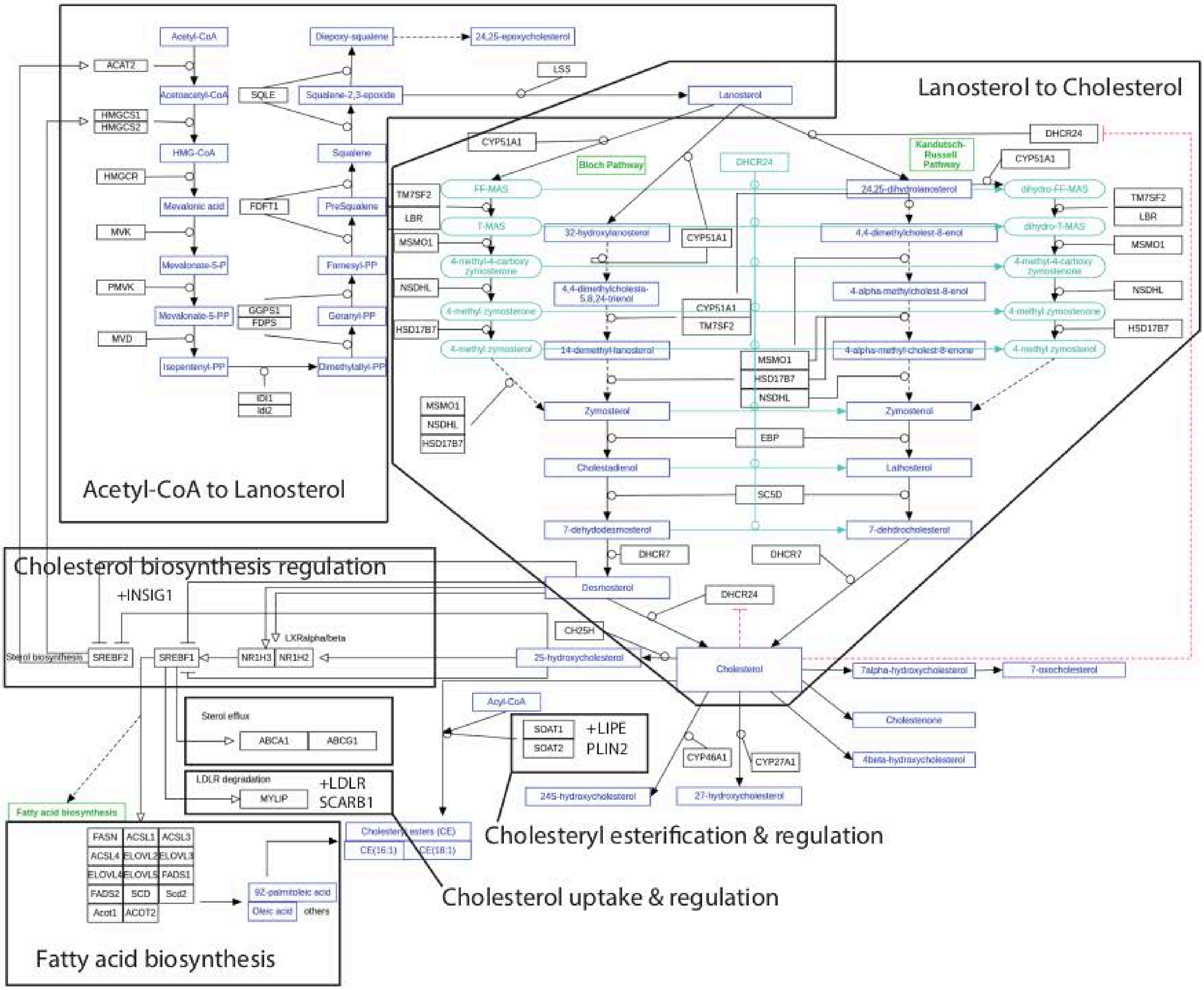

b Cholesterol metabolism pathways (WP4718 based)

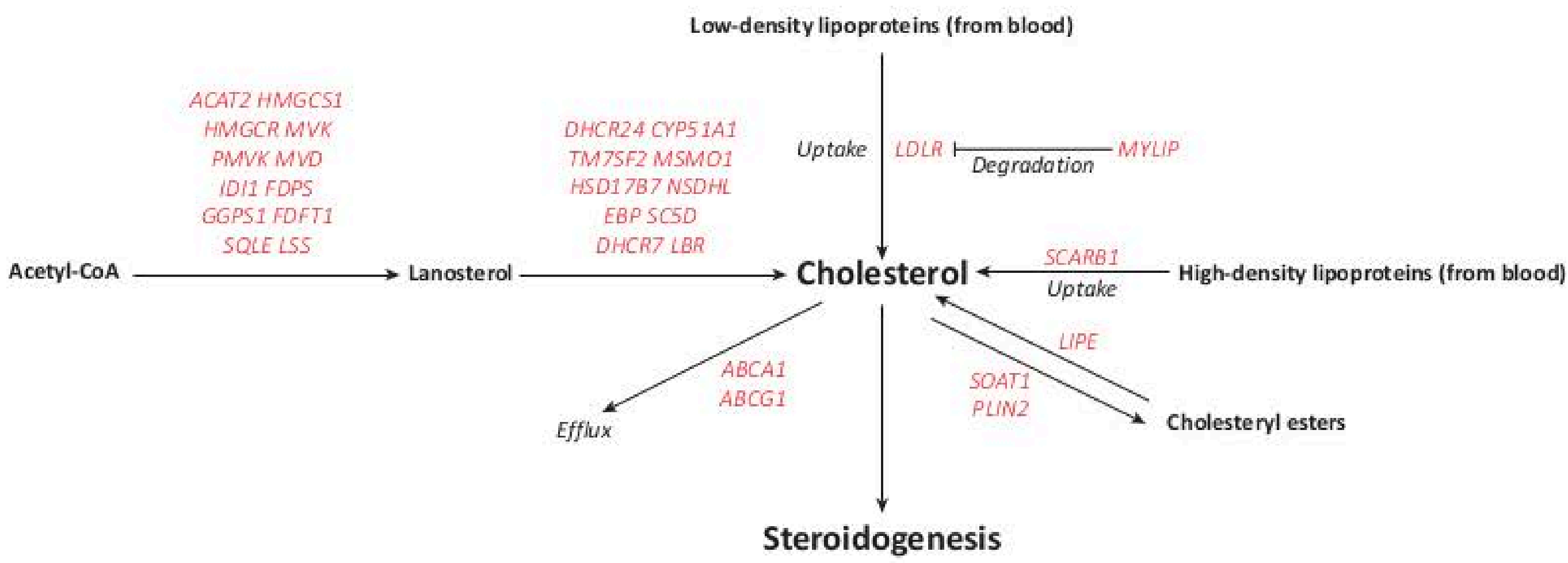

Supplementary Fig. 18 | Scheme of human cholesterol metabolism pathway. a, Scheme of human cholesterol metabolism with detailed focus on biosynthesis from Acetyl-CoA - pathway WP4718 from WikiPathways database “Cholesterol metabolism with Bloch and Kandush-Russell pathways”. Pathway is divided into several key areas highlighted by polygons. **b**, Simplified version of the human cholesterol metabolism pathway scheme (biosynthesis intermediates aren’t shown).

Cholesterol obtaining and usage relationsip

scRNA-seq data

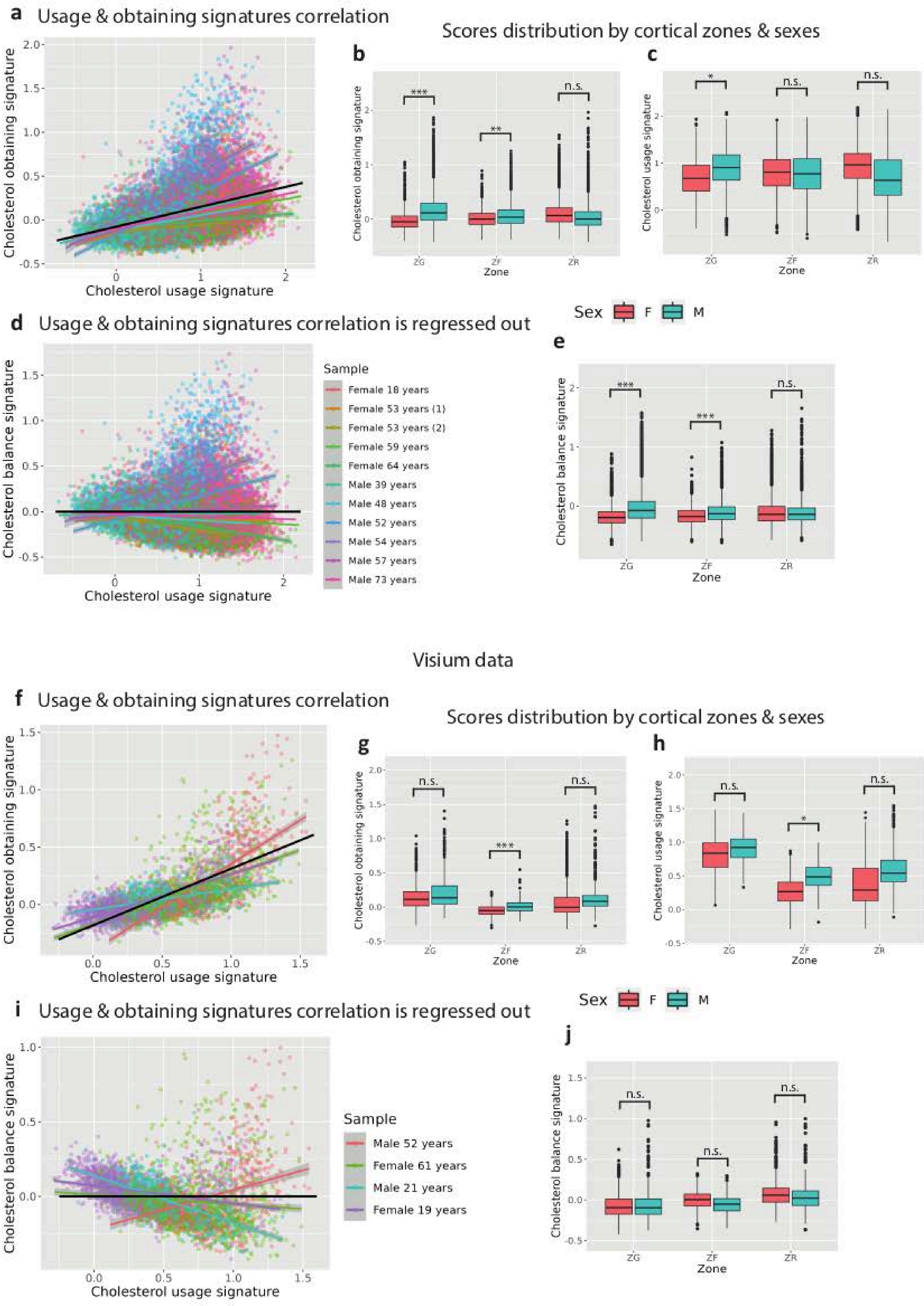

Supplementary Fig. 19 | Cholesterol obtaining & usage relationship analysis in the adult human adrenal cortex. a-e, Analysis of scRNA-seq data (34,433 cells). **f-j**, Analysis of Visium spatial transcriptomics data (4,298 spots). **a,f,** Linear correlation between cholesterol usage (steroidogenesis + esterification) and obtaining (biosynthesis + uptake + esters degradation). Each dot corresponds to a single cell (Visium spot), colors indicate samples. Colored lines indicate sample specific linear regression, while black line - general (linear mixture) regression. **b, c, e, g h, j**, Boxplots of scores distribution in male or female zona glomerulosa (ZG), zona fasciculata (ZF), zona reticularis (ZR). For scRNA-seq data female ZG - 4,610 cells, male ZG - 5,456 cells, female ZF - 3,946 cells, male ZF - 5,894 cells, female ZR - 6,322 cells, male ZR - 8,205 cells. For Visium data female ZG - 764 spots, male ZG - 486 spots, female ZF - 1,086 spots, male ZF - 593 spots, female ZR - 846 spots, male ZR - 523 spots. For statistical testing details see Methods section. **b, g**, Cholesterol obtaining (biosynthesis + uptake + esters degradation) signature expression scores distribution. Adjusted p-values are as follows: **b -** <0.0001, 0.0075, 0.4410, **g -** 0.302, <0.001, 0.302 (ZG, ZF and ZR respectively). **c**, **h**, Cholesterol usage (steroidogenesis + esterification) signature expression scores distribution. Adjusted p-values are as follows: **c -** 0.015, 0.372, 0.219, **h -** 0.147, 0.012, 0.195 (ZG, ZF and ZR respectively). **d**, **i**, Correlation between cholesterol balance score and cholesterol usage signature expression score. Cholesterol balance is a obtaining score after regressing out cholesterol usage score. Each dot corresponds to a single cell (Visium spot), colors indicate samples. Colored lines indicate sample specific linear regression, while black line - general (linear mixture) regression. **e, j**, Cholesterol balance scores distribution. Adjusted p-values are as follows: **e -** <0.001, <0.001, 0.244, **j -** 0.366, 0.237, 0.237 (ZG, ZF and ZR respectively). For each box-plot, center line, median; box limits, upper and lower quartiles; whiskers, 1.5x interquartile range; points, outliers. *, multiple testing adjusted p-value < 0.05; **, p < 0.01; ***, p < 0.001; n.s., p > 0.05 (not significant).

Cholesterol obtaining and usage relationsip

Healthy adult adrenal gland bulk RNA-seq (GTEx, 106 female & 168 male samples)

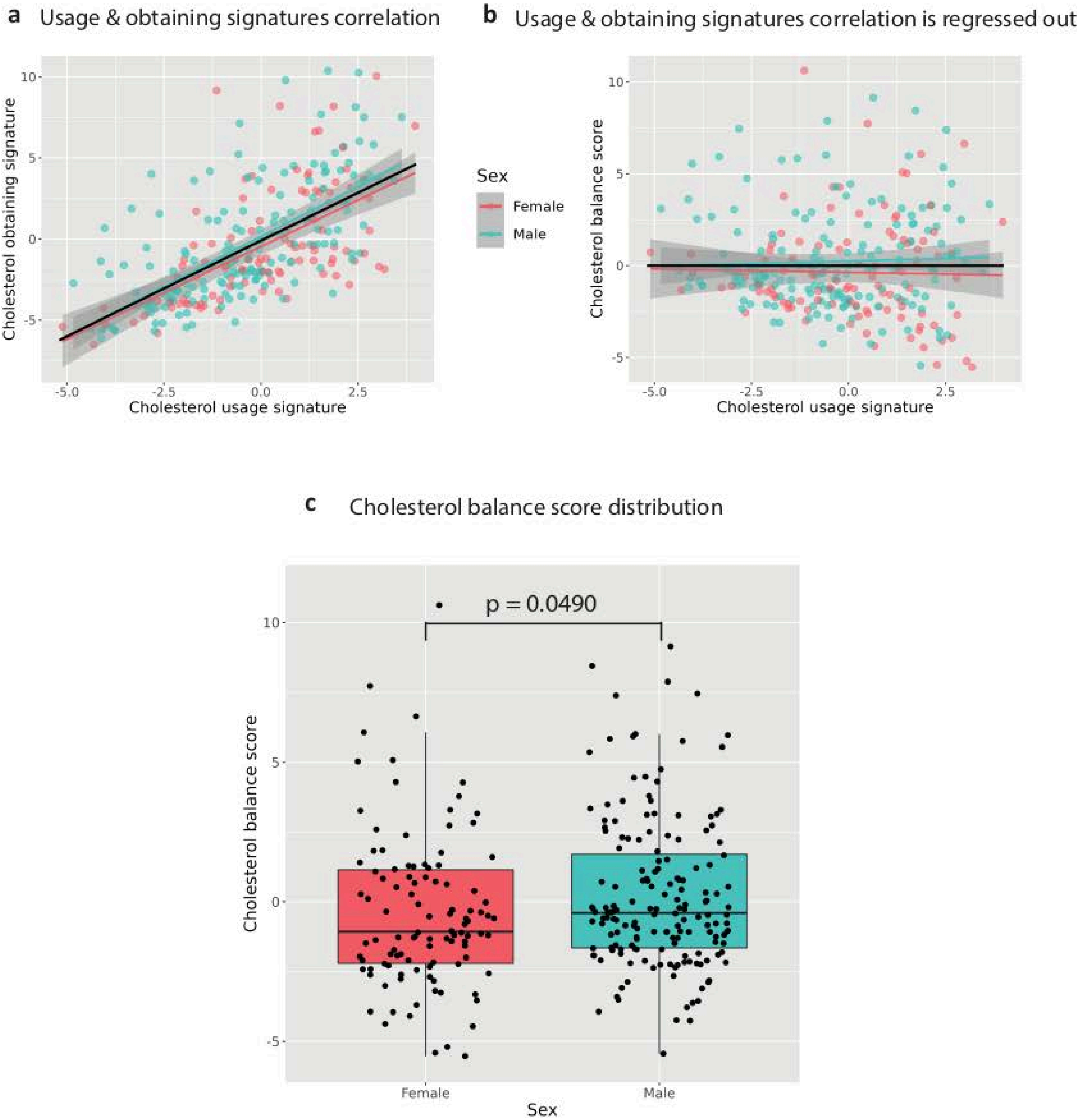

Supplementary Fig. 20 | Sexual dimorphism in cholesterol balance in normal adult adrenal bulk RNA-seq. a, High Pearson correlation (r = 0.64, p-value < 2.2e-16, 95% CI = [0.56, 0.71]) between cholesterol obtaining and usage signatures expression scores gives a basis for regression out the usage signature and cholesterol balance score calculation. Each dot corresponds to a normal adult adrenal bulk RNA-seq sample (n = 274). Correlation between cholesterol balance score and cholesterol usage signature expression score. **b**, Cholesterol balance is a obtaining score after regressing out cholesterol usage score. **c**, Cholesterol balance scores distribution in male and female groups, two-sided Wilcoxon test for comparison. For each box-plot, center line, median; box limits, upper and lower quartiles; whiskers, 1.5x interquartile range; points, outliers.

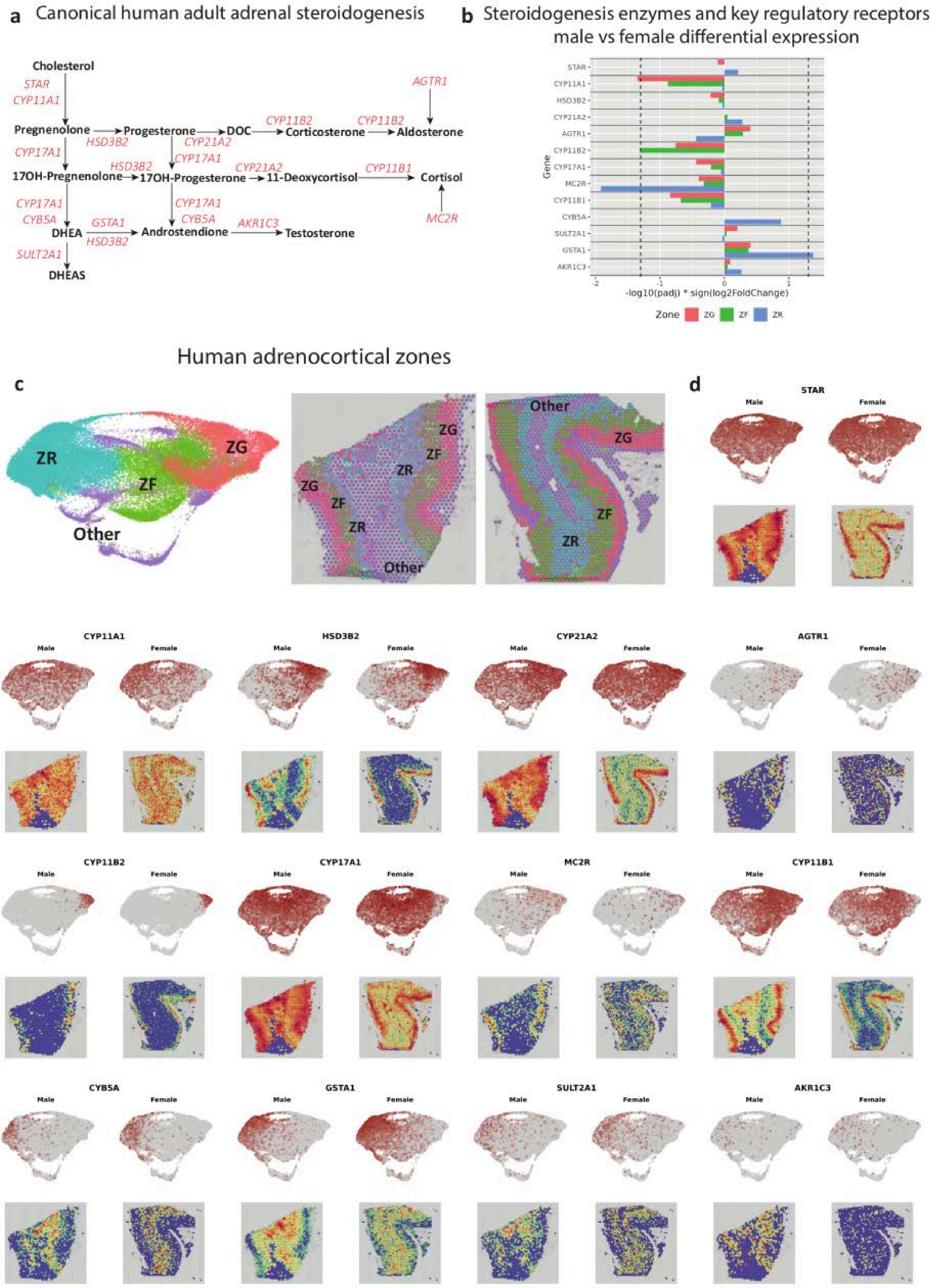
a Canonical human adult adrenal steroidogenesis
Cholesterol
STAR
CYP11A1
Pregnenolone
HSD3B2
Progesterone
DOC
CYP11B2
Corticosterone
CYP11B2
Aldosterone
AGTR1
CYP21A2
CYP17A1
17OH-Pregnenolone
HSD3B2
17OH-Progesterone
CYP21A2
11-Deoxycortisol
CYP11B1
Cortisol
MC2R
CYP17A1
CYB5A
DHEA
GSTA1
HSD3B2
Androstendione
AKR1C3
Testosterone
SULT2A1
DHEAS
b Steroidogenesis enzymes and key regulatory receptors male vs female differential expression
STAR
CYP11A1
HSD3B2
CYP21A2
AGTR1
CYP11B2
CYP17A1
MC2R
CYP11B1
CYB5A
SULT2A1
GSTA1
AKR1C3
Gene
-2
-1
0
1
-log10(padj) * sign(log2FoldChange)
Zone
ZG
ZF
ZR
Human adrenocortical zones
c
ZR
ZF
ZG
Other
d
STAR
Male
Female
CYP11A1
HSD3B2
CYP21A2
AGTR1
CYP11B2
CYP17A1
MC2R
CYP11B1
CYB5A
GSTA1
SULT2A1
AKR1C3

Supplementary Fig. 21 | Expression of steroidogenesis-related genes in the adult human adrenal cortex. a, A schematic diagram illustrating steroidogenesis in the human adrenal gland. **b**, Differential expression between males and females of key genes in each zone (ZG - zona glomerulosa, ZF - zona fasciculata, ZR - zona reticulatis), for each gene three bars are shown, one per zone (scRNA-seq data). The significance (calculated using cacoa R package, adjusted for multiple hypothesis testing with Benjamini-Hochberg method) differences is provided, but positive values correspond to overrepresentation in females, while negative - in males. The vertical line is set at the significance level p-value = 0.05. Female ZG - 4,610 cells, male ZG - 5,456 cells, female ZF - 3,946 cells, male ZF - 5,894 cells, female ZR - 6,322 cells, male ZR - 8,205 cells. **c**, Three cortical zones (ZG, ZF and ZR) on human adult adrenocortical and mesenchymal cells UMAP embedding and two Visium slides - male (left) and female (right). **d**, Expression of each gene from **a, b** on scRNA-seq UMAP and spatial transcriptomics slide in males and females.

a Cholesterol biosynthesis - from Acetyl-CoA to Lanosterol

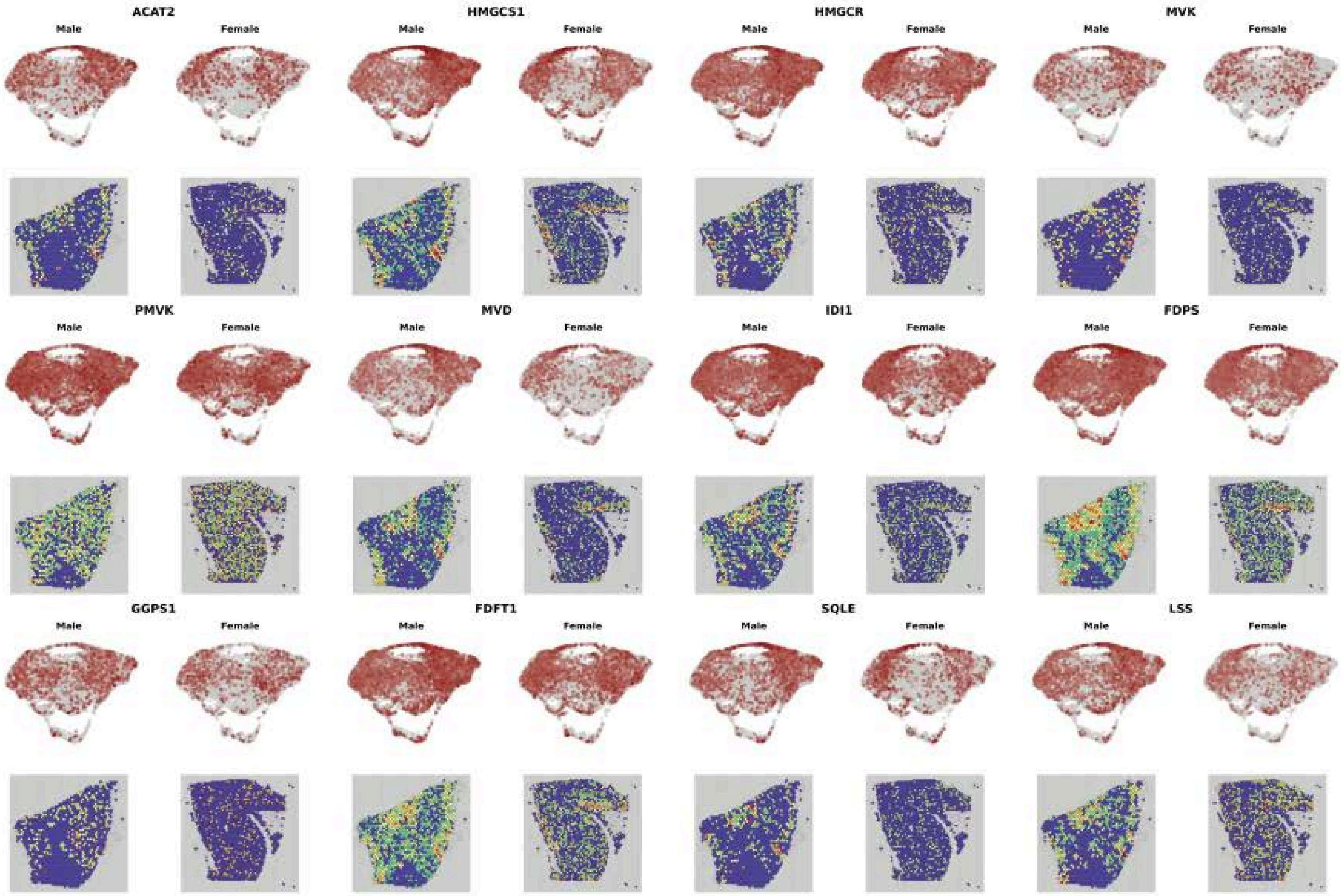

b Cholesterol biosynthesis - from Lanosterol to Cholesterol

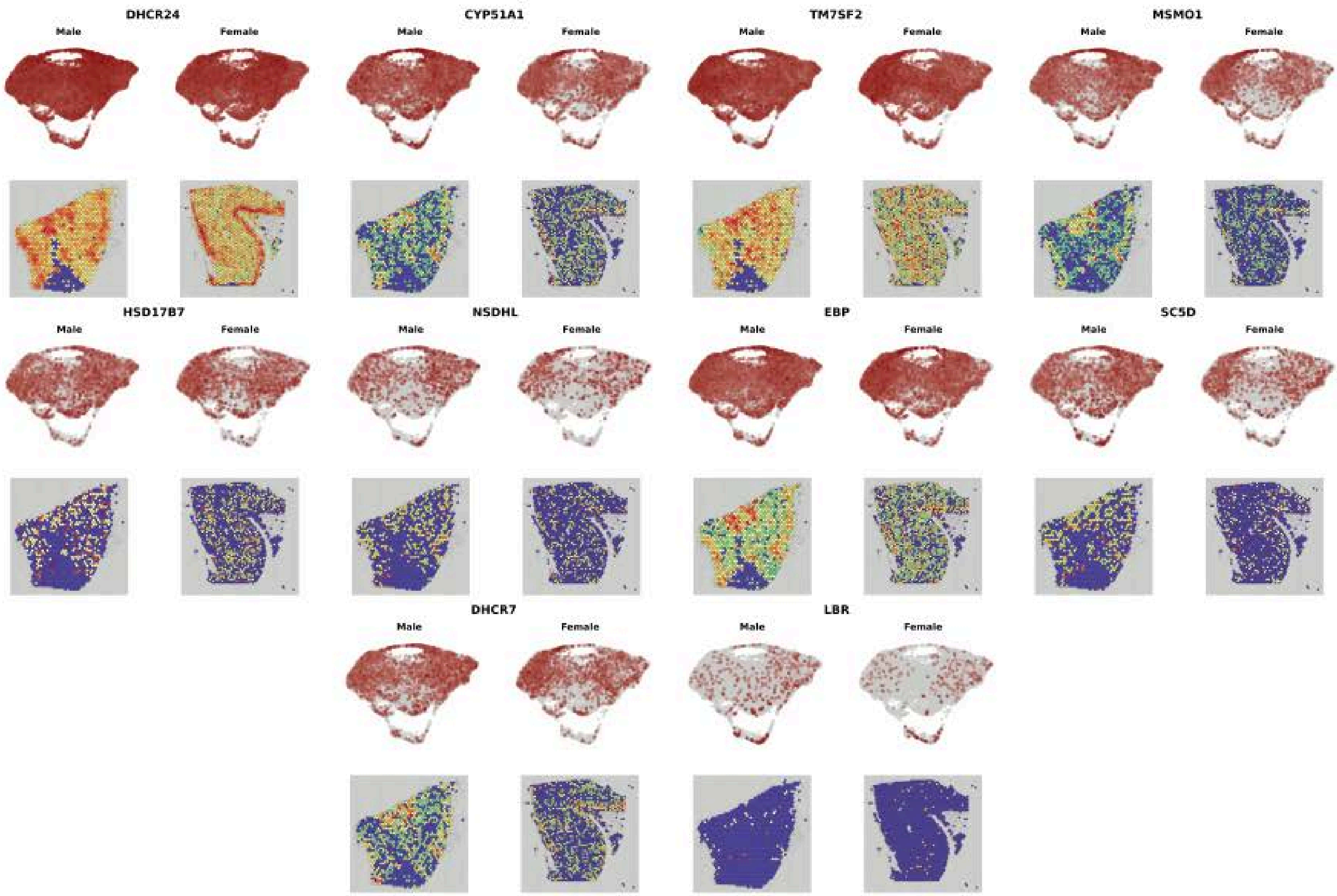

Supplementary Fig. 22 | Expression of genes involved in cholesterol biosynthesis in the adult human adrenal cortex. a-b, Expression of each gene involved in human cholesterol biosynthesis on scRNA-seq UMAP and spatial transcriptomics slide in males and females. **a**, First part of cholesterol biosynthesis pathway - from AcetylCoA to Lanosterol. **b**, Second part of cholesterol biosynthesis pathway - from Lanosterol to Cholesterol.

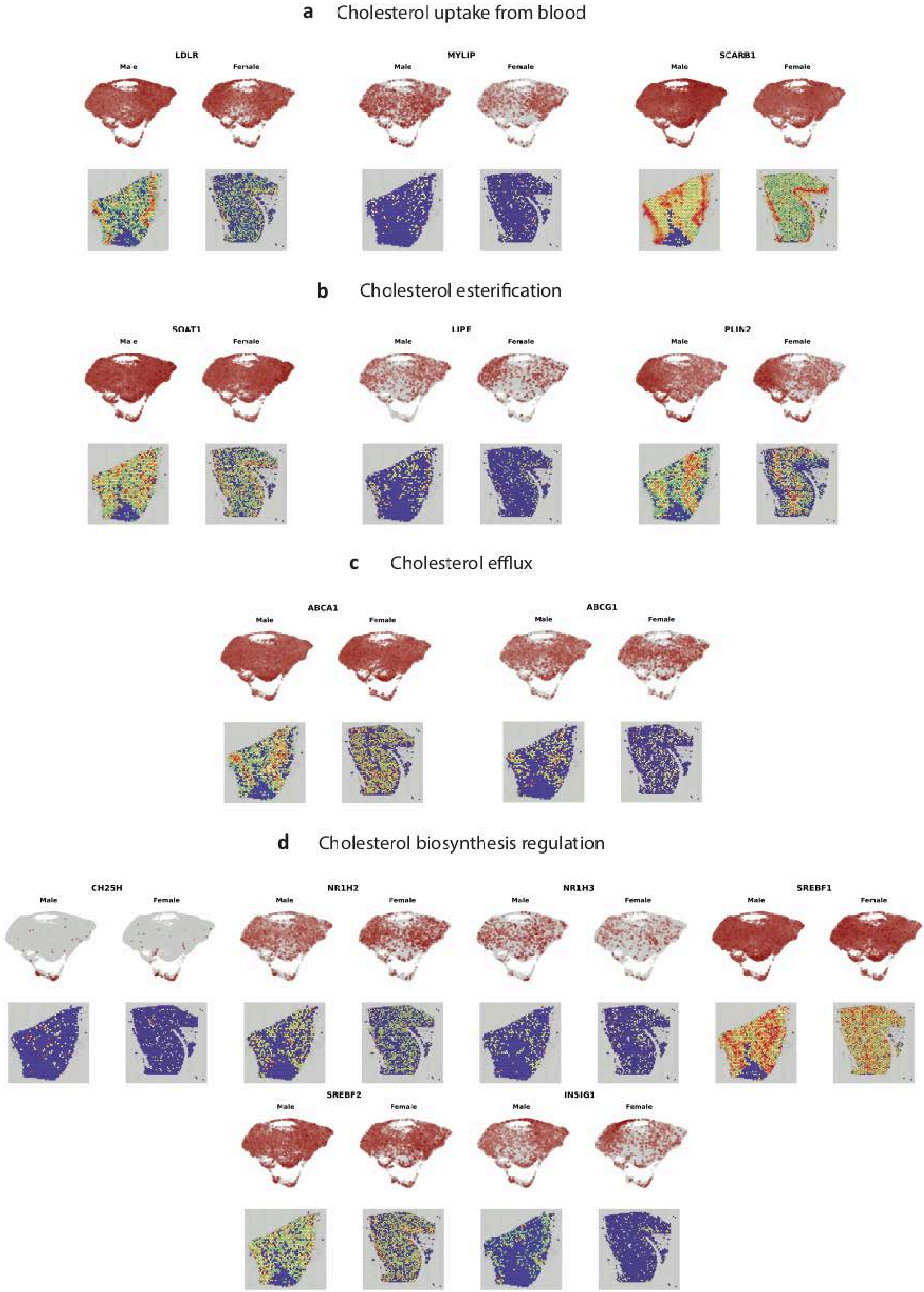
a Cholesterol uptake from blood
LDLR
MYLIP
SCARB1
Male
Female
b Cholesterol esterification
SOAT1
LIPE
PLIN2
c Cholesterol efflux
ABCA1
ABCG1
d Cholesterol biosynthesis regulation
CH25H
NR1H2
NR1H3
SREBF1
SREBF2
INSIG1

Supplementary Fig. 23 | Expression of genes involved in cholesterol uptake, efflux, esterification and metabolism regulation in the adult human adrenal cortex. a-b, Expression of genes involved in human cholesterol metabolism on scRNA-seq UMAP and spatial transcriptomics slide in males and females. **a**, Cholesterol uptake from blood. **b**, Cholesterol esterification. **c**, Cholesterol efflux. **d**, Cholesterol biosynthesis regulation.

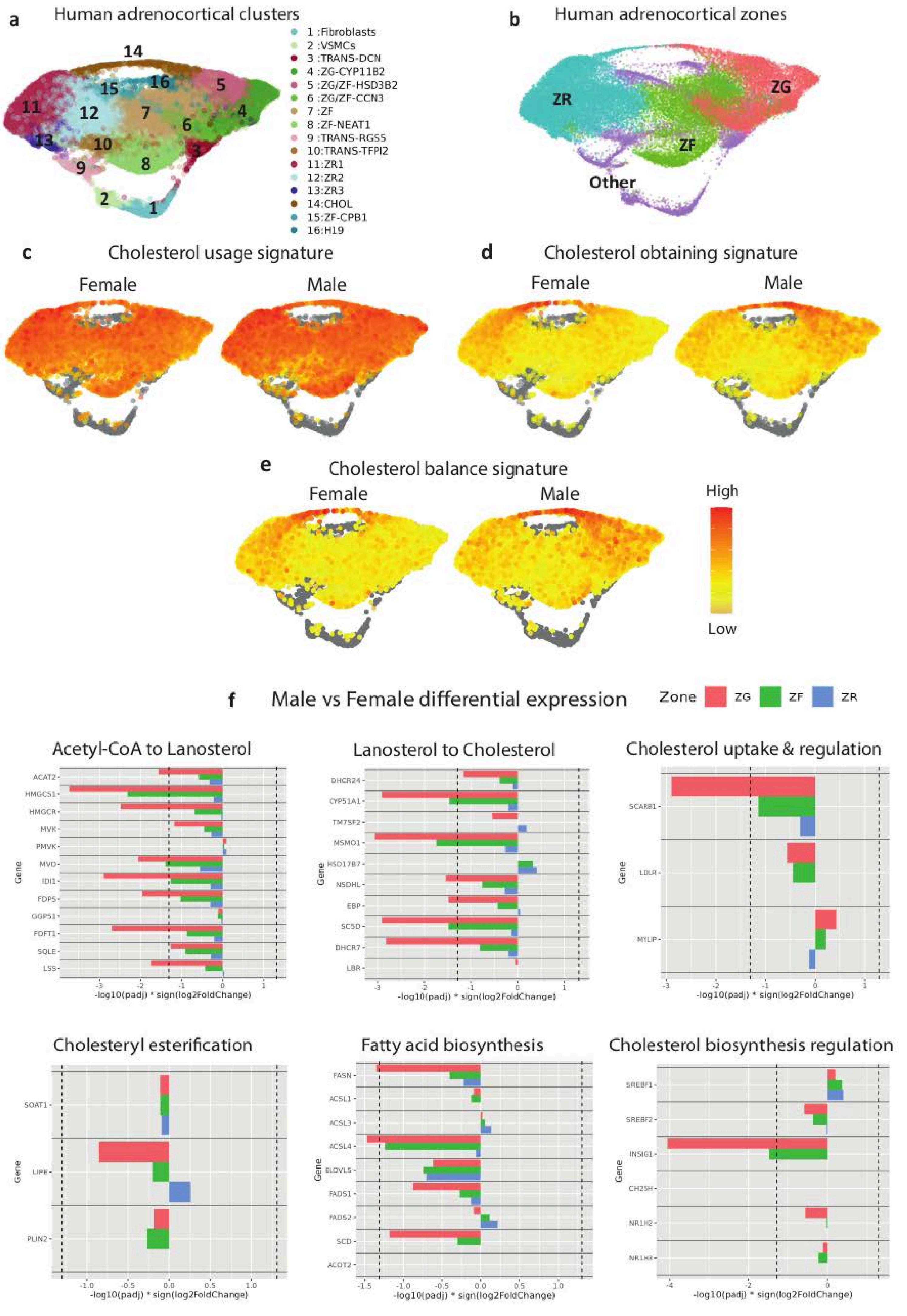
a
Human adrenocortical clusters
1 :Fibroblasts
2 :VSMCs
3 :TRANS-DCN
4 :ZG-CYP11B2
5 :ZG/ZF-HSD3B2
6 :ZG/ZF-CCN3
7 :ZF
8 :ZF-NEAT1
9 :TRANS-RGS5
10:TRANS-TFPI2
11:ZR1
12:ZR2
13:ZR3
14:CHOL
15:ZF-CPB1
16:H19
b
Human adrenocortical zones
ZR
ZG
ZF
Other
c
Cholesterol usage signature
Female
Male
d
Cholesterol obtaining signature
Female
Male
e
Cholesterol balance signature
Female
Male
High
Low
f
Male vs Female differential expression
Zone
ZG
ZF
ZR
Acetyl-CoA to Lanosterol
ACAT2
HMGCS1
HMGCR
MVK
PMVK
MVD
IDI1
FDPS
GGPS1
FDFT1
SQLE
LSS
Gene
-log10(padj) * sign(log2FoldChange)
Lanosterol to Cholesterol
DHCR24
CYP51A1
TM7SF2
MSMO1
HSD17B7
NSDHL
EBP
SC5D
DHCR7
LBR
Cholesterol uptake & regulation
SCARB1
LDLR
MYLIP
Cholesteryl esterification
SOAT1
LIPE
PLIN2
Fatty acid biosynthesis
FASN
ACSL1
ACSL3
ACSL4
ELOVL5
FADS1
FADS2
SCD
ACOT2
Cholesterol biosynthesis regulation
SREBF1
SREBF2
INSIG1
CH25H
NR1H2
NR1H3

Supplementary Fig. 24 | Cholesterol obtaining, usage (metabolic break-down) and balance scores on scRNA-seq UMAP. a, Human adult adrenocortical and mesenchymal cells embedding (39,985 cells). **b**, Three cortical zones (ZG, ZF and ZR) on human adult adrenocortical and mesenchymal cells UMAP embedding. Female ZG - 4,610 cells, male ZG - 5,456 cells, female ZF - 3,946 cells, male ZF - 5,894 cells, female ZR - 6,322 cells, male ZR - 8,205 cells. **c-e**, Scores visualization on human adult adrenocortical and mesenchymal cells embedding (in total 34,433 cells). **c**, Cholesterol usage signature expression score distribution. **d**, Cholesterol obtaining signature expression score distribution. **e**, Cholesterol balance score distribution. **f**, Differential expression between males and females (in scRNA-seq data) of key cholesterol metabolism pathway genes in each zone (ZG - zona glomerulosa, ZF - zona fasciculata, ZR - zona reticulatis), for each gene three bars are shown, one per zone. The significance (calculated using cacoa R package, adjusted for multiple hypothesis testing with Benjamini-Hochberg method) differences is provided, but positive values correspond to overrepresentation in females, while negative - in males. The vertical line is set at the significance level p-value = 0.05.

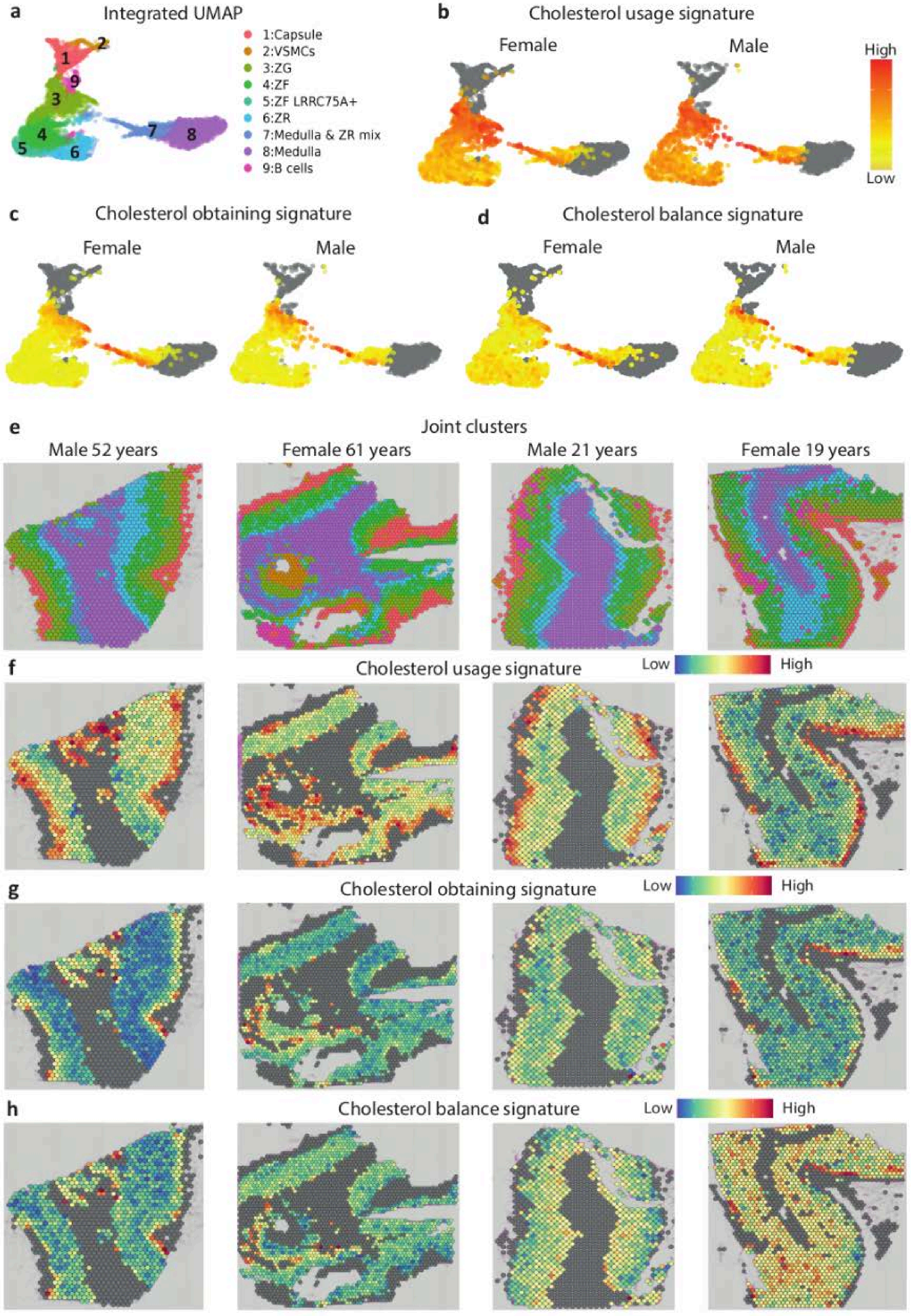

a
Integrated UMAP
1:Capsule
2:VSMCs
3:ZG
4:ZF
5:ZF LRRC75A+
6:ZR
7:Medulla & ZR mix
8:Medulla
9:B cells
b
Cholesterol usage signature
Female
Male
High
Low
c
Cholesterol obtaining signature
Female
Male
d
Cholesterol balance signature
Female
Male
e
Joint clusters
Male 52 years
Female 61 years
Male 21 years
Female 19 years
f
Cholesterol usage signature
Low
High
g
Cholesterol obtaining signature
Low
High
h
Cholesterol balance signature
Low
High

Supplementary Fig. 25 | Cholesterol obtaining, usage and balance scores on Visium slides and integrated UMAP. a, Joint clusters on the integrated UMAP embedding of four Visium samples (6,984 spots). **b-d**, Scores visualization on integrated Visium UMAP embedding. Total 4,298 spots, female ZG - 764 spots, male ZG - 486 spots, female ZF - 1,086 spots, male ZF - 593 spots, female ZR - 846 spots, male ZR - 523 spots. **b**, Cholesterol usage signature expression score distribution. **c**, Cholesterol obtaining signature expression score distribution. **d**, Cholesterol balance score distribution. **e**, Joint clusters of four Visium samples on tissue slide for each sample. **f-h,** Scores visualization on Visium tissue slide for each sample. **f**, Cholesterol usage signature expression score distribution. **g**, Cholesterol obtaining signature expression score distribution. **h**, Cholesterol balance score distribution.

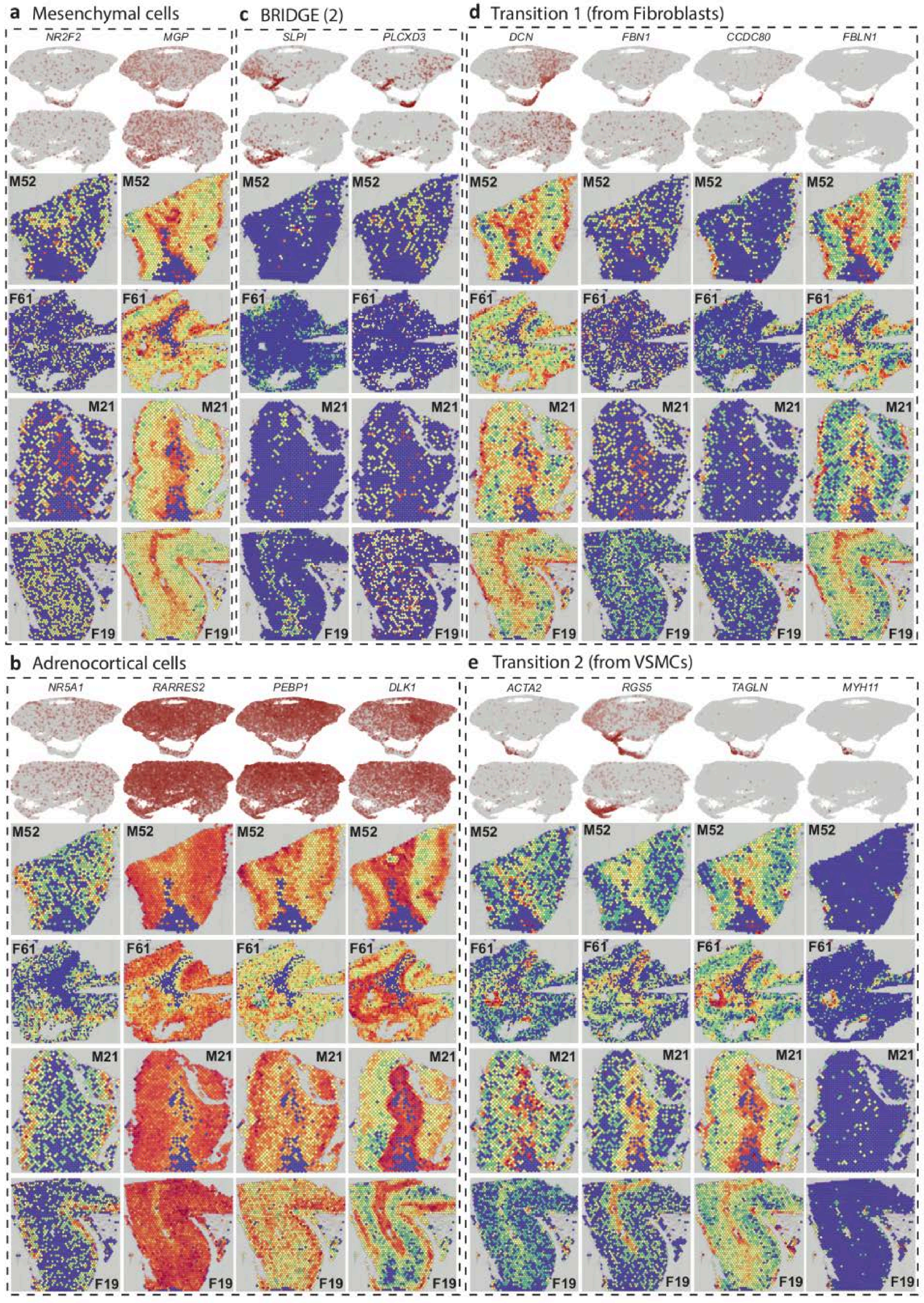

a Mesenchymal cells
NR2F2
MGP
c BRIDGE (2)
SLPI
PLCXD3
d Transition 1 (from Fibroblasts)
DCN
FBN1
CCDC80
FBLN1
b Adrenocortical cells
NR5A1
RARRES2
PEBP1
DLK1
e Transition 2 (from VSMCs)
ACTA2
RGS5
TAGLN
MYH11
M52
F61
M21
F19

Supplementary Fig. 26 | Marker genes of cell populations and transitions involved in adrenal renewal in a healthy adult (Part 1). a, Mesenchymal cells marker genes. **b**, Adrenocortical cells marker genes. **c**, Markers of transitional state between clusters 1 and 3 - cluster 2:BRIDGE on the focused embedding (Fig. 4). **d**, Markers of transition 1 on general UMAP (from cluster 1:Fibroblasts to cluster 3:TRANS-DCN - DCN+ ZG cells). **e**, Markers of transition 2 on general UMAP (from cluster 2:VSMCs - vascular smooth muscle cells to cluster 9:TRANS-RGS5).

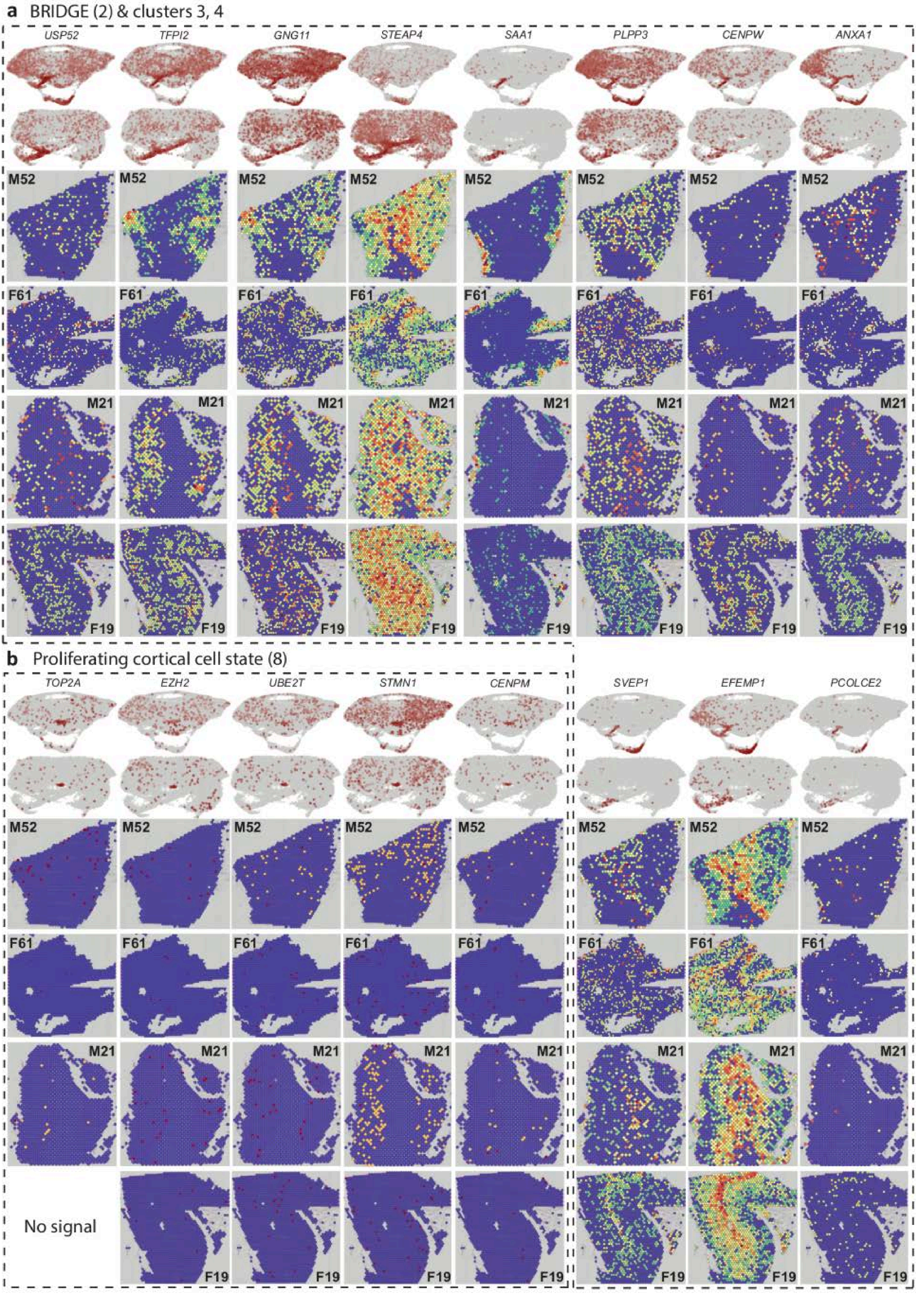

a BRIDGE (2) & clusters 3, 4
USP52
TFPI2
GNG11
STEAP4
SAA1
PLPP3
CENPW
ANXA1
M52
F61
M21
F19
b Proliferating cortical cell state (8)
TOP2A
EZH2
UBE2T
STMN1
CENPM
SVEP1
EFEMP1
PCOLCE2
No signal

Supplementary Fig. 27 | Marker genes of cell populations and transitions involved in adrenal renewal in a healthy adult (Part 2). **a**, Markers of transitional state between clusters 1 and 3 - cluster 2:BRIDGE on the focused embedding. **b**, Markers of proliferating cortical cells - cluster 8:ProlCS on the focused UMAP.

Transition 1 (from Fibroblasts)

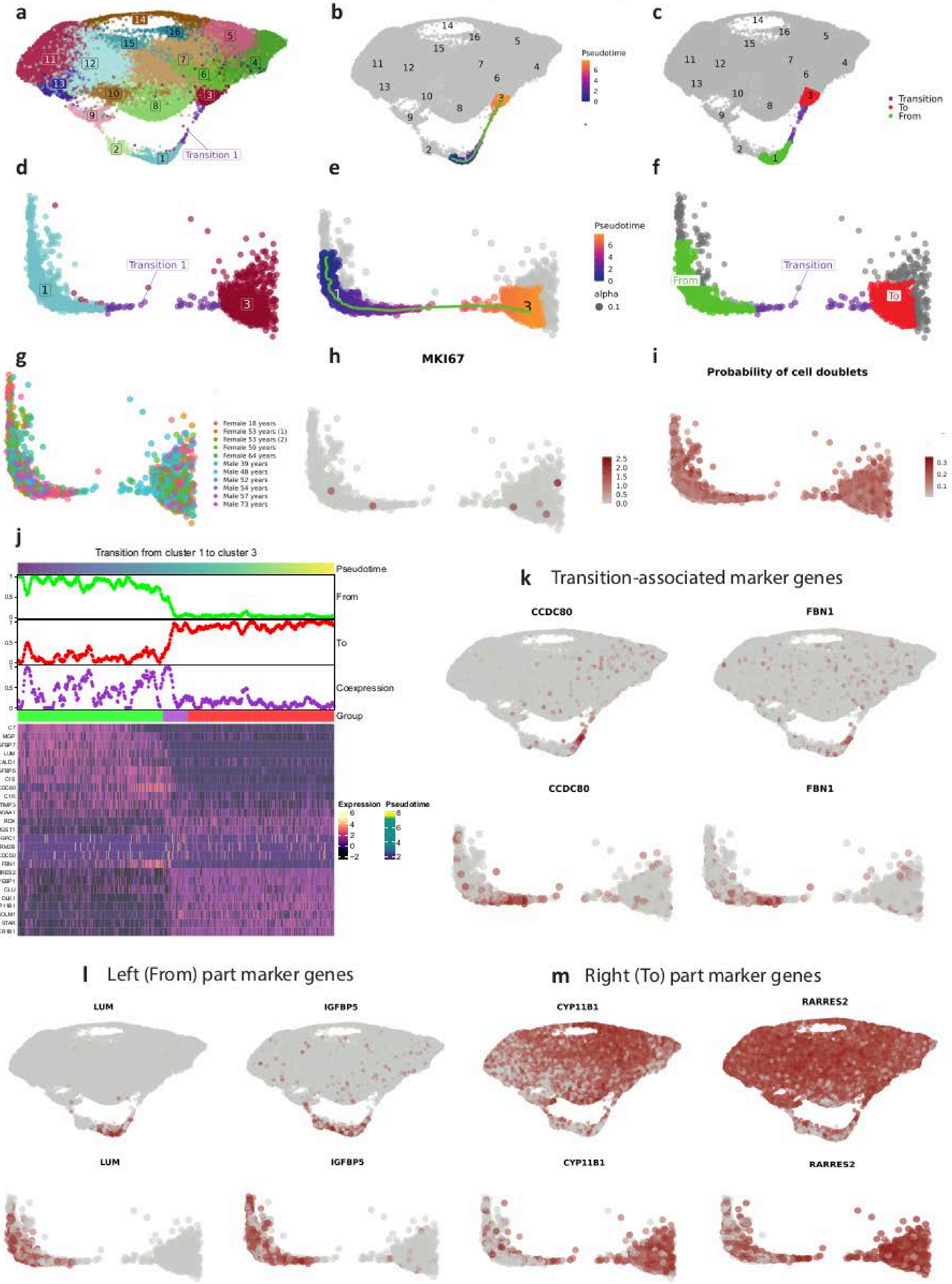

Supplementary Fig. 28 | Transition 1 between cluster 1:Fibroblasts (mesenchyme) and cluster 3:ZG-DCN (adrenal cortex). **a**, Transition 1 on human adult adrenocortical and mesenchymal cells UMAP embedding (39,985 cells). **b**, Corresponding trajectory and pseudotime analysis. **c**, Key transition parts - source cluster (From - 1:Fibroblasts, 798 cells), target cluster (To - 3:ZG-DCN, 547 cells), putative intermediate state (Transition, 50 cells). **d-f**, Similar to **a-c**, but only the part of UMAP of the trajectory location is shown (1,705 cells, for a more detailed view). **g**, Per sample compositional impact. **h**, *MKI67* gene expression (proliferating cells). **i**, Scrublet score (Probability of cell doublets) distribution. **j**, Heatmap of gene expression dynamics in the Transition 1. From top to bottom, first panel, pseudotime during transition. Second panel, expression magnitude of From (1:Fibroblasts) gene expression program in selected cells. Third panel, expression magnitude of To (3:ZG-DCN) gene expression programs in selected cells. Fourth panel, coexpression of the two programs. Fifth panel, transition part, green - From, red - To, dark orchid - Transition. Sixth panel, expression magnitude of the selected genes, visualized as a heatmap. **k-m**, Gene expression, visualized on the whole UMAP or its transition-related part. **k**, Genes, associated with Transition intermediate state. **l**, Left (From) part marker genes. **m**, Right (To) part marker genes.

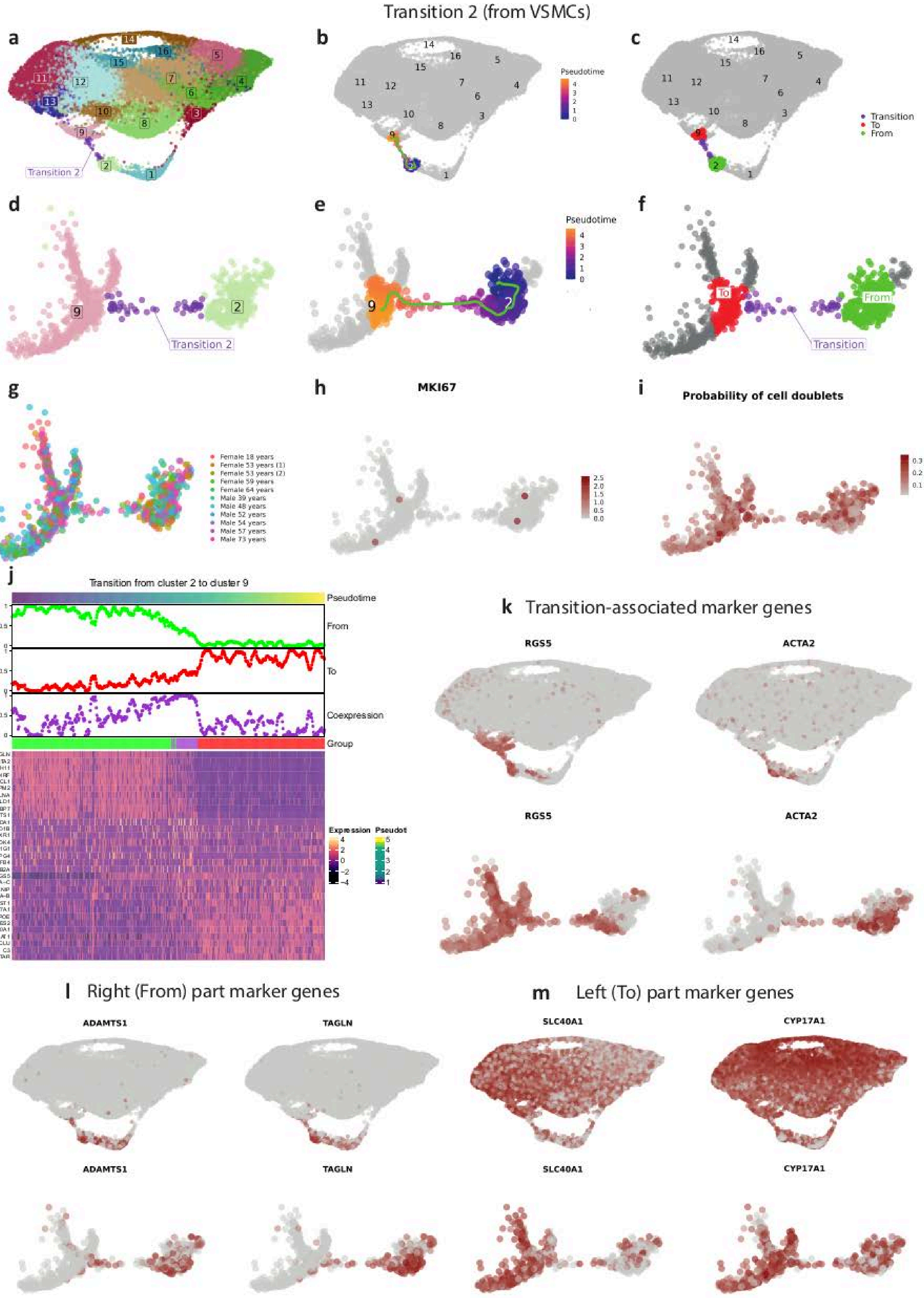

Transition 2 (from VSMCs)
a
b
c
Transition 2
Pseudotime
Transition
To
From
d
e
f
Transition 2
Pseudotime
To
From
Transition
g
h
i
MKI67
Probability of cell doublets
Female 18 years
Female 53 years (1)
Female 53 years (2)
Female 59 years
Female 64 years
Male 39 years
Male 48 years
Male 52 years
Male 54 years
Male 57 years
Male 73 years
j
Transition from cluster 2 to cluster 9
Pseudotime
From
To
Coexpression
Group
Expression
k
Transition-associated marker genes
RGS5
ACTA2
RGS5
ACTA2
l
Right (From) part marker genes
ADAMTS1
TAGLN
ADAMTS1
TAGLN
m
Left (To) part marker genes
SLC40A1
CYP17A1
SLC40A1
CYP17A1

Supplementary Fig. 29 | Transition 2 between cluster 2:VSMCs (mesenchyme) and cluster 9:TRANS-RGS5. a, Transition 2 on human adult adrenocortical and mesenchymal cells UMAP embedding. **b**, Corresponding trajectory and pseudotime analysis. **c**, Key transition parts - source cluster (From - 2:VSMCs, 470 cells), target cluster (To - 9:TRANS-RGS5, 237 cells), putative intermediate state (Transition, 46 cells). **d-f**, Similar to **a-c**, but only the part of UMAP of the trajectory location is shown (1,156 cells, for a more detailed view). **g**, Per sample compositional impact. **h**, *MKI67* gene expression (proliferating cells). **i**, Scrublet score (Probability of cell doublets) distribution. **j**, Heatmap of gene expression dynamics in the Transition 1. From top to bottom, first panel, pseudotime during transition. Second panel, expression magnitude of From (2:VSMCs) gene expression program in selected cells. Third panel, expression magnitude of To (9:TRANS-RGS5) gene expression programs in selected cells. Fourth panel, coexpression of the two programs. Fifth panel, transition part, green - From, red - To, dark orchid - Transition. Sixth panel, expression magnitude of the selected genes, visualized as a heatmap. **k-m**, Gene expression, visualized on the whole UMAP or its transition-related part. **k**, Genes, associated with Transition intermediate state. **l**, Right (From) part marker genes. **m**, Left (To) part marker genes.

Transition 3 (from cluster 9 to cluster 10)

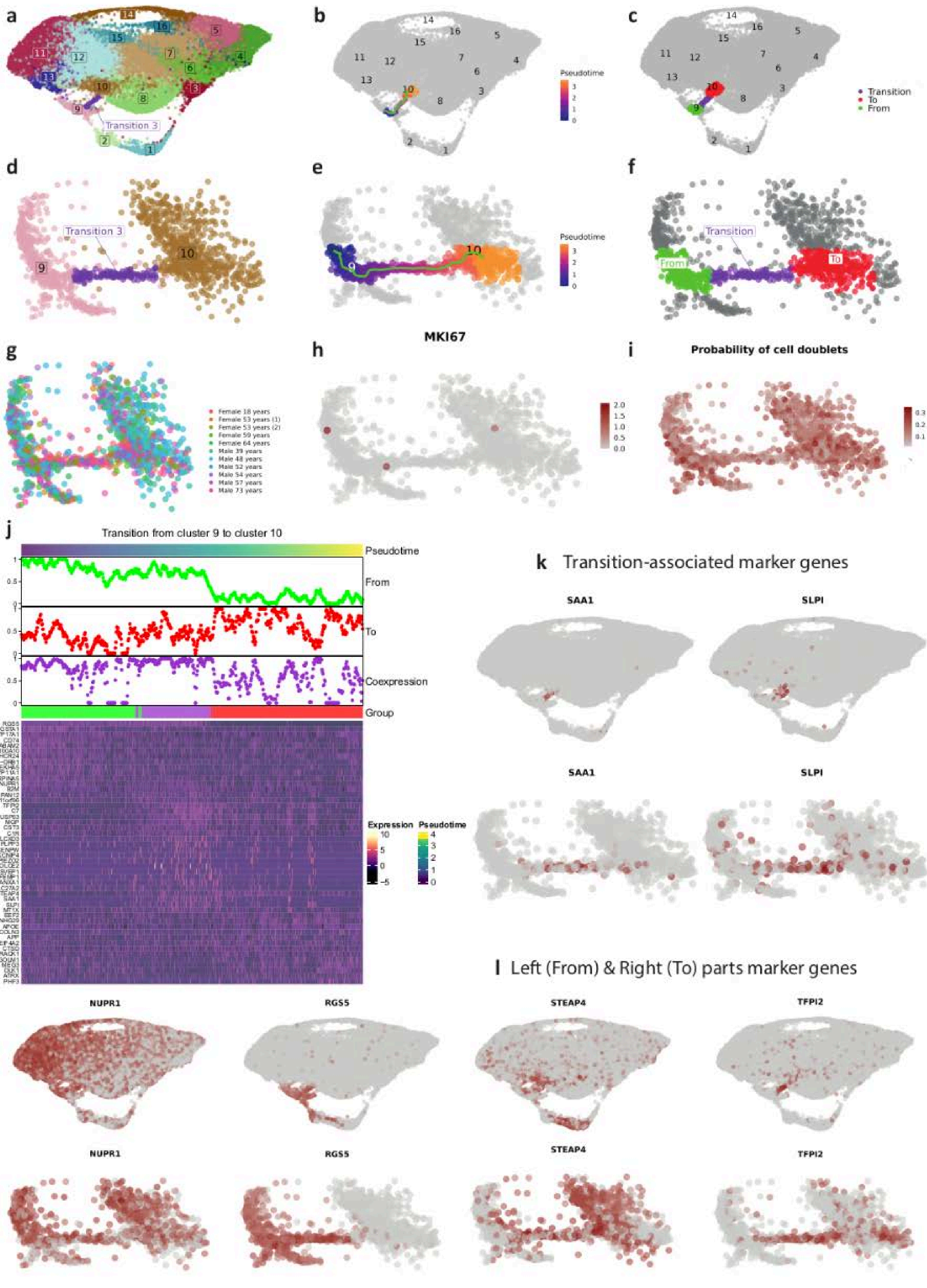

Supplementary Fig. 30 | Transition 3 between cluster 9:TRANS-RGS5 and cluster 10:TRANS-TFPI2. **a**, Transition 3 on human adult adrenocortical and mesenchymal cells UMAP embedding. **b**, Corresponding trajectory and pseudotime analysis. **c**, Key transition parts - source cluster (From - 9:TRANS-RGS5, 231 cells), target cluster (To - 10:TRANS-TFPI2, 304 cells), putative intermediate state (Transition, 142 cells). **d-f**, Similar to **a-c**, but only the part of UMAP of the trajectory location is shown (1,352 cells, for a more detailed view). **g**, Per sample compositional impact. **h**, *MKI67* gene expression (proliferating cells). **i**, Scrublet score (Probability of cell doublets) distribution. **j**, Heatmap of gene expression dynamics in the Transition 1. From top to bottom, first panel, pseudotime during transition. Second panel, expression magnitude of From (9:TRANS-RGS5) gene expression program in selected cells. Third panel, expression magnitude of To (10:TRANS-TFPI2) gene expression programs in selected cells. Fourth panel, coexpression of the two programs. Fifth panel, transition part, green - From, red - To, dark orchid - Transition. Sixth panel, expression magnitude of the selected genes, visualized as a heatmap. **k-m**, Gene expression, visualized on the whole UMAP or its transition-related part. **k**, Genes, associated with Transition intermediate state. **l**, Right (From) and Left (To) part marker genes.

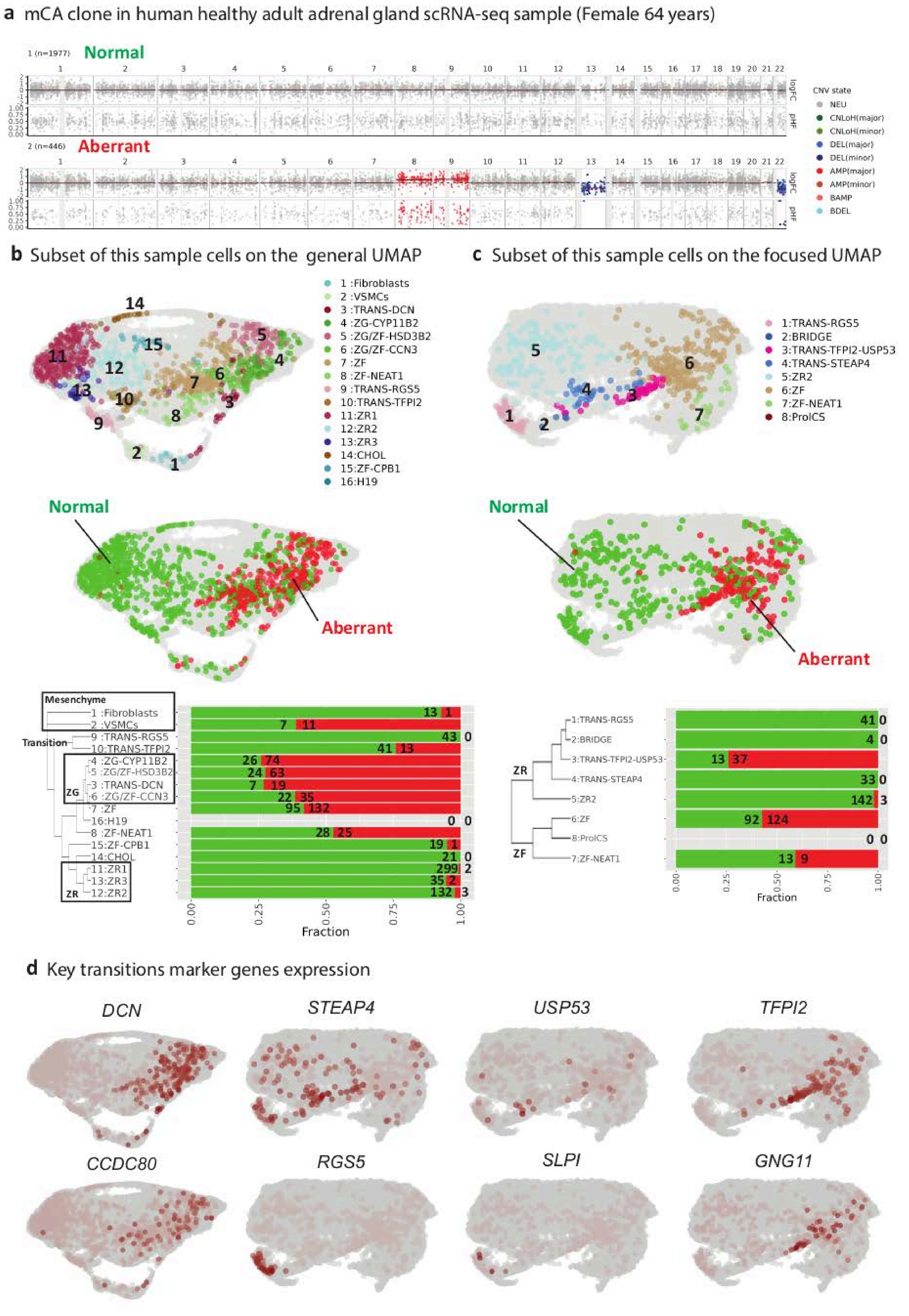
a mCA clone in human healthy adult adrenal gland scRNA-seq sample (Female 64 years)
1 (n=1977) Normal
2 (n=446) Aberrant
logFC
pHF
CNV state
NEU
CNLoH(major)
CNLoH(minor)
DEL(major)
DEL(minor)
AMP(major)
AMP(minor)
BAMP
BDEL
b Subset of this sample cells on the general UMAP
1 :Fibroblasts
2 :VSMCs
3 :TRANS-DCN
4 :ZG-CYP11B2
5 :ZG/ZF-HSD3B2
6 :ZG/ZF-CCN3
7 :ZF
8 :ZF-NEAT1
9 :TRANS-RGS5
10:TRANS-TFPI2
11:ZR1
12:ZR2
13:ZR3
14:CHOL
15:ZF-CPB1
16:H19
c Subset of this sample cells on the focused UMAP
1:TRANS-RGS5
2:BRIDGE
3:TRANS-TFPI2-USP53
4:TRANS-STEAP4
5:ZR2
6:ZF
7:ZF-NEAT1
8:ProlCS
Normal
Aberrant
Mesenchyme
Transition
ZG
ZR
ZF
Fraction
d Key transitions marker genes expression
DCN
STEAP4
USP53
TFPI2
CCDC80
RGS5
SLPI
GNG11

Supplementary Fig. 31 | Somatic CNV clone analysis in healthy human adult adrenal. **a**, Copy number profile inferred by Numbat in scRNA-seq data. **b,c**, Normal and Aberrant cell fractions on embeddings. Upper panel - subset of cells from scRNA-seq sample in analysis (Female 64 years) on embedding. Middle panel - compositional impact of normal and aberrant cell fraction. Lower - proportion of aberrant cells in each cluster (numbers indicate cells count). **b**, General adrenocortical and mesenchymal cells embedding (1,193 cells from sample in analysis). **c**, Focused embedding (511 cells from sample in analysis). **d**, Expression of key transition-associated genes on the focused UMAP embedding.

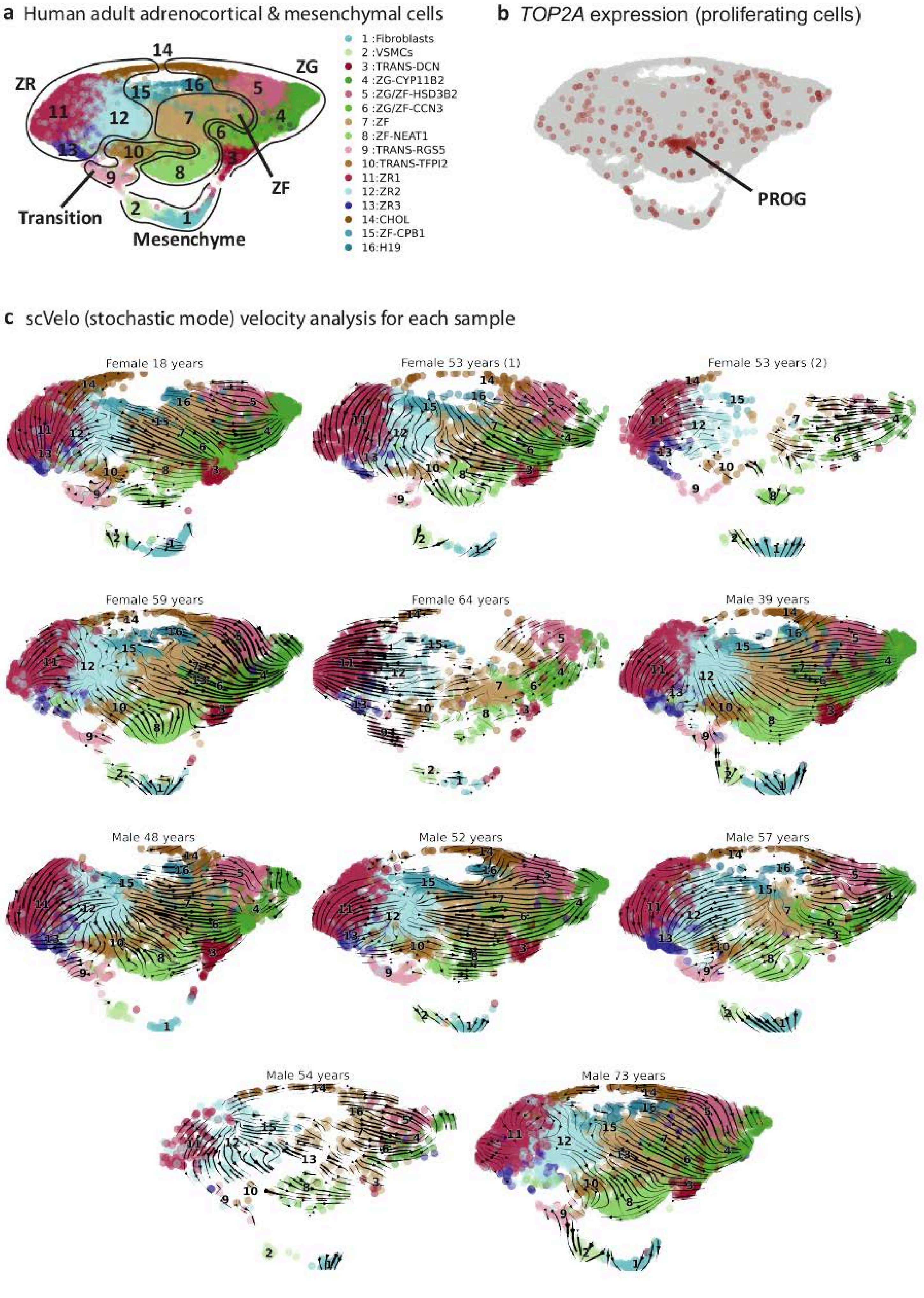
a Human adult adrenocortical & mesenchymal cells
1 :Fibroblasts
2 :VSMCs
3 :TRANS-DCN
4 :ZG-CYP11B2
5 :ZG/ZF-HSD3B2
6 :ZG/ZF-CCN3
7 :ZF
8 :ZF-NEAT1
9 :TRANS-RGS5
10:TRANS-TFPI2
11:ZR1
12:ZR2
13:ZR3
14:CHOL
15:ZF-CPB1
16:H19
ZR
ZG
ZF
Transition
Mesenchyme
b TOP2A expression (proliferating cells)
PROG
c scVelo (stochastic mode) velocity analysis for each sample
Female 18 years
Female 53 years (1)
Female 53 years (2)
Female 59 years
Female 64 years
Male 39 years
Male 48 years
Male 52 years
Male 57 years
Male 54 years
Male 73 years

Supplementary Fig. 32 | Velocity analysis of human adult adrenal samples. **a**, Human adult adrenocortical and mesenchymal cells embedding (39,985 cells). **b**, *TOP2A* gene expression indicates location of proliferating cells on UMAP. Actively proliferating adrenocortical cells (ProlCS) in the central part of the embedding **c**, Velocity analysis for each individual sample projected on joint embedding.

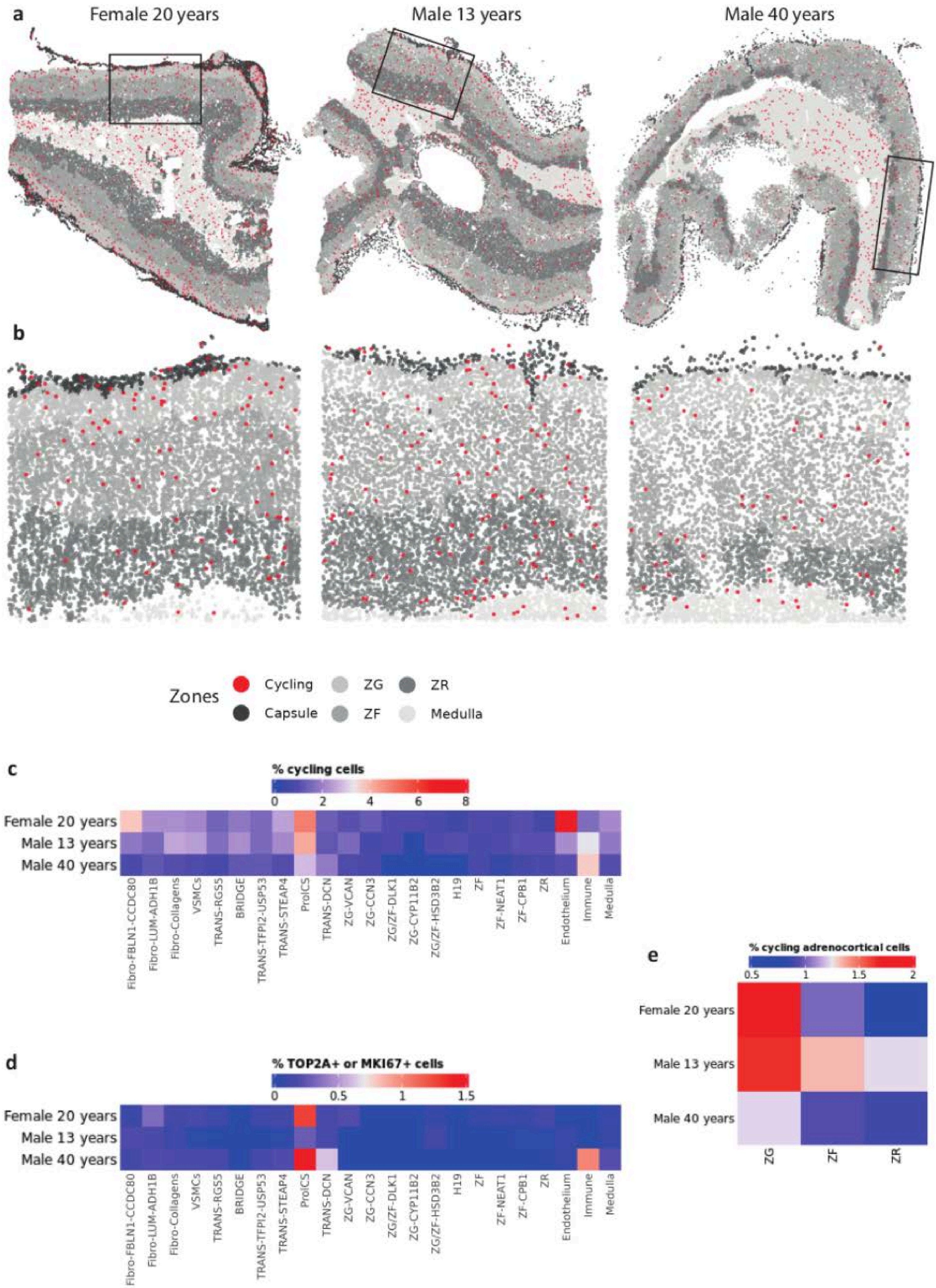

a
Female 20 years
Male 13 years
Male 40 years
b
Zones
Cycling
Capsule
ZG
ZF
ZR
Medulla
c
% cycling cells
0
2
4
6
8
Female 20 years
Male 13 years
Male 40 years
Fibro-FBLN1-CCDC80
Fibro-LUM-ADH1B
Fibro-Collagens
VSMCs
TRANS-RGS5
BRIDGE
TRANS-TFPI2-USP53
TRANS-STEAP4
ProlCS
TRANS-DCN
ZG-VCAN
ZG-CCN3
ZG/ZF-DLK1
ZG-CYP11B2
ZG/ZF-HSD3B2
H19
ZF
ZF-NEAT1
ZF-CPB1
ZR
Endothelium
Immune
Medulla
d
% TOP2A+ or MKI67+ cells
0
0.5
1
1.5
e
% cycling adrenocortical cells
0.5
1
1.5
2
ZG
ZF
ZR

Supplementary Fig. 33 | Spatial distribution of cycling cells in human Stereo-seq data. **a**, Cycling cells (defined as cells in G2M or S phases) on whole tissue sections. Selected areas with intact and representative layered structure are highlighted with rectangles. **b**, Cycling cells on selected section areas. **c-d**, Heatmaps representing for each sample which percent of cells of each cell type were identified as cycling. **c**, Cycling cells were identified using AUCell and mitotic signature (see Methods section). **d**, Cycling cells were identified as those having *MKI67* or *TOP2A* genes expression above zero. **e**, Heatmap representing for each sample which percent of cells of each zone were identified as cycling. Only cells annotated as adrenocortical included (i.e. without mesenchyme, endothelium etc.). Cycling cells were identified using AUCell and mitotic signature (see Methods section). ZG, zona glomerulosa; ZF, zona fasciculata; ZR, zona reticularis.

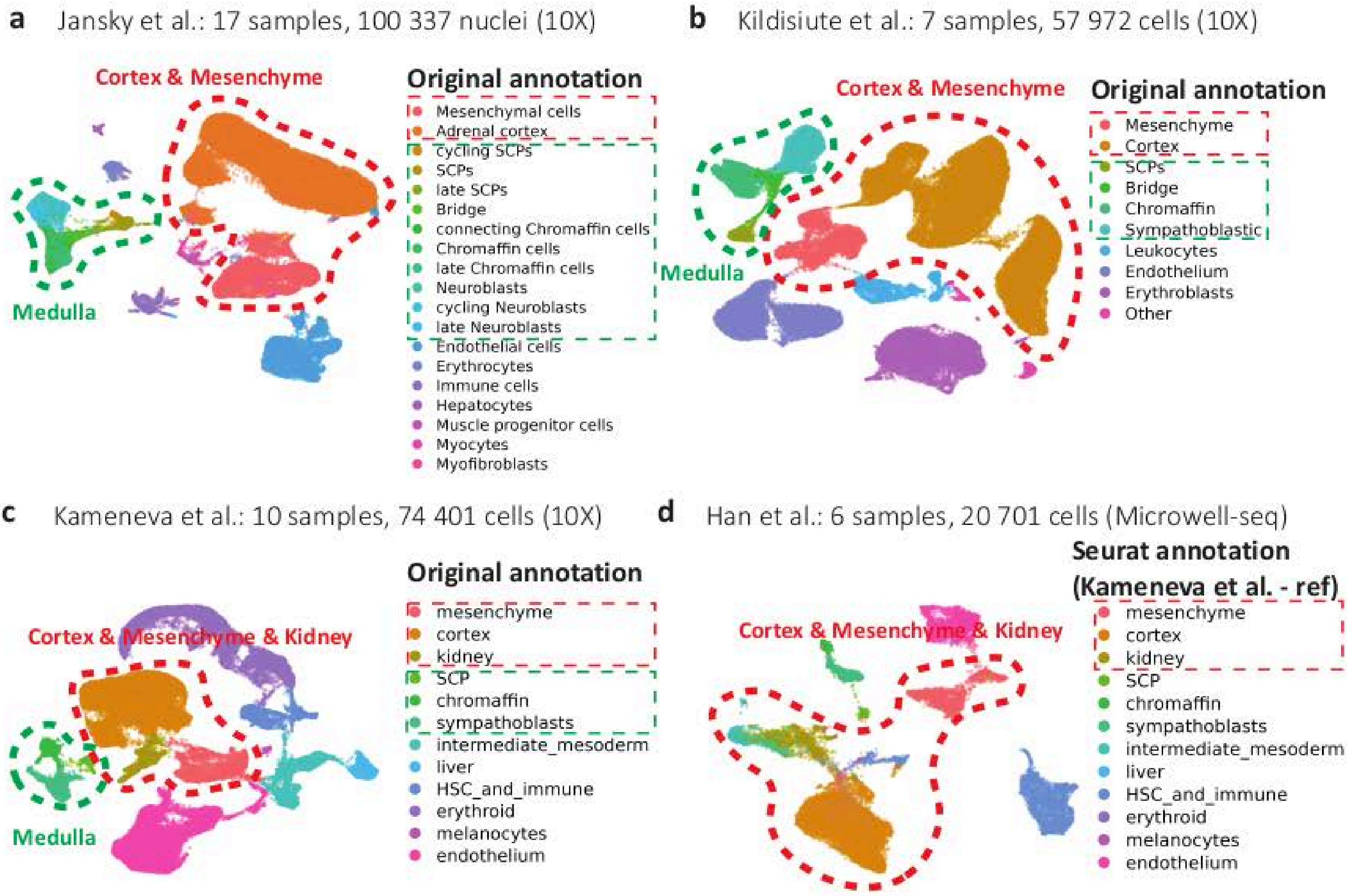

For Kameneva et al. and Han et al. iterative *Harmony* integration to exclude *PAX2+* kidney cells

Medulla - samples with > 100 target cells/nuclei integrated (Seurat), cluster with high *STAR* expression (471 cells) was excluded, the remaining cells reintegrated

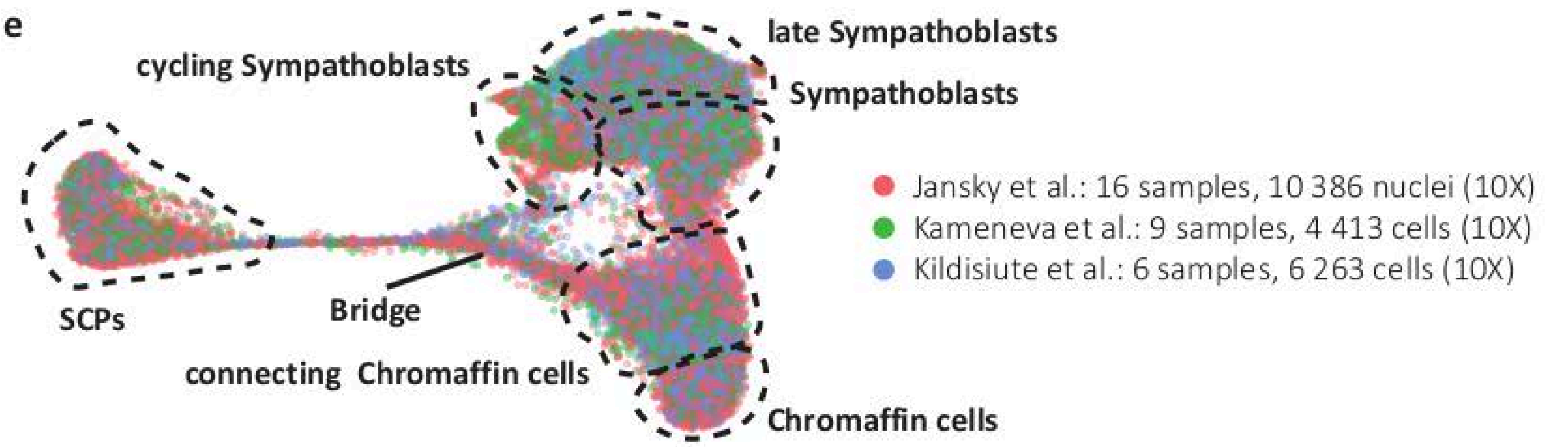

Cortex & Mesenchyme - samples with > 800 target cells/nuclei integrated (Seurat)

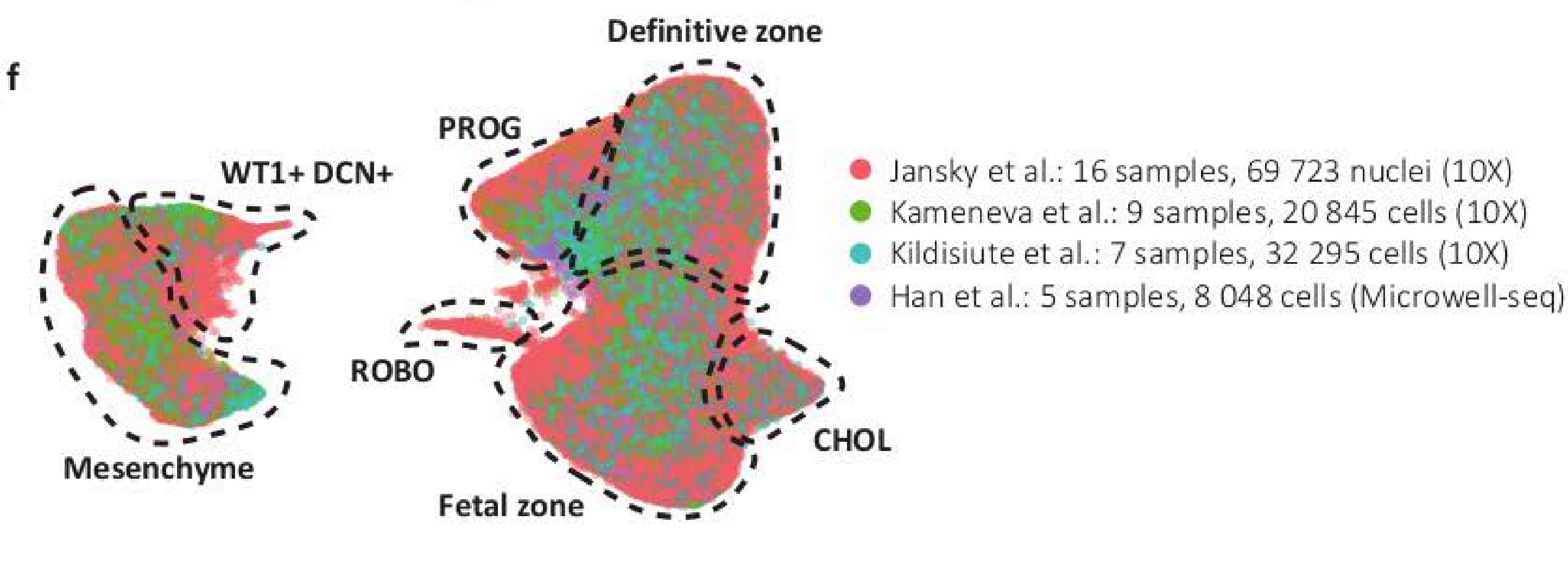

Supplementary Fig. 34 | Human fetal adrenal gland scRNA-seq data integration. a-d, Sources of scRNA-seq and snRNA-seq data for adrenal gland fetal development, with UMAP embeddings and cell type annotations for each dataset. The dotted lines highlight the cell types included in the integration and their position on the UMAP. The red line indicates cells included in the adrenocortical and mesenchymal cell integration, while the green line highlights cells for medullary integration. **a**, Jansky et al. dataset, original article cell type annotation and Harmony integration-based UMAP. **b**, Kildisiute et al. dataset, original UMAP and cell type annotation. **c**, Kameneva et al. dataset, original UMAP and annotation. **d**, Han et al. dataset with Harmony integration-based UMAP and annotation based on Seurat label transfer from Kameneva et al. as a reference dataset. **e-f**, Integrations, with dot colors indicating the compositional contribution of each dataset and black dotted lines highlighting key cell type groups. **e**, Fetal medulla integration UMAP (21,062 cells). **f**, Fetal adrenocortical and mesenchymal cell integration UMAP (130,911 cells).

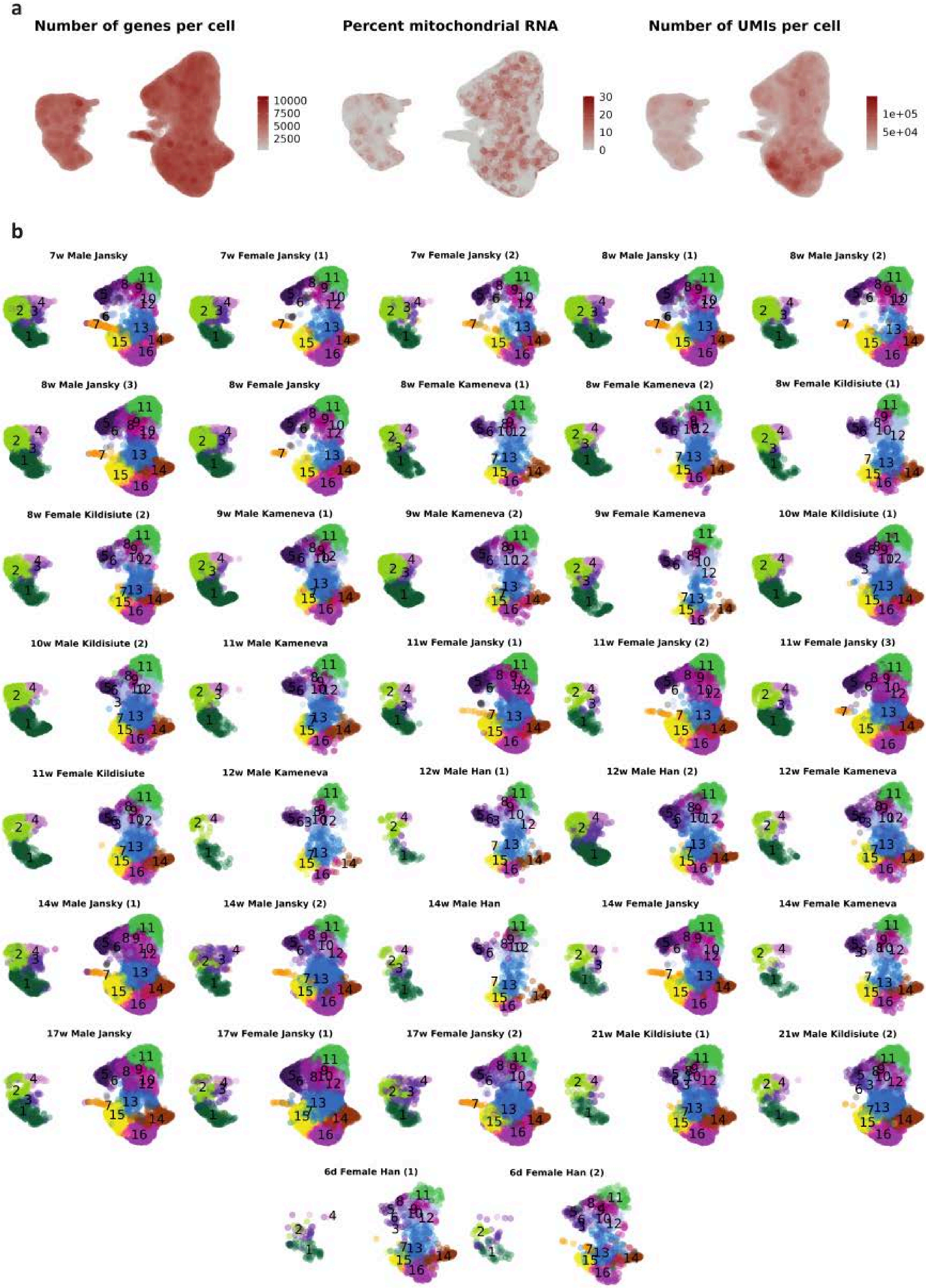
a
Number of genes per cell
10000
7500
5000
2500
Percent mitochondrial RNA
30
20
10
0
Number of UMIs per cell
1e+05
5e+04
b
7w Male Jansky
7w Female Jansky (1)
7w Female Jansky (2)
8w Male Jansky (1)
8w Male Jansky (2)
8w Male Jansky (3)
8w Female Jansky
8w Female Kameneva (1)
8w Female Kameneva (2)
8w Female Kildisiute (1)
8w Female Kildisiute (2)
9w Male Kameneva (1)
9w Male Kameneva (2)
9w Female Kameneva
10w Male Kildisiute (1)
10w Male Kildisiute (2)
11w Male Kameneva
11w Female Jansky (1)
11w Female Jansky (2)
11w Female Jansky (3)
11w Female Kildisiute
12w Male Kameneva
12w Male Han (1)
12w Male Han (2)
12w Female Kameneva
14w Male Jansky (1)
14w Male Jansky (2)
14w Male Han
14w Female Jansky
14w Female Kameneva
17w Male Jansky
17w Female Jansky (1)
17w Female Jansky (2)
21w Male Kildisiute (1)
21w Male Kildisiute (2)
6d Female Han (1)
6d Female Han (2)

Supplementary Fig. 35 | QC metrics and per sample compositional impact of human fetal adrenal cortex. a, Number of UMIs per cell, Number of genes per cell and Percent mitochondrial RNA on the fetal adrenocortical and mesenchymal cells embedding (130,911 cells). **b**, Per sample compositional impact - UMAP embedding subset of cells from each individual fetal human sample.

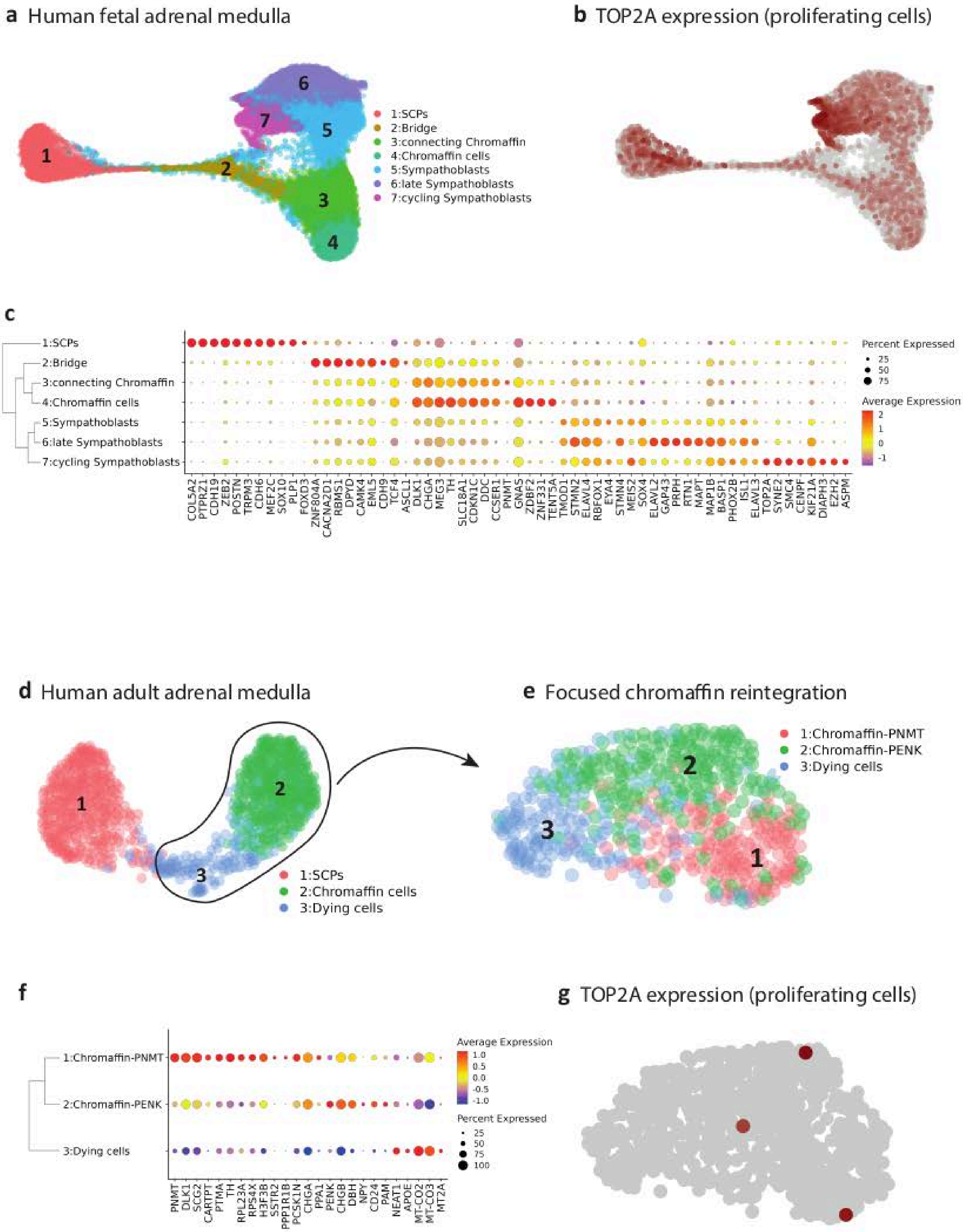

a Human fetal adrenal medulla
1:SCPs
2:Bridge
3:connecting Chromaffin
4:Chromaffin cells
5:Sympathoblasts
6:late Sympathoblasts
7:cycling Sympathoblasts
b TOP2A expression (proliferating cells)
c
Percent Expressed
25
50
75
Average Expression
2
1
0
-1
COL5A2 PTPRZ1 CDH19 ZEB2 POSTN TRPM3 CDH6 MEF2C SOX10 PLP1 FOXD3 ZNF804A CACNA2D1 RBMS1 DPYD CAMK4 EML5 CDH9 TCF4 ASCL1 DLK1 CHGA MEG3 TH SLC18A1 CDKN1C DDC CCSER1 PNMT GNAS ZDBF2 ZNF331 TENT5A TMOD1 STMN2 ELAVL4 RBFOX1 EYA4 STMN4 MEIS2 SOX4 ELAVL2 GAP43 PRPH RTN1 MAPT MAP1B BASP1 PHOX2B ISL1 ELAVL3 TOP2A SYNE2 SMC4 CENPF KIF21A DIAPH3 EZH2 ASPM
d Human adult adrenal medulla
1:SCPs
2:Chromaffin cells
3:Dying cells
e Focused chromaffin reintegration
1:Chromaffin-PNMT
2:Chromaffin-PENK
3:Dying cells
f
Average Expression
1.0
0.5
0.0
-0.5
-1.0
Percent Expressed
25
50
75
100
PNMT DLK1 SCG2 CARTPT PTMA TH RPL23A RPS4X H3F3B SSTR2 PPP1R1B PCSK1N CHGA PPA1 PENK CHGB DBH NPY CD24 PAM NEAT1 APOE MT-CO2 MT-CO3 MT2A
g TOP2A expression (proliferating cells)

Supplementary Fig. 36 | Transcriptional heterogeneity of the human fetal adrenal medulla and adult chromaffin cells. a-c, Human fetal adrenal medulla (21,062 cells）. **d-g**, Human adult adrenal medulla (1,082 cells) and chromaffin (558 cells) cells. **a,d,e**, A UMAP embedding resulting from the focused reintegration of target adrenal cell population. **b,g**, *TOP2A* gene expression indicates location of proliferating cells on UMAP. **c,f**, DotPlot representation of marker genes for subpopulations of target adrenal cell population. The hierarchy of clusters, based on transcriptional similarity, is shown on the left.

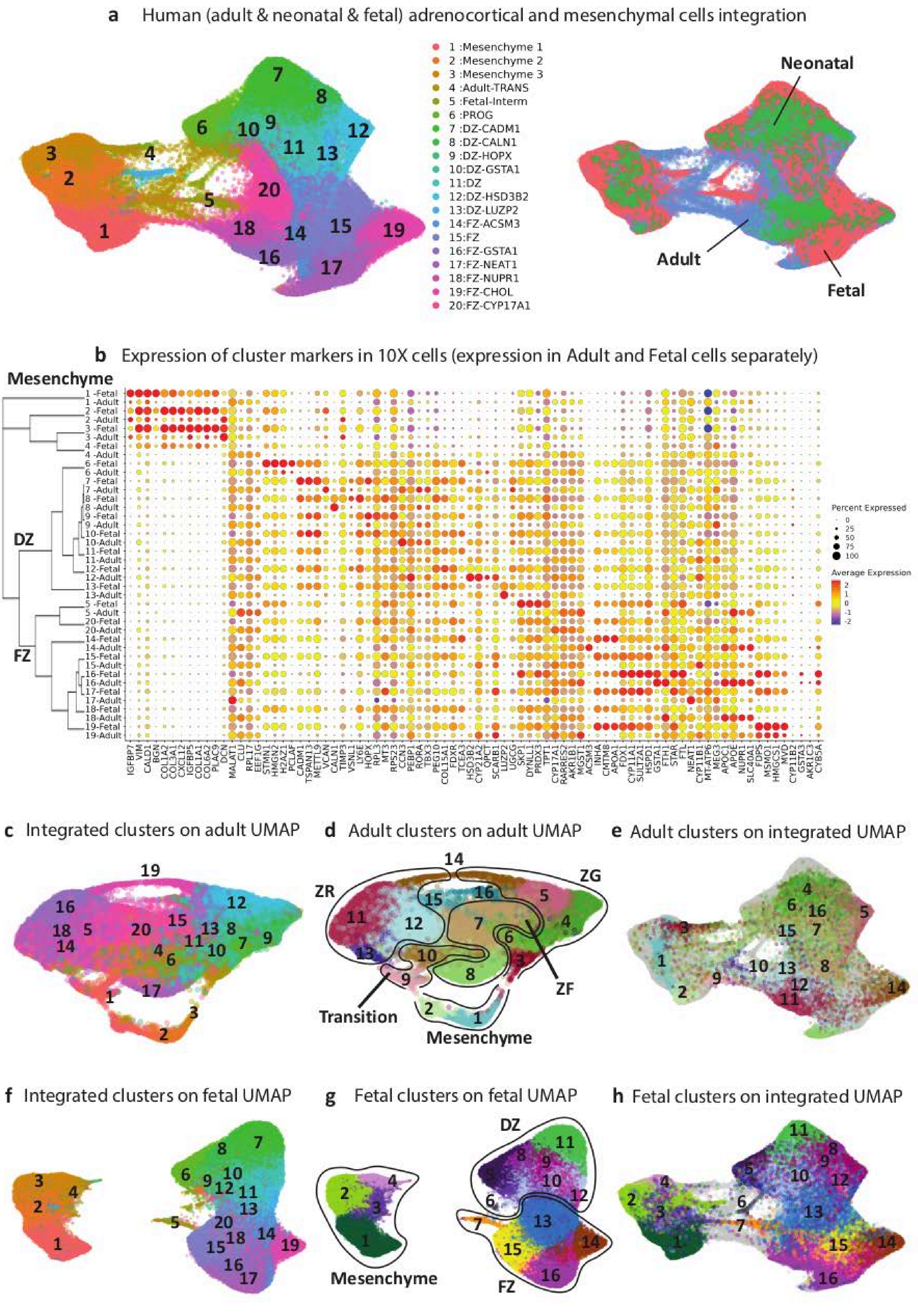
a Human (adult & neonatal & fetal) adrenocortical and mesenchymal cells integration
1 :Mesenchyme 1
2 :Mesenchyme 2
3 :Mesenchyme 3
4 :Adult-TRANS
5 :Fetal-Interm
6 :PROG
7 :DZ-CADM1
8 :DZ-CALN1
9 :DZ-HOPX
10:DZ-GSTA1
11:DZ
12:DZ-HSD3B2
13:DZ-LUZP2
14:FZ-ACSM3
15:FZ
16:FZ-GSTA1
17:FZ-NEAT1
18:FZ-NUPR1
19:FZ-CHOL
20:FZ-CYP17A1
Neonatal
Adult
Fetal
b Expression of cluster markers in 10X cells (expression in Adult and Fetal cells separately)
Mesenchyme
DZ
FZ
Percent Expressed
Average Expression
c Integrated clusters on adult UMAP
d Adult clusters on adult UMAP
ZR
ZG
ZF
Transition
Mesenchyme
e Adult clusters on integrated UMAP
f Integrated clusters on fetal UMAP
g Fetal clusters on fetal UMAP
DZ
FZ
Mesenchyme
h Fetal clusters on integrated UMAP

Supplementary Fig. 37 | Human adult, neonatal and fetal adrenocortical and mesenchymal cells integration. a, A UMAP embedding resulting from the integration of human adult (42,580 cells), neonatal (2,677 cells) and fetal (128,234 cells) adrenocortical and mesenchymal cells (total 173,491 cells). Left - integrated clusterization, right - compositional impact of adult (blue), fetal (red) and neonatal (green) data. **b**, DotPlot displaying cluster-specific marker genes and key steroidogenic enzymes. The hierarchy of clusters, based on transcriptional similarity, is shown on the left. For each cluster gene expression is shown for adult and fetal (10X cells only) separately. **c-h**, Comparison of adult or fetal specific UMAPs and clusterization with integrated ones. **c**, Integrated clusters on adult UMAP. **d**, Adult clusters on adult UMAP. **e**, Adult clusters on integrated UMAP. **f**, Integrated clusters on fetal UMAP. **g**, Fetal clusters on fetal UMAP. **h**, Fetal clusters on integrated UMAP.

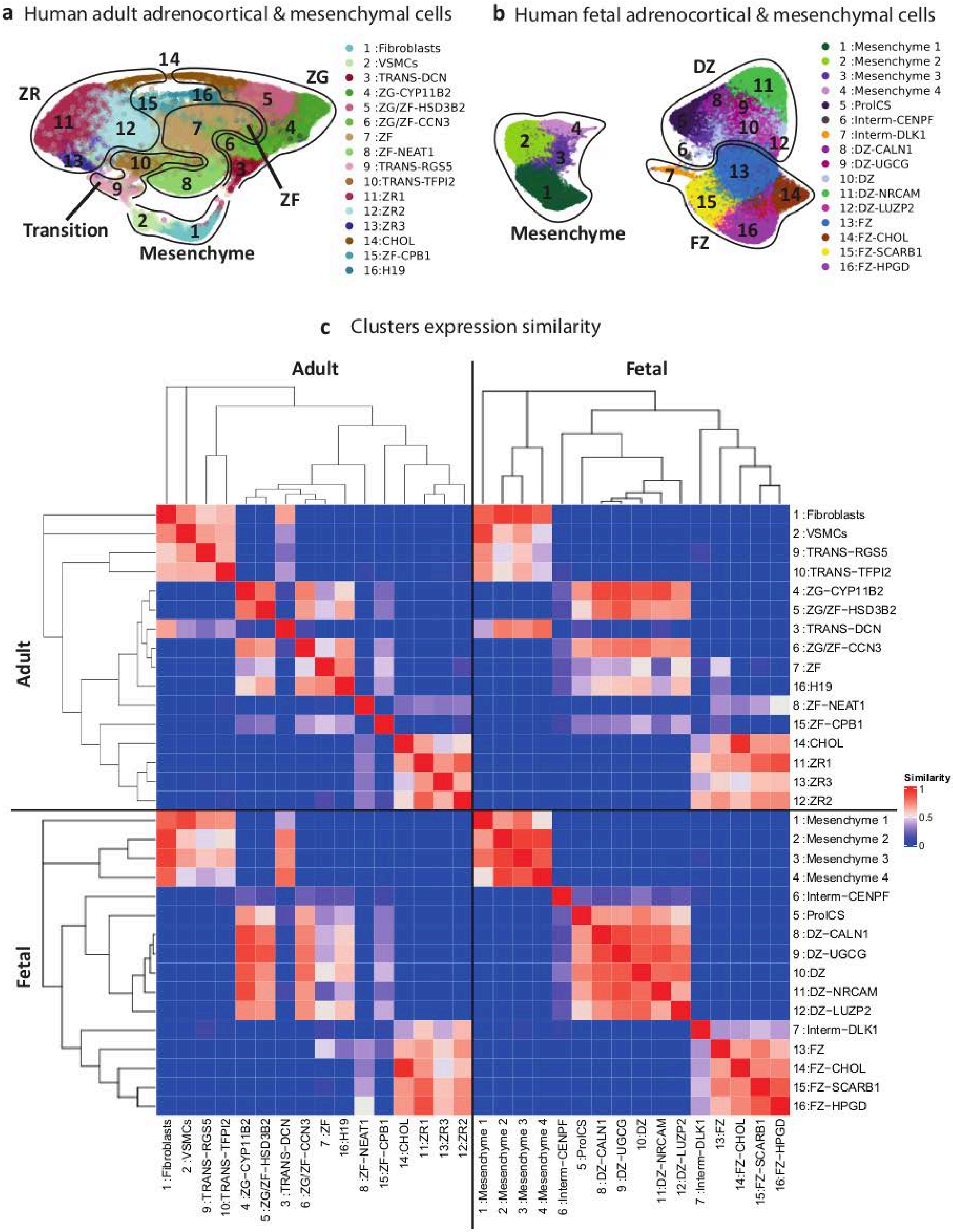
a Human adult adrenocortical & mesenchymal cells
ZR
14
ZG
ZF
Transition
Mesenchyme
1 :Fibroblasts
2 :VSMCs
3 :TRANS-DCN
4 :ZG-CYP11B2
5 :ZG/ZF-HSD3B2
6 :ZG/ZF-CCN3
7 :ZF
8 :ZF-NEAT1
9 :TRANS-RGS5
10:TRANS-TFPI2
11:ZR1
12:ZR2
13:ZR3
14:CHOL
15:ZF-CPB1
16:H19
b Human fetal adrenocortical & mesenchymal cells
DZ
FZ
Mesenchyme
1 :Mesenchyme 1
2 :Mesenchyme 2
3 :Mesenchyme 3
4 :Mesenchyme 4
5 :ProlCS
6 :Interm-CENPF
7 :Interm-DLK1
8 :DZ-CALN1
9 :DZ-UGCG
10:DZ
11:DZ-NRCAM
12:DZ-LUZP2
13:FZ
14:FZ-CHOL
15:FZ-SCARB1
16:FZ-HPGD
c Clusters expression similarity
Adult
Fetal
Similarity
1
0.5
0
1 :Fibroblasts
2 :VSMCs
9 :TRANS-RGS5
10:TRANS-TFPI2
4 :ZG-CYP11B2
5 :ZG/ZF-HSD3B2
3 :TRANS-DCN
6 :ZG/ZF-CCN3
7 :ZF
16:H19
8 :ZF-NEAT1
15:ZF-CPB1
14:CHOL
11:ZR1
13:ZR3
12:ZR2
1 :Mesenchyme 1
2 :Mesenchyme 2
3 :Mesenchyme 3
4 :Mesenchyme 4
6 :Interm-CENPF
5 :ProlCS
8 :DZ-CALN1
9 :DZ-UGCG
10:DZ
11:DZ-NRCAM
12:DZ-LUZP2
7 :Interm-DLK1
13:FZ
14:FZ-CHOL
15:FZ-SCARB1
16:FZ-HPGD

Supplementary Fig. 38 | Human adult and fetal adrenocortical and mesenchymal cell clusters comparison. a, Human adult adrenocortical and mesenchymal cells embedding (39,985 cells). **b**, Human fetal adrenocortical and mesenchymal cells embedding (173,491 cells). **c**, Expression similarity between clusters was calculated as a pearson correlation (clipped at value 0) between cell average batch corrected variable gene expression vectors and visualized as a heatmap. The original hierarchies of adult and fetal clusters, based on transcriptional similarity, are shown on the left and above. Black lines separate adult and fetal clusters.

a

	7w	8w	9w	10w	11w	12w	14w	17w	21w	6d
Jansky et al. (nuclei)	2F 1M	1F 3M	-	-	3F 0M	-	1F 2M	2F 1M	-	-
Kameneva et al. (cells)	-	2F 0M	1F 2M	-	0F 1M	1F 1M	1F 0M	-	-	-
Kildisiute et al. (cells)	-	2F 0M	-	0F 2M	1F 0M	-	-	-	0F 2M	-
Han et al. (Microwell)	-	-	-	-	-	-	0F 2M	-	0F 2M	2F 0M

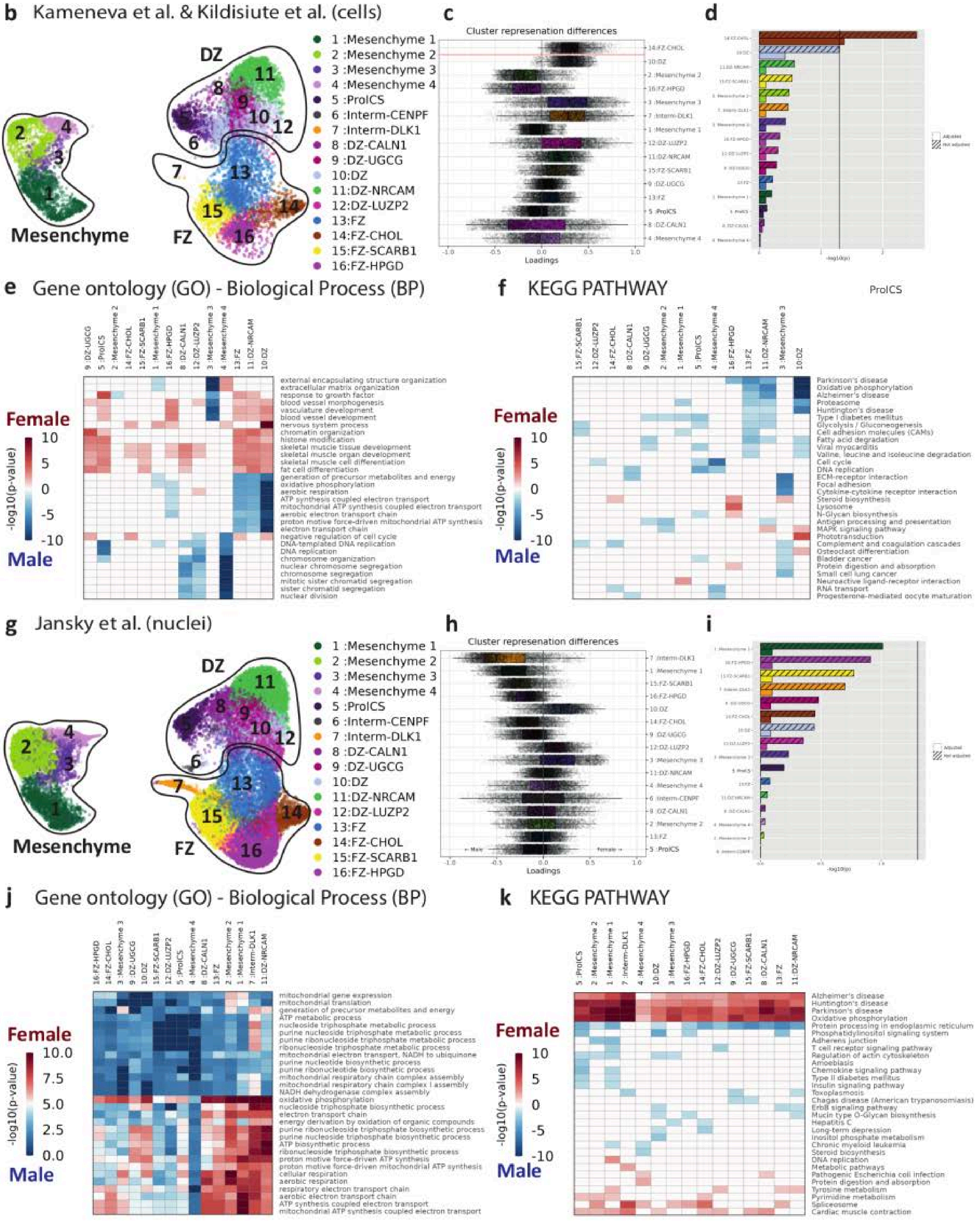

Supplementary Fig. 39 | Sexual dimorphism of the human fetal adrenal cortex. a, Human fetal adrenocortical scRNA-seq samples in analysis. Columns - time points, w - gestation weeks, d - days after birth. Rows - data sources. In table cells the number of samples of this time point in analysis, F - female samples, M - male samples. To analyze sexual dimorphism, groups of males and females must contain the same set of time points, and all samples must be obtained from the same scRNA-seq platform (here 10X cells or 10X nuclei). In red highlighted samples, limiting inclusion of samples of this time point. As a result, for sexual dimorphism analysis one male and one female sample was taken for each time point: for 10X cells - 9w, 11w, 12w (6 samples in total); for 10X nuclei - 7w, 8w, 14w, 17w (8 samples in total). **b-f**, Sexual dimorphism analysis in 10X cell samples (Kameneva et al. and Kildisiute et al. data sources; 12,849 cells). All results were obtained using cacoa R package, see Methods section. **g-k**, Sexual dimorphism analysis in 10X nuclei samples (Jansky et al. data sources; 30,692 cells). All results were obtained using cacoa R package, see Methods section. **b**,**g**, Subset of cells in analysis on UMAP embedding. **c-d, h-i,** Cluster based analysis of compositional differences. **c,h,** Estimated loadings for cluster representation differences. Each dot corresponds to the permutation iteration (n.boot=5000). Positive loading values indicate cluster overrepresentation in females, negative - in males. For each box-plot, center line, median; box limits, upper and lower quartiles; whiskers, 1.5x interquartile range. **d,i,** Statistical significance of observed cluster representation differences. Dashed bars correspond to p-values before adjusting for multiple hypothesis testing, not dashed - after correction. The vertical line is set at the significance level p-value = 0.05. **e-f, j-k,** Gene set enrichment analysis of differential expression between males and females in each cluster. Columns indicate clusters, rows - pathway names. Colors indicate statistical significance, red - overexpressed in females, blue - in males. **e**,**j**, Gene ontology (GO), Biological Process (BP). **f,k,** Kyoto Encyclopedia of Genes and Genomes (KEGG PATHWAY).

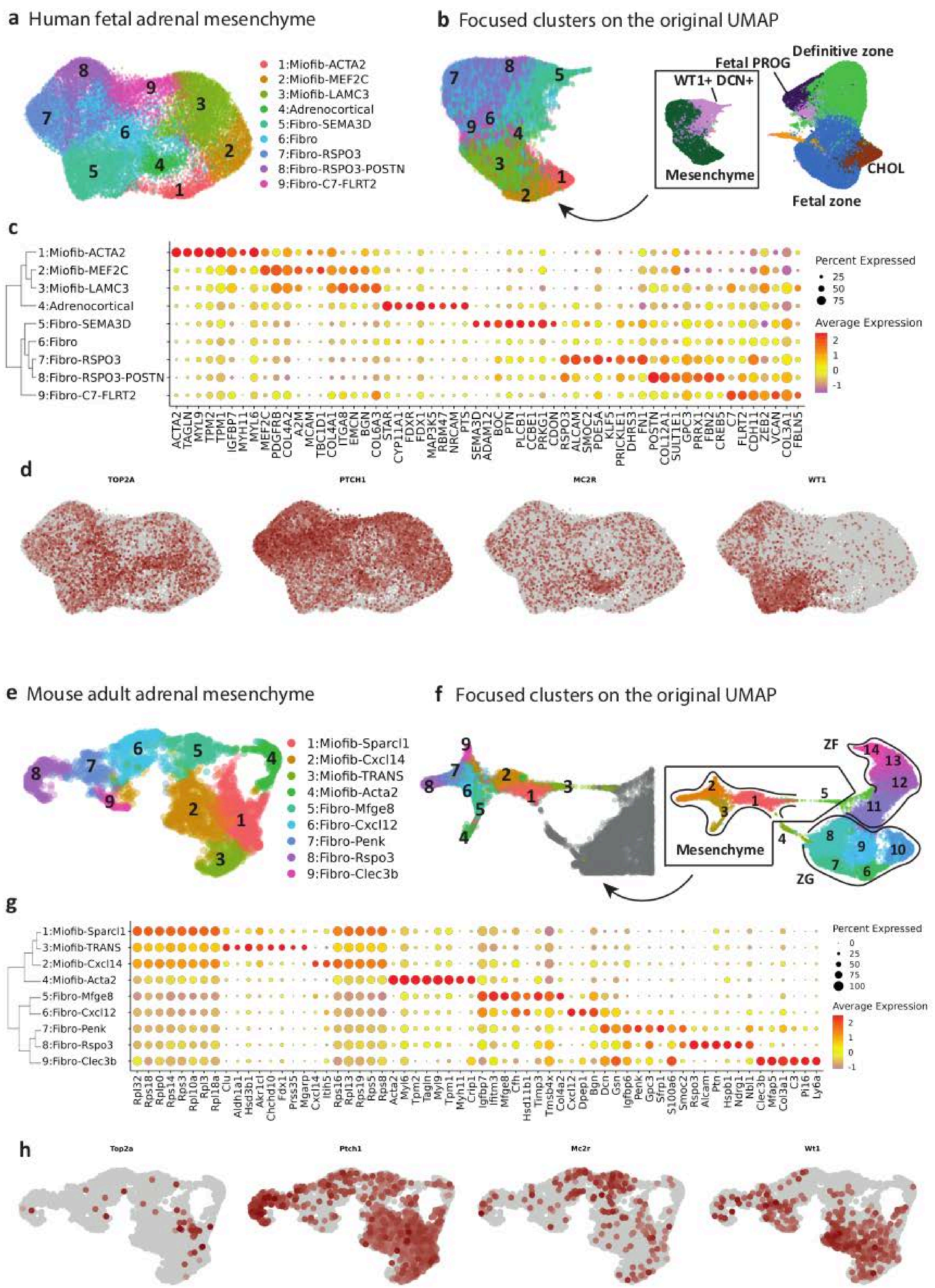
a Human fetal adrenal mesenchyme
1:Miofib-ACTA2
2:Miofib-MEF2C
3:Miofib-LAMC3
4:Adrenocortical
5:Fibro-SEMA3D
6:Fibro
7:Fibro-RSPO3
8:Fibro-RSPO3-POSTN
9:Fibro-C7-FLRT2
b Focused clusters on the original UMAP
WT1+ DCN+
Mesenchyme
Fetal PROG
Definitive zone
CHOL
Fetal zone
c
Percent Expressed
25
50
75
Average Expression
2
1
0
-1
ACTA2 TAGLN MYL9 TPM2 TPM1 IGFBP7 MYH11 MYL6 MEF2C PDGFRB COL4A2 A2M MCAM TBC1D1 COL4A1 ITGA8 EMCN BGN COL6A3 STAR CYP11A1 FDXR FDX1 MAP3K5 RBM47 NRCAM PTS SEMA3D ADAM12 BOC PTN PLCB1 CCBE1 PRKG1 CDON RSPO3 ALCAM SMOC2 PDE5A KLF5 PRICKLE1 DHRS3 FN1 POSTN COL12A1 SULT1E1 GPC3 PRRX1 FBN2 CREB5 C7 FLRT2 CDH11 ZEB2 VCAN COL3A1 FBLN5
d
TOP2A
PTCH1
MC2R
WT1
e Mouse adult adrenal mesenchyme
1:Miofib-Sparcl1
2:Miofib-Cxcl14
3:Miofib-TRANS
4:Miofib-Acta2
5:Fibro-Mfge8
6:Fibro-Cxcl12
7:Fibro-Penk
8:Fibro-Rspo3
9:Fibro-Clec3b
f Focused clusters on the original UMAP
Mesenchyme
ZF
ZG
g
Percent Expressed
0
25
50
75
100
Average Expression
2
1
0
-1
Rpl32 Rps18 Rplp0 Rps14 Rps3 Rpl10a Rpl3 Rpl18a Clu Aldh1a1 Hsd3b1 Akr1cl Chchd10 Fdx1 Prss35 Mgarp Cxcl14 Itih5 Rps16 Rpl13 Rps19 Rps5 Rps8 Acta2 Myl6 Tpm2 Tagln Myl9 Tpm1 Myh11 Crip1 Igfbp7 Ifitm3 Mfge8 Cfh Hsd11b1 Timp3 Tmsb4x Col4a2 Cxcl12 Dpep1 Bgn Dcn Gsn Igfbp6 Penk Gpc3 Sfrp1 S100a6 Smoc2 Rspo3 Alcam Ptn Hspb1 Ndrg1 Nbl1 Clec3b Mfap5 Col3a1 C3 Pi16 Ly6a
h
Top2a
Ptch1
Mc2r
Wt1

Supplementary Fig. 40 | Transcriptional heterogeneity of mesenchymal subpopulations in human fetal and mouse adult adrenal glands. a-d, Human fetal adrenal mesenchymal cells (including nuclei) (26,325 cells). **e-h**, Mouse adult adrenal mesenchymal cells (3,560 cells). **a,e**, A UMAP embedding resulting from the focused reintegration of adrenal mesenchymal cells. **b,f**, Mapping of focused mesenchymal integration clusters to adrenocortical-mesenchymal UMAP. **c,g**, DotPlot representation of marker genes for subpopulations of adrenal mesenchymal cells. The hierarchy of clusters, based on transcriptional similarity, is shown on the left. **d,h**, Expression of *TOP2A* (a proliferation marker), *PTCH1* (a marker of hedgehog pathway-regulated stem cells), *MC2R* (adrenocorticotropic hormone receptor), *WT1* (a marker of human adrenogenic coelomic epithelium and mouse capsular stem cell population involved in adrenal reparation) in adrenal mesenchymal cells.

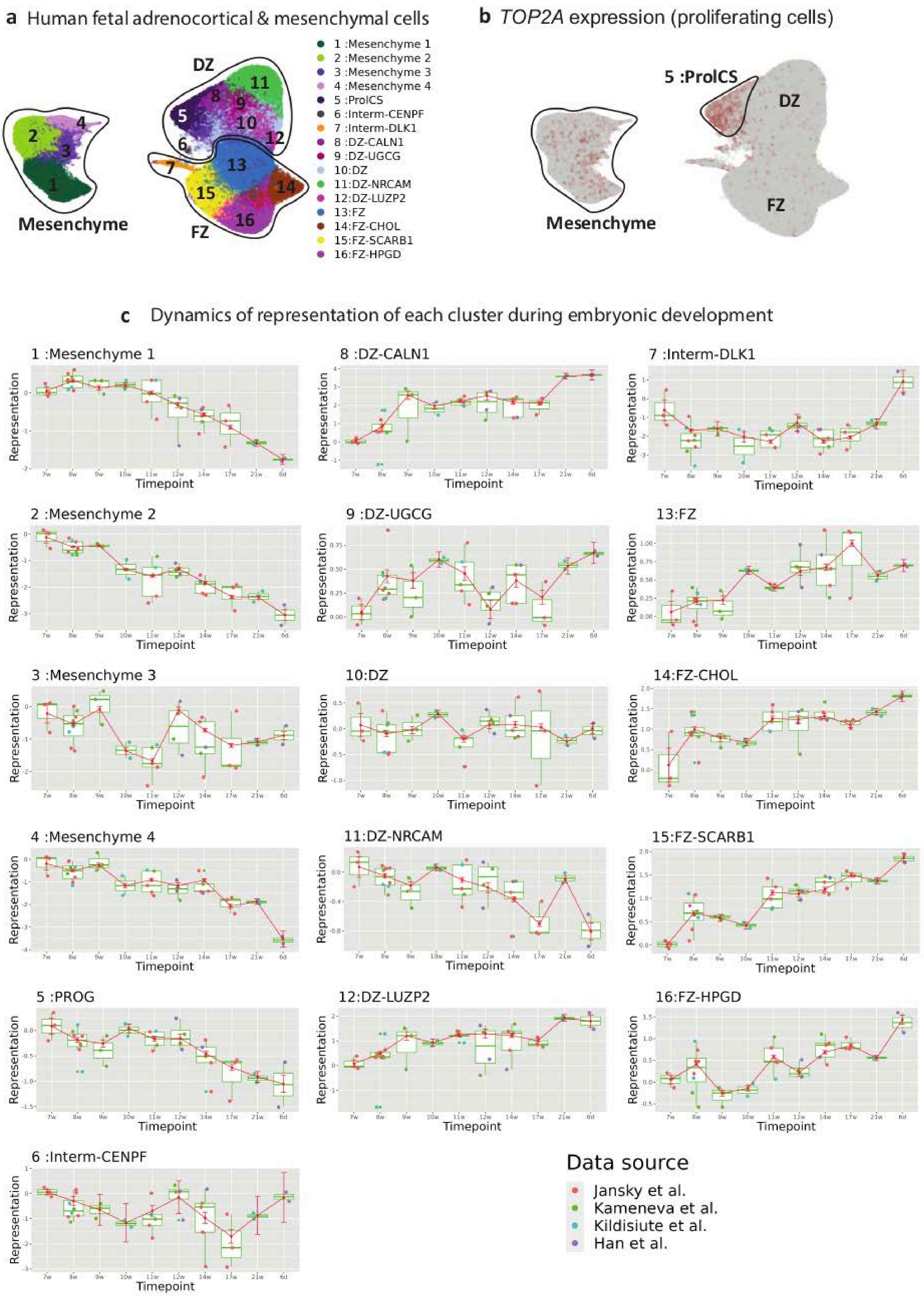
a Human fetal adrenocortical & mesenchymal cells
DZ
Mesenchyme
FZ
1 :Mesenchyme 1
2 :Mesenchyme 2
3 :Mesenchyme 3
4 :Mesenchyme 4
5 :ProlCS
6 :Interm-CENPF
7 :Interm-DLK1
8 :DZ-CALN1
9 :DZ-UGCG
10:DZ
11:DZ-NRCAM
12:DZ-LUZP2
13:FZ
14:FZ-CHOL
15:FZ-SCARB1
16:FZ-HPGD
b TOP2A expression (proliferating cells)
5 :ProlCS
DZ
Mesenchyme
FZ
c Dynamics of representation of each cluster during embryonic development
1 :Mesenchyme 1
8 :DZ-CALN1
7 :Interm-DLK1
2 :Mesenchyme 2
9 :DZ-UGCG
13:FZ
3 :Mesenchyme 3
10:DZ
14:FZ-CHOL
4 :Mesenchyme 4
11:DZ-NRCAM
15:FZ-SCARB1
5 :PROG
12:DZ-LUZP2
16:FZ-HPGD
6 :Interm-CENPF
Representation
Timepoint
7w 8w 9w 10w 11w 12w 14w 17w 21w 6d
Data source
Jansky et al.
Kameneva et al.
Kildisiute et al.
Han et al.

Supplementary Fig. 41 | Compositional representation dynamics of human fetal adrenal cortex clusters. **a**, Human fetal adrenocortical and mesenchymal cells embedding (130,911 cells). **b**, *TOP2A* gene expression indicates location of proliferating cells on UMAP. **c**, Dynamics of representation of each cluster during embryonic development (37 samples). One plot for each cluster, for time point w - gestation weeks, d - days after birth, dot colors indicate data source. For each box-plot, center line, median; box limits, upper and lower quartiles; whiskers, 1.5x interquartile range; points, outliers. Read dots and error bars, mean and SE of cluster representation for each time point, estimated from the Poisson linear mixture model (see Methods section).

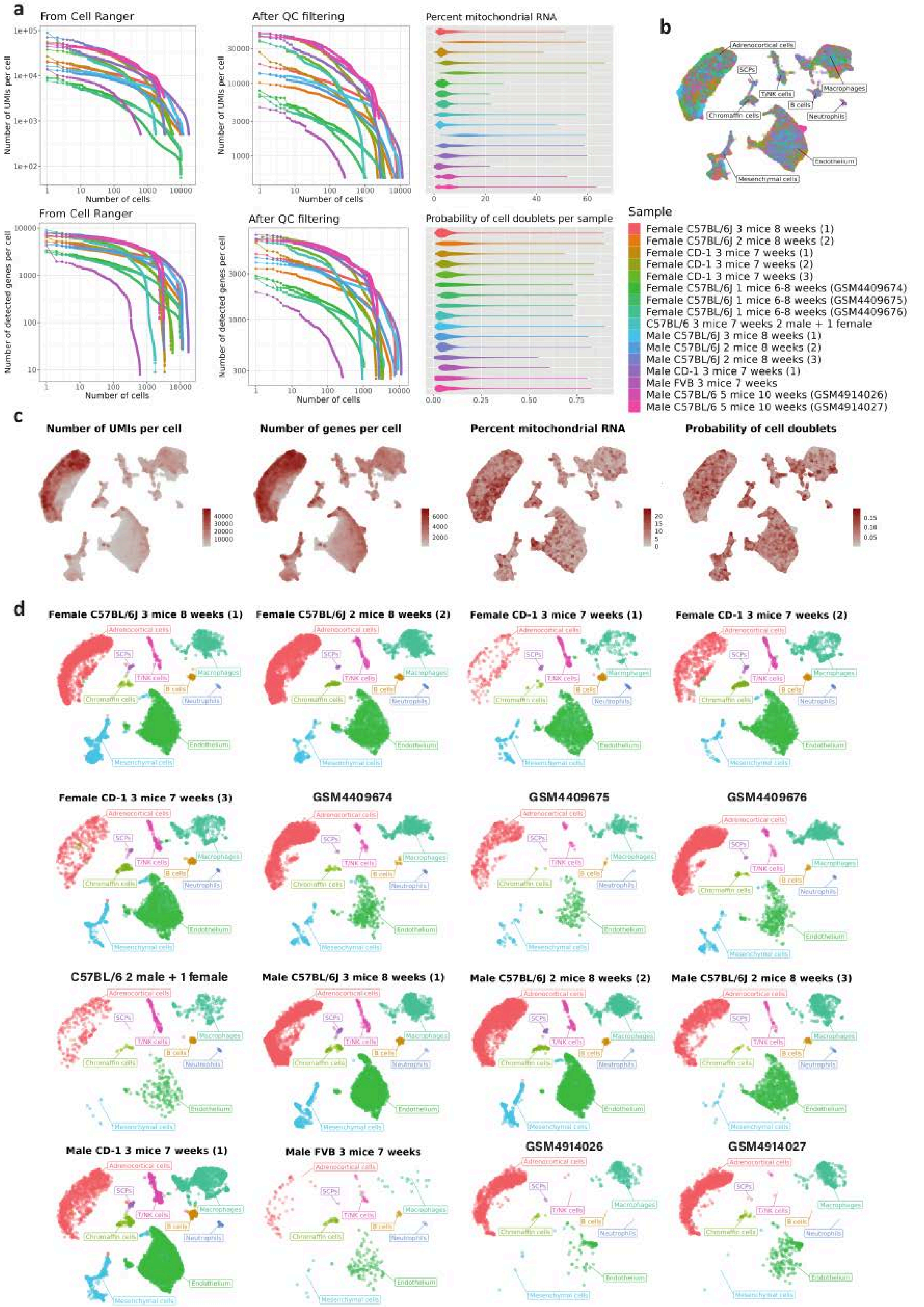
a
From Cell Ranger
Number of UMIs per cell
Number of cells
After QC filtering
Percent mitochondrial RNA
Number of detected genes per cell
Probability of cell doublets per sample
b
Adrenocortical cells
SCPs
T/NK cells
Macrophages
B cells
Chromaffin cells
Neutrophils
Endothelium
Mesenchymal cells
Sample
Female C57BL/6J 3 mice 8 weeks (1)
Female C57BL/6J 2 mice 8 weeks (2)
Female CD-1 3 mice 7 weeks (1)
Female CD-1 3 mice 7 weeks (2)
Female CD-1 3 mice 7 weeks (3)
Female C57BL/6J 1 mice 6-8 weeks (GSM4409674)
Female C57BL/6J 1 mice 6-8 weeks (GSM4409675)
Female C57BL/6J 1 mice 6-8 weeks (GSM4409676)
C57BL/6 3 mice 7 weeks 2 male + 1 female
Male C57BL/6J 3 mice 8 weeks (1)
Male C57BL/6J 2 mice 8 weeks (2)
Male C57BL/6J 2 mice 8 weeks (3)
Male CD-1 3 mice 7 weeks (1)
Male FVB 3 mice 7 weeks
Male C57BL/6 5 mice 10 weeks (GSM4914026)
Male C57BL/6 5 mice 10 weeks (GSM4914027)
c
Number of UMIs per cell
Number of genes per cell
Percent mitochondrial RNA
Probability of cell doublets
d
Female C57BL/6J 3 mice 8 weeks (1)
Female C57BL/6J 2 mice 8 weeks (2)
Female CD-1 3 mice 7 weeks (1)
Female CD-1 3 mice 7 weeks (2)
Female CD-1 3 mice 7 weeks (3)
GSM4409674
GSM4409675
GSM4409676
C57BL/6 2 male + 1 female
Male C57BL/6J 3 mice 8 weeks (1)
Male C57BL/6J 2 mice 8 weeks (2)
Male C57BL/6J 2 mice 8 weeks (3)
Male CD-1 3 mice 7 weeks (1)
Male FVB 3 mice 7 weeks
GSM4914026
GSM4914027

Supplementary Fig. 42 | Technical quality controls for scRNA-seq data of mouse adult adrenal gland. a, Numbers of detected genes per cell (upper panel) and numbers of UMIs per cell (lower panel) in each sample before (From Cell Ranger) and after QC filtering (After QC filtering). Fraction of mitochondrial RNA (Percent mitochondrial RNA) and Scrublet score (Probability of cell doublets per sample) distributions in each sample. The color legend is consistent across all plots. **b**, Per sample compositional impact on the general UMAP embedding (67,665 cells). **c**, Number of UMIs per cell, Number of genes per cell, Percent mitochondrial RNA and Probability of cell doublets (Scrublet scores) after all QC filtrations and ambient RNA correction on the general UMAP embedding. **d**, UMAP embedding subset of cells from each individual human sample.

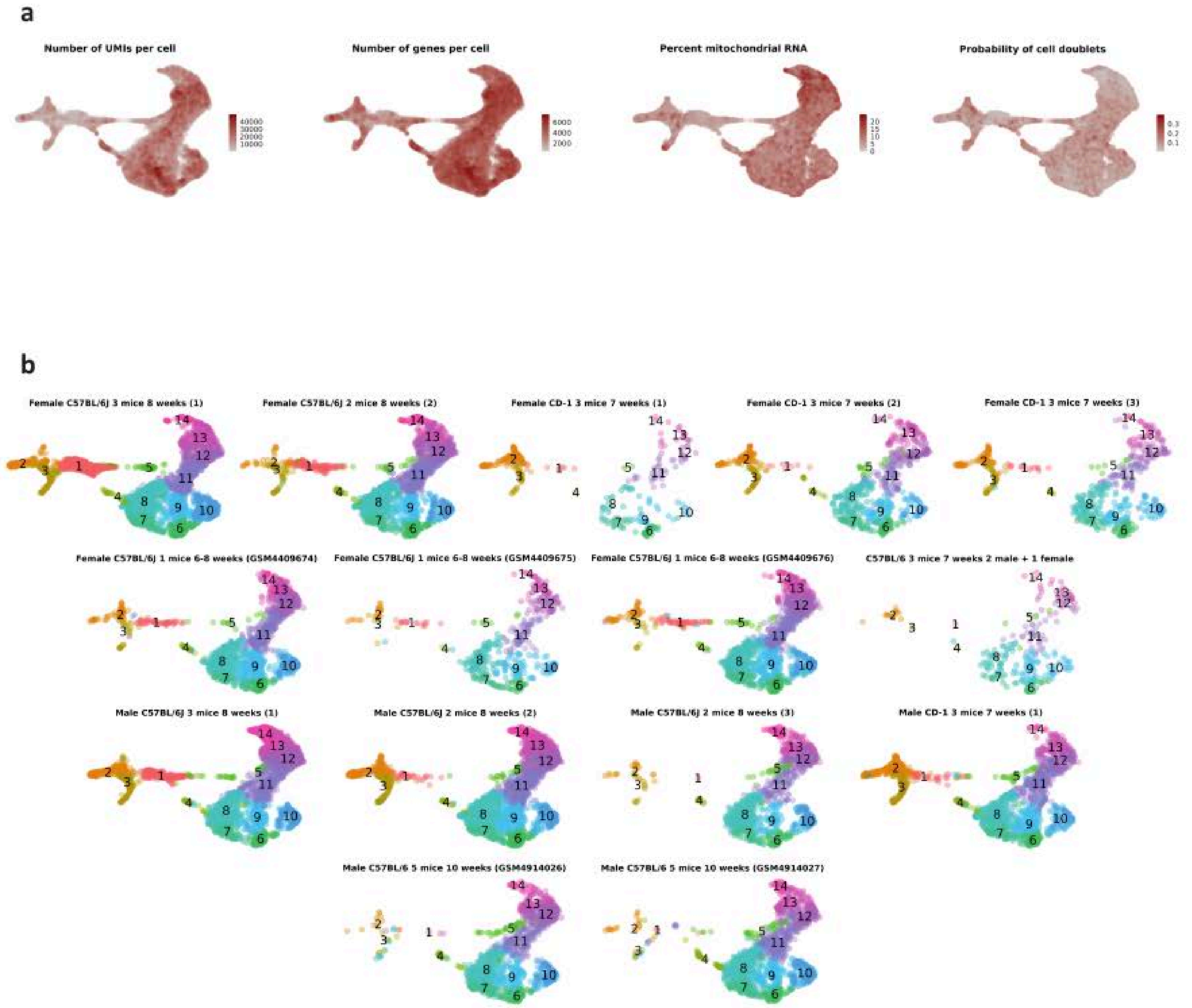

a
Number of UMIs per cell
40000
30000
20000
10000
Number of genes per cell
6000
4000
2000
Percent mitochondrial RNA
20
15
10
5
0
Probability of cell doublets
0.3
0.2
0.1
b
Female C57BL/6J 3 mice 8 weeks (1)
Female C57BL/6J 2 mice 8 weeks (2)
Female CD-1 3 mice 7 weeks (1)
Female CD-1 3 mice 7 weeks (2)
Female CD-1 3 mice 7 weeks (3)
Female C57BL/6J 1 mice 6-8 weeks (GSM4409674)
Female C57BL/6J 1 mice 6-8 weeks (GSM4409675)
Female C57BL/6J 1 mice 6-8 weeks (GSM4409676)
C57BL/6 3 mice 7 weeks 2 male + 1 female
Male C57BL/6J 3 mice 8 weeks (1)
Male C57BL/6J 2 mice 8 weeks (2)
Male C57BL/6J 2 mice 8 weeks (3)
Male CD-1 3 mice 7 weeks (1)
Male C57BL/6 5 mice 10 weeks (GSM4914026)
Male C57BL/6 5 mice 10 weeks (GSM4914027)

Supplementary Fig. 43 | QC metrics and per sample compositional impact of mouse adult adrenal cortex. a, Number of UMIs per cell, Number of genes per cell, Percent mitochondrial RNA and Probability of cell doublets (Scrublet scores) on the adrenocortical and mesenchymal cells embedding (25,932 cells). **b**, Per sample compositional impact - UMAP embedding subset of cells from each individual mouse sample. Subsets are shown for general adrenocortical and mesenchymal cells embedding.

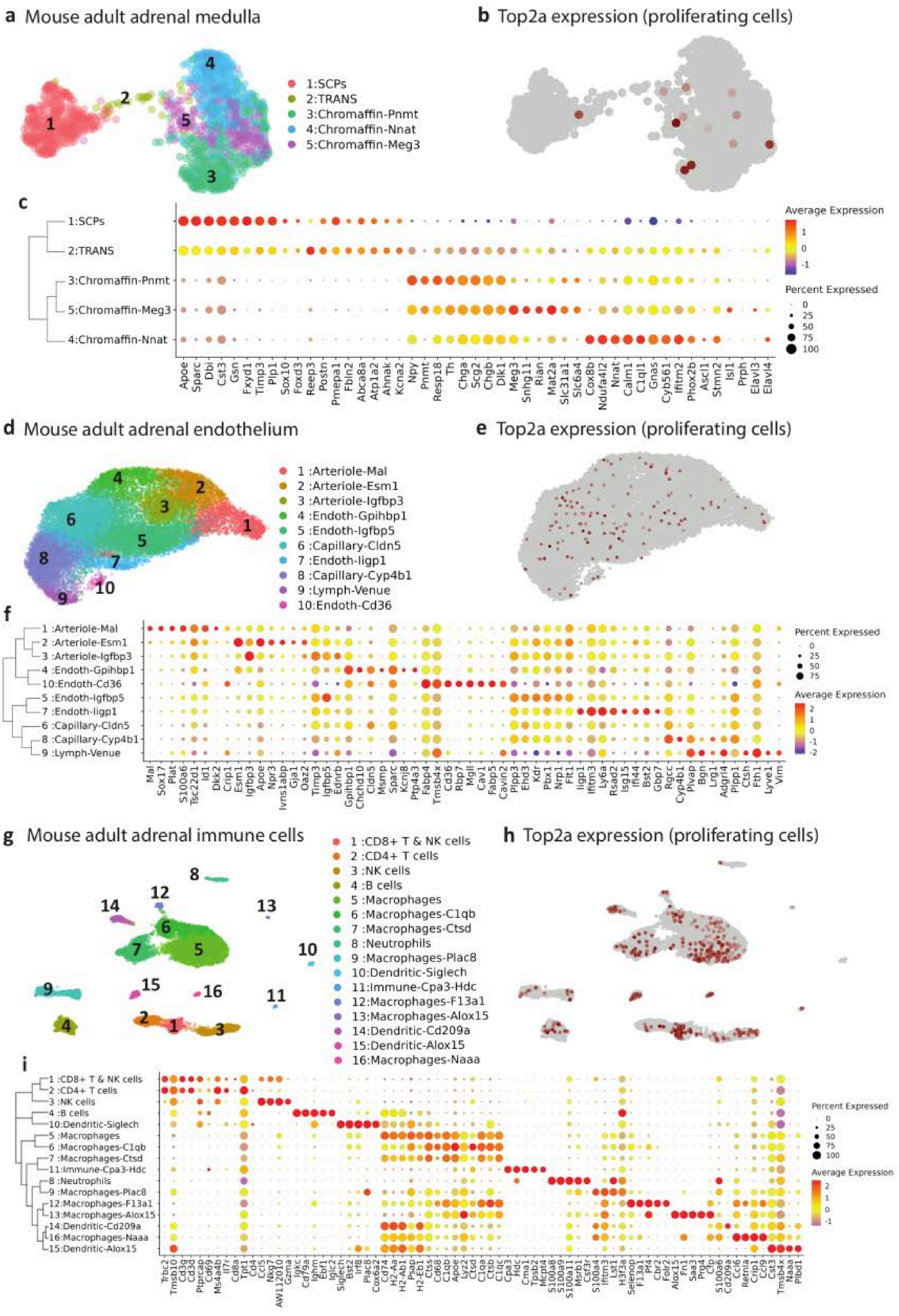

a Mouse adult adrenal medulla
1:SCPs
2:TRANS
3:Chromaffin-Pnmt
4:Chromaffin-Nnat
5:Chromaffin-Meg3
b Top2a expression (proliferating cells)
c
Average Expression
Percent Expressed
Apoe Sparc Dbi Cst3 Gsn Fxyd1 Timp3 Plp1 Sox10 Foxd3 Reep3 Postn Pmepa1 Fbln2 Abca8a Atp1a2 Ahnak Kcna2 Npy Pnmt Resp18 Th Chga Scg2 Chgb Dlk1 Meg3 Snhg11 Rian Mat2a Slc31a1 Slc6a4 Cox8b Ndufa4l2 Nnat Calm1 C1ql1 Gnas Cyb561 Ifitm2 Phox2b Ascl1 Stmn2 Isl1 Prph Elavl3 Elavl4
d Mouse adult adrenal endothelium
1 :Arteriole-Mal
2 :Arteriole-Esm1
3 :Arteriole-Igfbp3
4 :Endoth-Gpihbp1
5 :Endoth-Igfbp5
6 :Capillary-Cldn5
7 :Endoth-Iigp1
8 :Capillary-Cyp4b1
9 :Lymph-Venue
10:Endoth-Cd36
e Top2a expression (proliferating cells)
f
Percent Expressed
Average Expression
Mal Sox17 Plat S100a6 Tsc22d1 Id1 Dkk2 Crip1 Esm1 Igfbp3 Apoe Npr3 Ivns1abp Gja1 Oaz2 Timp3 Igfbp5 Ednrb Gpihbp1 Chchd10 Cldn5 Msmp Sparc Kcnj8 Ptp4a3 Fabp4 Tmsb4x Cd36 Rbp7 Mgll Cav1 Fabp5 Cavin2 Plpp3 Ehd3 Kdr Pbx1 Nrp1 Flt1 Iigp1 Ifitm3 Ly6a Rsad2 Isg15 Ifi44 Bst2 Gbp7 Rgcc Cyp4b1 Plvap Bgn Lrg1 Adgrl4 Plpp1 Ctsh Fth1 Lyve1 Vim
g Mouse adult adrenal immune cells
1 :CD8+ T & NK cells
2 :CD4+ T cells
3 :NK cells
4 :B cells
5 :Macrophages
6 :Macrophages-C1qb
7 :Macrophages-Ctsd
8 :Neutrophils
9 :Macrophages-Plac8
10:Dendritic-Siglech
11:Immune-Cpa3-Hdc
12:Macrophages-F13a1
13:Macrophages-Alox15
14:Dendritic-Cd209a
15:Dendritic-Alox15
16:Macrophages-Naaa
h Top2a expression (proliferating cells)
i
Percent Expressed
Average Expression

Supplementary Fig. 44 | Transcriptional heterogeneity of mouse adult adrenal medulla, endothelium and immune cells. a-c, Mouse adult adrenal medulla (1,325 cells). **d-f**, Mouse adult adrenal endothelium (22,948 cells). **g-i**, Mouse adult adrenal immune cells (12,056 cells). **a,d,g**, A UMAP embedding resulting from the focused reintegration of target mouse adrenal cell population. **b,e,h**, Top2a gene expression indicates location of proliferating cells on UMAP. **c,f,i**, DotPlot representation of marker genes for subpopulations of target mouse adrenal cell population. The hierarchy of clusters, based on transcriptional similarity, is shown on the left.

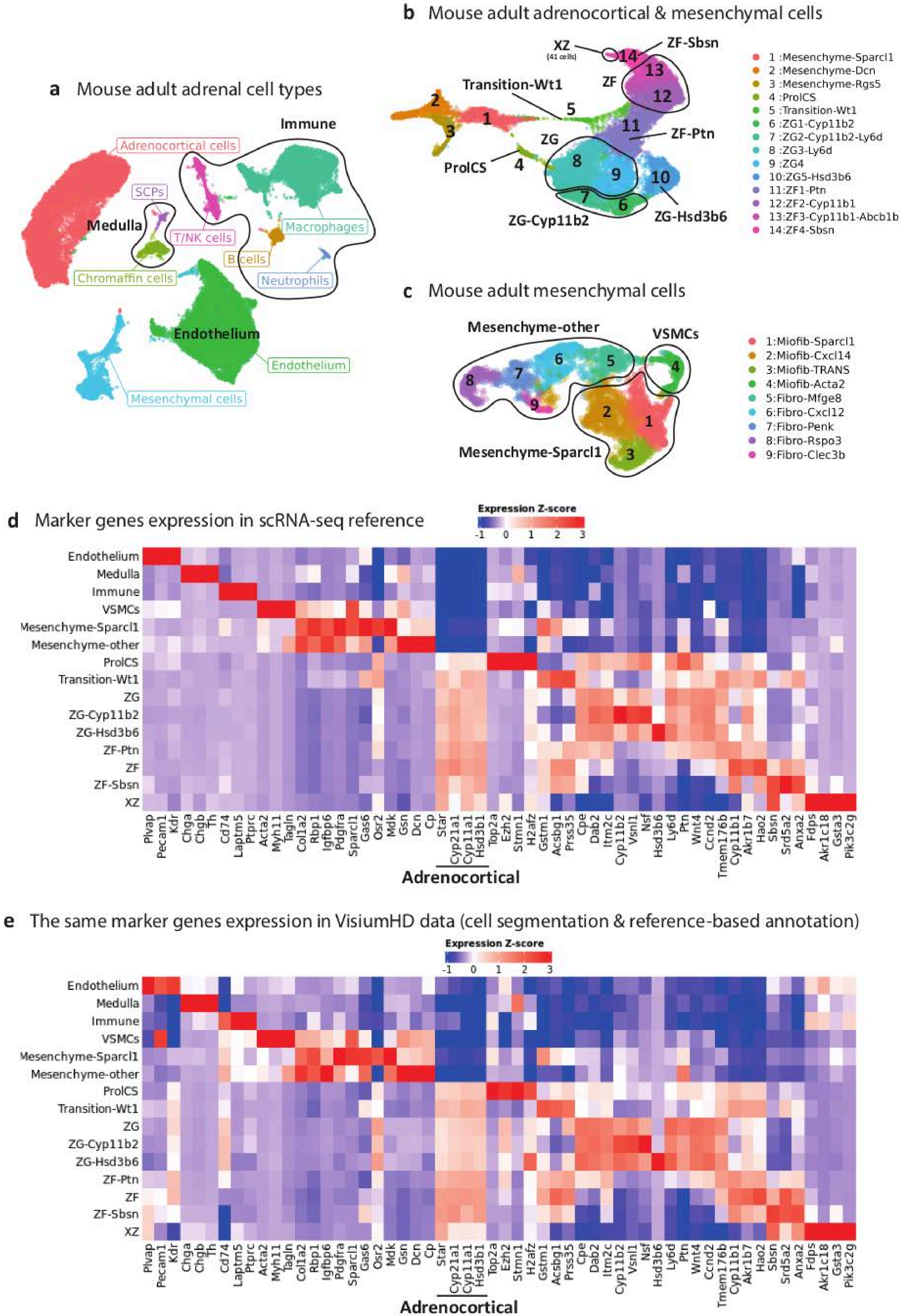
a Mouse adult adrenal cell types
Immune
Adrenocortical cells
SCPs
Medulla
T/NK cells
Macrophages
B cells
Chromaffin cells
Neutrophils
Endothelium
Endothelium
Mesenchymal cells
b Mouse adult adrenocortical & mesenchymal cells
XZ
(41 cells)
ZF-Sbsn
ZF
Transition-Wt1
ZG
ZF-Ptn
ProlCS
ZG-Cyp11b2
ZG-Hsd3b6
1 :Mesenchyme-Sparcl1
2 :Mesenchyme-Dcn
3 :Mesenchyme-Rgs5
4 :ProlCS
5 :Transition-Wt1
6 :ZG1-Cyp11b2
7 :ZG2-Cyp11b2-Ly6d
8 :ZG3-Ly6d
9 :ZG4
10:ZG5-Hsd3b6
11:ZF1-Ptn
12:ZF2-Cyp11b1
13:ZF3-Cyp11b1-Abcb1b
14:ZF4-Sbsn
c Mouse adult mesenchymal cells
Mesenchyme-other
VSMCs
Mesenchyme-Sparcl1
1:Miofib-Sparcl1
2:Miofib-Cxcl14
3:Miofib-TRANS
4:Miofib-Acta2
5:Fibro-Mfge8
6:Fibro-Cxcl12
7:Fibro-Penk
8:Fibro-Rspo3
9:Fibro-Clec3b
d Marker genes expression in scRNA-seq reference
Expression Z-score
-1 0 1 2 3
Endothelium
Medulla
Immune
VSMCs
Mesenchyme-Sparcl1
Mesenchyme-other
ProlCS
Transition-Wt1
ZG
ZG-Cyp11b2
ZG-Hsd3b6
ZF-Ptn
ZF
ZF-Sbsn
XZ
Plvap Pecam1 Kdr Chga Chgb Th Cd74 Laptm5 Ptprc Acta2 Myh11 Tagln Col1a2 Rbp1 Igfbp6 Pdgfra Sparcl1 Gas6 Osr2 Mdk Gsn Dcn Cp Star Cyp21a1 Cyp11a1 Hsd3b1 Top2a Ezh2 Stmn1 H2afz Gstm1 Acsbg1 Prss35 Cpe Dab2 Itm2c Cyp11b2 Vsnl1 Nsf Hsd3b6 Ly6d Ptn Wnt4 Ccnd2 Tmem176b Cyp11b1 Akr1b7 Hao2 Sbsn Srd5a2 Anxa2 Fdps Akr1c18 Gsta3 Pik3c2g
Adrenocortical
e The same marker genes expression in VisiumHD data (cell segmentation & reference-based annotation)
Expression Z-score
-1 0 1 2 3
Endothelium
Medulla
Immune
VSMCs
Mesenchyme-Sparcl1
Mesenchyme-other
ProlCS
Transition-Wt1
ZG
ZG-Cyp11b2
ZG-Hsd3b6
ZF-Ptn
ZF
ZF-Sbsn
XZ
Plvap Pecam1 Kdr Chga Chgb Th Cd74 Laptm5 Ptprc Acta2 Myh11 Tagln Col1a2 Rbp1 Igfbp6 Pdgfra Sparcl1 Gas6 Osr2 Mdk Gsn Dcn Cp Star Cyp21a1 Cyp11a1 Hsd3b1 Top2a Ezh2 Stmn1 H2afz Gstm1 Acsbg1 Prss35 Cpe Dab2 Itm2c Cyp11b2 Vsnl1 Nsf Hsd3b6 Ly6d Ptn Wnt4 Ccnd2 Tmem176b Cyp11b1 Akr1b7 Hao2 Sbsn Srd5a2 Anxa2 Fdps Akr1c18 Gsta3 Pik3c2g
Adrenocortical

Supplementary Fig. 45 | Cell type annotation of mouse high-resolution spatial transcriptomics (VisiumHD). a-c, Reference scRNA-seq UMAP embeddings with focused view on different aspects of adrenal cell type heterogeneity. On each UMAP or its cluster legend, highlighted cell states were included in reference and resolved in spatial data. **a,** The UMAP embedding of the mouse adult adrenal gland cells (67,665 cells), color coded by cell type. Medullary, immune or endothelial cells were annotated as single groups. **b,** The UMAP embedding resulting from integration of adrenocortical and mesenchymal cells, including mesenchymal cells (25,932 cells). Pairs of clusters without clear discriminative marker genes were joined to increase annotation procedure efficiency. **c,** The UMAP embedding resulting from the focused reintegration of mouse adult adrenal mesenchymal cells (3,560 cells). VSMCs subclusters don't have clear discriminative marker genes and were joined. **f-g,** Heatmaps representing the expression of marker genes in all cell states, identified using scRNA-seq reference data. Markers with high expression levels were manually selected based on differential expression analysis on scRNA-seq reference data. **f,** Expression in scRNA-seq reference data. **g,** Expression in spatial transcriptomics VisiumHD data (in segmented and annotated cells). ProlCS, proliferating cortical state; ZG, zona glomerulosa; ZF, zona fasciculata; XZ, X zone.

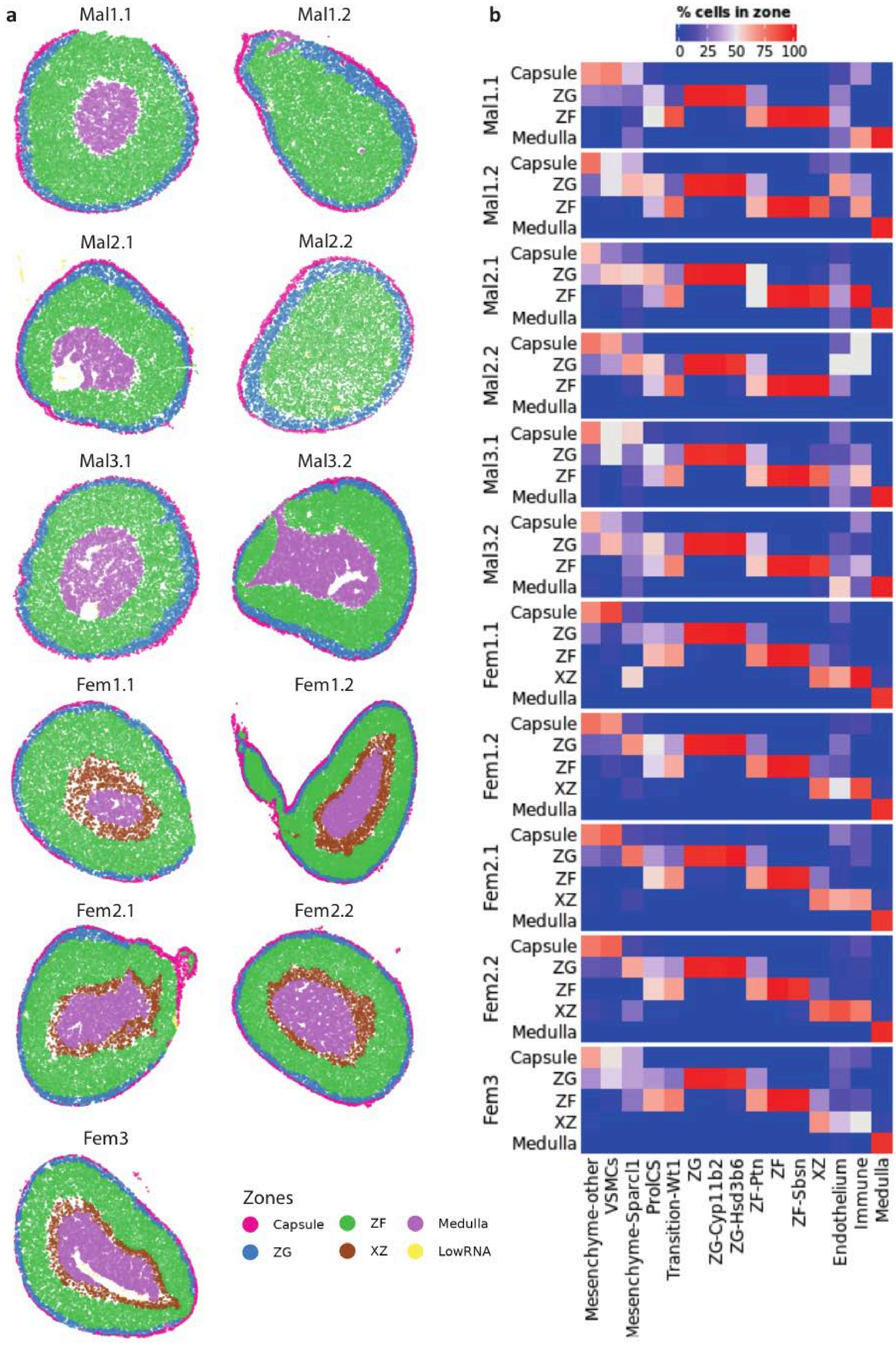

a
Mal1.1
Mal1.2
Mal2.1
Mal2.2
Mal3.1
Mal3.2
Fem1.1
Fem1.2
Fem2.1
Fem2.2
Fem3
Zones
Capsule
ZF
Medulla
ZG
XZ
LowRNA
b
% cells in zone
0 25 50 75 100
Capsule
ZG
ZF
XZ
Medulla
Mesenchyme-other
VSMCs
Mesenchyme-Sparcl1
ProlCS
Transition-Wt1
ZG
ZG-Cyp11b2
ZG-Hsd3b6
ZF-Ptn
ZF
ZF-Sbsn
XZ
Endothelium
Immune
Medulla

Supplementary Fig. 46 |. a, Adrenal zones and cell types spatial distribution across zones in mouse VisiumHD data. a, Adrenal zones on mouse adult adrenal tissue sections. Count of cells in each sample: Mal1.1 - 11,689; Mal1.2 - 10,632; Mal2.1 - 11,731; Mal2.2 - 7,157; Mal3.1 - 10,237; Mal3.2 - 17,830; Fem1.1 - 11,313; Fem1.2 - 20,685; Fem2.1 - 14,784; Fem2.2 - 16,446; Fem3 - 14,455. The cluster named Other mainly corresponds to poorly covered areas. **b**, Heatmaps (one row per sample) representing how each cell type is split by zones in the whole adrenal section (i.e. which percent of cells of ZG-Cyp11b2 cell type belong to ZG).

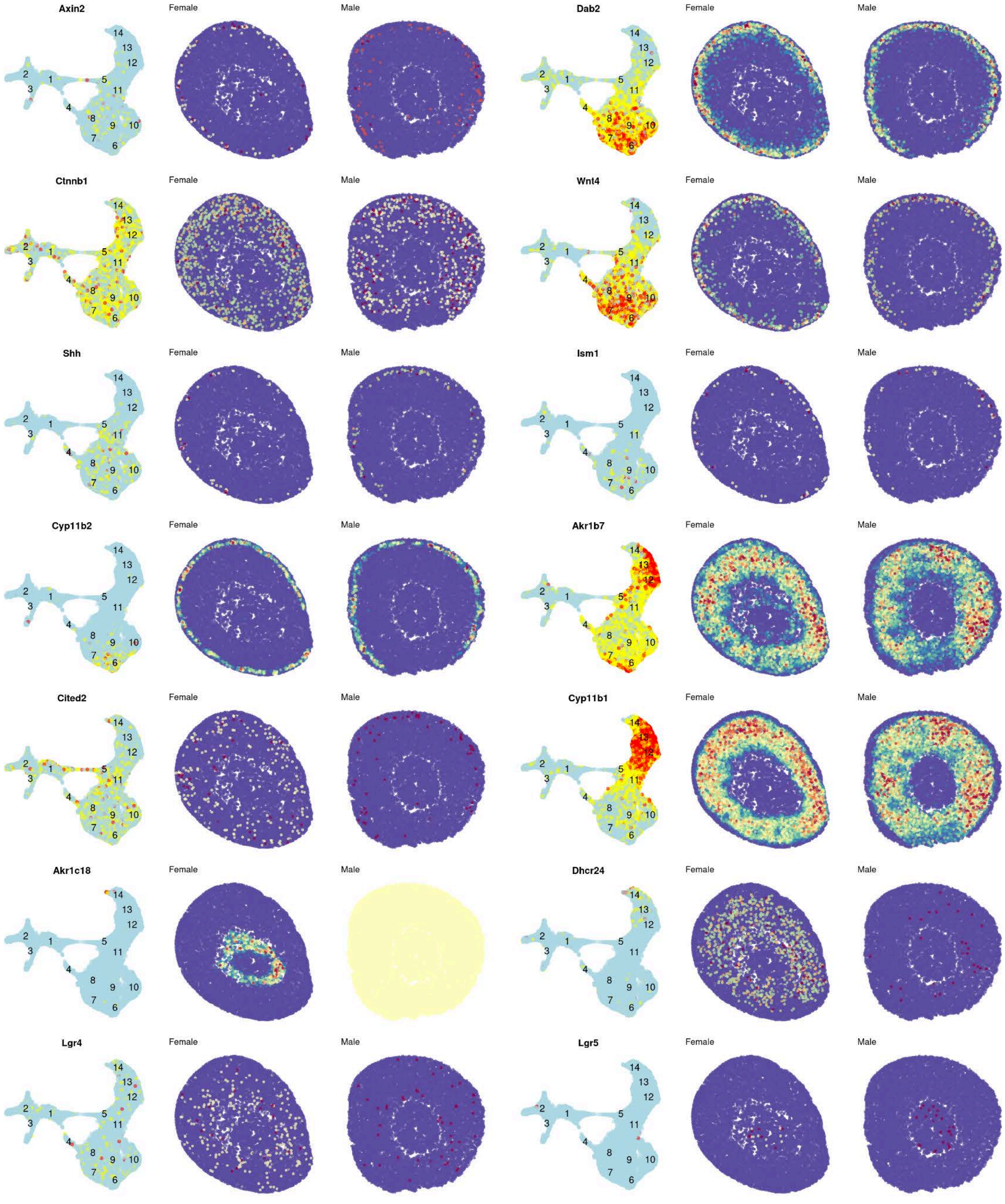
Axin2
Female
Male
Dab2
Female
Male
Ctnnb1
Female
Male
Wnt4
Female
Male
Shh
Female
Male
Ism1
Female
Male
Cyp11b2
Female
Male
Akr1b7
Female
Male
Cited2
Female
Male
Cyp11b1
Female
Male
Akr1c18
Female
Male
Dhcr24
Female
Male
Lgr4
Female
Male
Lgr5
Female
Male

Supplementary Fig. 47 | Marker genes driving mouse adult ZG and ZF transcriptomic heterogeneity. The expression of key marker genes in scRNA-seq data and high-resolution spatial transcriptomics (VisiumHD) of the two normal mouse adult adrenal samples: female (11,313 cells) and male (11,689 cells). Each dot corresponds to a segmented cell, with colors indicating gene expression level.

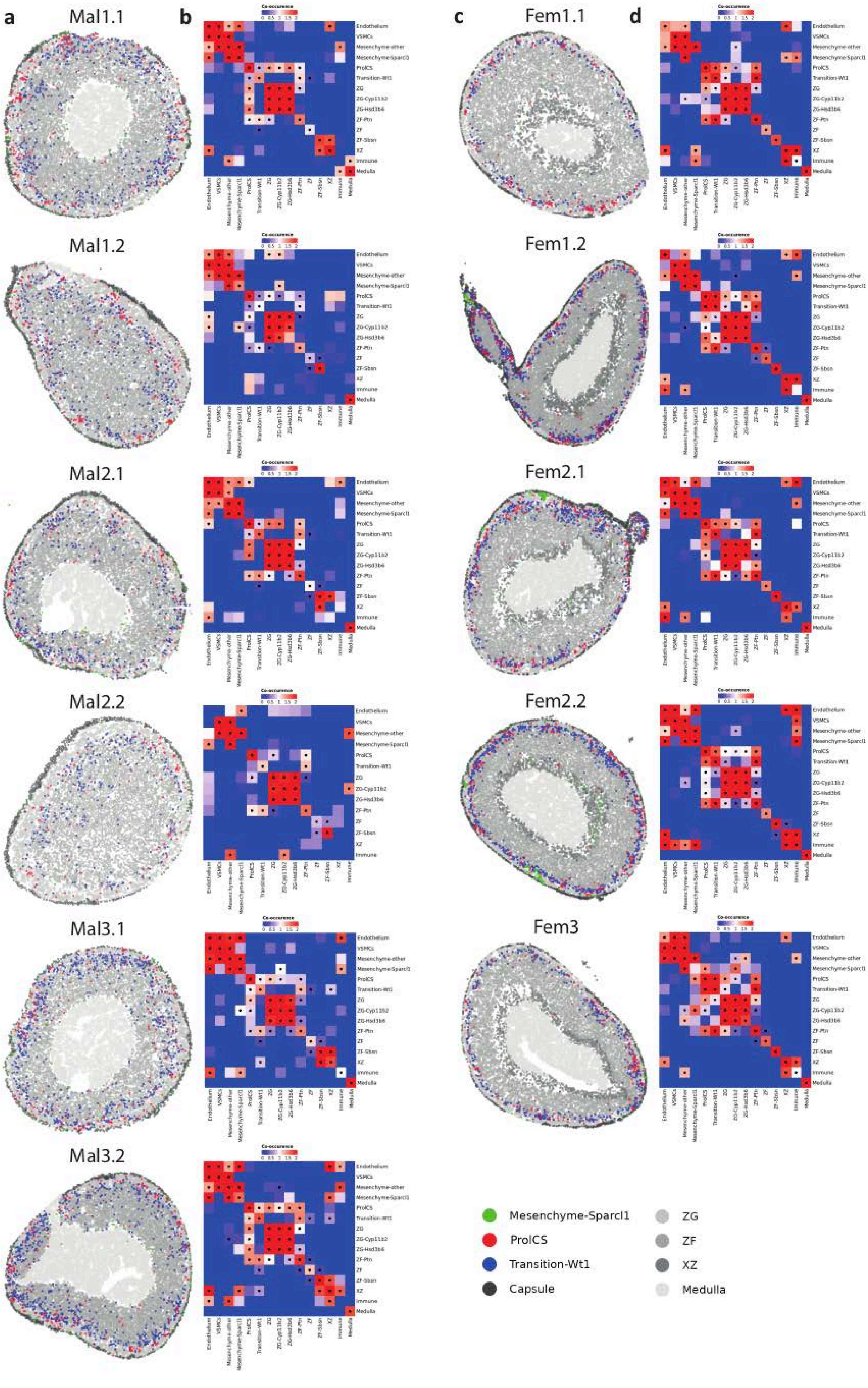
a
b
c
d
Mal1.1
Mal1.2
Mal2.1
Mal2.2
Mal3.1
Mal3.2
Fem1.1
Fem1.2
Fem2.1
Fem2.2
Fem3
Co-occurence
Endothelium
VSMCs
Mesenchyme-other
Mesenchyme-Sparcl1
ProlCS
Transition-Wt1
ZG
ZG-Cyp11b2
ZG-Hsd3b6
ZF-Ptn
ZF
ZF-Sbsn
XZ
Immune
Medulla
Mesenchyme-Sparcl1
ProlCS
Transition-Wt1
Capsule
ZG
ZF
XZ
Medulla

Supplementary Fig. 48 | Spatial distribution and co-occurrence of renewal-associated cell types or states in mouse VisiumHD data. a,**c**, Renewal-associated cell types (states) were identified in high-resolution spatial transcriptomics (VisiumHD) data. Each subplot represents one sample. Putative transitions are from Mesenchyme-Sparcl1 (green) to ProlCS (red) or Transition-Wt1 (blue). Background gray colors highlight adrenal zones. **b**,**d**, Spatial co-occurrence of cell states in the whole adrenal section. Dots highlight statistically significant enrichment of co-occurrence, adjusted p-value < 0.05 (see Methods section). **a-b**, Male samples. Count of cells in each sample: Mal1.1 - 11,689; Mal1.2 - 10,632; Mal2.1 - 11,731; Mal2.2 - 7,157; Mal3.1 - 10,237; Mal3.2 - 17,830. **c-d**, Female samples. Count of cells in each sample: Fem1.1 - 11,313; Fem1.2 - 20,685; Fem2.1 - 14,784; Fem2.2 - 16,446; Fem3 - 14,455. ProlCS, proliferating cortical state; ZG, zona glomerulosa; ZF, zona fasciculata; ZR, zona reticularis.

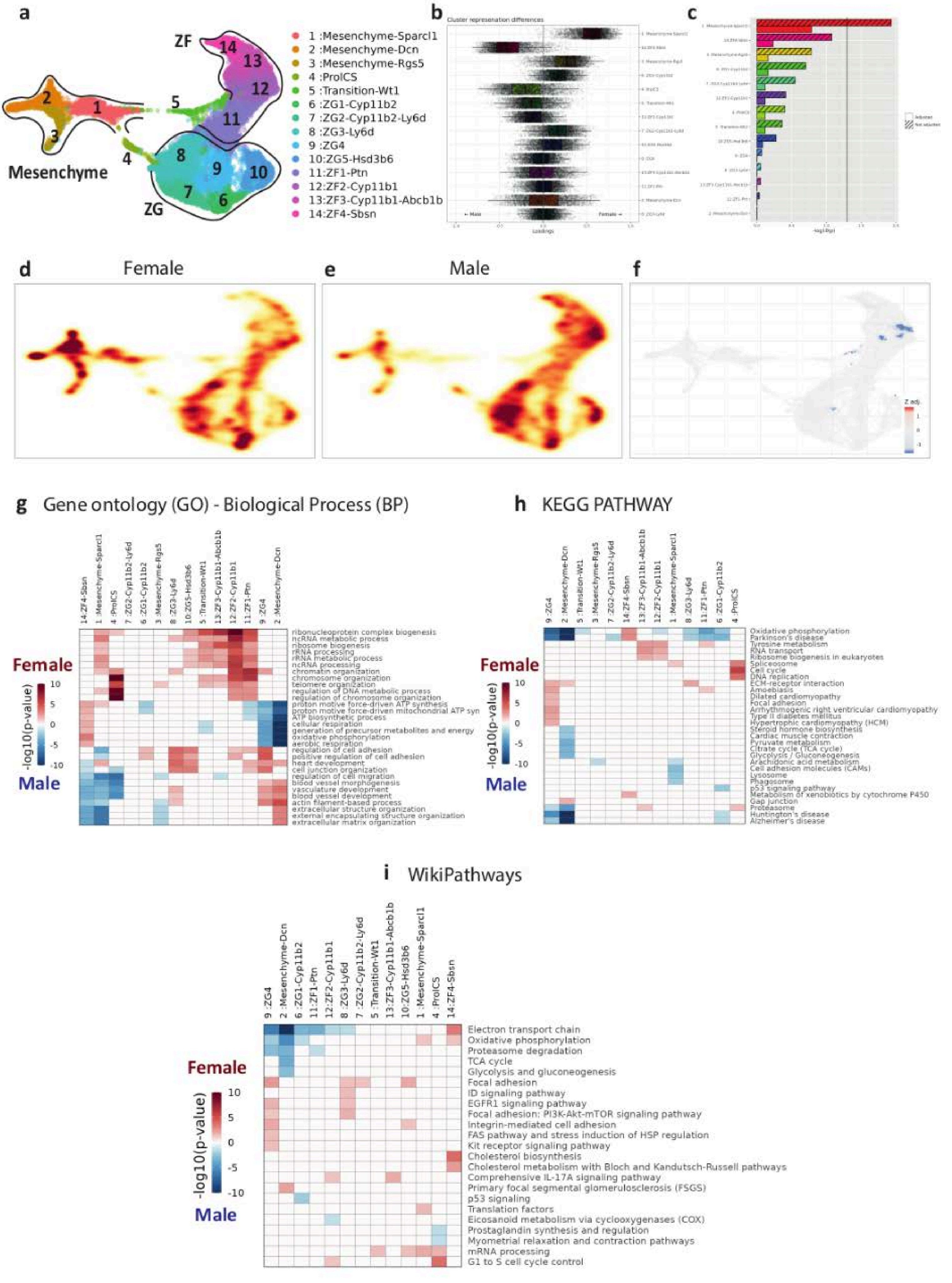
a
ZF
14
13
12
11
5
2
1
3
4
8
9
10
7
6
Mesenchyme
ZG
1 :Mesenchyme-Sparcl1
2 :Mesenchyme-Dcn
3 :Mesenchyme-Rgs5
4 :ProlCS
5 :Transition-Wt1
6 :ZG1-Cyp11b2
7 :ZG2-Cyp11b2-Ly6d
8 :ZG3-Ly6d
9 :ZG4
10:ZG5-Hsd3b6
11:ZF1-Ptn
12:ZF2-Cyp11b1
13:ZF3-Cyp11b1-Abcb1b
14:ZF4-Sbsn
b
Cluster representation differences
c
d
Female
e
Male
f
Z adj.
g
Gene ontology (GO) - Biological Process (BP)
14:ZF4-Sbsn
1 :Mesenchyme-Sparcl1
4 :ProlCS
7 :ZG2-Cyp11b2-Ly6d
6 :ZG1-Cyp11b2
3 :Mesenchyme-Rgs5
8 :ZG3-Ly6d
10:ZG5-Hsd3b6
5 :Transition-Wt1
13:ZF3-Cyp11b1-Abcb1b
12:ZF2-Cyp11b1
11:ZF1-Ptn
9 :ZG4
2 :Mesenchyme-Dcn
Female
-log10(p-value)
10
5
0
-5
-10
Male
ribonucleoprotein complex biogenesis
ncRNA metabolic process
ribosome biogenesis
rRNA processing
rRNA metabolic process
ncRNA processing
chromatin organization
chromosome organization
telomere organization
regulation of DNA metabolic process
regulation of chromosome organization
proton motive force-driven ATP synthesis
proton motive force-driven mitochondrial ATP syn
ATP biosynthetic process
cellular respiration
generation of precursor metabolites and energy
oxidative phosphorylation
aerobic respiration
regulation of cell adhesion
positive regulation of cell adhesion
heart development
cell junction organization
regulation of cell migration
blood vessel morphogenesis
vasculature development
blood vessel development
actin filament-based process
extracellular structure organization
external encapsulating structure organization
extracellular matrix organization
h
KEGG PATHWAY
9 :ZG4
2 :Mesenchyme-Dcn
5 :Transition-Wt1
3 :Mesenchyme-Rgs5
7 :ZG2-Cyp11b2-Ly6d
14:ZF4-Sbsn
13:ZF3-Cyp11b1-Abcb1b
12:ZF2-Cyp11b1
1 :Mesenchyme-Sparcl1
8 :ZG3-Ly6d
11:ZF1-Ptn
6 :ZG1-Cyp11b2
4 :ProlCS
Female
-log10(p-value)
10
5
0
-5
-10
Male
Oxidative phosphorylation
Parkinson's disease
Tyrosine metabolism
RNA transport
Ribosome biogenesis in eukaryotes
Spliceosome
Cell cycle
DNA replication
ECM-receptor interaction
Amoebiasis
Dilated cardiomyopathy
Focal adhesion
Arrhythmogenic right ventricular cardiomyopathy
Type II diabetes mellitus
Hypertrophic cardiomyopathy (HCM)
Steroid hormone biosynthesis
Cardiac muscle contraction
Pyruvate metabolism
Citrate cycle (TCA cycle)
Glycolysis / Gluconeogenesis
Arachidonic acid metabolism
Cell adhesion molecules (CAMs)
Lysosome
Phagosome
p53 signaling pathway
Metabolism of xenobiotics by cytochrome P450
Gap junction
Proteasome
Huntington's disease
Alzheimer's disease
i
WikiPathways
9 :ZG4
2 :Mesenchyme-Dcn
6 :ZG1-Cyp11b2
11:ZF1-Ptn
12:ZF2-Cyp11b1
8 :ZG3-Ly6d
7 :ZG2-Cyp11b2-Ly6d
5 :Transition-Wt1
13:ZF3-Cyp11b1-Abcb1b
10:ZG5-Hsd3b6
1 :Mesenchyme-Sparcl1
4 :ProlCS
14:ZF4-Sbsn
Female
-log10(p-value)
10
5
0
-5
-10
Male
Electron transport chain
Oxidative phosphorylation
Proteasome degradation
TCA cycle
Glycolysis and gluconeogenesis
Focal adhesion
ID signaling pathway
EGFR1 signaling pathway
Focal adhesion: PI3K-Akt-mTOR signaling pathway
Integrin-mediated cell adhesion
FAS pathway and stress induction of HSP regulation
Kit receptor signaling pathway
Cholesterol biosynthesis
Cholesterol metabolism with Bloch and Kandutsch-Russell pathways
Comprehensive IL-17A signaling pathway
Primary focal segmental glomerulosclerosis (FSGS)
p53 signaling
Translation factors
Eicosanoid metabolism via cyclooxygenases (COX)
Prostaglandin synthesis and regulation
Myometrial relaxation and contraction pathways
mRNA processing
G1 to S cell cycle control

Supplementary Fig. 49 | Sexual dimorphism of mouse adult adrenal cortex. a-f, Compositional differences related to the original adrenocortical and mesenchymal cell embedding and clusterization. All results were obtained using cacoa R package, see Methods section. **a**, Mouse adult adrenocortical and mesenchymal cells embedding (21,348 cells). **b-c**, Cluster based analysis of compositional differences. **b**, Estimated loadings for cluster representation differences. Each dot corresponds to a permutation iteration (n.boot=5000). Positive loading values indicate cluster overrepresentation in females, negative - in males. For each box-plot, center line, median; box limits, upper and lower quartiles; whiskers, 1.5x interquartile range. **c**, Statistical significance of observed cluster representation differences. Dashed bars correspond to p-values before adjusting for multiple hypothesis testing, not dashed - after correction. The vertical line is set at the significance level p-value = 0.05. **d-f**, Cluster-free, embedding cell density based analysis of compositional differences. **d**, Cell density in females. **e**, Cell density in males. **f**, Wilcox test estimation of difference significance in cell density. Red indicates areas of increased density in females, blue - in males.

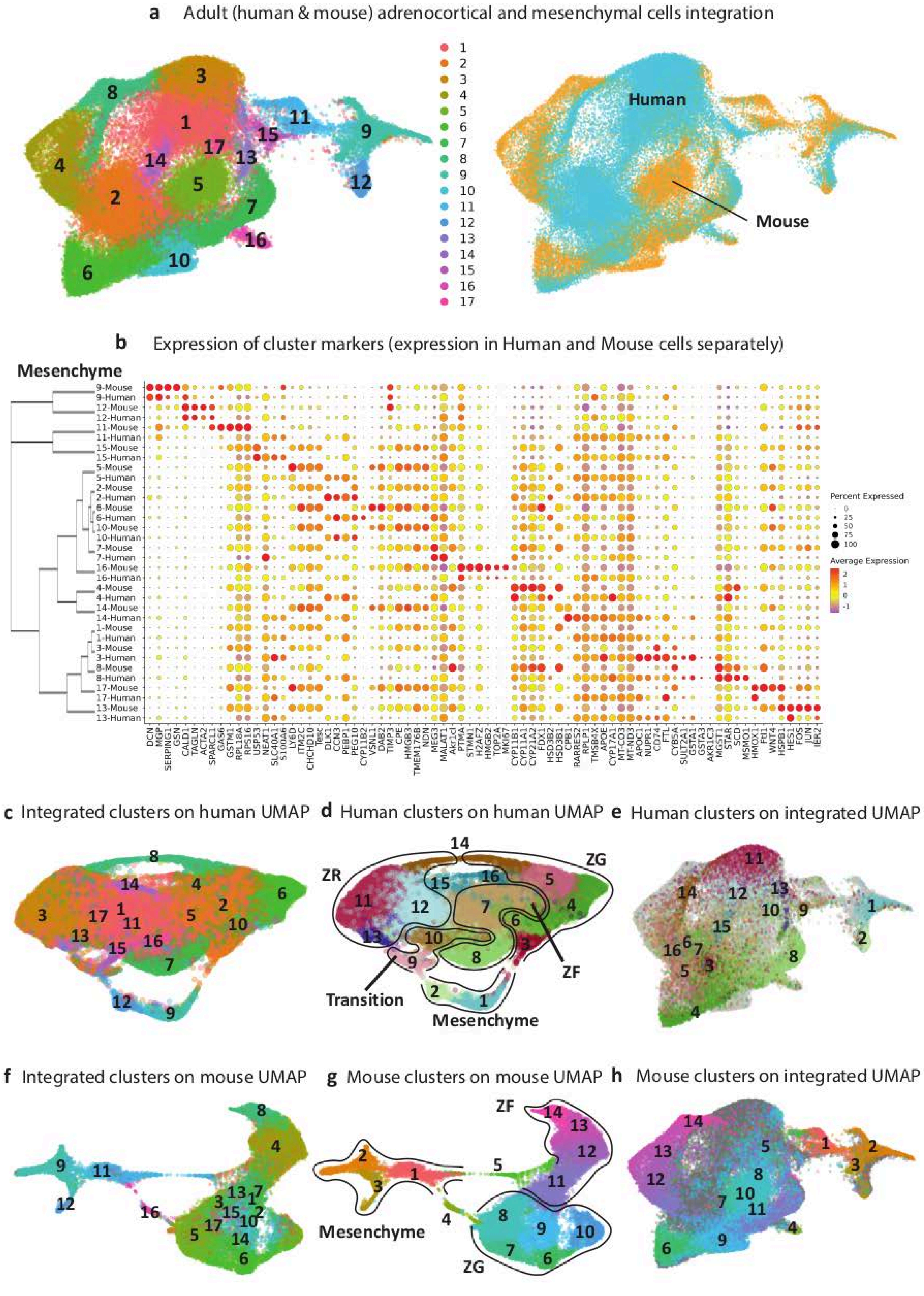
a Adult (human & mouse) adrenocortical and mesenchymal cells integration
Human
Mouse
b Expression of cluster markers (expression in Human and Mouse cells separately)
Mesenchyme
Percent Expressed
0
25
50
75
100
Average Expression
2
1
0
-1
9-Mouse
9-Human
12-Mouse
12-Human
11-Mouse
11-Human
15-Mouse
15-Human
5-Mouse
5-Human
2-Mouse
2-Human
6-Mouse
6-Human
10-Mouse
10-Human
7-Mouse
7-Human
16-Mouse
16-Human
4-Mouse
4-Human
14-Mouse
14-Human
1-Mouse
1-Human
3-Mouse
3-Human
8-Mouse
8-Human
17-Mouse
17-Human
13-Mouse
13-Human
DCN MGP SERPING1 GSN CALD1 TAGLN ACTA2 SPARCL1 GAS6 GSTM1 RPL18A RPS16 USP53 NEAT1 SLC40A1 S100A6 LY6D ITM2C CHCHD10 Tesc DLK1 CCN3 PEBP1 PEG10 CYP11B2 VSNL1 DAB2 TIMP3 CPE HMGB3 TMEM176B NDN MEG3 MALAT1 Akr1cl PTMA STMN1 H2AFZ HMGB2 TOP2A MKI67 CYP11B1 CYP11A1 CYP21A2 FDX1 HSD3B2 HSD3B1 CPB1 RARRES2 RPLP1 TMSB4X APOE CYP17A1 MT-CO3 MT-ND3 APOC1 NUPR1 CD74 FTL CYB5A SULT2A1 GSTA1 GSTA3 AKR1C3 MGST1 STAR SCD MSMO1 HMOX1 Ftl1 WNT4 HSPB1 HES1 FOS JUN IER2
c Integrated clusters on human UMAP
d Human clusters on human UMAP
ZR
ZG
ZF
Transition
Mesenchyme
e Human clusters on integrated UMAP
f Integrated clusters on mouse UMAP
g Mouse clusters on mouse UMAP
ZF
ZG
Mesenchyme
h Mouse clusters on integrated UMAP

Supplementary Fig. 50 | Human and mouse adult adrenocortical and mesenchymal cells integration. a, A UMAP embedding resulting from the integration of human (39,985 cells) and mouse (25,932 cells) adult adrenocortical and mesenchymal cells (total 65,917 cells). Left - integrated clusterization, right - compositional impact of human (blue) and mouse (yellow) data. **b**, DotPlot displaying cluster-specific marker genes and key steroidogenic enzymes. The hierarchy of clusters, based on transcriptional similarity, is shown on the left. For each cluster gene expression is shown for humans and mice separately. **c-h**, Comparison of human or mouse specific UMAPs and clusterization with integrated ones. **c**, Integrated clusters on human UMAP. **d**, Human clusters on human UMAP. **e**, Human clusters on integrated UMAP. **f**, Integrated clusters on mouse UMAP. **g**, Mouse clusters on mouse UMAP. **h**, Mouse clusters on integrated UMAP.

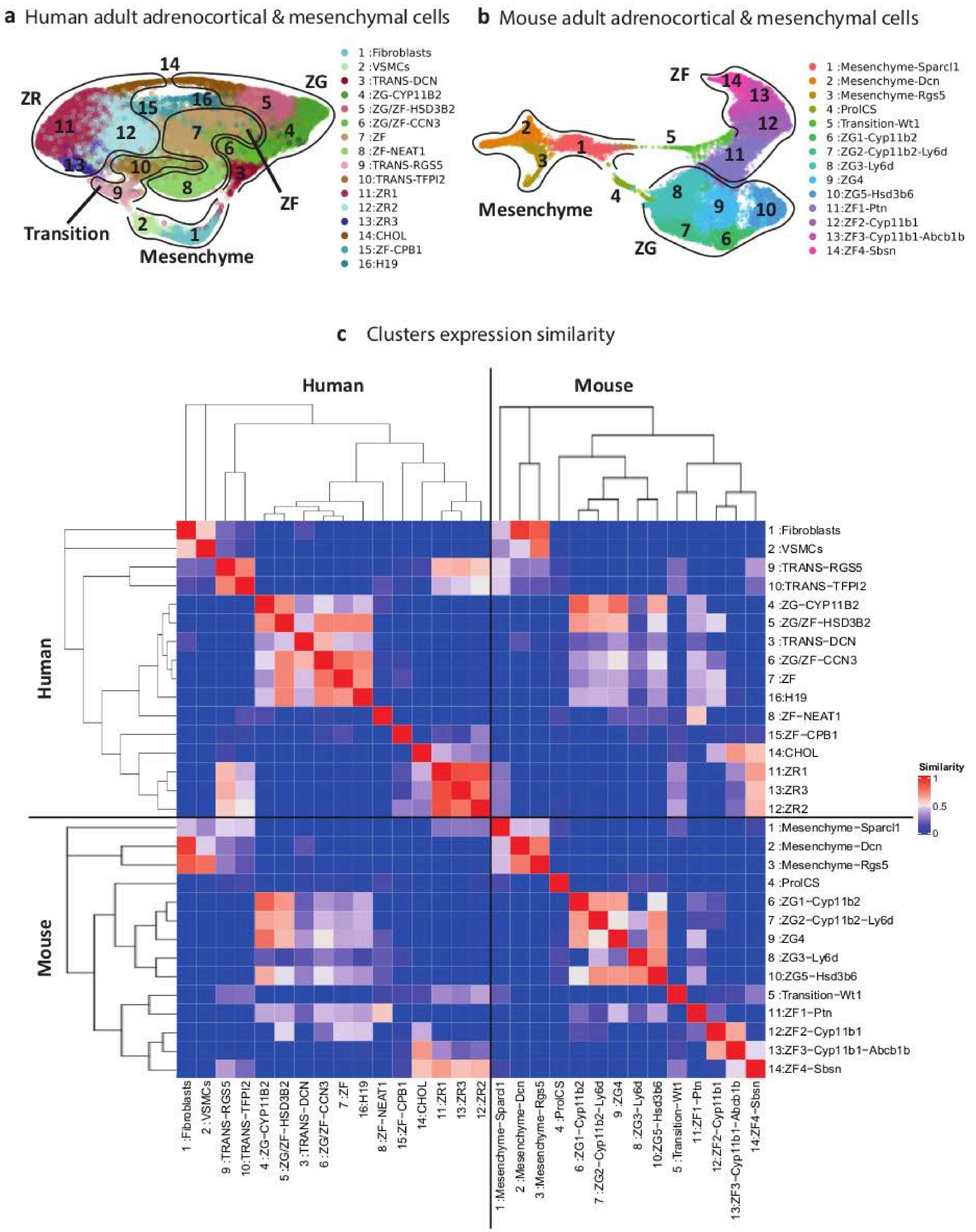
a Human adult adrenocortical & mesenchymal cells
ZR
ZG
ZF
Transition
Mesenchyme
1 :Fibroblasts
2 :VSMCs
3 :TRANS-DCN
4 :ZG-CYP11B2
5 :ZG/ZF-HSD3B2
6 :ZG/ZF-CCN3
7 :ZF
8 :ZF-NEAT1
9 :TRANS-RGS5
10:TRANS-TFPI2
11:ZR1
12:ZR2
13:ZR3
14:CHOL
15:ZF-CPB1
16:H19
b Mouse adult adrenocortical & mesenchymal cells
ZF
Mesenchyme
ZG
1 :Mesenchyme-Sparcl1
2 :Mesenchyme-Dcn
3 :Mesenchyme-Rgs5
4 :ProlCS
5 :Transition-Wt1
6 :ZG1-Cyp11b2
7 :ZG2-Cyp11b2-Ly6d
8 :ZG3-Ly6d
9 :ZG4
10:ZG5-Hsd3b6
11:ZF1-Ptn
12:ZF2-Cyp11b1
13:ZF3-Cyp11b1-Abcb1b
14:ZF4-Sbsn
c Clusters expression similarity
Human
Mouse
Similarity
1
0.5
0
1 :Fibroblasts
2 :VSMCs
9 :TRANS-RGS5
10:TRANS-TFPI2
4 :ZG-CYP11B2
5 :ZG/ZF-HSD3B2
3 :TRANS-DCN
6 :ZG/ZF-CCN3
7 :ZF
16:H19
8 :ZF-NEAT1
15:ZF-CPB1
14:CHOL
11:ZR1
13:ZR3
12:ZR2
1 :Mesenchyme-Sparcl1
2 :Mesenchyme-Dcn
3 :Mesenchyme-Rgs5
4 :ProlCS
6 :ZG1-Cyp11b2
7 :ZG2-Cyp11b2-Ly6d
9 :ZG4
8 :ZG3-Ly6d
10:ZG5-Hsd3b6
5 :Transition-Wt1
11:ZF1-Ptn
12:ZF2-Cyp11b1
13:ZF3-Cyp11b1-Abcb1b
14:ZF4-Sbsn

Supplementary Fig. 51 | Human and mouse adult adrenocortical and mesenchymal cell clusters comparison. **a**, Human adult adrenocortical and mesenchymal cells embedding (39,985 cells). **b**, Mouse adult adrenocortical and mesenchymal cells embedding (25,932 cells). **c**, Expression similarity between clusters was calculated as a pearson correlation (clipped at value 0) between cell average batch corrected variable gene expression vectors and visualized as a heatmap. The original hierarchies of human and mouse clusters, based on transcriptional similarity, are shown on the left and above. Black lines separate human and mouse clusters.

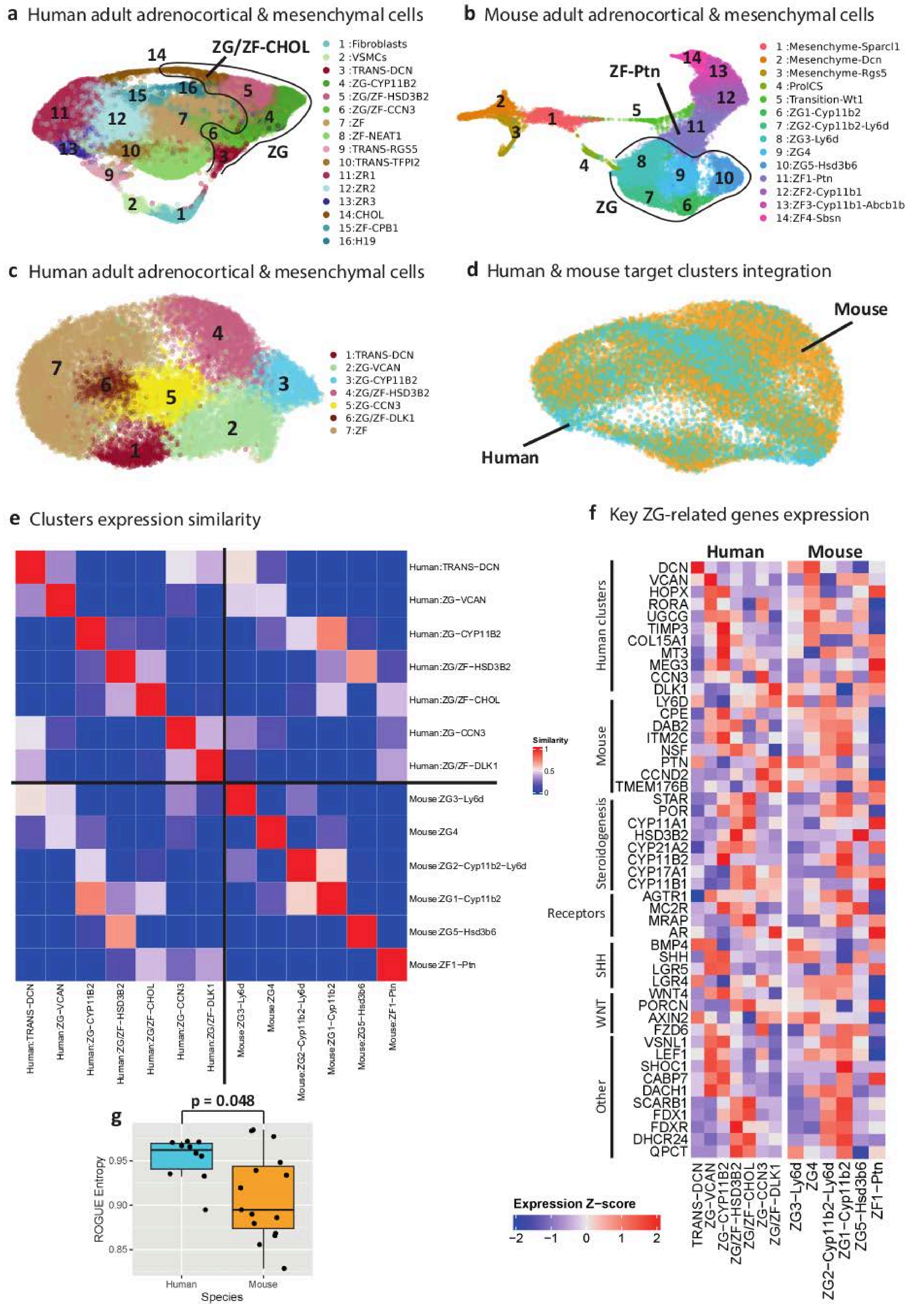
a Human adult adrenocortical & mesenchymal cells
ZG/ZF-CHOL
ZG
1 :Fibroblasts
2 :VSMCs
3 :TRANS-DCN
4 :ZG-CYP11B2
5 :ZG/ZF-HSD3B2
6 :ZG/ZF-CCN3
7 :ZF
8 :ZF-NEAT1
9 :TRANS-RGS5
10:TRANS-TFPI2
11:ZR1
12:ZR2
13:ZR3
14:CHOL
15:ZF-CPB1
16:H19
b Mouse adult adrenocortical & mesenchymal cells
ZF-Ptn
ZG
1 :Mesenchyme-Sparcl1
2 :Mesenchyme-Dcn
3 :Mesenchyme-Rgs5
4 :ProlCS
5 :Transition-Wt1
6 :ZG1-Cyp11b2
7 :ZG2-Cyp11b2-Ly6d
8 :ZG3-Ly6d
9 :ZG4
10:ZG5-Hsd3b6
11:ZF1-Ptn
12:ZF2-Cyp11b1
13:ZF3-Cyp11b1-Abcb1b
14:ZF4-Sbsn
c Human adult adrenocortical & mesenchymal cells
1:TRANS-DCN
2:ZG-VCAN
3:ZG-CYP11B2
4:ZG/ZF-HSD3B2
5:ZG-CCN3
6:ZG/ZF-DLK1
7:ZF
d Human & mouse target clusters integration
Mouse
Human
e Clusters expression similarity
Human:TRANS−DCN
Human:ZG−VCAN
Human:ZG−CYP11B2
Human:ZG/ZF−HSD3B2
Human:ZG/ZF−CHOL
Human:ZG−CCN3
Human:ZG/ZF−DLK1
Mouse:ZG3−Ly6d
Mouse:ZG4
Mouse:ZG2−Cyp11b2−Ly6d
Mouse:ZG1−Cyp11b2
Mouse:ZG5−Hsd3b6
Mouse:ZF1−Ptn
Similarity
1
0.5
0
f Key ZG-related genes expression
Human
Mouse
Human clusters
Mouse
Steroidogenesis
Receptors
SHH
WNT
Other
DCN
VCAN
HOPX
RORA
UGCG
TIMP3
COL15A1
MT3
MEG3
CCN3
DLK1
LY6D
CPE
DAB2
ITM2C
NSF
PTN
CCND2
TMEM176B
STAR
POR
CYP11A1
HSD3B2
CYP21A2
CYP11B2
CYP17A1
CYP11B1
AGTR1
MC2R
MRAP
AR
BMP4
SHH
LGR5
LGR4
WNT4
PORCN
AXIN2
FZD6
VSNL1
LEF1
SHOC1
CABP7
DACH1
SCARB1
FDX1
FDXR
DHCR24
QPCT
TRANS−DCN
ZG−VCAN
ZG−CYP11B2
ZG/ZF−HSD3B2
ZG/ZF−CHOL
ZG−CCN3
ZG/ZF−DLK1
ZG3−Ly6d
ZG4
ZG2−Cyp11b2−Ly6d
ZG1−Cyp11b2
ZG5−Hsd3b6
ZF1−Ptn
Expression Z−score
−2 −1 0 1 2
g
p = 0.048
ROGUE Entropy
0.95
0.90
0.85
Human
Mouse
Species

Supplementary Fig. 52 | Human and mouse adult zona glomerulosa focused integration. **a**, Human adult adrenocortical and mesenchymal cells embedding (39,985 cells). Target clusters for interspecies integration are indicated by line and labels (10,594 cells). **b**, Mouse adult adrenocortical and mesenchymal cells embedding (25,932 cells). Target clusters for interspecies integration are indicated by line and labels (15,316 cells). **c**, Focused UMAP embedding and focused clusterization of human zona glomerulosa (17,260 cells). It defines human clusters on **e**. **d**, A joint UMAP embedding resulting from the integration of target human and mouse clusters (25,910 cells). **e**, Expression similarity between clusters was calculated as a pearson correlation (clipped at value 0) between cell average batch corrected variable gene expression vectors and visualized as a heatmap. The original human and mouse clusters are shown on the right and below. Black lines separate human and mouse clusters. **f**, Expression heatmap of key marker genes. Z-scores were calculated for human and mouse data separately. **g**, ROGUE entropy of selected cell groups (ZG and early ZF, same as for the focused integration) in each sample was calculated on a common gene set. Two-sided Wilcoxon test was utilized for human vs mouse entropy comparison. Higher entropy points to higher transcriptional heterogeneity within a specified cell group.

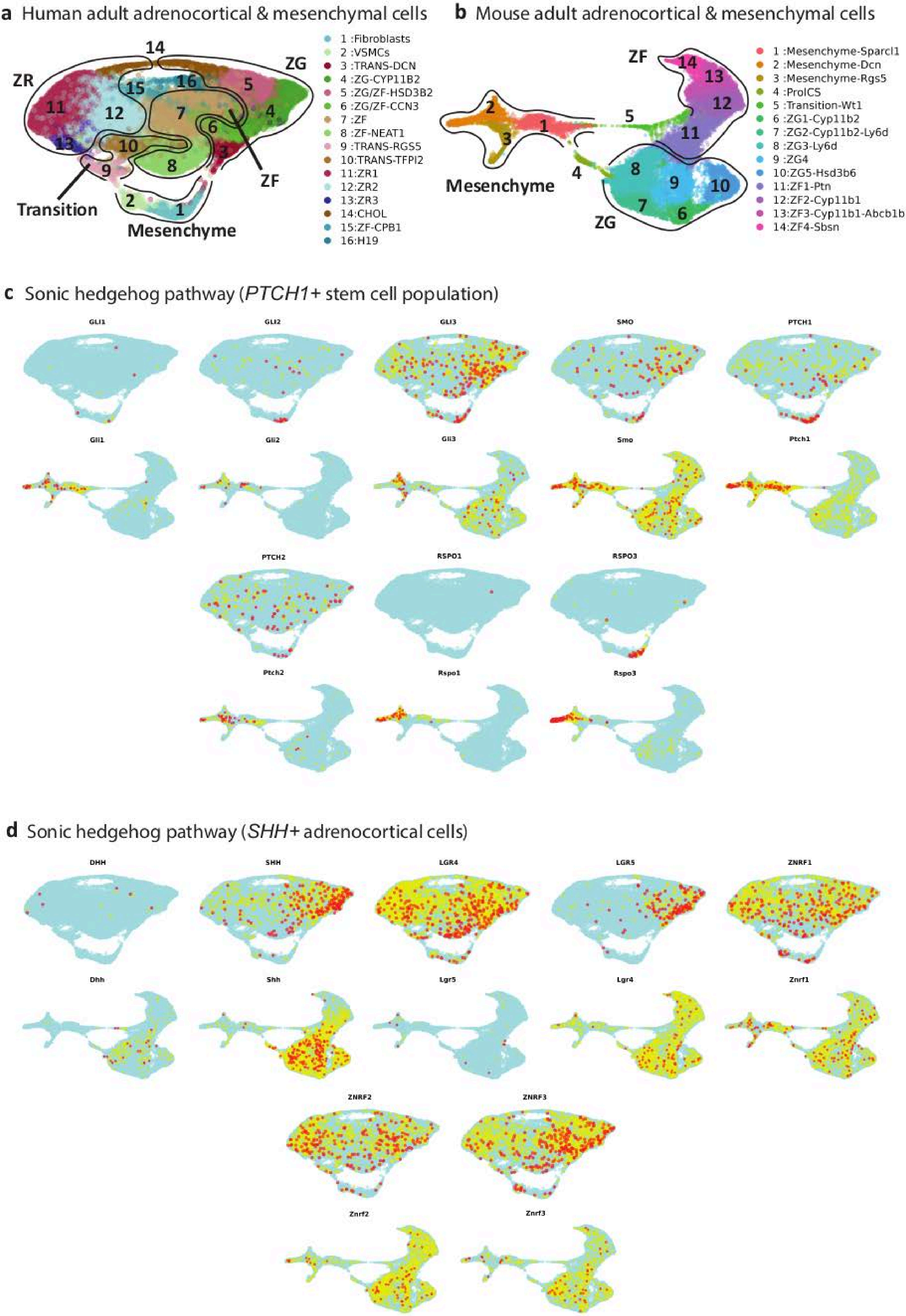
a Human adult adrenocortical & mesenchymal cells
ZR
14
ZG
ZF
Transition
Mesenchyme
1 :Fibroblasts
2 :VSMCs
3 :TRANS-DCN
4 :ZG-CYP11B2
5 :ZG/ZF-HSD3B2
6 :ZG/ZF-CCN3
7 :ZF
8 :ZF-NEAT1
9 :TRANS-RGS5
10:TRANS-TFPI2
11:ZR1
12:ZR2
13:ZR3
14:CHOL
15:ZF-CPB1
16:H19
b Mouse adult adrenocortical & mesenchymal cells
ZF
Mesenchyme
ZG
1 :Mesenchyme-Sparcl1
2 :Mesenchyme-Dcn
3 :Mesenchyme-Rgs5
4 :ProlCS
5 :Transition-Wt1
6 :ZG1-Cyp11b2
7 :ZG2-Cyp11b2-Ly6d
8 :ZG3-Ly6d
9 :ZG4
10:ZG5-Hsd3b6
11:ZF1-Ptn
12:ZF2-Cyp11b1
13:ZF3-Cyp11b1-Abcb1b
14:ZF4-Sbsn
c Sonic hedgehog pathway (PTCH1+ stem cell population)
GLI1
GLI2
GLI3
SMO
PTCH1
Gli1
Gli2
Gli3
Smo
Ptch1
PTCH2
RSPO1
RSPO3
Ptch2
Rspo1
Rspo3
d Sonic hedgehog pathway (SHH+ adrenocortical cells)
DHH
SHH
LGR4
LGR5
ZNRF1
Dhh
Shh
Lgr5
Lgr4
Znrf1
ZNRF2
ZNRF3
Znrf2
Znrf3

Supplementary Fig. 53 | Sonic Hedgehog pathway activity in human and mouse adrenals. **a**, Human adult adrenocortical and mesenchymal cells embedding (39,985 cells). **b**, Mouse adult adrenocortical and mesenchymal cells embedding (25,932 cells). **c-d**, Expression of key genes involved in Sonic Hedgehog pathway on human (above) and mouse (below) embeddings. **c**, Side of *PTCH1*+ stem cells. **d**, Side of *SHH*+ adrenocortical cells.

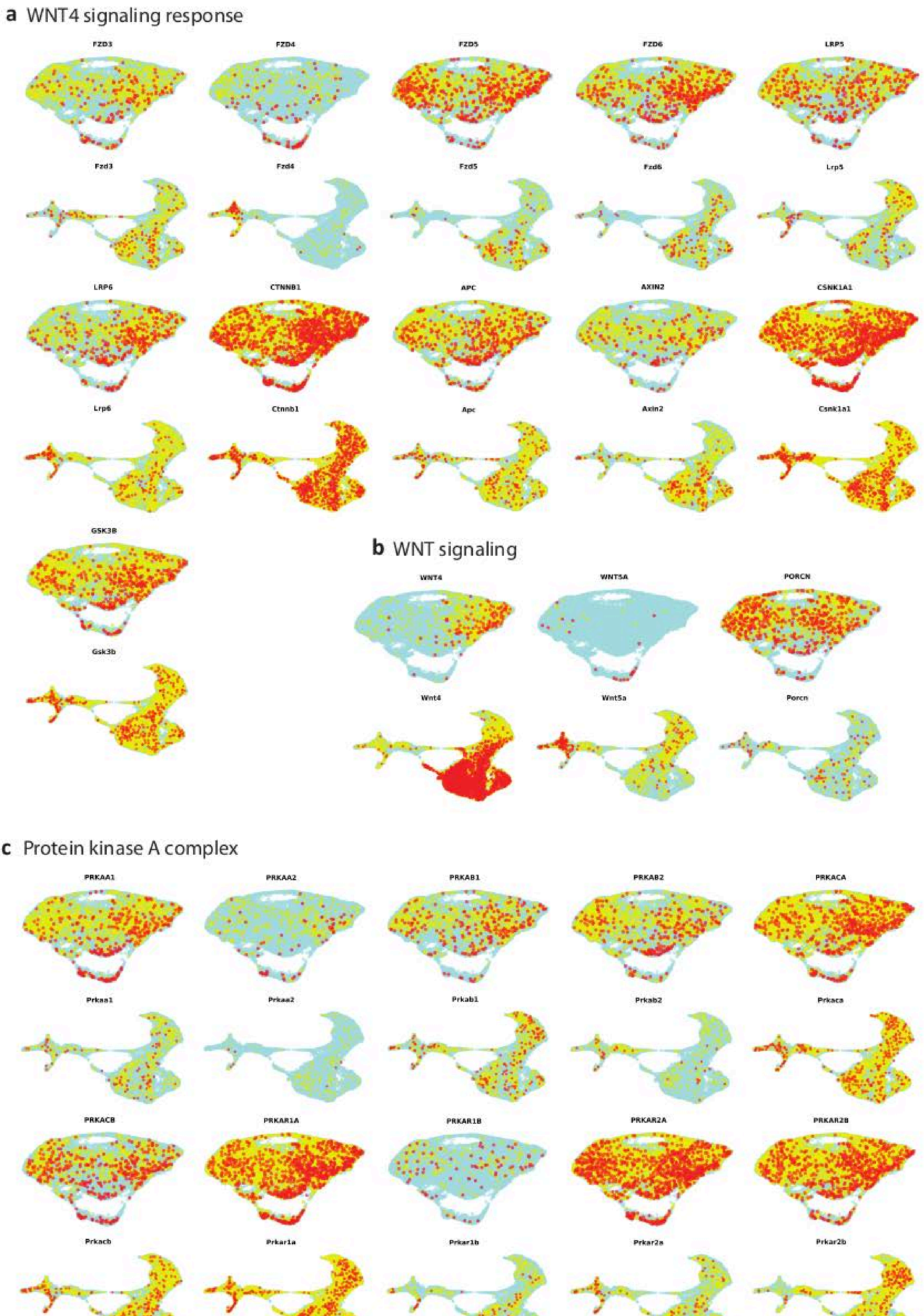

a WNT4 signaling response
FZD3
FZD4
FZD5
FZD6
LRP5
Fzd3
Fzd4
Fzd5
Fzd6
Lrp5
LRP6
CTNNB1
APC
AXIN2
CSNK1A1
Lrp6
Ctnnb1
Apc
Axin2
Csnk1a1
GSK3B
Gsk3b
b WNT signaling
WNT4
WNT5A
PORCN
Wnt4
Wnt5a
Porcn
c Protein kinase A complex
PRKAA1
PRKAA2
PRKAB1
PRKAB2
PRKACA
Prkaa1
Prkaa2
Prkab1
Prkab2
Prkaca
PRKACB
PRKAR1A
PRKAR1B
PRKAR2A
PRKAR2B
Prkacb
Prkar1a
Prkar1b
Prkar2a
Prkar2b

Supplementary Fig. 54 | WNT-PKA signaling activity in human and mouse adrenals. a-c, Expression of key genes involved in WNT-PKA signaling on human (above) and mouse (below) embeddings. **a**, Genes, involved in WNT4 signal response (WNT4 receptor and beta-catenin complex). **b**, WNT-ligand expression and its post-translational modification. **c**, Protein kinase A complex (PKA) subunits expression.

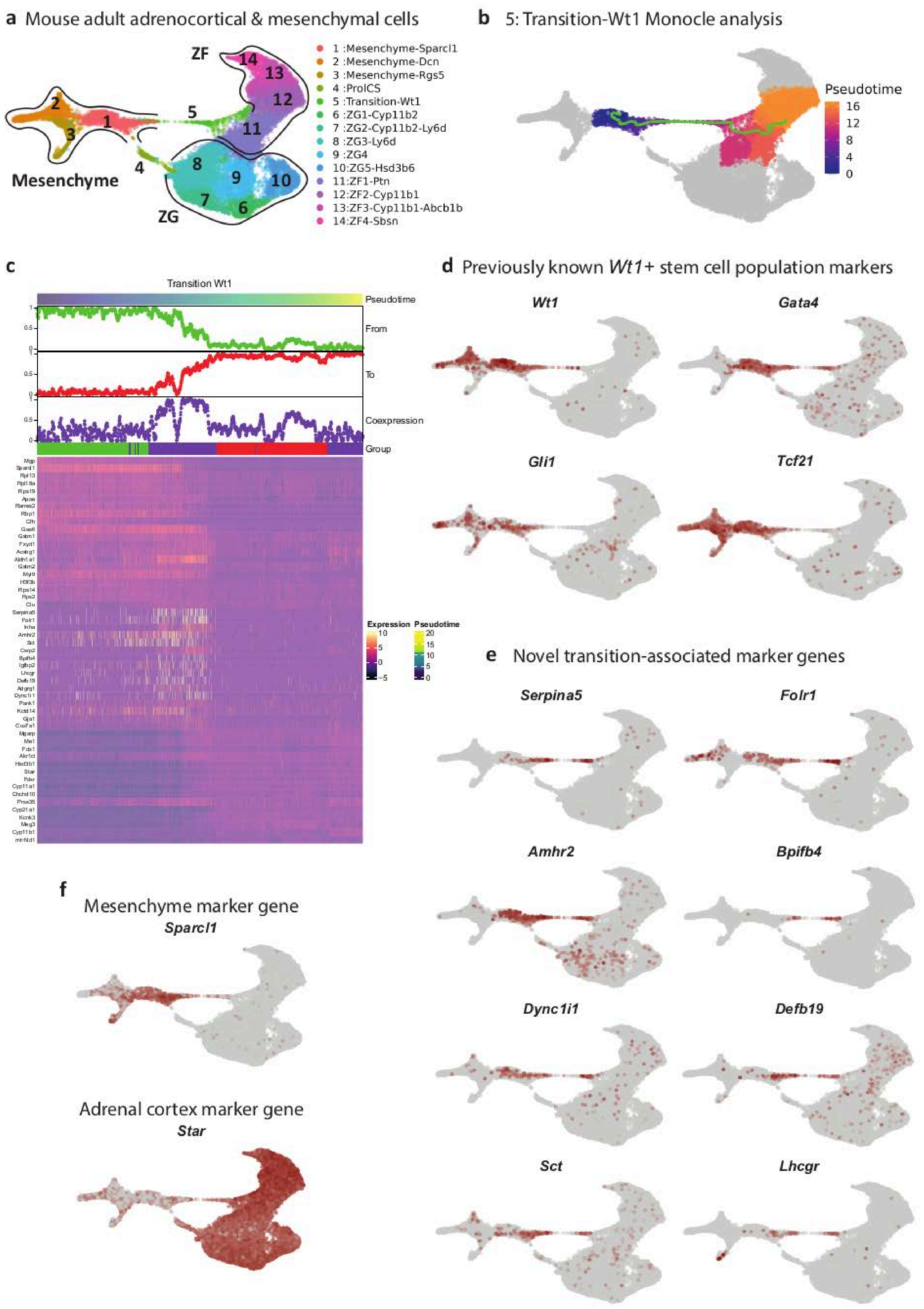
a Mouse adult adrenocortical & mesenchymal cells
ZF
ZG
Mesenchyme
1 :Mesenchyme-Sparcl1
2 :Mesenchyme-Dcn
3 :Mesenchyme-Rgs5
4 :ProlCS
5 :Transition-Wt1
6 :ZG1-Cyp11b2
7 :ZG2-Cyp11b2-Ly6d
8 :ZG3-Ly6d
9 :ZG4
10:ZG5-Hsd3b6
11:ZF1-Ptn
12:ZF2-Cyp11b1
13:ZF3-Cyp11b1-Abcb1b
14:ZF4-Sbsn
b 5: Transition-Wt1 Monocle analysis
Pseudotime
16
12
8
4
0
c
Transition Wt1
Pseudotime
From
To
Coexpression
Group
Expression
Pseudotime
d Previously known Wt1+ stem cell population markers
Wt1
Gata4
Gli1
Tcf21
e Novel transition-associated marker genes
Serpina5
Folr1
Amhr2
Bpifb4
Dync1i1
Defb19
Sct
Lhcgr
f
Mesenchyme marker gene
Sparcl1
Adrenal cortex marker gene
Star

Supplementary Fig. 55 | Transition between Wt1+ mesenchymal stem cells and mouse zona fasciculata. a, Mouse adult adrenocortical and mesenchymal cells embedding (25,932 cells). **b**, Corresponding trajectory and pseudotime analysis. **c**, Heatmap of gene expression dynamics in the transition. From top to bottom, first panel, pseudotime during transition. Second panel, expression magnitude of From (1:Mesenchyme-Sparcl1) gene expression program in selected cells. Third panel, expression magnitude of To (11:ZF1-Ptn) gene expression programs in selected cells. Fourth panel, coexpression of the two programs. Fifth panel, transition part, green - From, red - To, dark orchid - transition. Sixth panel, expression magnitude of the selected genes, visualized as a heatmap. **d**, Previously known *Wt1+* stem cell population marker genes expression. **e**, Novel transition-associated maker genes expression. **f**, Mesenchyme and adrenal cortex marker genes expression.

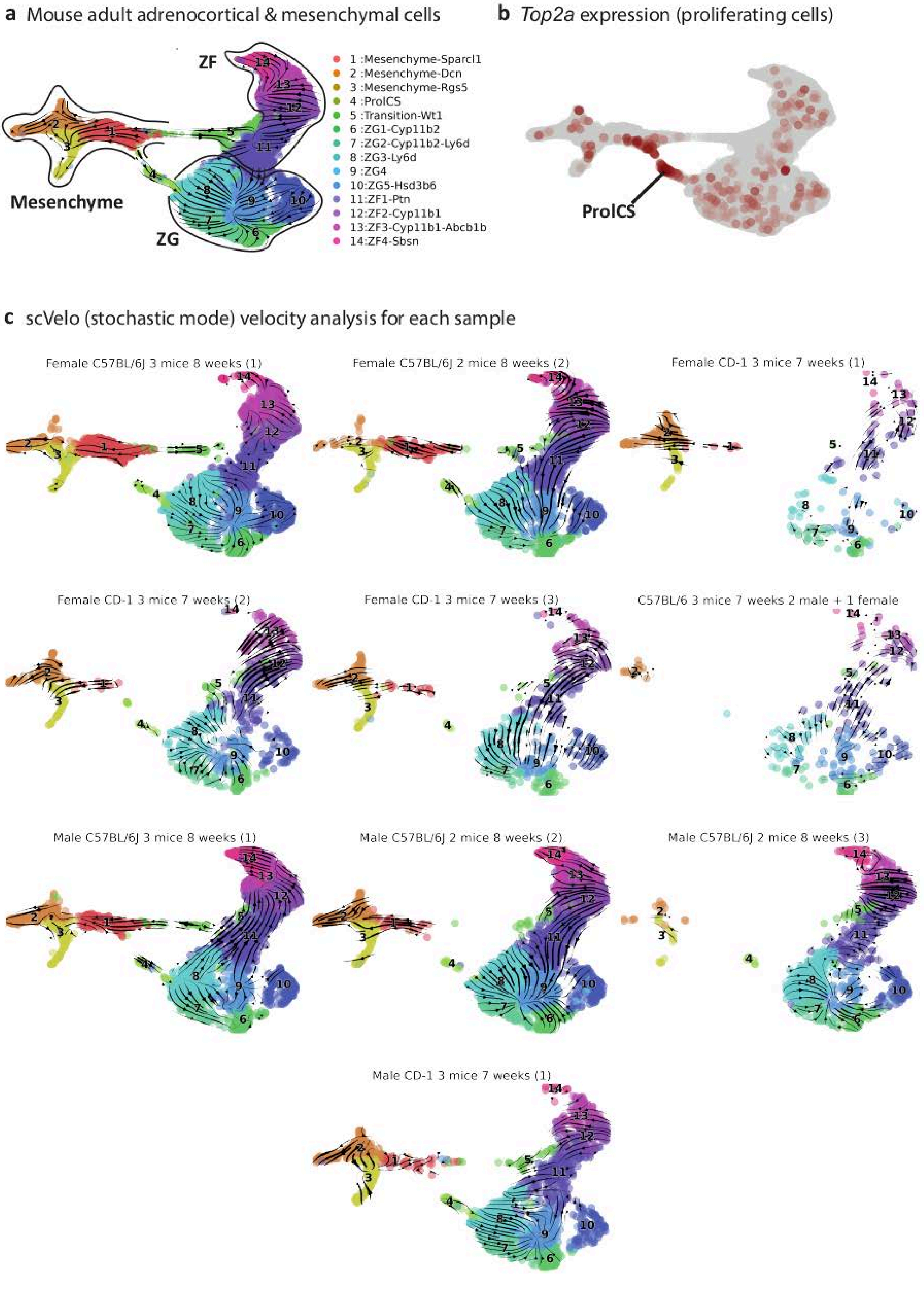
a Mouse adult adrenocortical & mesenchymal cells
ZF
Mesenchyme
ZG
1 :Mesenchyme-Sparcl1
2 :Mesenchyme-Dcn
3 :Mesenchyme-Rgs5
4 :ProlCS
5 :Transition-Wt1
6 :ZG1-Cyp11b2
7 :ZG2-Cyp11b2-Ly6d
8 :ZG3-Ly6d
9 :ZG4
10:ZG5-Hsd3b6
11:ZF1-Ptn
12:ZF2-Cyp11b1
13:ZF3-Cyp11b1-Abcb1b
14:ZF4-Sbsn
b Top2a expression (proliferating cells)
ProlCS
c scVelo (stochastic mode) velocity analysis for each sample
Female C57BL/6J 3 mice 8 weeks (1)
Female C57BL/6J 2 mice 8 weeks (2)
Female CD-1 3 mice 7 weeks (1)
Female CD-1 3 mice 7 weeks (2)
Female CD-1 3 mice 7 weeks (3)
C57BL/6 3 mice 7 weeks 2 male + 1 female
Male C57BL/6J 3 mice 8 weeks (1)
Male C57BL/6J 2 mice 8 weeks (2)
Male C57BL/6J 2 mice 8 weeks (3)
Male CD-1 3 mice 7 weeks (1)

Supplementary Fig. 56 | Velocity analysis of mouse adult adrenal samples. a, Mouse adult adrenocortical and mesenchymal cells embedding (18,534 cells, only samples obtained in the current study were included in this analysis). **b**, *Top2a* gene expression indicates location of proliferating cells on UMAP. **c**, Velocity analysis for each individual sample projected on joint embedding.

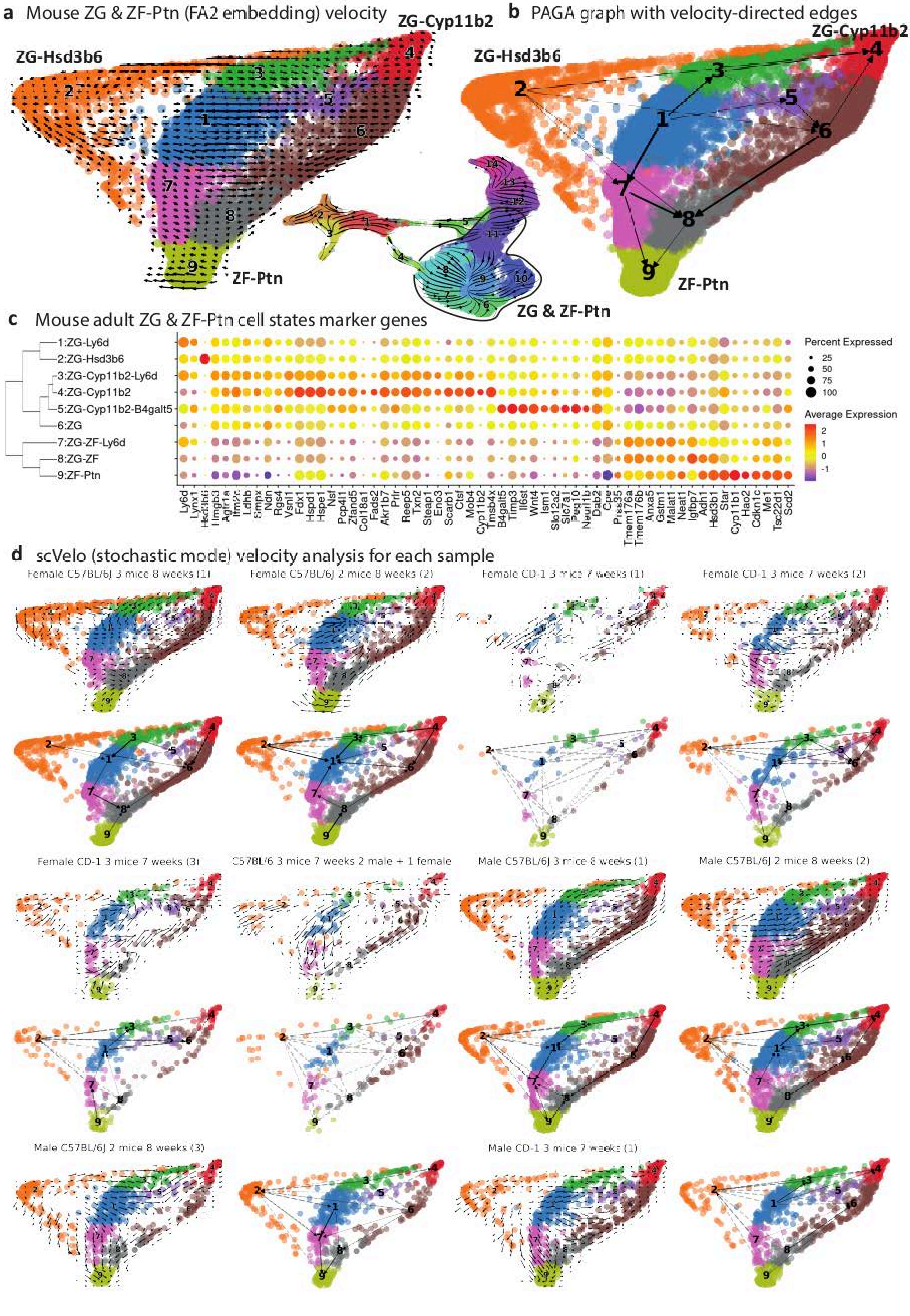
a Mouse ZG & ZF-Ptn (FA2 embedding) velocity
ZG-Hsd3b6
ZG-Cyp11b2
ZF-Ptn
ZG & ZF-Ptn
b PAGA graph with velocity-directed edges
ZG-Hsd3b6
ZG-Cyp11b2
ZF-Ptn
c Mouse adult ZG & ZF-Ptn cell states marker genes
1:ZG-Ly6d
2:ZG-Hsd3b6
3:ZG-Cyp11b2-Ly6d
4:ZG-Cyp11b2
5:ZG-Cyp11b2-B4galt5
6:ZG
7:ZG-ZF-Ly6d
8:ZG-ZF
9:ZF-Ptn
Ly6d Lynx1 Hsd3b6 Hmgb3 Agtr1a Itm2c Ldhb Smpx Ndn Rgs4 Vsnl1 Fdx1 Hspd1 Hspe1 Nsf Pcp4l1 Zfand5 Col18a1 Fads2 Akr1b7 Prlr Reep5 Txn2 Steap1 Eno3 Scarb1 Ctsf Mob4 Cyp11b2 Tmsb4x B4galt5 Timp3 Il6st Wnt4 Ism1 Slc12a2 Slc7a1 Peg10 Neurl1b Dab2 Cpe Prss35 Tmem176a Tmem176b Anxa5 Gstm1 Malat1 Neat1 Igfbp7 Adh1 Hsd3b1 Star Cyp11b1 Hao2 Cdkn1c Me1 Tsc22d1 Scd2
Percent Expressed
25
50
75
100
Average Expression
2
1
0
-1
d scVelo (stochastic mode) velocity analysis for each sample
Female C57BL/6J 3 mice 8 weeks (1)
Female C57BL/6J 2 mice 8 weeks (2)
Female CD-1 3 mice 7 weeks (1)
Female CD-1 3 mice 7 weeks (2)
Female CD-1 3 mice 7 weeks (3)
C57BL/6 3 mice 7 weeks 2 male + 1 female
Male C57BL/6J 3 mice 8 weeks (1)
Male C57BL/6J 2 mice 8 weeks (2)
Male C57BL/6J 2 mice 8 weeks (3)
Male CD-1 3 mice 7 weeks (1)

Supplementary Fig. 57 | Putative transitions between mouse zona glomerulosa clusters. **a**, A ForceAtlas2 (FA2) embedding illustrating zona glomerulosa (ZG) and early zona fasciculata ZF-Ptn cluster (15,624 cells). Clusters were obtained from reclustering to increase local resolution. The velocity analysis was performed on all samples as a merged count matrix and only on cells shown. **b**, PAGA graph with velocity-directed edges, which indicate the putative direction of transitions between clusters. The focused blue cluster 1:ZG-Ly6d (source of transitions) approximately corresponds to cluster 8:ZG3-Ly6d on the original embedding. **c**, DotPlot displaying cluster-specific marker genes. The hierarchy of clusters, based on transcriptional similarity, is shown on the left. **d**, Velocity analysis for each individual sample projected on joint embedding. For each sample two representations are provided: velocity direction with arrows and PAGA graph with velocity-directed edges.

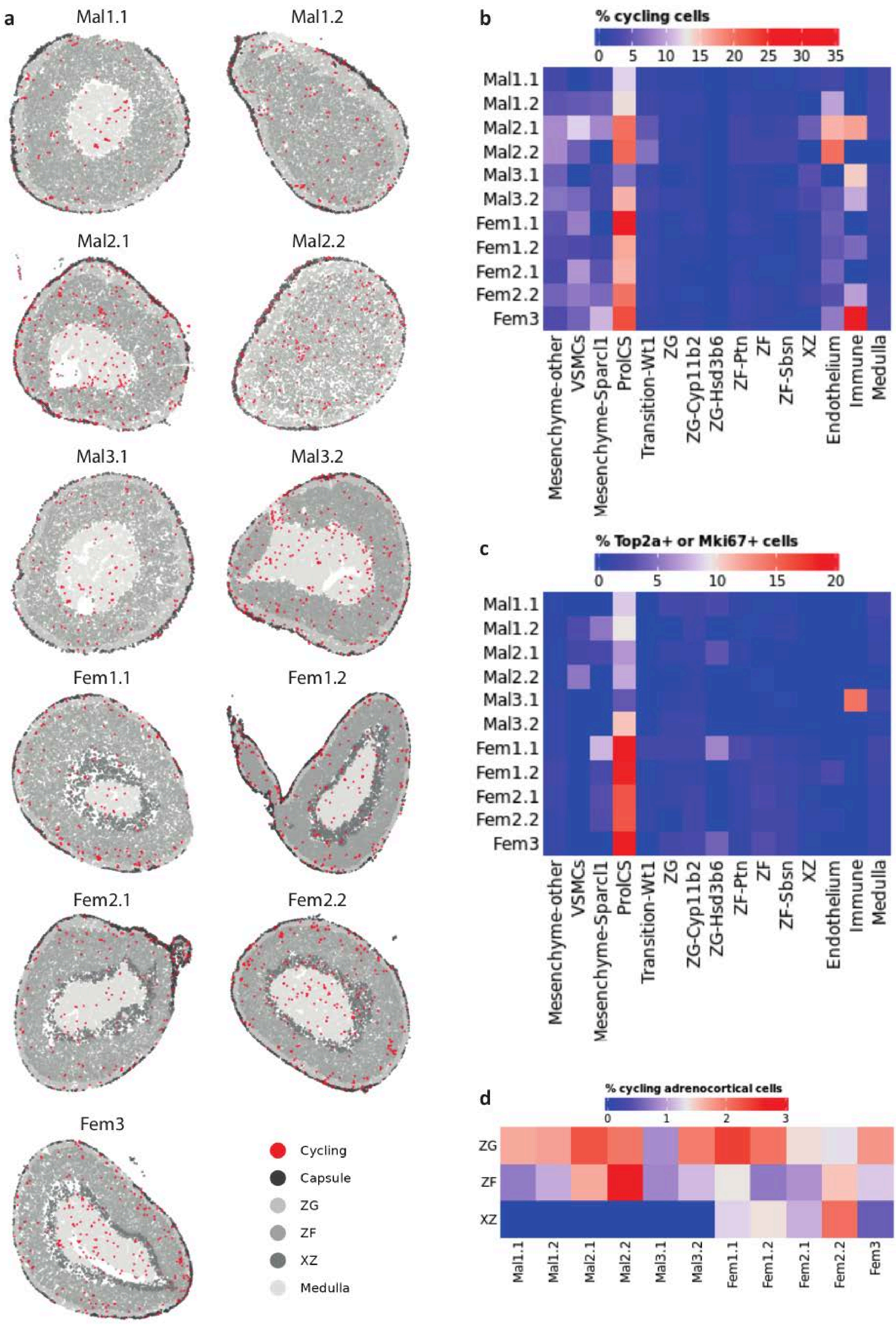
a
Mal1.1
Mal1.2
Mal2.1
Mal2.2
Mal3.1
Mal3.2
Fem1.1
Fem1.2
Fem2.1
Fem2.2
Fem3
Cycling
Capsule
ZG
ZF
XZ
Medulla
b
% cycling cells
0 5 10 15 20 25 30 35
Mal1.1
Mal1.2
Mal2.1
Mal2.2
Mal3.1
Mal3.2
Fem1.1
Fem1.2
Fem2.1
Fem2.2
Fem3
Mesenchyme-other
VSMCs
Mesenchyme-Sparcl1
ProlCS
Transition-Wt1
ZG
ZG-Cyp11b2
ZG-Hsd3b6
ZF-Ptn
ZF
ZF-Sbsn
XZ
Endothelium
Immune
Medulla
c
% Top2a+ or Mki67+ cells
0 5 10 15 20
Mal1.1
Mal1.2
Mal2.1
Mal2.2
Mal3.1
Mal3.2
Fem1.1
Fem1.2
Fem2.1
Fem2.2
Fem3
Mesenchyme-other
VSMCs
Mesenchyme-Sparcl1
ProlCS
Transition-Wt1
ZG
ZG-Cyp11b2
ZG-Hsd3b6
ZF-Ptn
ZF
ZF-Sbsn
XZ
Endothelium
Immune
Medulla
d
% cycling adrenocortical cells
0 1 2 3
ZG
ZF
XZ
Mal1.1
Mal1.2
Mal2.1
Mal2.2
Mal3.1
Mal3.2
Fem1.1
Fem1.2
Fem2.1
Fem2.2
Fem3

Supplementary Fig. 58 | Spatial distribution of cycling cells in mouse VisiumHD data. **a**, Cycling cells (defined as cells in G2M or S phases) on whole tissue sections. **b-c**, Heatmaps representing for each sample which percent of cells of each cell type were identified as cycling. **b**, Cycling cells were identified using AUCell and mitotic signature (see Methods section). **c**, Cycling cells were identified as those having *Mki67* or *Top2a* genes expression above zero. **d**, Heatmap representing for each sample which percent of cells of each zone were identified as cycling. Only cells annotated as adrenocortical included (i.e. without mesenchyme, endothelium etc.). Cycling cells were identified using AUCell and mitotic signature (see Methods section). ZG, zona glomerulosa; ZF, zona fasciculata; ZR, zona reticularis.

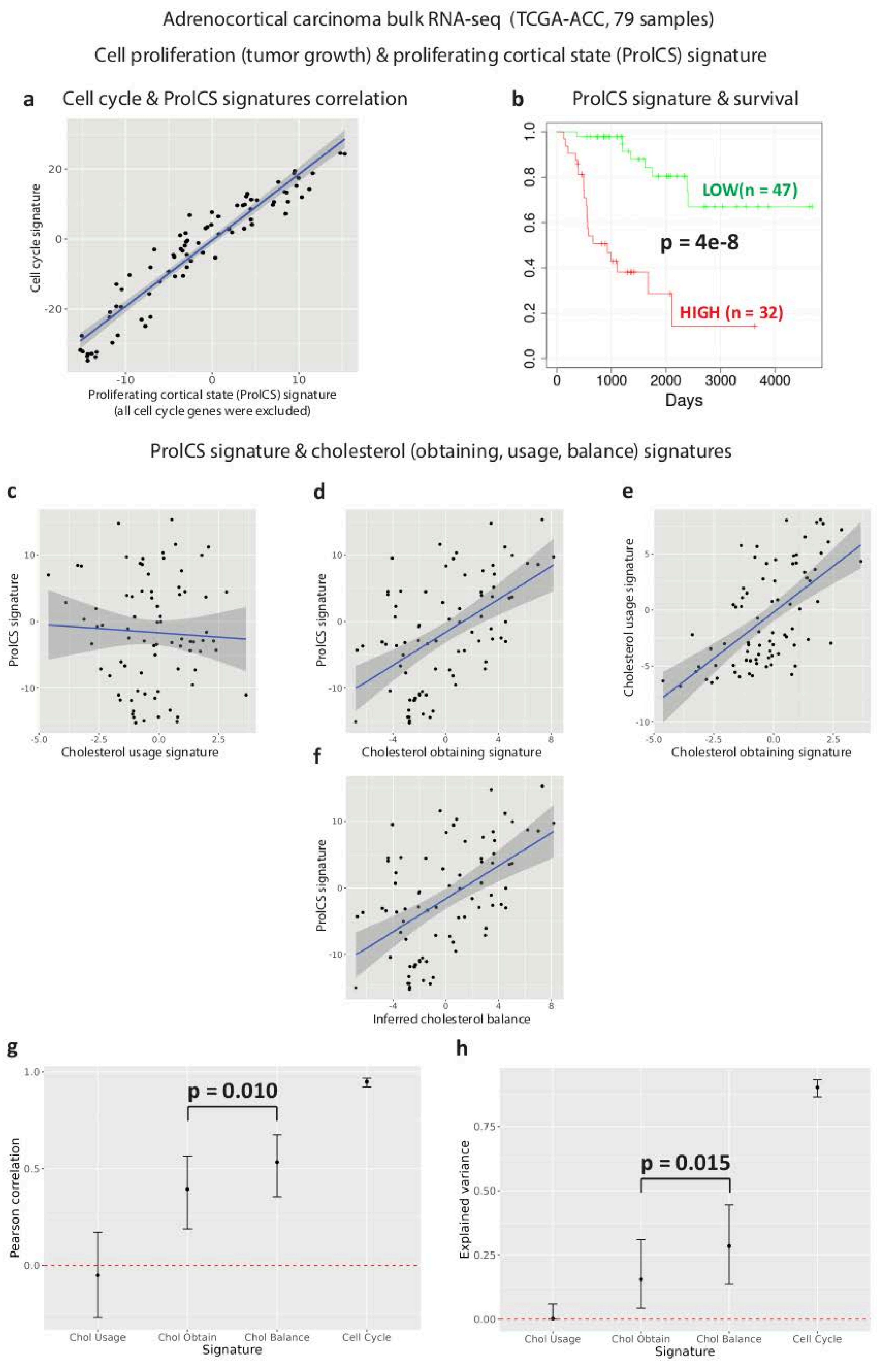
Adrenocortical carcinoma bulk RNA-seq (TCGA-ACC, 79 samples)
Cell proliferation (tumor growth) & proliferating cortical state (ProlCS) signature
a
Cell cycle & ProlCS signatures correlation
Cell cycle signature
Proliferating cortical state (ProlCS) signature
(all cell cycle genes were excluded)
b
ProlCS signature & survival
LOW(n = 47)
p = 4e-8
HIGH (n = 32)
Days
ProlCS signature & cholesterol (obtaining, usage, balance) signatures
c
ProlCS signature
Cholesterol usage signature
d
ProlCS signature
Cholesterol obtaining signature
e
Cholesterol usage signature
Cholesterol obtaining signature
f
ProlCS signature
Inferred cholesterol balance
g
Pearson correlation
p = 0.010
Chol Usage
Chol Obtain
Chol Balance
Cell Cycle
Signature
h
Explained variance
p = 0.015
Chol Usage
Chol Obtain
Chol Balance
Cell Cycle
Signature

Supplementary Fig. 59 | Correlation between cholesterol balance and proliferation activity in adrenocortical carcinoma bulk RNA-seq. a, **c-f,** Linear correlations between various pairs of scores. Each dot corresponds to an adrenocortical carcinoma bulk RNA-seq sample (n = 79). **a**, High correlation between proliferating cortical state (ProlCS) cells signature (doesn't include cell cycle associated genes) expression score and cell cycle marker genes expression score points to the key role of ProlCS in tumor growth (r = 0.95, p-value < 2.2e-16, 95% CI = [0.92, 0.97]). **b**, Association between ProlCS signature expression and survival probability. P-value is shown for two-sided log-rank tests between Kaplan–Meier curves for tumors with more (HIGH group, n = 33) or less (LOW group, n = 47) than average signature gene expression score. **c**, Cholesterol usage signature expression score and ProlCS signature expression score (r = -0.05, p-value > 0.6). **d**, Cholesterol obtaining signature expression score and ProlCS signature expression score (r = 0.40, p-value = 3.3e-4, 95% CI = [0.19, 0.57]). **e**, High Pearson correlation (r = 0.61, p-value = 3.3e-9, 95% CI = [0.44, 0.73]) between cholesterol obtaining and usage signatures expression scores gives a basis for regression out the usage signature and cholesterol balance score calculation. **f**, Cholesterol balance score and ProlCS signature expression score (r = 0.53, p-value = 4.0e-07, 95% CI = [0.35, 0.68]). **g**, Pearson correlations values between ProlCS signature expression score and cholesterol usage, cholesterol obtaining, cholesterol balance, cell cycle scores. For all correlations Fisher 95% confidence intervals are shown as error bars. Statistical significance of difference between obtaining and balance correlations (overlapping samples) was calculated using bootstrap. **h**, Proportions of ProlCS signature expression score variance explained by linear models with cholesterol usage, cholesterol obtaining, cholesterol balance or cell cycle score as a covariate. For all values bootstrap 95% confidence intervals are shown as error bars. Statistical significance of difference between obtaining and balance values was also calculated using bootstrap. ProlCS, proliferating cortical state
