# Supplementary material for: Human and mouse adrenal glands are characterized by species-specific steroidogenic states and tissue turnover
Source: Nat Genet. 2026 Sep 8;58(9):2270–83. doi: 10.1038/s41588-026-02737-1 (PMC13553300; doi:10.1038/s41588-026-02737-1)
Supplement: Supplementary file 2 [file 41588_2026_2737_MOESM2_ESM.pdf]

Corresponding author(s): Pavel Volchkov, Peter V. Kharchenko and Igor Adameyko

Last updated by author(s): May 19, 2026

Reporting Summary

Nature Portfolio wishes to improve the reproducibility of the work that we publish. This form provides structure for consistency and transparency in reporting. For further information on Nature Portfolio policies, see our Editorial Policies and the Editorial Policy Checklist.

Statistics

For all statistical analyses, confirm that the following items are present in the figure legend, table legend, main text, or Methods section.

n/a	Confirmed	
☐	☒	The exact sample size (*n*) for each experimental group/condition, given as a discrete number and unit of measurement
☐	☒	A statement on whether measurements were taken from distinct samples or whether the same sample was measured repeatedly
☐	☒	The statistical test(s) used AND whether they are one- or two-sided<br>*Only common tests should be described solely by name; describe more complex techniques in the Methods section.*
☐	☒	A description of all covariates tested
☐	☒	A description of any assumptions or corrections, such as tests of normality and adjustment for multiple comparisons
☐	☒	A full description of the statistical parameters including central tendency (e.g. means) or other basic estimates (e.g. regression coefficient) AND variation (e.g. standard deviation) or associated estimates of uncertainty (e.g. confidence intervals)
☐	☒	For null hypothesis testing, the test statistic (e.g. *F*, *t*, *r*) with confidence intervals, effect sizes, degrees of freedom and *P* value noted<br>*Give P values as exact values whenever suitable.*
☒	☐	For Bayesian analysis, information on the choice of priors and Markov chain Monte Carlo settings
☒	☐	For hierarchical and complex designs, identification of the appropriate level for tests and full reporting of outcomes
☐	☒	Estimates of effect sizes (e.g. Cohen's *d*, Pearson's *r*), indicating how they were calculated

Our web collection on statistics for biologists contains articles on many of the points above.

Software and code

Policy information about availability of computer code

Data collection	Single cell data sets were generated from human adrenal gland samples from eleven donors and spatial transcriptomics from seven donors.<br><br>Wild type mouse adrenal gland data sets were generated from six animals for spatial transcriptomics and eleven animals for single cell RNA-sequencing (males and females analyzed separately), with sample size determined by the requirement for sufficient cell yield following pooling of 4–6 glands per sample.<br><br>To access bulk RNA-seq data from GTEx cohort R package recont3 v.1.0.7 has been used. Access to some gene annotation entities (GO categories, chromosomal location, interspecies homology) was performed via R package biomaRt v.2.54.0.<br><br>Images acquired as z-stacks using a ZEISS confocal microscope (LSM780) using 10x and 20x objectives, and z-stacks projected into maximum projection pictures using FIJI image processing software (ImageJ 1.53t) and saved as jpegs.
Data analysis	For 10x Chromium scRNA-seq and Visium low resolution spatial transcriptomics raw sequencing data processing were used reference genomes refdata-gex-GRCh38-2020-A (human) and refdata-gex-mm10-2020-A (mouse) from 10x Genomics web site (https://www.10xgenomics.com/) and software bcl2fastq v.2.20.422, Cell Ranger v.7.0.0, spaceranger v.2.0.0.<br>For Stereo-seq human high resolution spatial transcriptomics raw sequencing data processing were used GRCh38 reference genome and software SAW v.7.0.0, the Stereopy v.1.1.0 python package, script h5ad2rds.R from Stereopy github.<br>For VisiumHD mouse high resolution spatial transcriptomics raw sequencing data processing were used refdata-gex-mm10-2020-A & Visium_Mouse_Transcriptome_Probe_Set_v2.0_mm10-2020-A.csv reference genome and software spaceranger v.3.0.1, bin2cell v.0.3.0 python package.

Downstream analysis has been performed in R 4.2.2 with packages Seurat v.4.3.0, pagoda2 v.1.0.10, Scrublet v.0.2.3, SoupX v.1.6.2, Harmony v.0.1.1, biomaRt v.2.54.0, conos v.1.5.0, cacoa v.0.4.0, DOSE v.3.24.2, clusterProfiler v.4.6.2, monocle3 v.1.3.3, lme4 v.1.1-34, corto v.1.2.2, boot v.1.3-28, survival v.3.4-0, DESeq2 v.1.38.3, spacexr v.2.2.1, ReductionWrappers v.2.5.4, reticulate v.1.28 (for fa2 v.0.3.5 package with Python v.3.8.20), clusterProfiler v.4.6.2, recount3 v.1.0.7, recount v.1.28.0, Giotto v.1.1.2, Banksy v.0.99.13, AUCell v.1.24.0

Velocity analysis has been perfoemed in python 3.9.7 with velocyto.py v.0.17.17 and scVelo v.0.2.4 package.

Source code is available at gitlab https://gitlab.com/MaksimovDenis/adrenalglandscatlas and Zenodo https://doi.org/10.5281/zenodo.20258071.

Figures with fluorescent images were assembled using Adobe Photoshop (version 26.4.1) and Illustrator (version 29.3.1).

For manuscripts utilizing custom algorithms or software that are central to the research but not yet described in published literature, software must be made available to editors and reviewers. We strongly encourage code deposition in a community repository (e.g. GitHub). See the Nature Portfolio guidelines for submitting code & software for further information.

Data

Policy information about availability of data

All manuscripts must include a data availability statement. This statement should provide the following information, where applicable:

- Accession codes, unique identifiers, or web links for publicly available datasets
- A description of any restrictions on data availability
- For clinical datasets or third party data, please ensure that the statement adheres to our policy

Sequenced reads of mouse scRNA-seq and VisiumHD samples, h5 files with gene counts in individual cells or spots and all data related to Visium/VisiumHD/Stereo-Seq spatial transcriptomics (coordinate matrices, slide images etc.) were deposited under GEO accession number GSE253852. Sequenced reads of human scRNA-seq, Visium and Stereo-seq spatial transcriptomics were deposited under EGA controllable access EGAC00001003383. We provide interactive access to the data via CellxGene74–76 (detailed list and links in Supplementary Notes)
https://adameykolab.hifo.meduniwien.ac.at/cellxgene_public/filecrawl/2026_NatGen_Kastriti_Maksimov
Human fetal scRNA-seq data were obtained from the following sources: Kameneva et al.22 processed Seurat object via the link from github https://github.com/artem-artemov/adrenal (http://pklab.med.harvard.edu/artem/adrenal/data/Seurat/adrenal.human.seurat.scrublet.rds); Jansky et al.23 count matrix and annotation from https://adrenal.kitz-heidelberg.de/developmental_programs_NB_viz/ , Kildisiute et al.24 processed Seurat object from https://www.neuroblastomacellatlas.org/, Han et al.25 samples count matrices from GSE134355. Previously published mouse adult scRNA-seq samples count matrices were downloaded from GSE134355 and GSE16175125,31. Normal adrenal gland bulk RNA-seq data from GTEx cohort15 were obtained via recount3 v.1.0.7 R package65. Adrenocortical carcinoma bulk RNA-seq and metadata were downloaded from the TCGA portal https://portal.gdc.cancer.gov/projects/TCGA-ACC.

Research involving human participants, their data, or biological material

Policy information about studies with human participants or human data. See also policy information about sex, gender (identity/presentation), and sexual orientation and race, ethnicity and racism.

Reporting on sex and gender	The study included human adrenal glands from 9 female (4 scRNA-seq, 1 scRNA-seq+Histology, 2 Visium, 1 Stereo-seq +Histology, 1 Histology) and 10 male donors (5 scRNA-seq, 1 scRNA-seq+Visium, 1 Visisum, 2 Stereo-seq, 1 Histology). The study design took into account only biological sex. Information about sex was onbatined as clinical metadata and confirmed by XIST gene expression in adrenal gland scRNA-seq data (expressed only in female samples). No gender information was collected.
Reporting on race, ethnicity, or other socially relevant groupings	Race, ethnicity, or any other socially relevant groupings were not considered in the study.
Population characteristics	The study utilised material only from adult (at least 18 years old) donors. The study did not include donors who died from adrenal-related diseases (e.g. adrenal neoplasms).
Recruitment	Human adrenals (one gland per donor) were excised simultaneously with explantation of kidneys from deceased organ donors. During kidney transplantation, adrenals are disposed of and not transplanted. Thus, we made use of tissue that would otherwise be discarded without causing unnecessary distress or complications during the explantation procedure.<br><br>The study included predominantly aged people (8 of 11 scRNA-seq donors wiht age >50 years old), which could potentially lead to bias.
Ethics oversight	The study protocol was approved by the Ethical agreement No 1544/2020, renewed on an annual basis by the Ethical Committee of the Medical University in Vienna, Austria in accordance with the Declaration of Helsinki.

Note that full information on the approval of the study protocol must also be provided in the manuscript.

Field-specific reporting

Please select the one below that is the best fit for your research. If you are not sure, read the appropriate sections before making your selection.

☒ Life sciences ☐ Behavioural & social sciences ☐ Ecological, evolutionary & environmental sciences

Life sciences study design

All studies must disclose on these points even when the disclosure is negative.

Sample size	No statistical test or analyses were used to predetermine sample size. In total 11 human adrenal glands (5 female and 6 male) and 16 mouse (8 female, 7 male and 1 mixed, including those obtained from public sources) were subjected to scRNA-seq analysis, 3 human adrenal sections from 3 individuals (1 female and 2 male) were subjected to Stereo-seq analysis and 11 mouse sectons (5 female and 6 male from 3 female and 3 male animals) were subjected to VisiumHD analysis, which followed standards in the field and allowed to perform an analysis of sexual dimorphism.
Data exclusions	ScRNA-seq data were subjected to multi-step quality control filtering and droplets that did not meet QC criteria were excluded from further analysis (see Methods section). For focused integrations of specific groups of cell types (e.g. adrenocortical, mesenchymal, immune), samples that did not contain sufficient numbers of target cells were excluded, and cells with detection at high level of genes that are markers for other cell types were considered to contain a high amount of ambient RNA and were excluded from integration (see Methods section).
Replication	All adrenal gland samples were analyzed once. The results of scRNA-seq data analysis were consistent between biological replicates - there were no critical compositional differences. Mouse adrenal gland samples were aligned well with previously published samples.
Randomization	No randomization were necessary because no prior treatment of samples with drugs or other interventions were involved in the study. Samples were treated equivalently in the computational analysis.
Blinding	Blinding was not relevant to the study. Samples were treated equivalently in the computational analysis, measurement and data did not require subjective judgement from the investigators.

Reporting for specific materials, systems and methods

We require information from authors about some types of materials, experimental systems and methods used in many studies. Here, indicate whether each material, system or method listed is relevant to your study. If you are not sure if a list item applies to your research, read the appropriate section before selecting a response.

Materials & experimental systems

n/a	Involved in the study	
☐	☒	Antibodies
☒	☐	Eukaryotic cell lines
☒	☐	Palaeontology and archaeology
☐	☒	Animals and other organisms
☒	☐	Clinical data
☒	☐	Dual use research of concern
☒	☐	Plants

Methods

n/a	Involved in the study	
☒	☐	ChIP-seq
☒	☐	Flow cytometry
☒	☐	MRI-based neuroimaging

Antibodies

Antibodies used	anti-VSNL1, Sigma-Aldrich, #MABN762, mouse monoclonal (clone 2D11), LOT 3959519<br>anti-KI67, Zytomed, #RBK027-05, rabbit monoclonal (clone SP6), LOT V223<br>anti-SF1, R&D Systems, #PP-N1665-0C, mouse monoclonal (clone # N1665), LOT B-4<br>anti-CYP11B1/2, Sigma-Aldrich, #HPA049171, rabbit polyclonal, LOT 000020197<br>anti-CYP11B2, Millipore, #MABS1251, mouse monoclonal, clone 41-17B, LOT4206942<br>anti-EZH2, Fisher Scientific, #MA5-18108, mouse monoclonal, clone 144CT2.1.1.5, LOT 79751985<br>anti-HSD3B2, Sigma-Aldrich, #HPA043261, rabbit polyclonal, LOT R39655<br>anti-CYP21A2, Sigma-Aldrich, #HPA053371, LOT 000013564<br>anti-NOV/CCN3, R&D Systems, #AF1640, goat polyclonal, LOT JKB022410A<br>anti-DLK1, Sigma-Aldrich, #HPA062262, polyclonal, LOT 000017636<br>anti-CYP17A1, Abcam, #ab134910, rabbit monoclonal (clone information proprietary), LOT 1018866-22<br>anti-KCNJ5, Fisher Scientific, #PA5-36967, rabbit polyclonal, LOT 79748411
Validation	anti-VSNL1, Sigma-Aldrich, #MABN762, species reactivity: bovine, rat, mouse, human, suitable for immunohistochemistry and western blot.<br>anti-KI67, Zytomed, #RBK027-05, species reactivity: human, designed for localisation of Ki-67 protein in formalin-fixed, paraffin-embedded tissue sections.<br>anti-SF1, R&D Systems, #PP-N1665-0C, species reactivity: human, mouse, rat, tested for immunohistochemistry, direct ELISA and western blot.<br>anti-CYP11B1/2, Sigma-Aldrich, #HPA049171, species reactivity: human, suitable for immunohistochemistry, cross-reacts with CYP11B1<br>anti-CYP11B2, Millipore, #MABS1251, mouse monoclonal, species reactivity: human, mouse (predicted by homology), tested for immunofluorescence, immunohistochemistry, western blot

anti-EZH2, Fisher Scientific, #MA5-18108, mouse monoclonal, clone 144CT2.1.1.5, species reactivity: human, tested for immunohistochemistry, ChIP and western blot.
anti-HSD3B2, Sigma-Aldrich, #HPA043261 , species reactivity: human, suitable for immunohistochemistry, cross-reacts with HSD3B1
anti-CYP21A2, Sigma-Aldrich, #HPA053371, species reactivity: human, suitable for immunohistochemistry
anti-NOV/CCN3, R&D Systems, #AF1640, species reactivity: human, 7% cross-reactivity with mouse in ELISA, designed for use in ELISA and western blot
anti-DLK1, Sigma-Aldrich, #HPA062262, species reactivity: human, suitable for immunohistochemistry
anti-CYP17A1, Abcam, #ab134910, species reactivity: human, mouse, rat, suitable for immunohistochemistry and western blot.
anti-KCNJ5, Fisher Scientific, #PA5-36967, species reactivity: human, mouse, rat, tested for immunohistochemistry and western blot

Animals and other research organisms

Policy information about studies involving animals; ARRIVE guidelines recommended for reporting animal research, and Sex and Gender in Research

Laboratory animals

Mouse adrenal glands were collected from 7-week-old C57BL-CD-1/10SnSlc-H-2aw18/H-2b mice, 7-week-old FVB mice and 6 to 8-week-old C57BL/6J mice. Cells from 4–6 adrenals (collected from 2 or 3 animals) were pooled into one sample, separated according to biological sex.

Wild animals

The study did not involve wild animals.

Reporting on sex

The sex was considered in study design, since the study included sexual dimorphism analysis of mouse healthy adult adrenal glands at single cell level. Sex was assigned accoring to visual inspection of the gonads of adult mice and confirmed by Xist gene expression in adrenal gland scRNA-seq data (expressed only in female samples). For each mouse adrenal scRNA-seq library infromation about sex, strain, number of animals whose adrenal samples pooled, and age is represented on Supplementary Fig. 42 (including jointly analyzed samples from public sources). Overall number of scRNA-seq libraries generated during this study was 5 for females (14 animals in total) and 5 for males (13 animas in total). One more scRNA-seq library of C57BL/6J mice included both male and female samples and was exluded from sexual dimorphism analysis, but not other analyses. For VisiumHD spatial transcriptomics analysis 5 female amd 6 male sections from 3 feamle and 3 male C57Bl/6J mouse animals were utilised.

Field-collected samples

The study did not involve samples collected from the field.

Ethics oversight

Part of the mouse work (C57/Bl6j genetic background, 8-10 weeks old) was conducted in compliance to European Union regulations and ethical permit approved by the Austrian Ministry of Science, Research and Technology (Bundesministerium für Wissenschaft, Forschung und Wirtschaft, ethical permit number BMWFW-66.009/0018-WF/V/3b/2017). Additional work with C57BL-CD-1/10SnSlc-H-2aw18/H-2b and FVB mice was performed in compliance with Directive 2010/63/EU and the recommendations of the local Bioethical Committee of Lomonosov Moscow State University.

Note that full information on the approval of the study protocol must also be provided in the manuscript.

Plants

Seed stocks

Report on the source of all seed stocks or other plant material used. If applicable, state the seed stock centre and catalogue number. If plant specimens were collected from the field, describe the collection location, date and sampling procedures.

Novel plant genotypes

Describe the methods by which all novel plant genotypes were produced. This includes those generated by transgenic approaches, gene editing, chemical/radiation-based mutagenesis and hybridization. For transgenic lines, describe the transformation method, the number of independent lines analyzed and the generation upon which experiments were performed. For gene-edited lines, describe the editor used, the endogenous sequence targeted for editing, the targeting guide RNA sequence (if applicable) and how the editor was applied.

Authentication

Describe any authentication procedures for each seed stock used or novel genotype generated. Describe any experiments used to assess the effect of a mutation and, where applicable, how potential secondary effects (e.g. second site T-DNA insertions, mosiacism, off-target gene editing) were examined.
