# Supplementary material for: Human and mouse adrenal glands are characterized by species-specific steroidogenic states and tissue turnover
Source: Nat Genet. 2026 Sep 8;58(9):2270–83. doi: 10.1038/s41588-026-02737-1 (PMC13553300; doi:10.1038/s41588-026-02737-1)
Supplement: Supplementary file 3 [file 41588_2026_2737_MOESM3_ESM.pdf]

Peer Review File

Human and mouse adrenal glands are characterized by species-specific steroidogenic states and tissue turnover

Corresponding Author: Professor Igor Adameyko

This file contains all editorial decision letters in order by version, followed by all author rebuttals in order by version.

Version 0:

Decision Letter:

17th Apr 2024

Dear Professor Adameyko,

hope this email finds you well.

Your Article, "Comparative transcriptomics of developing and adult human and mouse adrenal glands reveals novel cellular heterogeneity and tissue microniches" has now been seen by 3 referees. You will see from their comments copied below that while they find your work of considerable potential interest, they have raised quite substantial concerns that must be addressed. In light of these comments, we cannot accept the manuscript for publication, but would be very interested in considering a revised version that addresses these serious concerns.

We hope you will find the referees' comments useful as you decide how to proceed. If you wish to submit a substantially revised manuscript, please bear in mind that we will be reluctant to approach the referees again in the absence of major revisions.

To guide the scope of the revisions, the editors discuss the referee reports in detail within the team, including with the chief editor, with a view to identifying key priorities that should be addressed in revision and sometimes overruling referee requests that are deemed beyond the scope of the current study. In this case, we would like you to address Reviewers´ comments in full. Please do not hesitate to get in touch if you would like to discuss these issues further.

If you choose to revise your manuscript taking into account all reviewer and editor comments, please highlight all changes in the manuscript text file. At this stage we will need you to upload a copy of the manuscript in MS Word .docx or similar editable format.

We are committed to providing a fair and constructive peer-review process. Do not hesitate to contact us if there are specific requests from the reviewers that you believe are technically impossible or unlikely to yield a meaningful outcome.

If revising your manuscript:

*1) Include a “Response to referees” document detailing, point-by-point, how you addressed each referee comment. If no action was taken to address a point, you must provide a compelling argument. This response will be sent back to the referees along with the revised manuscript.

*2) If you have not done so already please begin to revise your manuscript so that it conforms to our Article format instructions, available here.
Refer also to any guidelines provided in this letter.

*3) Include a revised version of any required Reporting Summary: https://www.nature.com/documents/nr-reporting-summary.pdf
It will be available to referees (and, potentially, statisticians) to aid in their evaluation if the manuscript goes back for peer review.

A revised checklist is essential for re-review of the paper.

Please be aware of our guidelines on digital image standards.

You may use the link below to submit your revised manuscript and related files:

Link Redacted

Note: This URL links to your confidential home page and associated information about manuscripts you may have submitted, or that you are reviewing for us. If you wish to forward this email to co-authors, please delete the link to your homepage.

If you wish to submit a suitably revised manuscript we would hope to receive it within 6 months. If you cannot send it within this time, please let us know. We will be happy to consider your revision so long as nothing similar has been accepted for publication at Nature Genetics or published elsewhere. Should your manuscript be substantially delayed without notifying us in advance and your article is eventually published, the received date would be that of the revised, not the original, version.

Please do not hesitate to contact me if you have any questions or would like to discuss the required revisions further.

Nature Genetics is committed to improving transparency in authorship. As part of our efforts in this direction, we are now requesting that all authors identified as 'corresponding author' on published papers create and link their Open Researcher and Contributor Identifier (ORCID) with their account on the Manuscript Tracking System (MTS), prior to acceptance. ORCID helps the scientific community achieve unambiguous attribution of all scholarly contributions. You can create and link your ORCID from the home page of the MTS by clicking on 'Modify my Springer Nature account'. For more information please visit please visit www.springernature.com/orcid.

Thank you for the opportunity to review your work.

Sincerely,
Chiara

Chiara Anania, PhD
Associate Editor
Nature Genetics
https://orcid.org/0000-0003-1549-4157

Referee expertise:

Referee #1: computational; spatial transcriptomics

Referee #2: single-cell genomics; adrenal gland biology

Referee #3: sex-related differences adrenal gland

Reviewers' Comments:

Reviewer #1:
Remarks to the Author:
This study employs single-cell and spatial RNA-seq to investigate the diversity and self-renewal mechanisms of human adrenocortical cells, utilizing both adult and fetal human adrenal samples. The author identified new progenitor cells and highlight difference in human and murine adrenal gland.

Comments on the manuscript:

Upon reviewing the results and discussion, it's evident that the mechanisms about human adrenocortical cells are a primary focus for the authors. However, the narrative lacks coherence, particularly regarding the rationale behind the study design, which appears complex and extensive. The inclusion of fetal data is briefly mentioned in the discussion section, rather than being clearly articulated earlier. Also, the exploration on self-renewal reveals gender differences but lacks organization. In the abstract the authors say "Intriguingly, our comparisons between human and murine adrenal glands demonstrated reduced zona glomerulosa cell type heterogeneity in mice, which questions the applicability of mouse models in studies of human adrenal disorders." But it is not well supported in the main text. Most of the conclusions were draw from scRNAseq data but lack supporting evidence from other experiments such as functional assay or imaging. For example, "cell heterogeneity" has been extensively used throughout the manuscript, but mostly just from increased number of clusters, which can be a result of changing clustering parameters in data analysis. Overall, the data analysis human adrenal gland, mouse adrenal gland and human fetal adrenal gland seems discrete and does not form a complete story. The key message is unclear and does not provide enough novelty compared to similar recent studies

(https://www.biorxiv.org/content/10.1101/2022.08.27.505530v1, https://insight.jci.org/articles/view/168177 and https://academic.oup.com/jes/article/7/12/bvad131/7329838).

Minor comments:

1) Cluster 15, identified as ZF-CPB1, exhibits a transition-like form on the UMAP, yet this observation remains unaddressed in the section exploring the Origins of Cortical Renewal. While, Clusters 9 and 10 in Figure 2 are designated as transition cells but lack clear explanations why they are labelled as transition cells. Additionally, the rationale behind the three pairs mentioned in the section 'Deciphering the Origins of Cortical Renewal in the Human Adult Adrenal Gland' requires elucidation.
2) The analysis of focused clusters in Figure 3 utilizes color to show relationships between clusters, but clearer plots are needed to illustrate correspondence. Consider applying this new representation to other sections as well.
3) Are there differences between male and female in deciphering the origins of cortical renewal in the human adult adrenal gland?
4) Figure 4 d and e are not discussed, and several plots in the manuscript are overlooked. Reorganization is necessary.
5) In Figure 4c, it's unclear how transitions in human and mice were compared. Further explanation is needed to understand how unique transitions were identified in each species.
6) In Figure 6b, the depiction of the renewal process based on study results is informative, yet the relationship between ZF, ZG, and ZR remains obscure. It is crucial to clarify how these conclusions were derived from bioinformatics analysis. I believe the process involves examining whether transitions exist within minor clusters exhibiting transition-like expression patterns and considering their locations on the UMAP. But rationale behind this is needed and are there any experiments could be done to prove the conclusion?
7) Also, the study did not explore the regeneration of cortical cells via ZG cells, which differentiate into ZF cells according to existing literature.
8) Many plots in Figure 6 are not discussed.
9) While the idea of using Somatic CNV in human samples is good, it requires clearer explanation based on the plots generated.
10) The data and code are not available

Reviewer #2:
Remarks to the Author:
In this manuscript, Kastriti and his colleagues provided a comprehensive analysis of the adrenal glands in humans and mice. The authors employed spatial transcriptomics to 4 adult human adrenal glands samples. Then they integrated published single-cell data to study cellular turnover, tissue microarchitecture and signaling in human and mouse adrenal glands. Their research revealed novel cellular diversity within the adrenal glands, including previously unknown progenitor cells and sex-specific differences in cholesterol biosynthesis. Meanwhile, this research identified potential sources of adrenocortical renewal and compared the mechanisms between humans and mice. One concern is the lack of experimental validation for the conclusions drawn in this research. The article is descriptive, and many of the conclusions drawn have been reported in previous literature.

Several comments are listed noted below:
Although the novelty of the current manuscript lies in spatial transcriptomics, the authors only provide 4 visium data from four human donors. Since human samples are hard to acquire, I suggest the authors to supplement more visium analyses from mouse samples as well. These experiments will strengthen some of the conclusions as well, such as sex-specific differences and human mouse differences. In addition, their spatial transcriptomic data needs further interpretation. Most of their findings from single-cell analyses are not integrated into visium data.

Fig1-2
This section analyzed spatial and transcriptional heterogeneity in normal human adrenal cortex, combining scRNA sequencing and spatial transcriptomics approaches. Higher transcriptional activity of cholesterol biosynthesis pathways is observed in male ZG and ZF compared to female, and is partly attributed to sexual dimorphism. However, whether individual differences can be excluded from this conclusion based on the illustration of two samples (Fig 2e) remains unclear.

Fig3-4
1、The authors performed spatial transcriptomics on the adrenal glands of four individuals. However, when comparing sexual dimorphism in the adrenal cortex, gene expression data were only provided for two individuals (e.g., Fig 2e, sFig 8, 10-11). Furthermore, when focusing on the renewal of the human adult adrenal cortex, gene expression data was only presented for one male individual (e.g., Fig 3b-e, g, Extended Fig 5). It is essential to present key gene expression data from other individuals to support the authors' findings.

2、In the section on the renewal of the human adult adrenal cortex, the authors observed a "gradient" of DCN expression in transition 1 (line 289) and "gradients" of ACTA2, RGS5, PLCXD3, and STEAP4 expression in transition 2 (line 295). It is unclear how they defined the "gradients" of the given genes from the gene expression plot.

3、The authors uncovered a novel central SF1+ adrenocortical progenitor population and suggested that these cells are distributed throughout the cortex. They should validate their findings using immunofluorescence or other methods.

4、In line 366, the authors re-clustered the fetal mesenchyme, but the characteristics of these subpopulations do not seem to be mentioned thereafter.

5、In the section comparing the mesenchyme-to-cortex transitions in adult and fetal adrenal gland data, the authors simply projected the previous transitional cell population onto the integrated umap (Fig 4c). Is their any population in the visium data supporting this finding? Can you provide more validation using immunostaining? Can you label these transitional cells with EdU and perform single cell analyses experiments? Is it possible to do lineage tracing analyses based on somatic mutations?

Minor points:
1、In Extend Data Fig6, the legend of the first dot plot was incorrect. The maximum fraction of cells expressing the given genes is only 9%, it seems that '0' was omitted.

2、In line 293, "Transition 3" should be modified to "Transition 2"

3、Some plots in the manuscript are of poor readability. For example, in Extended Data Fig 6, it is difficult for readers to determine from which umap the cell type cluster originates, despite some description in the figure legend. I suggest that the authors modify their figure legend to specify the source figure.

Fig5-6
Firstly, it appears that most of the feature plots did not show bars for gene expression. In Fig. 5c, cluster 6/7/8/10 highly expressed Cyp11b2, which is a typical marker of zona glomerulata cells of the adrenal cortex. However, in Extended Data Fig. 9, dotplot showed that only cluster 6 seemed to have high expression of this gene, and the expression ratio was not high.

In line 470-474, Gli1+/Ptch1+/ Wt1-cells mentioned were not shown in the figure, and I wonder if this group of cells can be more intuitively represented. The conclusion "Gli1+/Ptch1+/Wt1-cells contribute directly to dividing to Nr5a1+/Shh+/Wnt4+ adrenocortical progenitors " mainly came from the featureplots, which seemed to be not convincing.

In Supplementary Fig. 29, the arrow of RNA velocity showed that cluster9 had a tendency to transform into other ZG cells. Could the authors explain this part in detail, such as showing the gene expression changes during transition?

In part of comparisons between adult human and mouse adrenal single cell, the authors primarily presented dimensionality reduction plots and dotplots of marker genes for each lineage in both species. However, a systematic cross-species comparison for each lineage was not explicitly detailed. Similarly, Fig. 5d-f only showed the expression differences of a few genes between the two species without in-depth analysis.

In the last section, the authors demonstrated lineage tracing using somatic mutation, but the process of the analysis was not specified in the methods, and the conclusions drawn from the results were not solid enough. Furthermore, the methods section contained an extensive explanation of dimensionality reduction and clustering processes. It raises the question of whether the presentation of the methods could be restructured for clarity and conciseness.

Reviewer #3:
Remarks to the Author:
Several single cell transcriptomic studies of the adrenal gland have been published in recent years, but the present manuscript is by far the most detailed and includes scRNA-seq and spatial (visium) RNA-seq data from adult samples from both sexes in human and mouse. In addition, meta-analysis on already published developmental data was performed. The major claims can be summarized as follows: 1) the human zG is comprised of a complex mixture of cell types with a 'novel' cell type that is proposed to express 11 deoxycortisone. Mouse zG is suggested to be less complex. 2) Sexual dimorphism of adrenal cortical cells appears to involve differential cholesterol biosynthesis between sexes. 3) Bioinformatic analysis suggests the existence of several progenitor cells that build and maintain the adrenal cortex in humans. 4) Integration of fetal and adult human data appear to have distinct routes of progenitor recruitment. 5) mouse adult adrenals appear to show lower cellular complexity and use routes of recruitment that differ from those in humans.
Taken together this atlas provides important information about adrenal biology with findings that are potentially of high interest to the scientific community. However, as it stands, the conclusions are based entirely on bioinformatic analyses, which must be interpreted with caution as they depend on proper normalization, data integration, and interpretation and can lead to misleading hypotheses. It is therefore essential to carry out orthologous studies to corroborate their claims.
Some major points are summarized below:

Major points:
1. The paper is very dense and hard to read and would benefit from reorganizing and streamlining.
2. Human samples come from 11 different individuals with highly diverse genetic background, ages and stress levels. While it is good to 'pool' samples to identify common mechanisms it is equally important to identify differences, in particular when subpopulations are identified. What is the distribution of each cell type in the different individuals? (this information is somewhat available in suppl. Figure 2, but it would be good to show a bar plot with the percentage of each cluster). Does the heterogeneity in the zG persist when clustering individuals?
zG complexity

3. While some cellular diversity within the zG has been previously reported, the authors claim this zone to be highly heterogenous. The identification of a new subpopulation of ZG cells (Cluster 5 - HSD3B2 positive and CYP11B2 negative ZG cells) is potentially of high interest, but the authors base there analysis entirely on the localization of HSD3B2 expression using Visium. The resolution of the 10x Genomics Visium is too low to identify these cells. Immuno- and/or high resolution ISH (e.g. RNA-Scope) analyses have to be carried out to identify these cells in vivo to confirm their localization and the hypothesis that they do not express CYP11B2. Is there a particular position within the rosette structures of the zG that harbors these cells? HSD3B2 is a key enzyme in steroid production and is therefore expected to be found in all steroidogenic cells (see for example the human protein atlas) How do the authors reconcile their findings with these data? Moreover, the authors predict cluster 5 cells to produce cortisol, which is simply inferred from the transcriptomic data. A functional confirmation is missing.
4. Lines 166-182: The authors performed reclustering focusing on a subpopulation of cells and identified 6 subclusters that they assigned to the zG. The basis for this assignment (zG versus zF) is unclear to me. Indeed, when looking at extended data 2 (panel e), it seems that zG-CCN3 (subcluster 5) and zG-DLK1 (subcluster 6 of the focused clustering) are transcriptionally more related to the zF than the zG. The visium data suggest CCN3 and DKL1 to be enriched in more cortical regions, but perhaps these are cells that are in the process of transitioning from zG to zF? The authors need to take care to differentiate between slightly different cellular states (that could occur in a transitional process) versus a real cellular subtype.
5. Cluster 2:ZG-VCAN: The use of VCAN as a marker for a zG specific subtype seems not particularly meaningful. According to the visium image (extended data Fig2e) VCAN expression appears to be scattered throughout the adrenal gland. How highly expressed is this gene? Additional cluster specific markers should be shown and the specificity confirmed using another method.

Sexual dimorphism:
6. The authors found cholesterol synthesis associated genes to be enriched in male samples. Is there any evidence that AR signaling suppresses cholesterol synthesis? Without any additional data, the discussion on sexual dimorphism of cholesterol (line 706 to 744) is very speculative and needs to be shortened.
7. A recent study by the group of Morohashi has found that androgens suppress global gene expression (doi: 10.1016/j.celrep.2024.113715) in mouse adrenocortical cells. Do the authors find increased transcripts in female mouse samples? Is this conserved in humans? If yes, could this explain some of the differential expression between males in females in different subclusters?
8. Cluster 16 (original clustering) is identified by high levels of H19 expression, but I don’t see any mention of this cluster in the text and according to the visium profile the expression seems to be not zone specific. Is this a real cell type?
9. Suppl. Fig.8b: the sexually dimorphic expression appears to differ quite dramatically when comparing scRNA-Seq and visium data. (e.g. enrichment of Cyp11B2 in males for scRNAseq data, but in females for visium data). This needs to be addressed.

Adrenal progenitors:
10. Transitions: The authors claim to have identified 3 different transition states. Additional data are necessary to proof this claim.
11. NR5a1+ cells that undergo cycling should not be considered as true progenitors, if they express other steroidogenic markers and are therefore likely to be actively involved in steroidogenesis. Instead these are differentiated cells that maintain the capacity to undergo cell cycling. Cycling of NR5A1+ cells has been reported in several studies and the statement in Line 279: “previously unidentified adrenocortical progenitors that lack mesenchymal features” is therefore incorrect.
12. The authors present evidence for 3 transitions which as far as I can see are primarily based on the arrangement of cells on the UMAP (connection between clusters). While this is indicative it is not formal proof that the cells transit. Moreover, velocity analysis appears to be highly variable between different samples thus questioning the usability of this analysis. If no additional data can be provided, the claims of transition between the different clusters need to be greatly toned down.
13. Reclustering focusing on progenitor cells: I do not understand why the authors excluded clusters 2 and 13 for their subclustering?
14. Line 278/279: The authors describe “previously unidentified adrenocortical progenitors’ (SF1+ cells lacking mesenchymal features). Proliferation of SF1 (NR5A1) positive adrenocortical cells is well established in the field. These cells should however not be considered as progenitors, but rather as differentiated cells that maintain their capacity to undergo cell cycling.
The proposed transition of ACTA2+/RGS5+/MYH11+ VSMCs to the zF and ZR is somewhat provocative as it contradicts studies in mice. I do not dispute the fact that cell renewal might be different in the two species, but the authors need to provide better evidence that there is a real transition into steroidogenic cells.
15. Concerning CCDC80: Line 284: According to the literature (https://doi.org/10.1210/me.2014-1060), Ccdc80 is a WNT dependent gene that is strongly expressed in zG cells, at least in the mouse. Is the expression in humans different? Line 287: Why does the expression of the “CCDC80+ gene, nearly exclusive to RSPO3+ fibroblasts, confirm that specifically capsular fibroblasts are involved in this transition” ?
16. Line 700: “Finally, we identified unique transitions from-mesenchyme-to-cortex in adult adrenal as compared to fetal” Some of these differences may stem from artefacts due to data integration

Integration of developmental data:
17. Figure 4b: This graph is very difficult to understand, without additional explanation. What characteristics were used to define fetal progenitors? The authors suggest that the transitions from progenitors to steroidogenic cells are profoundly different between developmental and adult samples. However, the overlap of data sets after data integration (shown in suppl. Fig 24) does not seem to be particularly strong. What is the evidence that the data normalization of the different data sets worked well? This is important for drawing firm conclusions about differences between fetal and adult adrenal biology.

18. Extended data Fig7a and Suppl. Fig.24 g: I am not sure I interpret this map correctly, but it seems to me that mesenchymal and steroidogenic cells are not connected. If this is the case, how can the authors draw conclusion about progenitor cell -> steroidogenic cell transitions during development?

Mouse adult data:
19. The proposed transition of cells from the mesenchyme to both zG and zF is interesting as it would suggest that cells are able to be directly recruited to the zF (without prior zG differentiation). This would be supporting data by Freedman et al., 2013 (10.1016/j.devcel.2013.07.016). This should be discussed.
20. Grabek et al. (2019), suggested recruitment to be higher in females than in males. Are female cells 'enriched' in transitional clusters? According to extended Fig10 transitional clusters 4 & 5 appear to be enriched in males – which is the opposite of what I would expect).
21. Supplementary data Figure 28: The expression profile does not well reflect well the known zonated expression of genes. For example Axin2 (b-catenin signaling responsive gene) was shown to be highly expressed in zG but only at low levels in the zF. Is this a problem of representing intensity on the UMAPs?
22. The discussion is rather long, at times highly speculative and should be shortened.

Minor points:
Line 67: the coelomic epithelium is lateral plate and not intermediate mesoderm derived
A previous manuscript has suggested that in mice nestin+ cells can contribute to the steroidogenic lineage. Is there any evidence from the single cell data for this transition (in both mice and human)?
Extended figure 6: LGR4 often substitutes for LGR5: please provide data for the expression of this gene
Fig 3f: Correct "Strerоidogenesis" -> 'Steroidogenesis"

Version 1:

Decision Letter:

2nd Oct 2025

Dear Professor Adameyko,

Your Article, "Human and mouse adrenal glands are characterized by species-specific and sexually heterogeneity and tissue turnover" has now been seen by 3 referees. You will see from their comments below that while they find your work of interest, some important points are raised. Particularly, Reviewer #3 still points to a lack of sufficient validation of some of the major claims of your study. We are interested in the possibility of publishing your study in Nature Genetics, but would like to consider your response to these concerns in the form of a revised manuscript before we make a final decision on publication.

To guide the scope of the revisions, the editors discuss the referee reports in detail within the team, including with the chief editor, with a view to identifying key priorities that should be addressed in revision and sometimes overruling referee requests that are deemed beyond the scope of the current study. In this case, we please ask you to address the remaining concerns in full. We hope that you will find the prioritized set of referee points to be useful when revising your study. Please do not hesitate to get in touch if you would like to discuss these issues further.

We therefore invite you to revise your manuscript taking into account all reviewer and editor comments. Please highlight all changes in the manuscript text file. At this stage we will need you to upload a copy of the manuscript in MS Word .docx or similar editable format.

We are committed to providing a fair and constructive peer-review process. Do not hesitate to contact us if there are specific requests from the reviewers that you believe are technically impossible or unlikely to yield a meaningful outcome.

When revising your manuscript:

*1) Include a "Response to referees" document detailing, point-by-point, how you addressed each referee comment. If no action was taken to address a point, you must provide a compelling argument. This response will be sent back to the referees along with the revised manuscript.

*2) If you have not done so already please begin to revise your manuscript so that it conforms to our Article format instructions, available
here.
Refer also to any guidelines provided in this letter.

*3) Include a revised version of any required Reporting Summary: https://www.nature.com/documents/nr-reporting-summary.pdf
It will be available to referees (and, potentially, statisticians) to aid in their evaluation if the manuscript goes back for peer review.
A revised checklist is essential for re-review of the paper.

Please be aware of our guidelines on digital image standards.

EXTENDED DATA FIGURES

When re-submitting your manuscript, please ensure that any supplementary figures and tables that are crucial to the manuscript's conclusions are converted into Extended Data figures and tables to increase visibility of these data. Extended Data figures and tables are online-only (present in the online PDF and full-text HTML versions of the paper), peer-reviewed display items that provide essential background to the article but are not included in the main article due to space constraints. A maximum of ten Extended Data display items (figures and tables) is permitted.

Please use the link below to submit your revised manuscript and related files:

Link Redacted

Note: This URL links to your confidential home page and associated information about manuscripts you may have submitted, or that you are reviewing for us. If you wish to forward this email to co-authors, please delete the link to your homepage.

We hope to receive your revised manuscript within four to eight weeks. If you cannot send it within this time, please let us know.

Please do not hesitate to contact me if you have any questions or would like to discuss these revisions further.

Nature Genetics is committed to improving transparency in authorship. As part of our efforts in this direction, we are now requesting that all authors identified as 'corresponding author' on published papers create and link their Open Researcher and Contributor Identifier (ORCID) with their account on the Manuscript Tracking System (MTS), prior to acceptance. ORCID helps the scientific community achieve unambiguous attribution of all scholarly contributions. You can create and link your ORCID from the home page of the MTS by clicking on 'Modify my Springer Nature account'. For more information please visit please visit www.springernature.com/orcid.

We look forward to seeing the revised manuscript and thank you for the opportunity to review your work.

Sincerely,
Chiara

Chiara Anania, PhD
Associate Editor
Nature Genetics
https://orcid.org/0000-0003-1549-4157

Reviewers' Comments:

Reviewer #1 (Remarks to the Author):

The revision addresses most of my major concerns with new data and a clearer narrative. The Results are now organized around four explicit questions, which greatly improves coherence, and the fetal analysis is elevated into a dedicated, data rich section (Fig. 5) instead of a brief mention in the Discussion. Orthogonal validation has been added throughout: high resolution spatial datasets (Stereo seq for human, Visium HD for mouse) anchor the single cell subclusters in tissue and help adjudicate transitional states (Figs. 2-4, 7), while RNAscope/IHC support key ZG markers (e.g., VCAN, CCN3, DLK1) and the ZG HSD3B2^high state (Fig. 3; Extended Data referenced). The renewal section is rewritten with three concrete sources (capsule→ZG via DCN^+, VSMC→ZF/ZR, and dispersed NR5A1^+/EZH2^+ progenitors) and linked to spatial evidence and a schematic (Fig. 4i-k). The interspecies comparison is now explicit (co integrated UMAP plus spatial alignment) and carefully distinguishes conserved aldosterone producing cells from human or mouse specific programs (Figs. 6-7). Sex effects are handled more systematically: no significant male-female difference in human progenitor counts/cycling, contrasted with stronger proliferative signatures in female mouse progenitors, corroborated spatially (Fig. 8b-c). Finally, the color/cluster correspondence in focused embeddings is clarified (Fig. 4 caption), the transitional labeling for prior "clusters 9-10" is justified with spatial and clonal context, and data/code access is now provided.
That said, several items remain only partially resolved. First, "heterogeneity" claims are still largely qualitative; consider adding a formal cross species heterogeneity metric (e.g., diversity/entropy or differential gene program dispersion) to substantiate statements beyond descriptive clustering (Figs. 2-3, 6-7). Second, the Methods document AUC ranked markers (FindAllMarkers, "roc") but do not state the explicit AUC threshold used to accept subclusters; if an AUC > 0.8 rule guided interpretation, please codify it and, ideally, add a brief table listing each retained subcluster and its qualifying marker(s) (Methods pp. 27-31). Third, lineage inferences in human should be phrased more cautiously: RNA velocity is explicitly "variable across samples," and the somatic CNV evidence derives from a single donor (Fig. 4g; pp. 8-9); I recommend "supports" rather than "validates," and per sample velocity maps in Supplement. Fourth, the ZG HSD3B2^high "direct cortisol" interpretation is compelling transcriptionally/spatially but remains inferred (p. 7; Fig. 3); please soften to

“transcriptionally consistent with,” pending functional assays. Finally, the “cholesterol balance” is a gene set-based residual model (Fig. 3g-h); renaming it “inferred cholesterol balance activity” would avoid implying flux measurements and adding per donor age (and rare WT1^+ cell) quantification would pre empt concerns about confounding and detection bias (Fig. 4e-f; Fig. 8b).

Reviewer #1 (Remarks on code availability):

README need to be improved with better guidance on how to reproduce the result.

Reviewer #2 (Remarks to the Author):

I do not have further comments.

Reviewer #3 (Remarks to the Author):

The sheer amount of data in this manuscript is impressive and the authors have included a significant amount of additional data including high resolution spatial analysis. Saying this, I still have a number of problems with the manuscript, as I feel that the authors often overinterpret bioinformatic data. I realise that they included a statement (caution) at the end of their study to highlight the fact that many of their interpretations are primarily based on bioinformatic analysis. However, publishing models that are not fully corroborated may lead to the establishment of wrong dogmas, especially when published in a prestigious journal such as Nature Genetics.

Given the large amount of data (8 figures, 10 extended figures, 59 supplementary figures), verifying every claim in this manuscript is almost impossible for a reviewer. Below you will find a list of some of my concerns.

Cellular complexity:
One of the key claims of the manuscript is a very high complexity of the adrenal cortex with - for example - the presence of at least 6 different zG clusters as defined by single cell seq analysis. Depending on settings, single cell analysis can yield a very high number of clusters that represent relatively subtle alterations between cells of the same type rather than truly independent cell types.

zG-VCAN population:
In the revised manuscript the authors aimed to corroborate there bioinformatic findings using RNA-Scope data. While the expression of VCAN (Supplementary fig10) is indeed nicely restricted to the zG, I am not sure that they form a truly independent cluster. When looking at sup Fig. 10e it seems that the vast majority (if not all) of the zG cells are VCAN positive (the occasional DAPI+ VCAN- cell could be endothelial or immune).

ZG-HSD3B2 (CYP11B2 negative) population:
I am puzzled by supplemental Figure.11: The signal for the CYP11B2 probe seems to be present in both zG and ZF (strong staining in the zF – which should be negative for Cyp11B2)– are you sure the probe does not also detect CYP11B1? If this was the case, the argument of CYP11B2 negative cells in the zG producing glucocorticoids is not valid, as these cells would be also negative for CYP11B1.
When looking at the high resolution spatial data (Fig. 3D) the ZG-HSD3B2 population seems to be located more in the zF than the zG, at least for the F20 years and M13 years samples.

ZG-DLK1 population (lines 187-197):
The bioinformatic analysis further suggests the existence of a DLK1+/CYP11B1+/CYP11B2- transitional population, which is grouped together with the zG (labelled as zG-). However, the DLK1 antibody staining as well as the slide seq data places these cells within the zF (extended data Fig3d; Suppl. Fig14).
The statement: “This pattern of presence of CCN3 and DLK1 within the CYP17A1low/CYP11B1+ population (potentially cells slowly differentiating towards ZF) reflects transcriptional states of an intermediate steroidogenic population towards maturation” appears too strong without a direct lineage confirmation.
More generally, why attributing the name “zG” to a cell cluster expressing CYP11B1 (and not CYP11B2)? I am not surprised then that DLK1+ cells are found within the zF in immunostaining and in slide-seq analysis.

Taken together, the claim that the study revealed “ previously unrecognized cell populations, especially in zona glomerulosa” (abstract) seems not 100% validated.

Lineages (lines 282 onwards):
Transition 1:
This transition is in agreement with capsular recruitment to the zG and the position of the cells (spatial mapping). The gap between cluster 1 and 3 and the sudden switch of expression profiles (gene program transitions in suppl. Fig28j) is surprising and raises the question to what extent this transition occurs in adults.

Transition 2:
The authors suggest the existence of VSMC+ progenitor cells throughout the adrenal cortex that can transition towards the steroidogenic fate. This is potentially very interesting, but formal proof for this transition is in my opinion still missing.

Also I am not sure I completely follow the arguments based on mCA transmission. In line 318, the authors link VSCMs with the zG (suppl. Fig31.b&c), but in Fig.4K this link is not represented? On the other hand, the authors claim a strong link of VSMCs to the zR, but while the majority of VSMCs appear to carry the rearrangement (11/18), only 7 out of 473 ZR cells are aberrant. Wouldn't you expect the ZR to have a much higher rate of rearranged cells?

Transition 3:
The authors further propose the existence of an NR5A1+EZH2+ progenitor cell population that can give rise to all three steroidogenic zones. As outlined in my previous review I am not convinced that these cells represent true progenitors. Indeed TOP2A, UBE2T, SMNT and to a lesser extent also EZH2 are all strongly linked to cell cycle progression. Although the authors have now 'nuanced 'the text, the cluster is still named as Prog (for progenitors) and are displayed (Figure 4K) as an independent cell population that has one-way differentiation capacity towards zG, zF, zR. The confusing Velocity analysis in different samples (suppl Fig.32) does not help in this context. (incidentally, I would have expected EZH2 antibody staining to be nuclear (Extended Fig. 6)

The model in Figure 4K tries to conceptualize the complex cellular dependencies and lineages, but without additional data it may mislead the scientific community. I realise that obtaining functional data from human material is difficult, but I believe this would be required to confirm these concepts. Since the attempted in vitro work did not yield conclusive results, I think the authors need to be much more careful in their statements.

Version 2:

Decision Letter:

Our ref: NG-A64638R1

10th Apr 2026

Dear Dr. Adameyko,

Thank you for submitting your revised manuscript "Human and mouse adrenal glands are characterized by species-specific steroidogenic states and tissue turnover" (NG-A64638R1). It has now been seen by the original referees and their comments are below. The reviewers find that the paper has improved in revision, and therefore we'll be happy in principle to publish it in Nature Genetics, pending minor revisions to satisfy the referees' final requests and to comply with our editorial and formatting guidelines.

If the current version of your manuscript is in a PDF format, please email us a copy of the file in an editable format (Microsoft Word or LaTex)-- we can not proceed with PDFs at this stage.

We are now performing detailed checks on your paper and will send you a checklist detailing our editorial and formatting requirements soon. Please do not upload the final materials and make any revisions until you receive this additional information from us.

Thank you again for your interest in Nature Genetics Please do not hesitate to contact me if you have any questions.

Sincerely,

Cong Zhao, PhD
Research Cross-Journal Editorial Team
Nature Genetics
https://orcid.org/0009-0008-5833-6662

Reviewer #1 (Remarks to the Author):

My concerns have been addressed and I have no further questions

Reviewer #1 (Remarks on figshare data availability):

The dataset seems valid and sufficient

Reviewer #3 (Remarks to the Author):

I appreciate the authors' further efforts to improve the manuscript and acknowledge that they have further toned down some of their claims. While the impressive amount of data and analysis will be a treasure trove for future research, personally I remain unconvinced that some of the proposed models -particularly those concerning cortical renewal - are sufficiently supported.

Editorial comments:

Word counts: Ensure abstract, main text, methods and figure legends comply with NG's standards (Abstract~150 words; Main text~ 4000-4400 words; Methods~3000 words; Main Figure legends & Extended Data Figure legends ~250 words). Online Methods should be included in the main manuscript file. Excess text can be included as part of the Supplementary Note.
Keywords section is not needed.
References: split into those for the main text and methods-only and number methods-only references starting from 68.
Move the contents of "Inclusion & ethics statement" to author contribution.
Code availability: deposit the code in a DOI-minting repository (in addition to GitHub).
Data access/data availability: All sequencing data needs to be released immediately, not just made available to the reviewers. Please do not use bullet points.
Main figures should be uploaded as separate files and not included in the main article file. Main figure legends should be included in the main manuscript file (word format) for word counts.
Move Table 1 in Methods to Supplementary Note; The table should fit on a single page.
Rename "Supplementary Information File 1" as "Supplementary Note": Supplementary Figures, Table 1 and other notes (e.g. excess texts) should be combined into this PDF.
Supplementary table isn't prepared properly. It should be uploaded as one file with multiple tabs (rename the tabs as Supplementary Table 1, Supplementary Table 2, and so on) and the titles and legends for Supplementary table should be included directly on the individual excel sheet. Please revise the manuscript text callouts accordingly to match after renaming the Supplementary Tables.
For deceased organ donors, it is essential that those individuals provided consent to have samples removed for research purposes. Please provide the consent.

Open Access This Peer Review File is licensed under a Creative Commons Attribution 4.0 International License, which permits use, sharing, adaptation, distribution and reproduction in any medium or format, as long as you give appropriate credit to the original author(s) and the source, provide a link to the Creative Commons license, and indicate if changes were made.
In cases where reviewers are anonymous, credit should be given to 'Anonymous Referee' and the source.
The images or other third party material in this Peer Review File are included in the article's Creative Commons license, unless indicated otherwise in a credit line to the material. If material is not included in the article's Creative Commons license and your intended use is not permitted by statutory regulation or exceeds the permitted use, you will need to obtain permission directly from the copyright holder.
To view a copy of this license, visit https://creativecommons.org/licenses/by/4.0/

Response to reviewer comments

Reviewer #1: (expertise: computational; spatial transcriptomics)

This study employs single-cell and spatial RNA-seq to investigate the diversity and self-renewal mechanisms of human adrenocortical cells, utilizing both adult and fetal human adrenal samples. The author identified new progenitor cells and highlight difference in human and murine adrenal gland.

Comments on the manuscript:

- Upon reviewing the results and discussion, it's evident that the mechanisms about human adrenocortical cells are a primary focus for the authors. However, the narrative lacks coherence, particularly regarding the rationale behind the study design, which appears complex and extensive.

We thank the reviewer for being so thorough when reading our manuscript and providing helpful comments. During this long 1,5 years revision process, we not only carried out new experiments, but significantly reworked the whole text. To improve the structure and focus, we reorganized our Results sections systematically according to a specific question we were investigating. These are the subsection headings we introduced to improve the flow:

"Cell type heterogeneity of the human adrenal gland"

"Mechanisms of renewal of steroidogenic cells in the human adult adrenal cortex"

"Comparison of adult and fetal human adrenocortical heterogeneity and transitions"

"Comparative analysis of human and mouse adult adrenal cortex"

In addition to this, we inserted explanatory passages and rationale for blocks of experiments and analysis. Here are few examples from the Results section:

"To better understand the spatial arrangement of these transcriptionally distinct ZG populations and to contextualize their potential functional roles we turned to high-resolution spatial transcriptomics. Using Stereo-seq[34] to analyze cortical regions of three donors (two men, one woman, ages 13-41), we visualized the distribution of the subpopulations across cortical zones on tissue sections (Supplementary Figs. 12-13, Fig. 3c-d)."

"Given the central role of cholesterol in steroidogenesis and observed sex-related differences in the respective genes, we systematically examined the balance between pathways for acquisition of cholesterol including de novo biosynthesis and uptake from blood, and its metabolic usage through steroidogenesis and esterification across different adrenal zones and sexes, extending this analysis to published bulk RNA sequencing data on a GTEx cohort of 274 individuals[35]."

"A fundamental unanswered question is whether cells across organs are replaced through similar mechanisms during adulthood versus development. This is key for organoid models, which thus far have been established using embryonic principles that may not recapitulate self-renewal mechanisms of adult organs. To examine whether adult adrenocortical cell renewal mechanisms are similar to the transitions in the developing adrenal cortex, we

reanalyzed published data on developing human adrenocortical primordium from 7 to 21 weeks post fertilization (or Carnegie stages 16/17 onwards) and 6 days postnatally (Supplementary Figs. 34-35)."

We also changed the structure of the Discussion going through logical blocks in a systematic sequence. We hope the review will appreciate the improvements and enjoy reading the revised version of the manuscript.

The inclusion of fetal data is briefly mentioned in the discussion section, rather than being clearly articulated earlier.

We want to thank the reviewer for pointing this out. We fixed this issue by improving the relevant figure (new Fig. 5), which in combination with earlier figures (Supplementary Fig. 41, Extended Data Fig. 7) is an extensive analysis of the fetal cortex in comparison to the adult (Supplementary Figs. 37-38). We also performed additional analysis integrating only adult and fetal 10x cells (excluding fetal 10x nuclei and Microwell-seq cells), which significantly decreased the amount of data and timepoints considered, but resulted in better alignment and allowed deeper exploration of cortical subpopulations (Extended Data Fig. 8). Finally, we wrote the fully dedicated section *"Comparison of adult and fetal human adrenocortical heterogeneity and transitions"* in Results, where we outline a thorough comparison of mesenchyme and major steroidogenic populations, recapped as follows in the Results:

"In conclusion, despite general functional homology of major progenitor and steroidogenic populations between fetal and adult tissues, there were notable differences. Cortical NR5A1+/EZH2+ progenitor cells are present in both fetal and adult stages, but decrease in abundance with fetal-to-postnatal transition. Next, adult renewal appears to involve adrenal capsule-derived progenitors, contrasting with the fetal cortex at 6-7 weeks post-conception and onwards -a time during which a direct mesenchyme-to-steroidogenic transition is not evident, and a bridging population between the two is not observed in data (Extended data Fig. 7a). This is in agreement with studies recapitulating human fetal cortex development and the subsequent expansion of the fetal cortex through proliferation, rather than mesenchymal-to-steroidogenic transitions[5,39,40]. In regards to the composition of the steroidogenic fetal and adult adrenal cortex our observations revealed the overrepresentation of CCN3+ cells in adult cortex and DLK1+ cells in fetal. Also, we detected the exclusive presence of DCN+ population within the adult ZG, which was absent in the fetal cortex, hinting at disparities in the maturation dynamics of the newly generated steroidogenic cells found in the outermost part of the adrenal."

We also recap our conclusions from this comparison in the Discussion:

"Our comparison between the human fetal and adult single cell data revealed only partial similarity, along with significant differences in key transitory DCN+, CCN3+ and DLK1+ adrenocortical populations and proliferating cortical NR5A1+/EZH2+ progenitors (largely underrepresented in adult adrenals)."

"We show a similarity between FZ of the developing cortex with ZR of the adult based on androgen-producing capacity, and DZ of the fetal cortex with adult ZG based on CCN3 and HSD3B2 expression. Importantly, the adult-specific VSMCs, as well as DCN+ state, mediate transitions towards ZG and ZF, whereas the fetal cortex at the analyzed stages of

development instead grows through local cortical proliferation of NR5A1+ cells as previously suggested[40]*."*

Also, the exploration on self-renewal reveals gender differences but lacks organization.

We thank the reviewer for this request. To address it, we decided to revisit proliferative signatures using more data we obtained during this revision. After systematic analysis of dissociate single cell transcriptomes and new spatial data obtained from adult human adrenals, we arrived to the conclusion that the numbers of proliferating progenitors do not differ significantly in men and women adrenal cortexes. Also, we found no difference in the proliferative gene expression signature in adrenals from different human sexes.

However, this situation is different in mice: despite the fact that numbers of proliferating cells do not differ in male and female glands according to the sum of all data (similar to humans), the proliferation gene expression signature is much stronger in females, suggesting that female cells are cycling faster.

To better deal with this, we also reorganized the text, with dedicated paragraphs to the interpretation of our results, and assembled new figures, following the addition of high resolution spatial transcriptomics in human (tissue from one female, two males analyzed through Stereo-seq) and adult mouse adrenals (from 3 male and 3 female mice, analyzed by Visium HD).

The data description and interpretation can be found in the following text in the Results section and Fig. 4, 8, Supplementary Fig. 49, 58):

"Interestingly, analysis of numbers of proliferating NR5A1+ progenitor cells did not reveal a significant difference between males and females in both mouse and human, in single cell or spatial data (Fig. 8b). Despite this fact, further exploration of mouse sexual dimorphism led to the observation of enrichment of cell cycle and DNA replication pathways in female-derived proliferating NR5A1+ progenitor cells (cluster 4:PROG) (Supplementary Fig. 49g-i, Fig. 8c). This finding was supported by Visium HD spatial transcriptomics data, whereby analysis of mitotic signature in mouse male versus female cortex as a function of a) cellular subtype and b) zone of enrichment, confirmed more extensive proliferation within the progenitors in female mice (Fig. 8c, Supplementary Fig. 58). Thus, the previously described increased rate of adrenal cortex turnover in female mice[14] *likely relies not on a larger number of progenitors but on an increased rate of their proliferation. In a similar analysis comparing proliferation activity between human male and female progenitors, statistical significance was not achieved (Fig. 8b,c)."*

- In the abstract the authors say "**Intriguingly, our comparisons between human and murine adrenal glands demonstrated reduced zona glomerulosa cell type heterogeneity in mice, which questions the applicability of mouse models in studies of human adrenal disorders.**" But it is not well supported in the main text. Most of the conclusions were draw from scRNAseq data but lack supporting evidence from other experiments such as functional assay or imaging. For example, "cell heterogeneity" has been extensively used throughout the manuscript, **but mostly just from increased number of clusters, which can be a result of changing clustering parameters in data analysis.**

In this revision, we introduced several layers helping to resolve this and other similar issues. Firstly, we rewrote the abstract, and it is now more specific and detailed. Secondly, we generate the orthogonal experimental data such as high resolution (in fact, single cell resolution) spatial transcriptomics for human and mouse adrenals, as well as RNAscope and immunohistochemistry results in human tissue. Any cluster that we get in single cell data analysis, we can nest in space and check its validity and biological meaning via exploring transcriptional states in spatial context.

Third, as for the valid concern related to the heterogeneity being affected by different clustering parameters, to avoid such technical over-clustering, we took measures both at the scRNA-seq computational analysis and validation stages. We updated all tissue-level integrations, focused integrations and re-clustering procedures, which are described in the Methods section with high detail. For instance, in the computational analysis we note that our in-depth study of ZG cells heterogeneity did not simply rely on increased clustering resolution. Instead, the target clusters were selected from the batch-corrected count matrix, then RunPCA, RunUMAP, FindNeighbors, FindClusters steps were performed de novo. Focused reclustering of target areas is a common procedure in scRNA-seq analysis and has been extensively used in various scRNA-seq studies (Cheng et al., 2022; Kameneva et al., 2021) to increase local resolution. After tissue-level (whole adrenal including immune, medulla and others) integration all other integrations have been performed with Seurat FindClusters parameter resolution = 0.5, which is even lower, than default one (0.8) and in range recommended by Seurat vignette (0.4-1.2). Importantly, we included in our interpretation only clusters with at least one well-defined marker gene with (AUC > 0.8), while technical overclustering leads to origination of not well-defined subpopulations without specific markers. We decided to experiment with this by inducing overclustering on purpose – for this we again reclustered central part and zona glomerulosa of human cortex. Large cluster 7:ZF split into two equal sized parts without significant differences in expression or specific marker genes (see Supplementary Information – Supplementary Methods section). Thus, we can detect overclustering, because we can define it as the absence of biological interpretation for the cluster based on specifically expressed features. We now clarified that in the Methods section.

We further addressed this question during our revision in the following way: we re-analysed human and mouse adrenal cortical populations and their transcriptomic signature with higher level of scrutiny (revised Fig. 3 and 6). in every step of the analysis (isolated human or mouse steroidogenic cells), we also show dotplots of key marker genes, to allow for unbiased exploration of the data (Extended Data Fig. 5, 9). To address our claim on ZG heterogeneity (relevant to ZF and ZR as well), we subselected and reclustered them in human single cell data sets (Fig. 3) and uncovered clear and discriminating transcriptional differences between the observed clusters (Fig. 3a,b), which support the realistic nature of our clustering resolution and biological robustness of clusters.

Now, in this revision, to validate the numerous subtypes we identified, we employed the in situ validation by means of Stereo-seq and spatial sequencing with single cell resolution Visium HD in both species and performed a comparative analysis, which allowed to visualize fundamental differences between the two species, such as human-specific ZG (*DCN*, *VCAN*, *CCN3* and *DLK1*) or ZF markers (*SULT2A1*, *CYP17A1*) and mouse-specific *Ly6d* and *Ptn* (overview in revised Fig. 7). In the revised manuscript we avoid vague terms such as "increased heterogeneity" or "decreased heterogeneity", but perform meticulous interspecies comparison of marker genes of identified cell clusters in both spatial and scRNA-seq data (revised Fig. 7).-Our revised findings are laid out in the following section of Results:

"First, we explored the basic composition of murine adrenocortical zones prior to inter-species comparisons. Murine ZG (clusters 6-10) consisted of Dab2/Wnt4-expressing clusters characterized by mutually exclusive expression of Hsd3b6 (cluster 10), linked to glucocorticoid-producing identity, and Cyp11b2 expression, catalyzing mineralocorticoid/aldosterone synthesis (clusters 6, 7, 8, 9) (Extended Data Fig. 9a-c). Cyp11b2-expressing subclusters were discriminated by the expression of Ly6d, the calcium-sensor Vsnl1[45] (a well-established marker of ZG in human cortex) and Steap1, a gene with unexplored role in adrenocortical physiology (Extended Data Fig. 9c).

Murine ZF (clusters 11-14) consisted of Hsd3b1/Cyp11b1-expressing glucocorticoid-synthesizing clusters lacking well-defined specific markers, apart from gradients of genes such as Meg3, Idh1, Akr1cl and others (cluster 14)."

"Finally, to carry out interspecies comparative analysis we constructed a combined alignment and UMAP embedding of both mouse and human adult adrenal cortex (Fig. 6b-d, Supplementary Figs. 50-51). Such embedding should allow to cluster cells with functional and transcriptional homology between the two species in the same domains. We observed co-clustering of a) mesenchymal and b) steroidogenic (NR5A1+/EZH2+) cycling populations of the two species, indicating high transcriptional overlap within each subgroup. Furthermore, STAR+ steroidogenic cells co-clustered in the case of aldosterone-producing Cyp11b2/CYP11B2 cells of mouse and human ZG and occupied similar subcapsular positions, indicating conserved placement despite human CYP11B2+ cells found in more wide domains (Fig. 6c-f, Supplementary Fig. 52). Interestingly, analysis of the expression pattern of 3β-hydroxysteroid dehydrogenase, involved in the synthesis of all steroid hormones, revealed differential distribution of the human-specific HSD3B2 (type II isoform) at ZG and ZF, versus the inner ZG-specific expression of the murine isoform Hsd3b6 (type VI isoform) (Fig. 6e,f), previously reported by an immunohistochemical analysis on mouse adrenal[47]. However, all other adrenocortical subclusters occupied species-restricted domains in the UMAP embedding, revealing significant transcriptional divergence despite general-level similarity (Fig. 6c,d, Supplementary Fig. 52), with a similar general spatial distribution of clusters based on functional specialization (Fig. 7a,b) revealed by an in-house developed spatial alignment procedure (Extended Data Fig. 10, see detailed Methods)."

- Overall, the data analysis human adrenal gland, mouse adrenal gland and human fetal adrenal gland seems discrete and does not form a complete story. The key message is unclear and does not provide enough novelty compared to similar recent studies (https://www.biorxiv.org/content/10.1101/2022.08.27.505530v1, https://insight.jci.org/articles/view/168177 and https://academic.oup.com/jes/article/7/12/bvad131/7329838).

To respond to this comment, we invested our time and efforts into additional experimental work to complement our atlas with single cell resolved spatial data for humans and mice to be able to make and validate additional discoveries. We hope that we significantly improved the level of novelty in the revised manuscript. Also, following this comment, we took care to present the original motivation and differentiate our study from earlier works in the revised manuscript. For this, we wrote a new section in the discussion:

"A handful of earlier studies already relied on single cell methods to address the development of fetal human adrenals, without addressing the homology of steroidogenic cell types or

comparing fetal progenitors to those in the adult cortex[30,38]. Our comparison between the human fetal and adult single cell data revealed only partial similarity, along with significant differences in key transitory DCN+, CCN3+ and DLK1+ adrenocortical populations and proliferating cortical NR5A1+/EZH2+ progenitors (largely underrepresented in adult adrenals).

Next, we further pinpointed self-renewal sources unique to adult adrenal glands (ACTA2+/RGS5+ VSMC mesenchymal population and DCN+ progenitors derived from the capsule). We show a similarity between FZ of the developing cortex with ZR of the adult based on androgen-producing capacity, and DZ of the fetal cortex with adult ZG based on CCN3 and HSD3B2 expression. Importantly, the adult-specific VSMCs, as well as DCN+ state, mediate transitions towards ZG and ZF, whereas the fetal cortex at the analyzed stages of development instead grows through local cortical proliferation of NR5A1+ cells as previously suggested[40].

When it comes to previous single cell studies focusing on adult human adrenal glands, those have been technically challenging and constrained when it comes to sample diversity, type of assay, or cell type resolution. …. A previous interspecies analysis between human and mouse by Lai et al. revealed generally conserved zones and tissue types[52] (cortex, medulla, immune cells) but lacked the fine resolution to compare subpopulations of adrenocortical cells, a point that we addressed extensively."
"Building from this foundation, we raised several questions about human-specific features including highly specialized steroidogenic populations, self-renewal and sexual dimorphism in glands from men and women, all of which would require the level of fine-grain data at both single cell and spatial levels. This was implemented by incorporation of spatial data with true single-cell resolution (Visium HD and StereoSeq) for both adult human and mouse adrenals, offering a detailed view of tissue organization."

Importantly, in our work (and in contrast to other studies), we are focusing on interspecies and intersexual comparisons in the adult adrenal cortex, and we also do show for the first time that the cellular transitions in the cortex of the fetal adrenal are not recapitulated in the adult cortex. This is important in the context of an existing organoid model that mimics the fetal adrenal cortex (Sasaki et al).

The study by Fu and colleagues was published based on the spatial analysis of 2 adrenal samples, concluding in support of the centripetal differentiation model in adult human cortex, with cells progressing from the outer capsule to the zona glomerulosa, zona fasciculata, and zona reticularis. However, the study did not employ single-cell resolving technology, which we did in our extensive cohort, both on dissociated adrenal tissue and through spatial technology (Stereo-seq and Visium HD) on sections for mice and human samples, as well as both sexes (revised Fig. 3, 6, 7, Supplementary Fig. 12-15, Supplementary Fig. 45-48). We appreciate the validation of centripetal model by Fu et al., as this is also consistent with our results, and we expand further by exploring the progenitors and transitions beyond the centripetal model and features which outline sexual dimorphism and interspecies differences.

During this revision, we unveiled previously unrecognised populations with the human adrenal cortex, especially in the ZG and ZF (revised Fig. 2, 3), while performing extensive comparisons with the fetal human cortex (revised Fig. 5) and adult mouse cortex (revised Fig. 6,7) and generating a

valuable resource of high resolution spatial atlases. We hope that the reviewer will agree that we diversified our work from previous studies.

To be more precise, through the addition of extensive spatial data during the revision, we expanded into the following findings beyond previously published results:

1. Discovery and validation (using high resolution spatial transcriptomics (Stereo-seq), in situ hybridization and immunostainings, revised Fig. 3, Extended Data Figs. 3-4, Supplementary Figs. 12-15) of specific steroidogenic populations within the ZG, including cortisol-producing *HSD3B2*high/*CYP21A2*high cells, *CCN3+* and *DLK1+* cells possibly reflecting immature/transient states, a *VCAN*+ population, all together composing a view on ZG beyond the canonical *CYP11B2*-expressing aldosterone population.

2. Modes of de novo generation of steroidogenic cells, including capsule-derived ZG and VSMC-derived contribution to ZG and ZR and their validation in three donors through Stereo-seq (Fig. 4, Supplementary Figs. 15), as well as an extensive transcriptomic profile of rare *EZH2+*/*SF1+* proliferating cells in the adult human cortex (both males and females).

3. Robust differences between fetal and adult mesenchyme, which includes the ZG-maintaining capsule, with the fetal-specific WT1 and IGFBP7 expression and extensive proliferating SF1+ progenitors, along with the adult-specific presence of capsule- and VSCM-derived new cell generation.

4. Sexual dimorphism in adult and fetal human cholesterol pathway activity, with major implications for future research, and confirmation of sexual dimorphism in the extent of ZG turnover in adult mouse cortex.

5. Comparative analysis unveiling human- and mouse-specific populations in the cortex, with striking examples being the DCN+ transitional population from capsule-to-ZG, VCAN, CCN3, DLK1 ZG populations, apart from known absence of expression of SULT2A1 and CYP17A1 in mice. This was complimented the notable finding of different isoforms of 3β-hydroxysteroid dehydrogenase in the two species (revised Fig 6e) which highlights an interesting direction for Evolutionary Biology.

Minor comments:

1) Cluster 15, identified as ZF-CPB1, exhibits a transition-like form on the UMAP, yet this observation remains unaddressed in the section exploring the Origins of Cortical Renewal.

To address the significance and the nature of this cell population during the revision we analysed the distribution of the corresponding transcripts using new Stereo-seq data at single cell resolution (please see revised Fig. 3, Supplementary Fig. 13), which revealed that they are found in lower positions within the ZF, neighbouring ZR. This agrees with centripetal model and is expected, and for this reason we do not make any special claim here:

"Using Stereo-seq[34] to analyze cortical regions of three donors (two men, one woman, ages 13-41), we visualized the distribution of the subpopulations across cortical zones on tissue sections (Supplementary Figs. 12-13, Fig. 3c-d). ... Within ZF, the NEAT1^{high} population was in the apical part of the zone, while CPB1^{high} cells were found in the basal part (Supplementary Figs. 13-14)."

Also, we are pointing out to the current limited knowledge regarding the function of *CBP1* in the cortex in the revised Results section:

"ZF cells were found connected to ZG smoothly, showing a gradual and directional gene expression change. They also exhibited less heterogeneity, being separated into a central CYP11B2-/CYP17A1^{high}/CYP11B1+ population, along with two populations of NEAT1^{high} and CPB1^{high} cells respectively (Fig. 3a,b). NEAT1 is a long non-coding mRNA associated with the nuclear envelope and involved in some cancer types[31,32], while the CPB1 gene encodes a plausibly secreted carboxypeptidase, both with unknown functions in the adrenal gland."

While, Clusters 9 and 10 in Figure 2 are designated as transition cells but lack clear explanations why they are labelled as transition cells.

These clusters were identified as transitional cells based on their position on the UMAP and forming intermediate clusters between mesenchyme and known adrenocortical clusters (in this case, ZR) and pseudotime analysis (Supplementary Figs. 28-30). In the revised manuscript we renamed them to "plausible transitional cells" or cells, which are "putatively directly involved in transition between mesenchymal and adrenocortical cells". Also, we would like to mention that transitory clusters show transcriptomes, which change gradually in a directional manner on the UMAP embedding or along the devised trajectory.

During the revision, to validate their presence in the tissue we performed spatial transcriptomics at single cell resolution, which implemented the presence of the intermediate states (see revised Fig 4i,j and Supplementary Figs. 13-15). To further support that this transition from VSMCs to cluster 9 and then cluster 10 is possible, we used the single cell data from the individual carrying fraction of cells with somatic copy number alterations to reconstruct the lineage from mesenchymal cells of clusters 9 and 10 towards steroidogenic cells (revised Fig 4g, Supplementary Fig. 31), which validated the hypothesized principle of generation of ZF and ZR cells from different clones of cells rooted in VSMCs.

The rationale and analysis in explained in the Results:

"Inferred transition 2 (Supplementary Fig. 29) from ACTA2+/RGS5+/MYH11+ VSMCs contributes through an RGS5+/ACTA2- transitory state to ZF and ZR (according to the UMAP representation of single cell transcriptomes) and possibly to ZG adrenocortical cells through intermediate states. Specifically, this is an intermediate step of differentiation from cluster 9, composed of RGS5+/ACTA2- cells, to cluster 10, composed of STEAP4+/TFPI2+ cells, depicted as transition 3 (Supplementary Fig. 30). Of note, vascular smooth muscle cells (VSMCs) are also present in the capsule, upper ZG and space between ZR and medulla. According to observed gene expression of ACTA2, RGS5, PLCXD3, STEAP4 in single cells and Visium in VSMCs and steroidogenic cells (and other genes shown in Supplementary Figs. 26-27), this transition mainly drives renewal of ZR. In a previous study by Gao et al.[36], we identified an individual carrying mosaic chromosomal alterations (mCAs), which can serve for cellular lineage reconstruction (Fig. 4g, Supplementary Fig. 31). The pattern of mCAs in this sample, composed of genetically normal ZR cells and clonally-linked aberrant ZG and ZF cells, supports a transition from VSMCs through TFPI2+/GNG11+ cells differentiating towards cells of the ZF and ZG, thus, validating the mesenchymal bridge. Cluster 3:TRANS-DCN also contained a high fraction of aberrant cells, indicating potential presence of aberrant fibroblasts and the transition from 1:Fibroblasts towards transient cluster 3:TRANS-DCN."

Additionally, the rationale behind the three pairs mentioned in the section 'Deciphering the Origins of Cortical Renewal in the Human Adult Adrenal Gland' requires elucidation.

We want to thank the reviewer for pointing out this problematic part of the text. In the revised manuscript we rewrote entirely this section, and we hope the clarity is now improved (now found as "Mechanisms of cell renewal of steroidogenic cells of the human adult adrenal cortex") and we are clearly spelling out the three sources of new adrenocortical cell generation. Specifically, we are referring to 1) capsular fibroblasts generating ZG cells, 2) VSMCs contributing to ZF and ZR and 3) proliferating EZH2+/SF1+ progenitors, found interspersed. We then move on to validating those bridges/populations with new Stereo-seq data generated from adrenal biopsies from three newly-acquired tissue donors and making a schematic overview for the readers' eased (revised Fig 4i-k).

2) The analysis of focused clusters in Figure 3 utilizes color to show relationships between clusters, but clearer plots are needed to illustrate correspondence. Consider applying this new representation to other sections as well.

We tried to address this important comment by changing pallets, and we were dissatisfied with these attempts. The result was misleading because of color overlaps, which were inevitable – we have too many clusters to color code in multiple instances.

To cope with the problem, we clarified in Results that the clusters that were subselected from UMAP on the left part of the revised Fig. 4 (clusters 7,8,9,10,12) were reclustered to increase their resolution, while keeping the original colors and using new ones only in the case of previous cluster 9 and 10, which now is reflected by clusters 1-4 in the smaller UMAP on the right. We also placed an additional dotted line to Figure 4a subselection to show that it is the same dotted line in the main embedding. This makes it now more comprehensive, and we thank the reviewer for helping us.

Finally, to fully address this issue, we instead inserted an explanation in the figure legend (revised Fig 4, legend for panel a):

"Clusters 7, 8, 9, 10 and 12 were re-analysed separately to increase local embedding and clustering resolution. Clusters 9, 12, 7, 8 from original embedding correspond to clusters 1, 5, 6, 7, respectively, on the new (focused) embedding and have the same colors on both UMAPs, while original cluster 10 due to increased resolution is divided into clusters 2, 3 and 4 with new colors."

3) **Are there differences between male and female** in deciphering **the origins of cortical renewal** in the human adult adrenal gland?

We want to thank the reviewer for bringing up this question, which we addressed thoroughly during the revision.

Following up this finding, during the revision we performed the analysis of our single cell data sets of human adrenal cortex and our newly generated spatial transcriptomics (Stereo-seq), but did not find statistically significant differences between men and women (revised Fig. 8b,c). We now state this clearly in the following section in the Results:

"Interestingly, analysis of numbers of proliferating NR5A1+ progenitor cells did not reveal a significant difference between males and females in both mouse and human, in single cell or spatial data (Fig. 8b). Despite this fact, further exploration of mouse sexual dimorphism led to the observation of enrichment of cell cycle and DNA replication pathways in female-derived proliferating NR5A1+ progenitor cells (cluster 4:PROG) (Supplementary Fig. 49g-i, Fig. 8c). This finding was supported by Visium HD spatial transcriptomics data, whereby analysis of mitotic signature in mouse male versus female cortex as a function of a) cellular subtype and b) zone of enrichment, confirmed more extensive proliferation within the progenitors in female mice (Fig. 8c, Supplementary Fig. 58). Thus, the previously described increased rate of adrenal cortex turnover in female mice[14] likely relies not on a larger number of progenitors but on an increased rate of their proliferation. In a similar analysis comparing proliferation activity between human male and female progenitors, statistical significance was not achieved (Fig. 8b,c)."

4) Figure 4 d and e are not discussed, and several plots in the manuscript are overlooked. Reorganization is necessary.

During the revision of the manuscript, this figure was fundamentally changed (see revised Fig. 5). The panels pointed out by the reviewer were removed and replaced by plots (revised Fig. 5d-f), which show transcriptomic differences between adult and fetal adrenocortical progenitor populations. The corresponding text in the Results section is the following:

"Comparisons of the mesenchymal, cortical and mesenchyme-to-cortex transitions between adult and fetal adrenal glands showed that the transition from ACTA2+/RGS5+ perivascular mesenchyme (shown in blue) and from DCN+ mesenchyme (shown in green) occur only in adult adrenals and that WT1+ fetal mesenchyme shows significant transcriptomic differences from adult mesenchyme, characterized by lower levels of IGFBP7 and other genes (Fig. 5c,e)."

"Finally, NR5A1+/EZH2+ cells were retained in much smaller although detectable numbers in the adult human adrenal gland. Importantly, the transcriptomic signature of fetal NR5A1+/EZH2+ progenitors differed significantly compared to that in adult, with higher levels of NR5A1 (gene coding for SF1) and MC2R (coding for ACTH receptor) (Fig. 5e)."

In the revised manuscript, we checked extensively whether all panels are cited and we discussed all relevant data.

5) In Figure 4c, it's unclear how transitions in human and mice were compared. Further explanation is needed to understand how unique transitions were identified in each species.

Our focus in this manuscript was the identification of transitions from mesenchymal populations to steroidogenic cells, since transdifferentiation within ZG/ZF/ZR obey the well-described centripetal model. After identification of VSMC-derived ZR/ZF cells and capsule-derived ZG cells in the single cell data set from human, we validated said transitions using our newly generated Stereo-seq data (revised Fig. 4, Supplementary Figs. 15).

Next, we identified two mesenchyme-to-cortex transitions in the single cell data set of mouse adrenal cortex, reflecting two states within the capsule (*WT1+/PTCH1+* and *WT1-/PTCH1+*) which

give rise to either ZF or ZG (Fig. 6a, Extended Data Fig. 9, Supplementary Figs. 55-56), also validated via high-resolution spatial transcriptomics (VisiumHD) (Supplementary Figs. 55).

The strategy of inter-species comparison was the following: 1) co-integration of single cell sets from the two species which would allow for identification of biologically similar populations (revised Fig. 6a-d, Supplementary Figs. 50-51), and 2) visualizing marker genes from adrenocortical/mesenchymal populations and known signaling pathways SHH, WNT and PKA from either human or mouse in both species-specific embeddings in the single cell and spatial data (revised Fig. 6e,f, revised Figs. 7, 8). We focused not only on whether a marker gene was found expressed in each species, but also whether it was present in the capsule/mesenchyme or within steroidogenic populations.

Please find our strategy and findings recapped in the section " Comparative analysis of human and mouse adult adrenal cortex" of the Results:

"To uncover the inter-species differences at a fine-grained scale, we generated single cell transcriptomic data from five male and six female adult mice, combined with existing data sets of three female and two male mice[43,44] (Supplementary Figs. 42-43). This integrated dataset was to ensure representation of rare subpopulations such as cycling progenitors and transient populations."

"Secondly, we generated a high-resolution spatial transcriptomics atlas of adult mouse adrenal glands from 3 males and 3 females using Visium HD (Supplementary Figs. 45-48). "

"We then performed detailed analysis of murine adrenal cortex and mesenchymal to steroidogenic transitions. After filtering and integration, adrenocortical and mesenchymal/perivascular murine populations were subselected and reclustered on a resulting UMAP of 25,932 cells with robust representation of mesenchyme, including capsule, and bridges between mesenchyme and either ZG or ZF (Fig. 6a), which we later compare to human adrenal gland structure (Fig. 6b-f).
First, we explored the basic composition of murine adrenocortical zones prior to inter-species comparisons."

"Finally, to carry out interspecies comparative analysis we constructed a combined alignment and UMAP embedding of both mouse and human adult adrenal cortex (Fig. 6b-d, Supplementary Figs. 50-51). Such embedding should allow to cluster cells with functional and transcriptional homology between the two species in the same domains. "

"Taken together, we established transcriptomic cellular homology between 1) human and mouse adrenal gland mesenchyme (VSMCs and capsule), 2) human and mouse aldosterone-producing ZG cells, along with the conserved pan-ZG marker Vsnl1/VSNL1 (Fig. 6c,d) and 3) a homologous cellular population with specialization on 3β-hydroxysteroid dehydrogenase activity in both human and mouse cortex, interestingly mediated by different genes (HSD3B2 in humans versus Hsd3b6 in mice) (Fig. 6e). Importantly, major signaling pathways appeared similar in humans and mice, specifically WNT4 and SHH expression in ZG (found lower in human cortex) and proliferative progenitors, despite differences in levels of expression (Supplementary Fig. 53-54, Fig. 8). Cycling NR5A1+/EZH2+ populations were found in both species, however they were more uniformly scattered throughout the cortex in human, while more enriched in ZG in mice. A key difference was also identified in the restricted expression of ZG marker genes (Cyp11b2 and Hsd3b6) in mouse, which were found

expanded into ZF in the human cortex (CYP11B2 and HSD3B2). This suggests that more cells in human adrenals are involved in endocrine regulation of kidney function (via aldosterone), which can be an adaptation to larger body size. Finally, Wt1+/Shh+ and Wt1+/Gata4+ progenitors play a major role in driving generation of ZG and ZF cells respectively in the murine cortex. Contrary to this, other mechanisms and stem cell sources have taken over this role of ZG/ZF renewal in human adrenals (VSMC-derived transition, capsular progenitors lacking WT1 expression in adult and proliferative NR5A1+/EZH2+ cells interspersed throughout the cortex). Taken together, our atlases reveal much deeper differences between mouse and human adrenal glands as compared to the previous knowledge, especially in the contexts of self-renewal and hormonal dedication of cortical cell populations. "

6) In Figure 6b, the depiction of the renewal process based on study results is informative, yet the relationship between ZF, ZG, and ZR remains obscure. It is crucial to clarify how these conclusions were derived from bioinformatics analysis. I believe the process involves examining whether transitions exist within minor clusters exhibiting transition-like expression patterns and considering their locations on the UMAP. But rationale behind this is needed and are there any experiments could be done to prove the conclusion?

To clearly improve how we reflect the situation with cortical transitions, we reanalysed the data and redesigned the figures (please see new *Supplementary Fig. 7a*.). We also wrote the following parts in the Results:

"We further confirmed zonation annotations by expression of Baker et al. ZG and ZR signatures (Supplementary Fig. 7a). ZR and ZG were the most distal clusters, converging at the top of the embedding, while ZF intercalated between the two (Fig. 2a). This continuity between ZG, ZF and ZR reflects known biology of the adrenal cortex with cells undergoing state transitions between the major cortical zones during adult adrenal gland self-renewal[11–14,22,23]. Despite the fact that our clustering reliably revealed cortical populations based on expression of combined specific marker genes, the transitions between these biologically-relevant clusters appeared smooth. Such transitions were generally characterized by directional gradual changes of gene expression programs – from one cortical zone to another, recapitulating previous knowledge[24,25]. This smoothness of transitions (ZG and ZR signature analysis, Supplementary Fig. 7a) is an important notion that must be kept in mind, even though the unbiased automated clustering generates sharp borders separating clusters (Fig. 2a)."

"ZF cells were found connected to ZG smoothly, showing a gradual and directional gene expression change. They also exhibited less heterogeneity, being separated into a central CYP11B2-/CYP17A1^{high}/CYP11B1+ population, along with two populations of NEAT1^{high} and CPB1^{high} cells respectively (Fig. 3a,b). NEAT1 is a long non-coding mRNA associated with the nuclear envelope and involved in some cancer types[31,32], while the CPB1 gene encodes a plausibly secreted carboxypeptidase, both with unknown functions in the adrenal gland."

Additionally, we provide RNA velocity results and re-clustering of cortex from humans and mice in several occasions and composition (Fig 3a, Supplementary Fig 32, 56, 57). All these results show the continual structure of gene expression change and, at the same time, biologically meaningful nature of devised clusters.

We understand that the reviewer is reasonable and is specifically interested in transitory cell states and their characterization and we share the same interest. However, within the context of this atlas, the analysis of fine transitory states within the adrenal cortex requires a huge amount of work and space (even for description), as it cannot be done using the unbiased clustering methodology – mainly because the transitions are smooth and gradual, and not abrupt. The standard automated clustering will always generate sharp borders between clusters that may be transiting into each other, whereas these borders can be accepted with a grain of salt and the remark of a compromise. This question must be tackled with biological experiments highlighting where the transition space becomes meaningfully leaning or invariably biasing towards one cell type or the other. For this, massive perturbation experiments are necessary to tease out the biology, which must be guiding fine cluster separation – contrary to how this is done in unbiased way (which has its own advantages and must be presented as objective instrument). We currently undertake this challenge as a separate project, and for sure it cannot be fit into the space and time of this revision.

Also, to address this point, we took advantage of new spatial transcriptomics data at single cell resolution to depict transitions between the zones not only transcriptomically, but also in physical space – on sections of human adrenals. We incorporated these data as an atlas with a convenient online interface ready for exploration by non-computational scientists (see Cell x gene in "Data availability" section of the revised manuscript). These spatial results were used to better understand the single cell data in revised Figs. 3, 4. Combined, these results generate an informative interpretation, which follows the centripetal model of differentiation within the adrenal cortex, which we have no reason to question and is further supported by the smooth transitions between zones in our UMAP (revised Figs. 2a, 3a). We also focused deeper on mesenchyme-to-steroidogenic transitions, which we identified as explained above and performed high resolution spatial transcriptomics (3 samples analyzed with StereoSEQ) for their validation (revised Fig. 4, Supplementary Figs. 13-15).

7) Also, the study did not explore the regeneration of cortical cells via ZG cells, which differentiate into ZF cells according to existing literature.

To address this, we performed the subclustering of ZG and ZF, which helped to navigate transitory and hybrid states (revised Fig. 3). We now complement it with new spatial data, which show that the transient state is quite confined in space as compared to main bodies of zones. Overall, we concluded that such a transition is again in agreement to the centripetal model. Experimental validation beyond our newly generated spatial data sets is challenging as we tried to model such transition using human iPSCs, and we were not able to produce cells resembling adult human ZG/ZR.

In addition, one of the individuals in our study carried several chromosomal alterations, as we reported previously (Gao et al., 2023). Upon examination of the cells with genetic aberrations, we identified that they largely contribute to ZG and ZF. The fact that the two zones shared genetically aberrant cells is in support of the centripetal model and ZG differentiation towards ZF. This interpretation can be found in the revised Results section:

"In a previous study by Gao et al.[36], we identified an individual carrying mosaic chromosomal alterations (mCAs), which can serve for cellular lineage reconstruction (Fig. 4g, Supplementary Fig. 31). The pattern of mCAs in this sample, composed of genetically normal ZR cells and clonally-linked aberrant ZG and ZF cells, supports a transition from VSMCs through TFPI2+/GNG11+ cells differentiating towards cells of the ZF and ZG, thus, validating the mesenchymal bridge. Cluster 3:TRANS-DCN also contained a high fraction of aberrant

cells, indicating potential presence of aberrant fibroblasts and the transition from 1:Fibroblasts towards transient cluster 3:TRANS-DCN. Finally, although the aberrant clone carrying mCAs predominantly contributed to ZG, the contribution of cells of this clone to steroidogenic cell populations of ZG and ZF is in agreement with the centripetal model of differentiation which supports transdifferentiation of ZG cells towards ZF[11–13]."

We also show the expression of genes driving ZG to ZF lineage conversion (Supplementary Fig. 53-54, Extended Data Fig. 5), which, as the reviewer noted, are well established in the literature, specifically SHH and WNT4/PKA signaling, as reviewed by (Lyraki & Schedl, 2021). The expression of these marker genes matched expected patterns. Thus, we focused our narrative around novel heterogeneity and mesenchyme-to-cortex transitions.

8) Many plots in Figure 6 are not discussed.

In the revised manuscript this figure is now presented earlier (revised Fig. 4) and redesigned to a large extent. We made sure to cite all panels, focusing on the transitions (panels a,b), mesenchymal markers, heterogeneity and signalling (panels c-f, h), along with using the clonal analysis in the individual with chromosomal aberrations (panel g) to trace the transition from VSMCs, validation of mesenchyme-to-steroidogenic cell transitions in Stereo-seq data and corresponding schematic (panels i,j,k).

The relevant description is found in the Results, in the section "Mechanisms of cell renewal of steroidogenic cells of the human adult adrenal cortex":

"Next, we shifted our focus towards all proliferative populations involved in adult adrenal cortex renewal, also forming transitions from mesenchymal cells (Fig. 4a,b, Supplementary Figs. 26-27). To enhance resolution of transitional states connecting to differentiated ZG/ZF or ZR, we limited subclustering to the minimal set of adjacent clusters composed of NR5A1+ cells relevant to transitions from the proliferative mesenchyme (clusters 7,8,9,10 and 12, Fig. 4a-e). Our systematic analysis of cell states bridging cortical and mesenchymal clusters, as well analysis of proliferation signatures, suggest three potential sources of new steroidogenic cells intercalated between discrete differentiated clusters: 1) transition 1 (from fibroblasts/capsule); 2) transition 2 (from vascular smooth muscle cells (VSMCs); and 3) a population of proliferating NR5A1+/EZH2+ cells that lack mesenchymal features but may represent differentiated cortical cells with cycling capacity as proposed from BrdU labelling in mouse adrenal glands[13] (Fig. 4a, Supplementary Figs. 26-27, Extended Data Fig. 5).

Transition 1 corresponds to the canonical transition described in mice from GLI1+/PTCH1+ mesenchymal stem cells of the adrenal capsule towards ZG cells[14–16] (Extended Data Fig. 5), characterized by Wt1 expression in rodents. Of note, WT1+ cells are found in very low numbers in the adult human cortex (Fig. 4c-f). When examining this transition from cells of cluster 1 (capsule, CCDC80+/FBN1+/FBLN1+/DCN+/NR5A1- cells mapped through Visium and subsequently Stereo-seq to the outer surface of the gland (Fig. 4h) towards cells of cluster 3 (ZG, NR5A1+/DCN+ cells, negative for capsular markers) we did not find intermediate transitory states with exclusive markers, suggesting fast direct differentiation of mesenchyme into cortex (Supplementary Fig. 28)."

"In a previous study by Gao et al.[36], we identified an individual carrying mosaic chromosomal alterations (mCAs), which can serve for cellular lineage reconstruction (Fig. 4g, Supplementary Fig. 31)."

9) While the idea of using Somatic CNV in human samples is good, it requires clearer explanation based on the plots generated.

We thank the reviewer for this question, and we reorganized the text and the figures to respond to this comment. In brief, the clonal analysis was used for the very important and specific task: to specifically validate identified mesenchymal-to-steroidogenic transitions in human data. The transcriptional embeddings and transcriptional similarity forming bridges in UMAP projections is not a lineage tracing, although it is likely to reflect the lineage connections. As we cannot lineage trace human organs, we are left with limited options to validate lineage transitions – and clonal inference is one of such options. Our clonal data in revised Fig. 4g and Supplementary Fig. 31 confirm that mesenchymal VSMC population is clonally connected with steroidogenic populations of adrenal cortex via distribution of a clone with CNVs and chromosomal abnormalities. Also, the distribution of this clone in cortical tissue supported the connectedness of steroidogenic populations in our embedding and fit the centripetal model. Please see the revised Fig. 4g and Supplementary Fig. 31.

We include this improved explanation of our results in the relevant part of the revised text below:

"In a previous study by Gao et al.[36], we identified an individual carrying mosaic chromosomal alterations (mCAs), which can serve for cellular lineage reconstruction (Fig. 4g, Supplementary Fig. 31). The pattern of mCAs in this sample, composed of genetically normal ZR cells and clonally-linked aberrant ZG and ZF cells, supports a transition from VSMCs through TFPI2+/GNG11+ cells differentiating towards cells of the ZF and ZG, thus, validating the mesenchymal bridge. Cluster 3:TRANS-DCN also contained a high fraction of aberranells, indicating potential presence of aberrant fibroblasts and the transition from 1:Fibroblasts towards transient cluster 3:TRANS-DCN. Finally, although the aberrant clone carrying mCAs predominantly contributed to ZG, the contribution of cells of this clone to steroidogenic cell populations of ZG and ZF is in agreement with the centripetal model of differentiation which supports transdifferentiation of ZG cells towards ZF[11–13]."

10) The data and code are not available

The data will be available to the scientific community after manuscript acceptance (EGA data sets EGAD00001011288 and EGAD50000000394 containing human single cell and spatial transcriptomic raw data and GEO accession GSE253852 for human and mouse spatial and mouse single cell data set), but to facilitate browsing of the data by the reviewers we have created the following Cell x gene domains for browsing all data sets:

https://adameykolab.hifo.meduniwien.ac.at/cellxgene_public/filecrawl/.review/.2024_Kastriti_Maksimov

Login: reviewer

Password: Cellxgene?AcceSS_ForRevie.wers

The code is available on Gitlab at https://gitlab.com/MaksimovDenis/adrenalglandscatlas (please see "Data and Code Availability Statement" of revised manuscript).

Reviewer #2: (expertise: single-cell genomics; adrenal gland biology)

Remarks to the Author:

In this manuscript, Kastriti and his colleagues provided a comprehensive analysis of the adrenal glands in humans and mice. The authors employed spatial transcriptomics to 4 adult human adrenal glands samples. Then they integrated published single-cell data to study cellular turnover, tissue microarchitecture and signaling in human and mouse adrenal glands. Their research revealed novel cellular diversity within the adrenal glands, including previously unknown progenitor cells and sex-specific differences in cholesterol biosynthesis. Meanwhile, this research identified potential sources of adrenocortical renewal and compared the mechanisms between humans and mice.

We thank the reviewer for providing the insightful and constructive advice and we hope the long experimental revision and careful revised phrasing will be satisfactory. Below we provide our approach to address the reviewer's comments.

One concern is the lack of experimental validation for the conclusions drawn in this research.

We understand this concern and indeed, it is impossible to run functional experiments on intact human organs because of obvious ethical concerns. Also, because of specifics of human adrenals, mouse is not a great substitute in this specific project.

To respond to this challenge, we improved the manuscript in the following ways:

1. During this revision, we generated new spatial transcriptomics data with single cell resolution, which validated the conclusions of the traditional single cell sequencing results (see revised Fig. 1). The newly generated spatial transcriptomics provided orthogonal experimental validations (Stereo-seq and Visium HD) on newly collected human adrenal tissue from three human individuals and adrenals from six adult mice (three males and three females) (revised Fig. 3, 7, Supplementary Figs. 13-15, 46-48). This allowed for robust validation of all cellular populations described and a more thorough interspecies analysis, as well as the generation of the resource for the adrenal gland scientific community.
2. We also performed RNAscope and immunostaining validations of our findings through single cell transcriptomics (new Extended Data Figs. 3-4, 6, Supplementary Figs. 10-11).
3. We improved the interpretation of clonal identification and lineage analysis in human adrenal cortex in revised Fig. 4. This analysis confirmed the major claims, such as mesenchymal VSMC populations contributing to adrenal cortex.
4. We thoroughly reanalyzed our single cell data subjecting it to stronger scrutiny.

Finally, in an effort to address this more experimentally, the first author of the paper visited the lab of a known expert in human iPSC-derived adrenocortical organoids, Dr Kotaro Sasaki (Mayama et al., 2025; Sakata et al., 2022). During her stay, the team exposed her to the recently developed in vitro model of adrenal cortex, but it was evident that it was not able to recapitulate the complexity of the human adult adrenal cortex, with all mesenchymal populations and cortical zones. This model rather reflected the fetal state. We will keep this collaboration going, although we cannot predict if adult cortical organoids will be developed in the nearest future.

Here are examples of our human cortical fetal-like organoids according to the protocol by Mayama et al, that we try to improve to generate adult-like cortex, grown in our laboratory after the visit of the first author to the lab of Dr. Kotaro Sasaki (currently undergoing analysis):

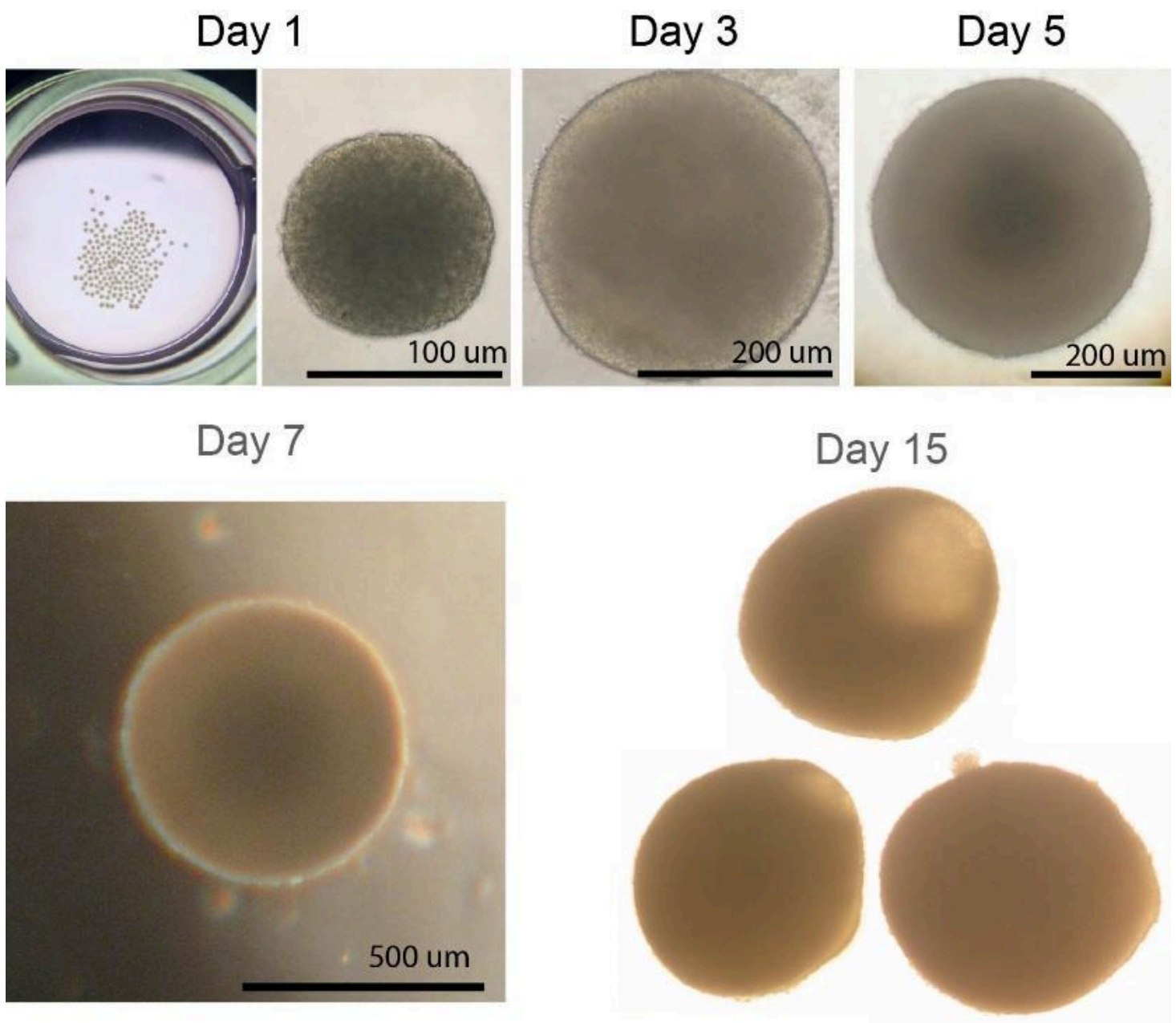

The article is descriptive, and many of the conclusions drawn have been reported in previous literature.

We understand this criticism and to address it we significantly expanded the novelty level in the revised manuscript (through the addition of new data, such as single cell-resolved spatial transcriptomics of mouse and human adrenals, as well immunohistochemistry and RNAscope based validations):

1. We revealed the existence of specific ZG populations, such as cortisol-producing HSD3B2/CYP21A2 cells, CCN3 and DLK1 cells possibly reflecting immature states, a VCAN^{high} population, all beyond known canonical *CYP11B2*+ aldosterone producing cells, and we validated of these populations using high resolution spatial transcriptomics (Stereo-seq) (revised Fig. 3, Supplementary Figs. 13-15), RNAscope and immunostainings (new Extended Data Figs. 3-4, 6, Supplementary Figs. 10-11).

2. We came up with three sources of de novo generation of steroidogenic cells, including capsule-derived ZG and novel VSMC-derived contribution to ZG/ZF and ZR and their validation in three donors through Stereo-seq (Fig. 4, Supplementary Figs. 13-15). Importantly, even though *EZH2*+/SF1+ proliferating fate-biased progenitor cells were assumed, we here obtained an extensive transcriptomic profile of these rare cells in the adult human cortex (both males and females).

3. We uncovered for the first time the fundamental differences between fetal and adult mesenchyme, which includes the ZG-maintaining capsule, with the fetal-specific *WT1* and *IGFBP7* expression and extensive proliferating SF1+ progenitors that are particularly underrepresented in the adult. Adult-specific features were the capsule- and VSCM-derived de novo steroidogenic cell generation.

4. We analyzed sexual dimorphism in adult and fetal human cholesterol pathway activity, along with confirmation of sexual dimorphism in the extent of ZG turnover in adult mouse cortex.

5. Lastly, we performed an extensive comparative analysis of human- and mouse-specific populations in the cortex, with striking examples being the *DCN*+ transitional population from capsule-to-ZG, VCAN, CCN3, DLK1 ZG populations, apart from known absence of expression of *SULT2A1* and *CYP17A1* in mice. This was complemented by the notable finding of different isoforms of

3β-hydroxysteroid dehydrogenase in the two species (revised Fig. 6e) which highlights an interesting direction for Evolutionary Biology.

6. Last but not least, we re-built the spatial atlas of mouse and human adrenal glands, both males and females, at single cell resolution, which will be a valuable resource for the community.

While our study confirms previously established facts such as the centripetal model of adrenocortical cell generation and the sexually dimorphic turnover observed in mice, our additional and new single cell and spatial transcriptomics data at single cell resolution allowed us to carry out original observations during this revision. We hope the amount of work we invested in this revision significantly improves the scientific content, the resource part and the logic of this story.

Several comments are listed noted below:

Although the novelty of the current manuscript lies in spatial transcriptomics, the authors only provide 4 visium data from **four human donors**. Since human samples are hard to acquire, I suggest the authors to supplement more visium analyses from mouse samples as well.

These experiments will strengthen some of the conclusions as well, such as sex-specific differences and human mouse differences. In addition, their spatial transcriptomic data needs further interpretation. Most of their findings from single-cell analyses are not integrated into visium data.

We thank the reviewer for this suggestion to improve the strength of our manuscript. During the revision we were successful at obtaining new material from three human donors which we analyzed through high resolution spatial transcriptomics (Stereo-SEQ). With these, we were able perform cellular deconvolution of identified mesenchyme-to-cortical transitions and subtypes of ZG and ZF cells, and validated marker genes, as well as the spatial distribution of rare EZH2+/SF1+ proliferating progenitors (revised Fig 4i, Supplementary Figs. 12-15, 33).

Additionally, to improve our interspecies comparison of human and mouse adrenal cortex, during the revision we analyzed the adrenals from three male and three female mice with high resolution Visium HD spatial transcriptomics (Supplementary Figs. 45-48). Combined with the newly generated Stereo-seq from human donors, we were able to address extensively human- or mouse-specific features of the cortex (revised Figs. 6, 7). Lastly, addition of new immunofluorescence and RNAscope in situ hybridizations in tissue from human donors validated our findings, especially observed ZG subtypes (revised Extended Data Fig. 3, 4, 6, Supplementary Fig. 10 and 11).

Fig1-2

This section analyzed spatial and transcriptional heterogeneity in normal human adrenal cortex, combining scRNA sequencing and spatial transcriptomics approaches. Higher transcriptional activity of cholesterol biosynthesis pathways is observed in male ZG and ZF compared to female, and is partly attributed to sexual dimorphism. **However, whether individual differences can be excluded from this conclusion based on the illustration of two samples (Fig 2e) remains unclear.**

We addressed this point by analysing our single cell transcriptomic data set from 11 individuals, which consistently showed increased activity of components of the cholesterol synthesis pathway specifically in males versus females. Furthermore, the cells that were most active in cholesterol biosynthesis are located in ZG and ZF of males, and, quite differently from this, in ZR of females. Thus, different cortical zones are mainly responsible for cholesterol biosynthesis in two sexes. We

clarified all observed cholesterol-related sexual differences in the revised version of figure and manuscript (revised Fig. 3e-j).

Furthermore, to answer this comment, we deepened the analysis of the cholesterol synthesis pathway by using adrenal gland bulk RNA sequencing data from the GTEx cohort (Lonsdale et al., 2013), which includes samples from 274 male and female individuals (new Supplementary Figs. 20). This analysis confirmed that the ratio of cholesterol production and uptake from blood to the production of steroid hormones in adrenals is increased in males, which we interpret as increased cholesterol balance. These data are discussed in the following section of the Results:

"Furthermore, transcriptional activity of genes of the cholesterol biosynthesis pathway showed statistically higher activity in male ZG and ZF compared to females (Supplementary Fig. 17), which led us to examine quantitatively the expression of genes involved in cholesterol uptake, synthesis, metabolism and catabolism (Supplementary Fig. 18).
Given the central role of cholesterol in steroidogenesis and observed sex-related differences in the respective genes, we systematically examined the balance between pathways for acquisition of cholesterol including de novo biosynthesis and uptake from blood, and its metabolic usage through steroidogenesis and esterification across different adrenal zones and sexes, extending this analysis to published bulk RNA sequencing data on a GTEx cohort of 274 individuals[35]. Approximating the overall pathway activity using the gene expression combined levels, we observed a clear correlation between rates of cholesterol uptake/synthesis and use across all cortical zones and individuals (Supplementary Figs. 19a, 20a, see Methods).
We then asked whether the increased cholesterol uptake/synthesis observed in male ZG/ZF (Supplementary Fig. 17c, Supplementary Figs. 19b) may be simply a reflection of increased steroidogenic activity, since hypothetically increased cholesterol uptake and synthesis can be explained by increased steroid hormone production. Modeling the overall cholesterol balance using a mixed linear model (Supplementary Figs. 19d, 20b), we find that this overall cholesterol balance is still increased male ZG/ZF (Fig. 3g,h, Supplementary Fig. 19e). This finding suggests a male-specific surplus in adrenal cholesterol production that exceeds the demands of steroidogenesis (Supplementary Fig. 20c), which may result in increased cholesterol levels in male adrenal tissue."

Fig3-4

1. The authors performed spatial transcriptomics on the adrenal glands of four individuals. However, when comparing sexual dimorphism in the adrenal cortex, gene expression data were only provided for two individuals (e.g., Fig 2e, sFig 8, 10-11). Furthermore, when focusing on the renewal of the human adult adrenal cortex, gene expression data was only presented for one male individual (e.g., Fig 3b-e, g, Extended Fig 5). It is essential to present key gene expression data from other individuals to support the authors' findings.

We did that now: in the new redesigned supplementary figures, we are including spatial visuals from all individuals from Visium and StereoSEQ data (3 individuals in addition to Visium 4 individuals, now 7 in total) (Supplementary Figs. 15, 26-27). Next, we organized an online exploratory tool Cellxgene providing embeddings and gene expression for all individuals, so the readers will be able to navigate all human and mouse samples and enquire the expression of marker genes and their distribution systematically. Please find the CellxGene interface available at:

https://adameykolab.hifo.meduniwien.ac.at/cellxgene_public/filecrawl/.review/.2024_Kastriti_Maksimov

Login: reviewer

Password: Cellxgene?AcceSS_ForRevie.wers

We hope this systematic approach will make the reviewer satisfied with the introduced changes.

2. In the section on the renewal of the human adult adrenal cortex, the authors observed a "gradient" of DCN expression in transition 1 (line 289) and "gradients" of ACTA2, RGS5, PLCXD3, and STEAP4 expression in transition 2 (line 295). It is unclear how they defined the "gradients" of the given genes from the gene expression plot.

First of all, we understand the importance of this question. The "gradient" is defined as a gradual smooth change of gene expression from one cluster to another evident in UMAP embedding and trajectory analysis. This is typically happening in transition zones amongst hybrid cell phenotypes. Such an example can be seen in revised Supplementary Fig. 7a, 14, 21. To avoid any confusion, we removed the wording involving that gradient of markers, and instead the following phrasing is used:

"Inferred transition 2 (Supplementary Fig. 29) from ACTA2+/RGS5+/MYH11+ VSMCs contributes through an RGS5+/ACTA2- transitory state to ZF and ZR (according to the UMAP representation of single cell transcriptomes) and possibly to ZG adrenocortical cells through intermediate states. Specifically, this is an intermediate step of differentiation from cluster 9, composed of RGS5+/ACTA2- cells, to cluster 10, composed of STEAP4+/TFPI2+ cells, depicted as transition 3 (Supplementary Fig. 30)."

"According to observed gene expression of ACTA2, RGS5, PLCXD3, STEAP4 in single cells and Visium in VSMCs and steroidogenic cells (and other genes shown in Supplementary Figs. 26-27), this transition mainly drives renewal of ZR."

"We further confirmed zonation annotations by expression of Baker et al. ZG and ZR signatures (Supplementary Fig. 7a). ZR and ZG were the most distal clusters, converging at the top of the embedding, while ZF intercalated between the two (Fig. 2a). This continuity between ZG, ZF and ZR reflects known biology of the adrenal cortex with cells undergoing state transitions between the major cortical zones during adult adrenal gland self-renewal[11–14,22,23]. Despite the fact that our clustering reliably revealed cortical populations based on expression of combined specific marker genes, the transitions between these biologically-relevant clusters appeared smooth. Such transitions were generally characterized by directional gradual changes of gene expression programs – from one cortical zone to another, recapitulating previous knowledge[24,25]. This smoothness of transitions (ZG and ZR signature analysis, Supplementary Fig. 7a) is an important notion that must be kept in mind, even though the unbiased automated clustering generates sharp borders separating clusters (Fig. 2a)."

"ZF cells were found connected to ZG smoothly, showing a gradual and directional gene expression change. They also exhibited less heterogeneity, being separated into a central CYP11B2-/CYP17A1high/CYP11B1+ population, along with two populations of NEAT1^{high} and

CPB1^{high} cells respectively (Fig. 3a,b). NEAT1 is a long non-coding mRNA associated with the nuclear envelope and involved in some cancer types31,32, while the CPB1 gene encodes a plausibly secreted carboxypeptidase, both with unknown functions in the adrenal gland."

Previously, the low resolution Visium didn't allow us to confidently identify cellular position, especially for "states", defined by specific expression of few marker genes. Now we resolve this issue via obtaining high resolution spatial transcriptomic data (Stereo-seq), which provides the ability to directly identify any "subtype" or "state" in tissue as a specifically localized cells without vague terms such as "gradient".

Please see new figures (revised Fig. 4, Supplementary Figs. 13-15) with true single cell resolved spatial transcriptomics for human adrenals visualizing the transitions mentioned in the relevant section of the Results.

3. The authors uncovered a novel central SF1+ adrenocortical progenitor population and suggested that these cells are distributed throughout the cortex. They should validate their findings using immunofluorescence or other methods.

In the revised manuscript, we validated this population (proliferating SF1+/EZH2+ cells) by the orthogonal method - high resolution (single cell) spatial transcriptomics (StereoSEQ) and found it interspersed throughout the cortex in all independent samples examined (three cases, revised Fig. 4, Supplementary Fig. 15). Additionally, we performed immunostainings in several donors, showing that proliferating cells are found in low numbers throughout the cortex, with an enrichment in ZG, while several proliferating steroidogenic columns were also detected within ZF (please see revised Extended Data Fig. 10, Supplementary Fig. 33).

4. In line 366, the authors re-clustered the fetal mesenchyme, but the characteristics of these subpopulations do not seem to be mentioned thereafter.

To respond to this comment, we now break down differences shown in the dot plot comparison between fetal and adult (revised Fig. 5e), where we show the gene expression signature of fetal adrenal mesenchyme in comparison to adult mesenchyme in adult human adrenals. That analysis showed that fetal mesenchyme was composed of many clusters, decreases in representation as development progresses and differs transcriptionally from adult mesenchyme by harbouring a subcluster with *WT1* expression and lower levels of *IGFBP7* overall, whereas the adult mesenchyme has higher levels of *IGFBP7* and unique expression of *RGS5*.

The observed fetal mesenchyme heterogeneity may be interesting for the community and drive future studies. Of note, origin, cell type composition and role in adrenocortical growth of human fetal adrenal capsule have not been extensively studied.

In the text of Results, we now clarify the findings of this analysis:

"Fetal mesenchyme analysis revealed predominant expression of PTCH1, RSPO3+ and a WT1+/DCN+ mesenchyme subpopulation (Supplementary Fig. 40a-d). Compositional dynamics showed a decrease of mesenchymal clusters and adrenocortical mesenchymal progenitors as development progressed, while the numbers of FZ cells gradually increased (Supplementary Fig. 41), while WT1+ mesenchymal cells are nearly absent in adult adrenals (Fig. 4e). Comparisons of the mesenchymal, cortical and mesenchyme-to-cortex transitions

between adult and fetal adrenal glands showed that the transition from ACTA2+/RGS5+ perivascular mesenchyme (shown in blue) and from DCN+ mesenchyme (shown in green) occur only in adult adrenals and that WT1+ fetal mesenchyme shows significant transcriptomic differences from adult mesenchyme, characterized by lower levels of IGFBP7 and other genes (Fig. 5c,e).”

5. In the section comparing the mesenchyme-to-cortex transitions in adult and fetal adrenal gland data, the authors simply projected the previous transitional cell population onto the integrated umap (Fig 4c). Is their any population in the visium data supporting this finding? Can you provide more validation using immunostaining? Can you label these transitional cells with EdU and perform single cell analyses experiments? Is it possible to do lineage tracing analyses based on somatic mutations?

We thank the reviewer for this comment. When it comes to the comparative analysis of fetal and adult, we didn’t observe co-integration of transitional (mesenchyme-to-cortex) populations (revised Fig. 5, Extended Data Fig. 8a-b), which indicates that most likely transitions we describe are adult-specific. Furthermore, we observed significant transcriptomic differences between adult and *WT1*+ fetal mesenchyme (Fig. 5e). As requested by the reviewer, we mapped adult mesenchyme to cortex transitions in new single cell resolution Stereo-seq spatial data (revised Fig. 4i, Supplementary Figs. 13-15). Also, the clonal analysis confirmed this transition because of the shared distribution of the clones with SNVs and chromosomal aberrations (through VSMC-to-cortex transitions) (revised Fig. 4g). We added the following text to the manuscript concisely expressing the differences observed between fetal and adult cortical transitions:

“Comparisons of the mesenchymal, cortical and mesenchyme-to-cortex transitions between adult and fetal adrenal glands showed that the transition from ACTA2+/RGS5+ perivascular mesenchyme (shown in blue) and from DCN+ mesenchyme (shown in green) occur only in adult adrenals and that WT1+ fetal mesenchyme shows significant transcriptomic differences from adult mesenchyme, characterized by lower levels of IGFBP7 and other genes (Fig. 5c,e).”

“Next, adult renewal appears to involve adrenal capsule-derived progenitors, contrasting with the fetal cortex at 6-7 weeks post-conception and onwards -a time during which a direct mesenchyme-to-steroidogenic transition is not evident, and a bridging population between the two is not observed in data (Extended data Fig. 7a). This is in agreement with studies recapitulating human fetal cortex development and the subsequent expansion of the fetal cortex through proliferation, rather than mesenchymal-to-steroidogenic transitions[5,39,40]. “

We also note that EdU and other labeling experiments are not possible in human intact tissue, for technical and ethical reasons.

<u>Minor points:</u>

1. In Extend Data Fig6 (revised Extended Data Fig. 5), the legend of the first dot plot was incorrect. The maximum fraction of cells expressing the given genes is only 9%, it seems that '0' was omitted.

We want to thank the reviewer for being so meticulous when reading the paper. In this figure, we are visualizing cells that express particularly low expressed genes, which reflects a small fraction of cells in our overall data set, thus leading to the observed small numbers in the scale.

2. In line 293, "Transition 3" should be modified to "Transition 2"

We want to thank the reviewer for spotting this issue. To fix this, we have modified the revised manuscript and are now referring to transition 1 as the canonical capsule-to-steroidogenic differentiation, while breaking down all stages of dynamic gene expression following the acquisition of steroidogenic markers, and complex transition with VSMCs-to-steroidogenic differentiation (see revised Fig. 4a), comprising two transitional cell clusters: cluster 9 (TRANS-RGS5) and cluster 10 (TRANS-TFIP2) (see revised Fig. 4a). Specifically, transition 2 is from VSMCs to TRANS-RGS5, while transition 3 is from TRANS-RGS5 to TRANS-TFIP2 and captures BRIDGE intermediate state (see revised Fig. 4k).

Please see relevant parts of the text below:

"Inferred transition 2 (Supplementary Fig. 29) from ACTA2+/RGS5+/MYH11+ VSMCs contributes through an RGS5+/ACTA2- transitory state to ZF and ZR (according to the UMAP representation of single cell transcriptomes) and possibly to ZG adrenocortical cells through intermediate states. Specifically, this is an intermediate step of differentiation from cluster 9, composed of RGS5+/ACTA2- cells, to cluster 10, composed of STEAP4+/TFPI2+ cells, depicted as transition 3 (Supplementary Fig. 30)."

3. Some plots in the manuscript are of poor readability. For example, in Extended Data Fig 6 (revised Extended Data Fig. 5), it is difficult for readers to determine from which umap the cell type cluster originates, despite some description in the figure legend. I suggest that the authors modify their figure legend to specify the source figure.

We agree and we improved the plots and their comprehensiveness throughout the manuscript. We also modified the relevant figure legend according to this suggestion (please see revised Extended Data Fig. 5) and hope that all visuals are of high quality.

Fig5-6

Firstly, it appears that most of the feature plots did not show bars for gene expression. In Fig. 5c, cluster 6/7/8/10 highly expressed Cyp11b2, which is a typical marker of zona glomerulata cells of the adrenal cortex. However, in Extended Data Fig. 9, dotplot showed that only cluster 6 seemed to have high expression of this gene, and the expression ratio was not high.

To address this, we changed the color palette used in the feature plots to match the one in the dotplots (see revised Fig 6, 7) and added bars for gene expression (scale of expression is now unified for all genes). The discrepancy between the two types of gene expression visualization is also due to the fact that dotplots depict averaged per cluster and universally scaled (z-scores) gene expression, while singular gene expression plots have a cell-level resolution.

In line 470-474, Gli1+/Ptch1+/ Wt1-cells mentioned were not shown in the figure, and I wonder if this group of cells can be more intuitively represented. The conclusion "Gli1+/Ptch1+/Wt1-cells contribute directly to dividing to Nr5a1+/Shh+/Wnt4+ adrenocortical progenitors " mainly came from the featureplots, which seemed to be not convincing.

We agree that the visual representation of the two populations was not optimal, so we introduced a panel showing *Wt1* expression in mouse capsule and upper bridge connecting the capsule to ZF in the revised Fig. 7k, to complement *Ptch1*, *Nr5a1*, *Shh* and *Wnt4* gene expression plots in Supplementary Figs. 53-54.

Our claim about these two populations was not novel, but we are confirming knowledge based on previous lineage tracing studies by *Gabrek* and colleagues and *Bandiera* and colleagues. These studies showed that only a few cells within the mouse capsule express *Wt1*, also co-expressing *Gata4*, and lineage tracing showed that they give rise to subcapsular Gli1+ progenitors at locations where *Shh* expression was seen by others (Huang et al., 2010; King et al., 2009), and differentiate according to the centripetal model.

However, our single cell embedding of adult mouse cortical cells shows two disparate transitional bridges from the capsule to ZF and ZG respectively. Specifically, the Wt1+ bridge (upper) gives rise to cells of ZF, while the Gli1+/Ptch1+/WT- bridge (lower) gives rise to Shh^{high}/Wnt4+ cells of ZG, as confirmed by our RNA velocity and pseudotime analysis (see Supplementary Figs. 55-56).

In Supplementary Fig. 29, the arrow of RNA velocity showed that cluster9 had a tendency to transform into other ZG cells. Could the authors explain this part in detail, such as showing the gene expression changes during transition?

We thank the reviewer for pointing out an interesting pattern in the velocity signal inside the mouse zona glomerulosa. To understand exactly how the transitions between intermediate states of the ZG are organized, we obtained focused embedding with increased resolution of mouse ZG and adjacent ZF-Ptn cells (see Supplementary Fig. 57) of the initial combined UMAP (see revised Fig. 6a). Cluster 9 from the old embedding turned out to be splitted into clusters 5 and 6 on the new embedding. The converging lines of the original velocity analysis converge precisely in the new cluster 5 (see Supplementary Fig. 57a). Then, we utilized a velocity re-analysis with the PAGA graph approach, which simplifies signals on the nearest neighbour graph to find any transitions between definite clusters (Supplementary Fig. 57b,d). Unfortunately, the results of this analysis were unstable on individual samples (Supplementary Fig. 57d), so we didn't manage to identify robust transitions (it is not clear which state goes where) and didn't manage to build a reliable pseudotime. Nevertheless, we provide an embedding with increased ZG clustering resolution (Supplementary Fig. 57a) and a DotPlot with extended marker gene signatures (Supplementary Fig. 57c), including those with weak differences between ZG cell states and likely to change during transitions. We also propose probable transition directions, based on the analysis of merged all samples (Supplementary Fig. 57b). These results, although providing some interesting observations, are not credible due to the variability between individual samples and the inconsistency with the results of lineage tracing experiments. In particular, the state of aldosterone-producing *Cyp11b2+* cells (cluster 4) is clearly terminally differentiated in our analysis, while being widely known to be able to transit into ZF (Freedman et al., 2013). Further research is needed to determine developmental relationships between finely-resolved ZG cell states.

In part of comparisons between adult human and mouse adrenal single cell, the authors primarily presented dimensionality reduction plots and dotplots of marker genes for each lineage in both species. However, a systematic cross-species comparison for each lineage was not explicitly detailed. Similarly, Fig. 5d-f only showed the expression differences of a few genes between the two species without in-depth analysis.

During the revision, we focused on improving our interspecies analysis and to this end we generated high resolution spatial transcriptomics from human and mouse adult adrenal cortex (Stereo-seq and Visium HD respectively, see revised Figs. 1, 3c, 6-7, Supplementary Figs. 13-15, 46-48). These novel data allowed for thorough comparison between the two species and unveiled a variety of human-specific ZG and ZF markers (i.e. ZG: *DCN*, *VCAN*, *CCN3*, *DLK1*, with *Dcn* and *Dlk1* found only in the mouse capsule), along with mouse-specific features such as *Ly6d* expression by specific ZG clusters and extensive *Ptn* expression (please see revised Figs. 6-8). To perform systematic cross-species comparison for each lineage we focused on ZG and early ZF, because ZR is clearly human-specific and ZF also significantly differs by human-specific 17α-hydroxylase expression (and cortisol vs corticosterone production). So, we performed focused re-integration of human and mouse ZG/early ZF (Supplementary Fig. 52) and observed some homology for *CYP11B2+/Cyp11b2+* (aldosterone-producing) and *HSD3B2+/Hsd3b6+* (3β-hydroxysteroid dehydrogenase) cell sates (Supplementary Fig. 52e, Fig. 6e-f), while marker genes of other ZG cell states were human- or mouse-specific (see revised Fig. 7). A key novelty was the identification of two disparate isoforms of 3β-hydroxysteroid dehydrogenase (type II in human versus type VI in mouse), which raises exciting evolutionary directions. On the other hand, we confirmed a common core of signaling pathways in the capsule and steroidogenic cells, namely WNT4, PKA/cAMP and SHH signaling (revised Supplementary Fig. 53-54).

All these observations are described in the new relevant text below in the Results:

"Interestingly, analysis of the expression pattern of 3β-hydroxysteroid dehydrogenase, involved in the synthesis of all steroid hormones, revealed differential distribution of the human-specific HSD3B2 (type II isoform) at ZG and ZF, versus the inner ZG-specific expression of the murine isoform Hsd3b6 (type VI isoform) (Fig. 6e,f), previously reported by an immunohistochemical analysis on mouse adrenal[47]. However, all other adrenocortical subclusters occupied species-restricted domains in the UMAP embedding, revealing significant transcriptional divergence despite general-level similarity (Fig. 6c,d, Supplementary Fig. 52), with a similar general spatial distribution of clusters based on functional specialization (Fig. 7a,b) revealed by an in-house developed spatial alignment procedure (Extended Data Fig. 10, see detailed Methods).

Examination of mesenchymal, ZG and ZF signatures and key genes in steroidogenesis revealed conserved expression of DCN in mesenchyme and CYP11B1 in ZF between mouse and human, while cortical DCN, VCAN, CCN3, CYP17A1, SULT2A1 were confirmed as human-specific (Fig. 7c,d). Of note, there is the major difference in expression of Notch inhibitor Dlk1/DLK1 in ZG in human versus mesenchyme (capsule) in mice, suggesting differential regulation of Notch signaling and cell-cell communication in mice and humans (Fig. 7c). Accordingly, the mouse ZG-specific Ly6d gene turned out to be a pan-cortical marker in human adrenals (Fig. 7e). At the same time, Ptn, gradually expressed in mouse ZG, was mosaically expressed in human (Fig. 7e). Taken together, we conclude that only some adrenocortical steroidogenic subtypes are clearly conserved between humans and mice, whereas others were subject to notable evolutionary changes in gene regulation within the adrenal tissue, similarly to significant differences between human and mouse adrenal capsule."

In the last section, the authors demonstrated lineage tracing using somatic mutation, but the process of the analysis was not specified in the methods, and the conclusions drawn from the results were not solid enough. Furthermore, the methods section contained an extensive explanation of

dimensionality reduction and clustering processes. It raises the question of whether the presentation of the methods could be restructured for clarity and conciseness.
We want to thank once again the reviewer for this constructive comment. In the revised version of our Methods, we introduced the following changes:

1. Included the description of analysis of Stereo-seq and Visium data sets
2. Introduced a separate section on the CNV analysis
3. Moved the detailed information about integrations and re-clusterings into Supplementary Note.

The latter significantly simplified the Methods structure and shortened. We hope they are now clear enough.

Reviewer #3: (expertise: sex-related differences adrenal gland)

Several single cell transcriptomic studies of the adrenal gland have been published in recent years, but the present manuscript is by far the most detailed and includes scRNA-seq and spatial (visium) RNA-seq data from adult samples from both sexes in human and mouse. In addition, meta-analysis on already published developmental data was performed. The major claims can be summarized as follows: 1) the human zG is comprised of a complex mixture of cell types with a 'novel' cell type that is proposed to express 11 deoxycortisone. Mouse zG is suggested to be less complex. 2) Sexual dimorphism of adrenal cortical cells appears to involve differential cholesterol biosynthesis between sexes. 3) Bioinformatic analysis suggests the existence of several progenitor cells that build and maintain the adrenal cortex in humans. 4) Integration of fetal and adult human data appear to have distinct routes of progenitor recruitment. 5) mouse adult adrenals appear to show lower cellular complexity and use routes of recruitment that differ from those in humans.
Taken together this atlas provides important information about adrenal biology with findings that are potentially of high interest to the scientific community.

We thank the reviewer for their thorough analysis of our study. We invested additional 1.5 years of work to complete the spatial part of mouse and human adrenal atlases and bring them to single cell resolution. We carefully attended every comment and did our best and what was techinically possible to address everything.

However, as it stands, the conclusions are based entirely on bioinformatic analyses, which must be interpreted with caution as they depend on proper normalization, data integration, and interpretation and can lead to misleading hypotheses. It is therefore essential to carry out orthologous studies to corroborate their claims.

Some major points are summarized below:

We fully agree that even though bioinformatics are a powerful tool in inferring biologically relevant conclusion, we also felt the need to perform orthologous studies, and for this reason we opted for single cell resolved spatial transcriptomics in human and mice, which we added in this revision (revised Fig. 3, 4, 6ef, 7, Supplementary Figs. 12-15, 33, 45-48, 58). We also performed additional validations through in situ hybridization and immunofluorescent stainings to validate our findings (please navigate to Extended Data Figs. 3-4, 6, Supplementary Figs. 10-11 and relevant descriptions in corresponding sections of Results).

Last but not least, we have inserted a relevant disclaimer in the Discussion section regarding the interpretation of the data and necessary further validation in vivo or in vitro:

"Importantly, we recognize that our proposed model of adrenocortical renewal, centered around several inferred transitions, is inherently limited by the observational nature of human transcriptomic data including RNA velocity and directional gene expression changes and are not validated by experimental evidence in intact human adrenal tissue. To strengthen the validity of our inferences, we complemented our scRNA-seq analysis with multiple orthogonal approaches, including single-cell resolution spatial transcriptomics and lineage insights gained from a rare clonal mosaic case, which together support the existence of revealed mesenchymal-to-steroidogenic transitions. Even so, we invite the readers to

interpret our findings with caution and emphasize that definitive evidence for these lineage relationships will require functional validation, which is currently not feasible in human tissue."

We provide a point-by-point response below:

Major points:

1. The paper is very dense and hard to read and would benefit from reorganizing and streamlining.

We agree with the reviewer and we rewrote extensively the text during the revision to ensure that findings and narrative are easy to follow. The reorganized Results sections proceeds systematically according to a specific question we were investigating. These are the new subsection headings we introduced to improve the flow:

"Cell type heterogeneity of the human adrenal gland"

"Mechanisms of renewal of steroidogenic cells in the human adult adrenal cortex"

"Comparison of adult and fetal human adrenocortical heterogeneity and transitions"

"Comparative analysis of human and mouse adult adrenal cortex"

We also re-structured the Discussion section by improving the sequence of arguments and overall compaction. We hope the new version is improved and will satisfy the reviewer and readers.

2. Human samples come from 11 different individuals with highly diverse genetic background, ages and stress levels. While it is good to 'pool' samples to identify common mechanisms it is equally important to identify differences, in particular when subpopulations are identified. What is the distribution of each cell type in the different individuals? (this information is somewhat available in suppl. Figure 2, but it would be good to show a bar plot with the percentage of each cluster). Does the heterogeneity in the zG persist when clustering individuals?

To clarify this point in the revised manuscript we show the distribution of all major cell types across key UMAPs in each donor (Supplementary Fig. 3). We also performed individual reclustering of 8/11 individuals, each one containing at least 800 ZG & ZF cells of good quality (Supplementary Fig. 9), heterogeneity persisted to a large extent.

Overall, we observe that some of the older individuals have higher proportions of immune and endothelial cells versus adrenocortical, which is expected as aging takes place. In relation to adrenocortical cells, all major subpopulations were represented across individuals, with some deviations in proportions of different zones that can reflect microniches within the human adrenal cortex, possibly due to the limited sampling area. We further show ZG subcluster marker gene expression in the newly generated high resolution spatial transcriptomic data (two individuals shown in revised Supplementary Fig. 14 and all three individuals in the explorable Cell x gene: *https://adameykolab.hifo.meduniwien.ac.at/cellxgene_public/filecrawl/.review/.2024_Kastriti_Maksimov*

Login: reviewer, Password: Cellxgene?AcceSS_ForRevie.wers

zG complexity

3.While some cellular diversity within the zG has been previously reported, the authors claim this zone to be highly heterogenous. The identification of a new subpopulation of ZG cells (Cluster 5 - HSD3B2 positive and CYP11B2 negative ZG cells) is potentially of high interest, but the authors base their analysis entirely on the localization of HSD3B2 expression using Visium. The resolution of the 10x Genomics Visium is too low to identify these cells. Immuno- and/or high resolution ISH (e.g. RNA-Scope) analyses have to be carried out to identify these cells in vivo to confirm their localization and the hypothesis that they do not express CYP11B2. Is there a particular position within the rosette structures of the zG that harbors these cells? HSD3B2 is a key enzyme in steroid production and is therefore expected to be found in all steroidogenic cells (see for example the human protein atlas). How do the authors reconcile their findings with these data?

During the revision we addressed this by 1) performing subselection and re-clustering of ZG and ZF in order to uncover specific markers for the subclusters (revised Fig. 3), and b) we performed new high resolution spatial transcriptomics on newly collected human adrenal biopsies from three tissue donors with Stereo-seq (revised Fig. 3 and Supplementary Fig. 13, revised Fig. 6ef). Thus, we investigated thoroughly potential subpopulations and validated them orthogonally. Using immunofluorescence and RNAscope in situ hybridization, we detected HSD3B2+ cells in ZG and ZF and several HSD3B2high cells with either low or negligible levels of CYP11B2 in variable positions within small CYP21A2+ cellular clusters/rosettes at outermost locations of ZG (Extended Data Fig. 4, Supplementary Fig. 11). These cells did not occupy a specific location within the rosettes, but they were identifiable in every tissue donor analyzed. This is supporting the idea that glucocorticoid production in human adrenal cortex is not exclusive to ZF but is additionally taking place by cells of ZG. This is not in disagreement to what is known about HSD3B2 expression, which we would like to note it is higher in ZG and ZF, but much less in ZR (see spatial transcriptomics in revised Supplementary Fig. 14), in accordance to stainings in the Human Protein Atlas (https://www.proteinatlas.org/ENSG00000203859-HSD3B2/tissue/adrenal+gland#imid_152) and a proteomic/transcriptomic study by Baker and colleagues (Baker et al., 2021). In addition, cells annotated as ZG-HSD3B2 in Stereo-seq data were located in both ZG and ZF (Supplementary Fig. 13c, revised Fig. 7a) with preferences to ZF in younger tissue donors and ZG in the aged individual.

This interpretation of our data is mentioned in the following part of the revised manuscript:

“In addition to the expected classical CYP11B2^{high} and more recently described CCN3^{high} cells of ZG, we identified a previously unrecognized steroidogenic population distinguished by higher expression levels of HSD3B2 compared to other ZG and ZF cells[21–23] (cluster 5, Fig. 1a, cluster ZG-HSD3B2, Fig. 3a,b). To further investigate the functional identity of this cluster, we analyzed the expression of key enzymes involved in glucocorticoid biosynthesis and identified the co-expression of HSD3B2, CYP21A2, and CYP11B1. Based on their transcriptional profile, we hypothesize their involvement in pregnenolone (17OH-pregnenolone) to progesterone (17-OH progesterone) conversion via HSD3B2 enzymatic activity and progesterone (17-OH progesterone) to 11-deoxycorticosterone (11-deoxycortisol) via CYP21A2 (expressed in all cortical zones but in higher levels in the HSD3B2+ cluster) (Fig. 2b-d). These cells lack CYP11B2 required for aldosterone synthesis despite expressing CYP17A1+/CYP11B1+ (Fig. 3b), suggesting direct cortisol production within the ZG, as supported by a previous proteomic study[33].”

Moreover, the authors predict cluster 5 cells to produce cortisol, which is simply inferred from the transcriptomic data. A functional confirmation is missing.

We appreciate the reviewer's concern regarding the functional validation of steroidogenic activity in cluster 5. Indeed, our inference that these cells contribute to cortisol production is based on transcriptomic data. To explore this further experimentally, we initiated a collaboration with the lab of Dr. Kotaro Sasaki (University of Pennsylvania), where the first author attempted to model adult adrenocortical function using iPSC-derived adrenal organoids. Unfortunately, as also reported in prior studies (Mayama et al., 2025; Sakata et al., 2022), these organoids largely recapitulate fetal adrenal zones, but do not accurately reflect adult adrenocortical steroidogenic profile. Thus, we were unable to achieve functional confirmation of adult ZG/ZF activity in this system.

However, in the recently submitted manuscript in Biorxiv, the team shows proof of cortisol production by the transient-zone-like cells they model within their iPSC-derived organoids. These cells are transcriptomically similar to our cluster 5 (expressing *HSD3B2* and *CYP21A2*) and the authors show ACTH-dependent cortisol secretion through ELISA (Mayama et al., 2025).

In our case, in lieu of functional assays, we performed a more detailed analysis of our single-cell and spatial data. Cells in cluster 5 (see Fig. 2, and revised reclustering in Fig. 3b) express high levels of *HSD3B2*, *CYP21A2*, and *CYP11B1*—enzymes that together support the biosynthesis of progesterone, 11-deoxycortisol, and ultimately cortisol. In the revised manuscript, we now present combined RNAscope and immunofluorescence data showing co-expression of *HSD3B2* and *CYP21A2* in defined cellular clusters with ZG (Supplementary Fig. 11). While direct functional validation (e.g., hormone measurements) is not feasible from fixed tissue, the robust expression of these key steroidogenic enzymes strongly supports the identity of these cells as functionally competent cortisol-producing cells.

We are describing the validation of this cluster in the tissue and the rationale behind the interpretation of our results in the following new part of the manuscript:

"To further investigate the functional identity of this cluster, we analyzed the expression of key enzymes involved in glucocorticoid biosynthesis and identified the co-expression of HSD3B2, CYP21A2, and CYP11B1. Based on their transcriptional profile, we hypothesize their involvement in pregnenolone (17OH-pregnenolone) to progesterone (17-OH progesterone) conversion via HSD3B2 enzymatic activity and progesterone (17-OH progesterone) to 11-deoxycorticosterone (11-deoxycortisol) via CYP21A2 (expressed in all cortical zones but in higher levels in the HSD3B2+ cluster) (Fig. 2b-d). These cells lack CYP11B2 required for aldosterone synthesis despite expressing CYP17A1+/CYP11B1+ (Fig. 3b), suggesting direct cortisol production within the ZG, as supported by a previous proteomic study[33]. Although direct hormone measurement in our samples was not possible due to the nature of fixed tissue, we performed combined RNAscope in situ hybridization and immunofluorescence, confirming spatial co-expression of HSD3B2 and CYP21A2 within ZG, as well as several HSD3B2+/CYP11B2- cells within classical rosettes (Extended Data Fig. 4, Supplementary Fig. 11). These findings provide orthogonal support for the steroidogenic role of this cluster and reinforce our inference of its functional relevance."

4. Lines 166-182: The authors performed reclustering focusing on a subpopulation of cells and identified 6 subclusters that they assigned to the zG. The basis for this assignment (zG versus zF) is unclear to me. Indeed, when looking at extended data 2 (panel e), it seems that zG-CCN3 (subcluster

5) and zG-DLK1 (subcluster 6 of the focused clustering) are transcriptionally more related to the zF than the zG. The visium data suggest CCN3 and DKL1 to be enriched in more cortical regions, but perhaps these are cells that are in the process of transitioning from zG to zF?

We recap the rationale behind our annotation of ZG and ZF cells in the revised manuscript (revised Fig. 2e) as follows, according to findings of Baker and colleagues from their zone-specific bulk proteomics/transcriptomics:

“The resulting embedding of the 39,985 sub-selected cells revealed continuous transitions of transcriptional states (Fig. 2a), which we annotated using established markers of adrenocortical subtypes from a proteomic and transcriptomic study by Baker and colleagues[21] and known enzymes of steroidogenesis (Fig. 2b,e): CYP11B2, KCNJ5, LGR5, WNT4, CCN3, VSNL1-expressing cells represented aldosterone-producing ZG, CYB5A, SLC13A5, SLC27A2 and HSD3B2^{low}-expressing the androgenic ZR, while ZF was represented by the rest of STAR+ cells, expressing high levels of genes CYP11B1/CYP17A1/HSD3B2 involved in glucocorticoid production (Fig. 2b-d). We further confirmed zonation annotations by expression of Baker et al. ZG and ZR signatures (Supplementary Fig. 7a).”

Further finer analysis of subpopulations within the major cortical zones and identification of the DLK1 population was performed in an unbiased manner and during the revision we addressed this population, along with CCN3+ cells, through our novel Stereo-seq spatial transcriptomics, immunostainings and RNAscope. With all said methods we found CCN3 strongly expressed in ZG and in apical ZF in a mutually exclusive pattern with the classical ZF/ZR marker CYP17A1, except for a few cells (revised Extended Data Fig. 3, Supplementary Fig. 14). We observe some deviation between mRNA and protein, which is important to consider (revised Extended Data Fig. 3). In addition, cells annotated as ZG-CCN3 in Stereo-seq data were preferentially located in ZG (Supplementary Fig. 13c, revised Fig. 7a). As for ZG-DLK1, this transcriptional state was present in both ZG and ZF (Supplementary Fig. 13c, revised Fig. 7a) with preferences to ZF in younger patients and ZG in the aged individual. In agreement to our Stereo-seq data (Supplementary Fig. 14), immunofluorescent signal for DLK1 was detectable in high levels in columns within the ZF, and found co-expressed with CCN3 in very few cells within ZG through immunofluorescence (see revised Extended Data Fig. 3). However, these two states were transcriptomically similar (revised Fig. 3b) and represented a single cluster 6:ZG-CCN3 in the initial clustering analysis (Fig. 2a,c, Supplementary Fig. 8a-d), that's why we annotated *DLK1*+ cell state with prefix ZG-, despite the fact that it occupies more than one zone - to emphasize that it is part of the lineage originating in ZG. Such an interesting pattern of transcriptional heterogeneity and spatial arrangement may indeed, as the reviewer points out, indicate a differentiation pathway. However, we cannot confirm or refute this due to poor interpretability of velocity analysis results (Supplementary Fig. 32). Of note, both CCN3 and DLK1 proteins were seen in a gradient from outer to inner ZG/ZF, which indeed may point to their nature as transitional, especially when considering that DLK1 is a NOTCH signaling inhibitor. We are concisely mentioning our observations in the revised Results section:

“Detection of CCN3 through immunofluorescence combined with in situ hybridization placed CCN3^{high} cells within ZG and upper ZF, devoid of CYP17A1 (Supplementary Fig. 8, Fig. 3b, Extended Data Fig. 3a-c). On the other hand, DLK1 and CCN3 immunostaining revealed DLK1 enrichment in cellular columns within ZF, while it was absent in ZG, as opposed to CCN3 protein only in outer ZF (Extended Data Fig. 3d,e). This pattern of presence of CCN3 and DLK1 within the CYP17A1^{low}/CYP11B1+ population (potentially cells slowly differentiating towards

ZF) reflects transcriptional states of an intermediate steroidogenic population towards maturation."

"Using Stereo-seq[34] to analyze cortical regions of three donors (two men, one woman, ages 13-41), we visualized the distribution of the subpopulations across cortical zones on tissue sections (Supplementary Figs. 12-13, Fig. 3c-d). Combining an unsupervised approach to identify tissue patterns and scRNA-seq reference-based deconvolution approaches (see Methods), we uncovered the capsule, ZG, ZF, ZR and all subclusters and reconstructed the cellular composition in the cortex of donors (Supplementary Figs. 12-15). The expression patterns of key cluster-specific markers were consistent between scRNA-seq reference and annotated cells in spatial transcriptomics data (Supplementary Fig. 12 f,g). Most apical populations within ZG were VCAN^{high} and CCN3^{high}, CYP11B2^{high} and DLK1^{high} populations expanded further basally, while HSD3B2^{high} cells were positioned in expanded subcapsular domains (Fig. 3d, Supplementary Figs. 13-14)."

The authors need to take care to differentiate between slightly different cellular states (that could occur in a transitional process) versus a real cellular subtype.

To respond to this, in this revision, we generated new spatial transcriptomics, immunofluorescence and RNAscope data to visualize the transitions between cortical zones vs central zones and connect them to our main atlas. Please find these new data in revised Fig. 3, Extended Data Figs. 3-4, Supplementary Figs. 10-15. We additionally explored our 10x single cell transcriptomic data and generated specific UMAPs to visualize differentiated and transitory states (revised Fig. 3 and CellXGene app online (link above and "Data availability" section of the revised manuscript).

Taken together, our combined data – including freshly added single cell-resolved spatial transcriptomics – show transitory cells characterized by mixed/hybrid gene expression profiles. We believe that the readers will have a good understanding of what is a mixed transitory profile or a mature end-state by exploring the datasets of the atlas and seeing where the transitional processes take place rather directly. In the follow-up study we currently explore hybrid cell states in transitions reconstructing their mixed GRNs and setting up human embryonic adrenocortical organoids to test the drivers of transitions.

5. Cluster 2:ZG-VCAN: The use of VCAN as a marker for a zG specific subtype seems not particularly meaningful. According to the visium image (extended data Fig2e) VCAN expression appears to be scattered throughout the adrenal gland. How highly expressed is this gene? Additional cluster specific markers should be shown and the specificity confirmed using another method.

During the revision we performed more thorough analysis of cells of ZG/ZF, which resulted in the observation that cells of this cluster are largely negatively defined, with *VCAN* being almost the only positively discriminating marker cross-validated in ZG in our newly generated Stereoseq data set (Supplementary Fig. 14). Additionally, to improve upon the previous validation of VCAN expression in the low resolution Visium data, in the revised paper we show through RNAscope in situ hybridization combined with immunofluorescence that those cells are found within CYP11B1/2+ ZG rosettes and almost none or very limited coincidence with the ZF marker CYP17A1 (Supplementary Fig. 10). Our findings are supported by experiments addressing gene expression in ZG versus ZF by Zhou and colleagues (Hypertension, 2015), which identified VCAN as highly expressed within ZG along with VSNL1, DACH1, and CYP11B1/2 among others. However, there are no available data on the functional role of VCAN in the adrenal cortex, hence we refrain from elaborating further on this finding in the

revised manuscript. Thus, we mention the *VCAN*+ cluster within our data and its validation within ZG cells in the tissue without further speculation:

"ZG cells were divided in a) canonical aldosterone-producing VCAN+/CYP11B2+ cells (ZG-CYP11B2), b) VCAN^{high}/CYP11B2- cells (ZG-VCAN) corresponding to immature CYP11B2-/CYP17A1-/CYP11B1- cells in ZG[17], c) a HSD3B2^{high} cluster, d) a DLK1^{high} cluster, e) a CCN3^{high} cluster and f) a cluster enriched in genes involved in cholesterol metabolism (ZG-CHOL) (Fig. 3a,b, Supplementary Fig. 8)."

"VCAN detection through in situ hybridization validated its expression in ZG in CYP11B1/2+ steroidogenic rosettes and almost negligible levels in CYP17A1+ ZF (Supplementary Fig. 10)."

"Using Stereo-seq[34] to analyze cortical regions of three donors (two men, one woman, ages 13-41), we visualized the distribution of the subpopulations across cortical zones on tissue sections (Supplementary Figs. 12-13, Fig. 3c-d).... The expression patterns of key cluster-specific markers were consistent between scRNA-seq reference and annotated cells in spatial transcriptomics data (Supplementary Fig. 12 f,g). Most apical populations within ZG were VCAN^{high} and CCN3^{high}, CYP11B2^{high} and DLK1^{high} populations expanded further basally, while HSD3B2^{high} cells were positioned in expanded subcapsular domains (Fig. 3d, Supplementary Figs. 13-14)."

Lastly, we concisely recap the details of ZG heterogeneity our study uncovered, including VCAN+ populations, in the Discussion:

"Furthermore, we demonstrate that human ZG includes previously unrecognized cell states with coordinated gene programs regulating extracellular matrix composition, cell adhesion, migration, growth, proliferation and differentiation including the known ZG marker CCN3, NOTCH signaling inhibitor DLK1 or proteoglycan VCAN[55–57], pointing to complex signaling in adrenal renewal and zonal structure maintenance. Even though previous studies had independently uncovered these markers in human ZG[27,29,58,59], our analysis is carefully pinpointing the presence of numerous subpopulations with discrete markers. Overall, these results emphasize the complexity within human adrenocortical zones, instructive for further investigations in human organoid systems[39,60]."

<u>Sexual dimorphism:</u>

6. The authors found cholesterol synthesis associated genes to be enriched in male samples. Is there any evidence that AR signaling suppresses cholesterol synthesis?

Previous studies proposed a positive correlation between androgen receptor signalling and cholesterol biosynthesis and accumulation in in vitro models of prostate cancer (Han et al., 2018; Krycer & Brown, 2011), as well as hepatic cholesterol uptake in vivo and in vitro (Yuefeng et al., 2022). To our knowledge, this has not been investigated in the adrenal cortex, especially in human. Thus, we plotted the levels of genes related to cholesterol obtaining and androgen receptor expression or strength of androgen receptor signaling in all individuals from which we have single cell transcriptomic data, which did not reveal a positive or negative correlation (see plots below).

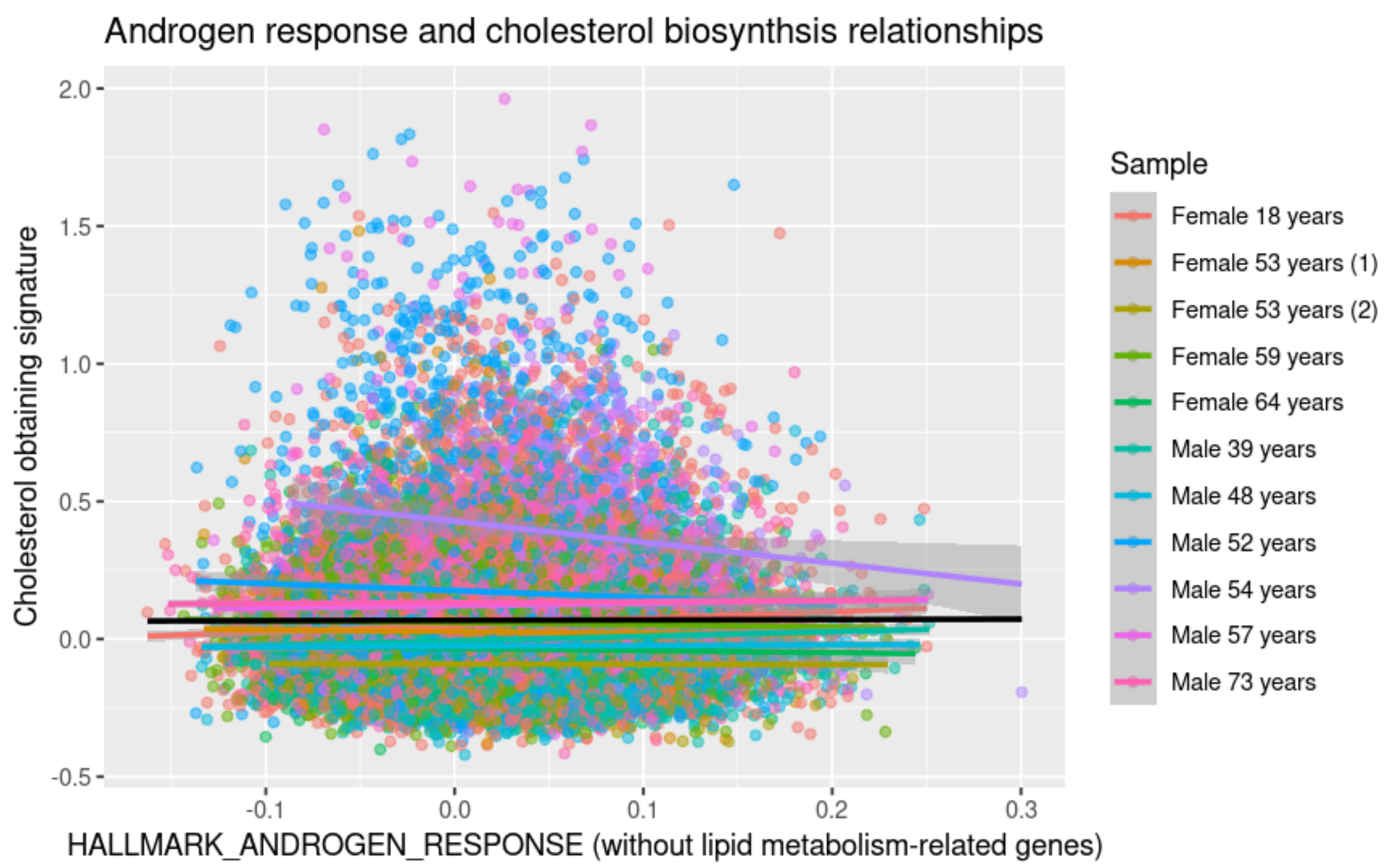

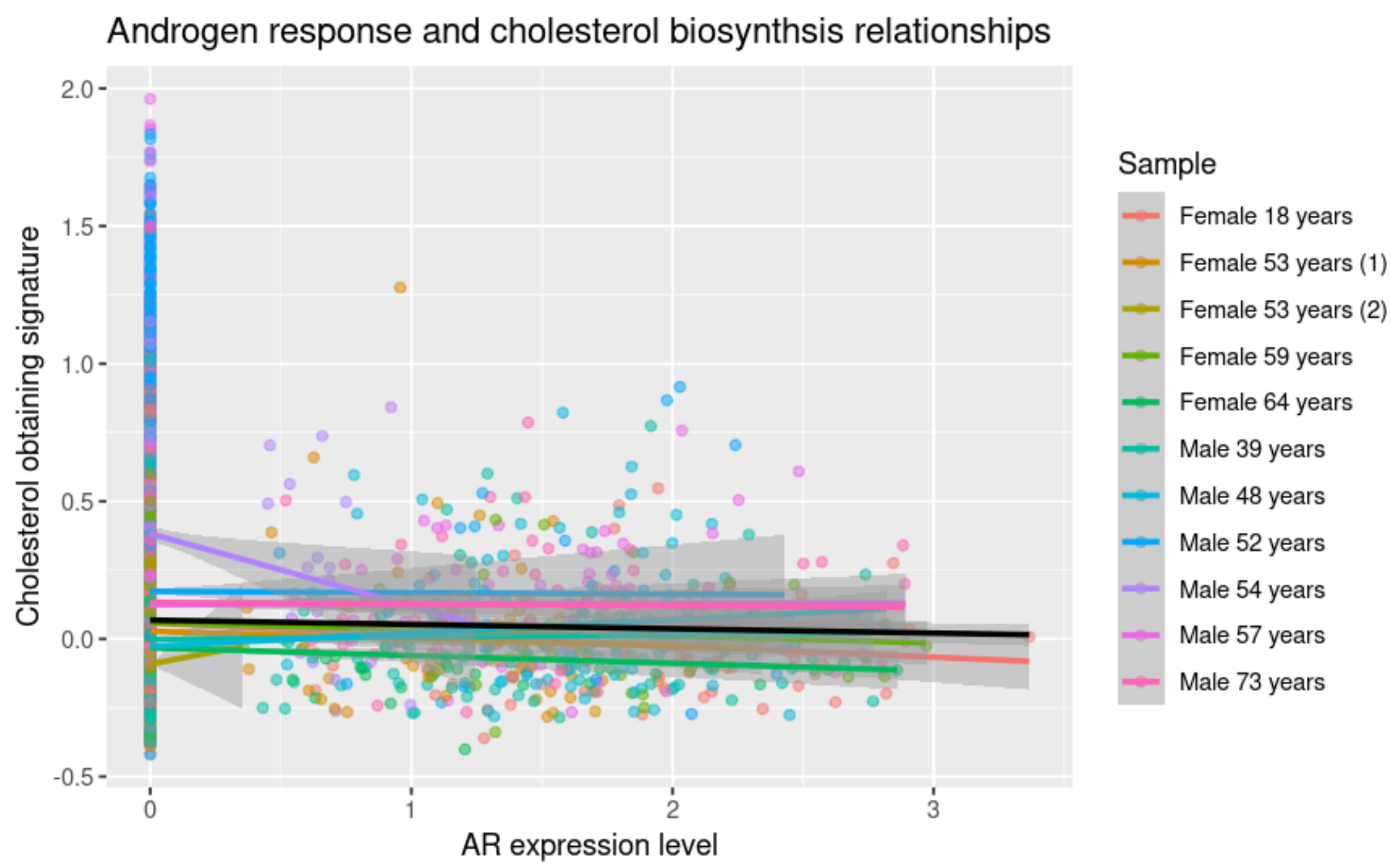

However, we have to point out that the gene coding for the androgen receptor is not expressed by all adrenocortical cells in the human gland (see figure below, panel d), as opposed to mice. Since to fully assess the effect of androgen receptor signaling requires further experiments and is not the focus of our study, we prefer not to include these negative results in the manuscript due to space limitations.

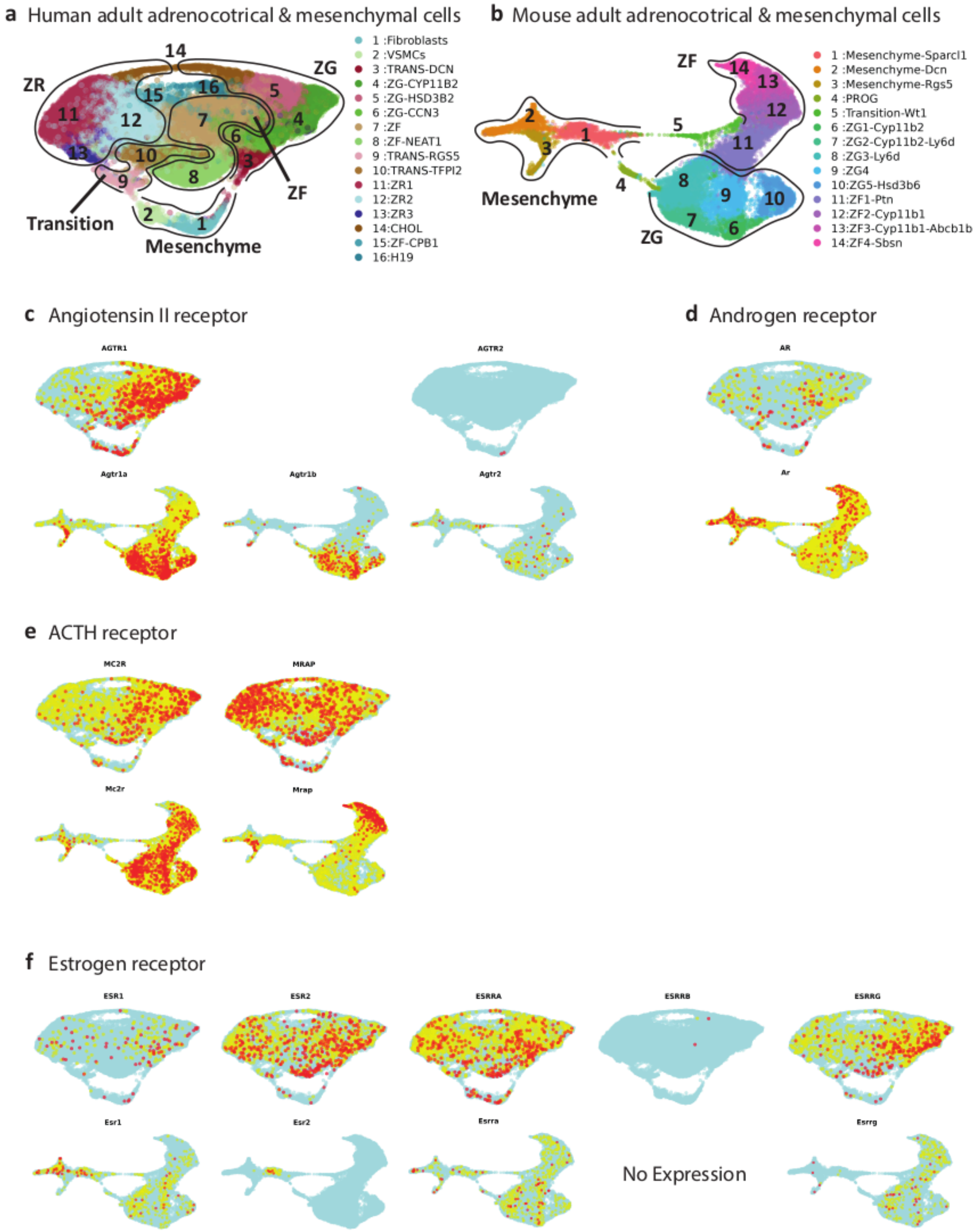

Without any additional data, the discussion on sexual dimorphism of cholesterol (line 706 to 744) is very speculative and needs to be shortened.

In the revised manuscript we made an extensive re-analysis of cholesterol metabolism in the male and female cortex, and report differential distribution of cells with increased cholesterol uptake and biosynthesis in the male ZG and ZF, along with enhanced cholesterol metabolism in female ZR versus male ZG (revised Fig 3, panels d,i).

Thus, we have accordingly shortened the relevant part of the discussion by concisely recapping our findings on the sexual dimorphic cholesterol metabolism we observed and briefly comment on a potential link between cholesterol and adrenocortical proliferation in cancer:

"In another example, the earlier studies have shown a negative effect of cholesterol deprivation on adrenal cortex cancer growth[53]. This is consistent with our results from The Cancer Genome Atlas analysis (Supplementary Fig. 59), which demonstrated a positive correlation between the cholesterol uptake signature and the adrenocortical progenitor signature, indicating the influence of cholesterol abundance on proliferative progenitor activity and potential tumor growth."

"With this approach, we observed differential expression of cholesterol biosynthesis pathways between men and women, suggesting sex-specific and spatially-organized regulation of steroidogenesis in adult human adrenals."

7. A recent study by the group of Morohashi has found that androgens suppress global gene expression (doi: 10.1016/j.celrep.2024.113715) in mouse adrenocortical cells. Do the authors find increased transcripts in female mouse samples? Is this conserved in humans? If yes, could this explain some of the differential expression between males in females in different subclusters?

To respond to this comment, we addressed the levels global gene expression in males versus females in our single cell transcriptomic data from human and mouse (please see figure below). We observed a slight increase in counts within human male ZF, although no statistically-significant trend was observed (in any cluster) after accurate testing (all p-values > 0.1 even before multiple testing correction) - see plot below. This is not surprising, since median RNA counts per cell is a popular metric for scRNA-seq quality control, which would reflect technical aspects (suspension preparation quality for the specific sample, cell viability etc.) rather than actual RNA content.

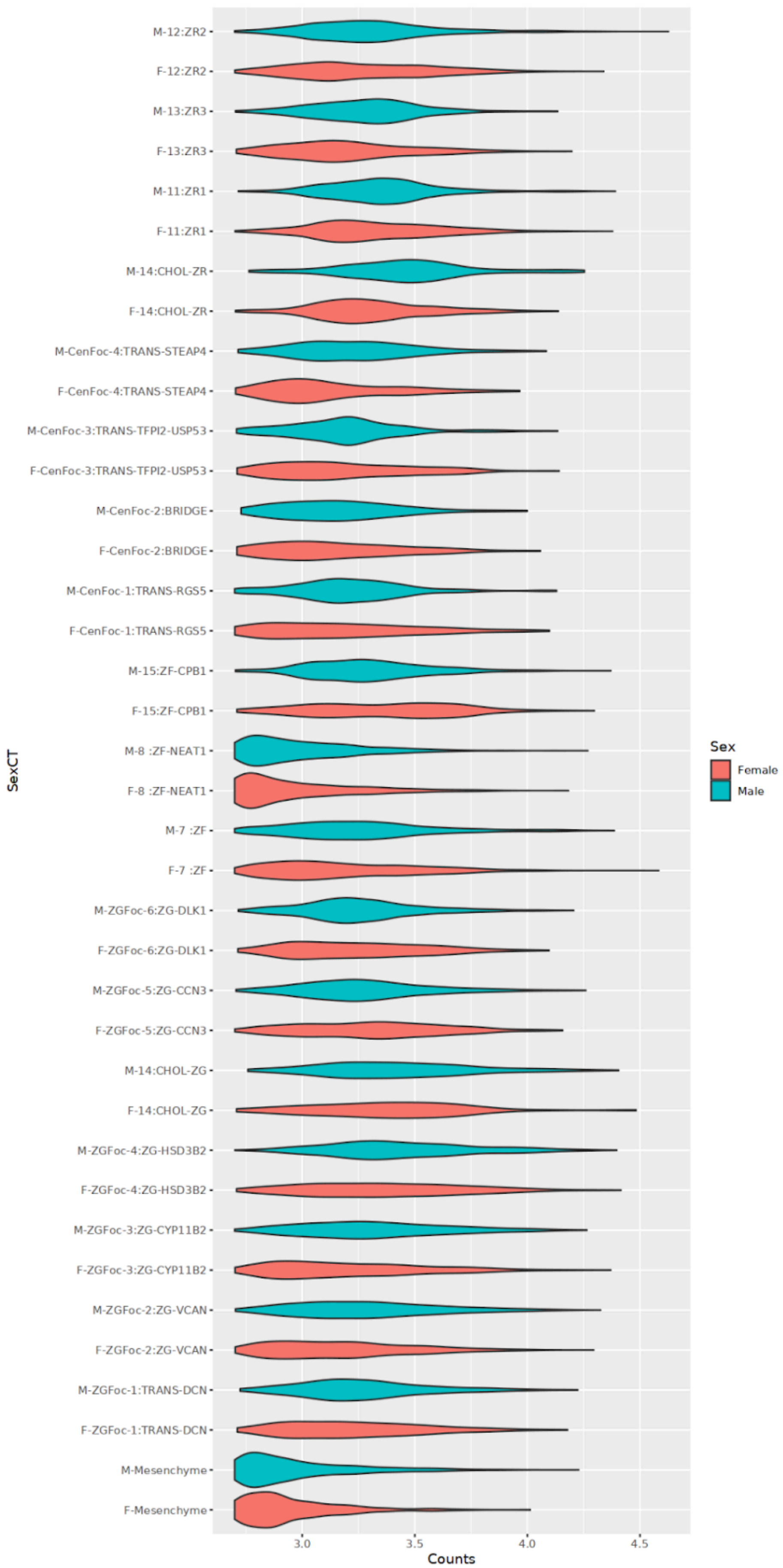

M-12:ZR2
F-12:ZR2
M-13:ZR3
F-13:ZR3
M-11:ZR1
F-11:ZR1
M-14:CHOL-ZR
F-14:CHOL-ZR
M-CenFoc-4:TRANS-STEAP4
F-CenFoc-4:TRANS-STEAP4
M-CenFoc-3:TRANS-TFPI2-USP53
F-CenFoc-3:TRANS-TFPI2-USP53
M-CenFoc-2:BRIDGE
F-CenFoc-2:BRIDGE
M-CenFoc-1:TRANS-RGS5
F-CenFoc-1:TRANS-RGS5
M-15:ZF-CPB1
F-15:ZF-CPB1
M-8 :ZF-NEAT1
F-8 :ZF-NEAT1
M-7 :ZF
F-7 :ZF
M-ZGFoc-6:ZG-DLK1
F-ZGFoc-6:ZG-DLK1
M-ZGFoc-5:ZG-CCN3
F-ZGFoc-5:ZG-CCN3
M-14:CHOL-ZG
F-14:CHOL-ZG
M-ZGFoc-4:ZG-HSD3B2
F-ZGFoc-4:ZG-HSD3B2
M-ZGFoc-3:ZG-CYP11B2
F-ZGFoc-3:ZG-CYP11B2
M-ZGFoc-2:ZG-VCAN
F-ZGFoc-2:ZG-VCAN
M-ZGFoc-1:TRANS-DCN
F-ZGFoc-1:TRANS-DCN
M-Mesenchyme
F-Mesenchyme
SexCT
3.0
3.5
4.0
4.5
Counts
Sex
Female
Male

To provide the additional evidence against detecting androgen suppression of gene expression in our samples, we observed an increase the expression levels of genes related to cholesterol biosynthesis in males (please see Supplementary Fig. 20), in contrast to what would be expected from the work of Morohashi and colleagues. Importantly, we performed our analysis on both pseudobulk DE & GSEA and signature inference from normalized scRNA-seq data, which shouldn't be affected by global expression increase. Since we cannot consistently show a strong effect of androgen on global gene expression, we prefer not to comment on this in the manuscript.

8. Cluster 16 (original clustering) is identified by high levels of H19 expression, but I don't see any mention of this cluster in the text and according to the visium profile the expression seems to be not zone specific. Is this a real cell type?

We thank the reviewer for this comment regarding cluster 16 marked by H19. This cluster emerged in our unbiased analysis, and we also see these H19+ cells in Visium spatial transcriptomics. Therefore, to be fair, we have to keep this cluster.

Overall, this cluster emerged from our unbiased single-cell clustering analysis based on its distinct transcriptomic signature. While H19 is its most prominent marker, cells of this cluster also express low levels of several key steroidogenic genes (CYP11B1, HSD3B2, CYP17A1, CYP21A2), suggesting a mixed steroidogenic identity. The spatial distribution of H19 in our original Visium data (Fig. 2d - shows deconvolution analysis based adrenocortical cells-specific gene expression) was widespread across the cortex, consistent with previous reports indicating that H19, a long non-coding RNA, is upregulated in adrenocortical cells following ACTH stimulation (Liu et al., 1995). However, H19 expression was not zone-specific, and the cluster does not correspond to canonical cortical zones. Further re-analysis of both Visium and single-cell data showed enrichment of this population near the capsule and the zona reticularis–medulla interface (see plot below), but with some presence in other regions as well. Unfortunately, due to technical limitations of the newly generated Stereo-seq data—which does not align non-coding RNAs—we were unable to delineate it further.

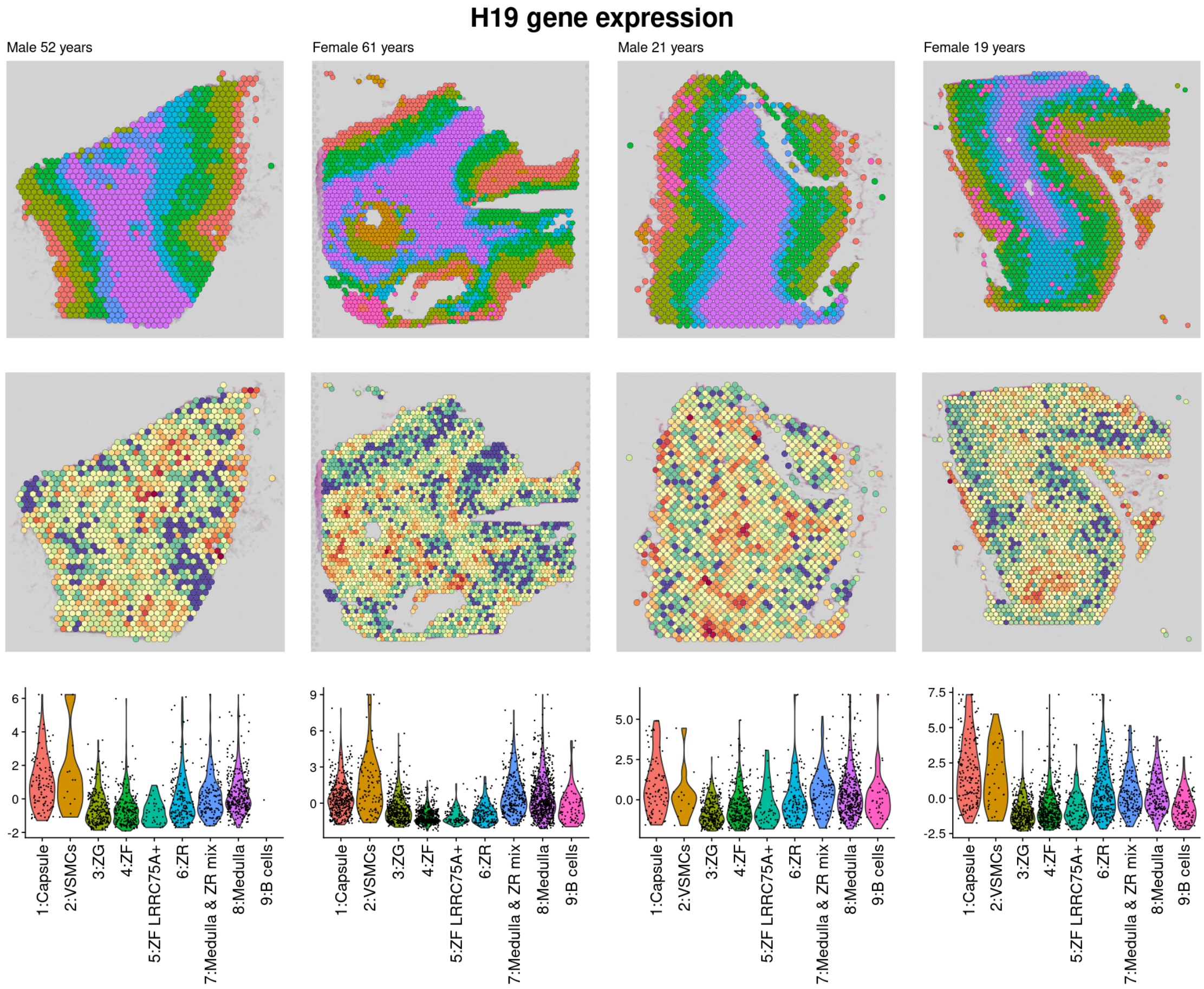

Given its reproducible spatial identification and distinct molecular profile, we have retained cluster 16 in our analysis but are now providing additional clarification in the text, while we emphasize the potential for this cluster to represent a functionally distinct or transitional cell state, although further validation will be required:

"Among the populations intercalated between the distal ZG and ZR, we unbiasedly identified a transcriptionally distinct population (cluster 16, Fig. 2a,c) by high expression of H19, a long non-coding RNA previously shown to be induced in adrenocortical cells upon ACTH stimulation[26]. Cells in cluster 16 also expressed key steroidogenic genes (CYP11B1, CYP17A1, HSD3B2, CYP21A2), suggesting a mixture of steroidogenic identities rather than corresponding to a single adrenal zone. Visium spatial transcriptomics revealed zonation consistent with the single cell data and known markers, with the pan-steroid marker gene CYP21A2 delineating the whole cortex and ZG, ZF and ZR spanning from the outer to inner cortex (Fig. 2a,d). These data also showed the $H19^{high}$ population enriched near the capsule and the ZR–medulla interface, and being scattered across other regions of the cortex (Fig. 2d)."

9. Suppl. Fig.8b: the sexually dimorphic expression appears to differ quite dramatically when comparing scRNA-Seq and visium data. (e.g. enrichment of Cyp11B2 in males for scRNAseq data, but in females for visium data). This needs to be addressed.

We thank the reviewer for pointing out the apparent discrepancy in CYP11B2 expression between the scRNA-seq and Visium spatial transcriptomics datasets. After going in depth into the reasons behind this result, we see that spatial data are very noisy, represented by few samples and cannot be compared directly and quantitatively to better-quality 10X single cell sequencing. To avoid overinterpretation and confusion, we have removed direct comparisons of CYP11B2 and other steroidogenic marker genes between scRNA-seq and spatial data (Supplementary Fig. 21). The Visium dataset comprises only 4 individuals (2 males and 2 females), which limits statistical power to assess sexual dimorphism reliably. In addition, spatial transcriptomics data are subject to several technical limitations, including lower gene detection sensitivity, spatial averaging over multiple cells per spot, and batch effects that can confound expression patterns. Given these caveats, we have revised the figure and text to eliminate this unsupported claim.

Adrenal progenitors:

10. Transitions: The authors claim to have identified 3 different transition states. Additional data are necessary to proof this claim.

We thank the reviewer for requesting additional evidence for these proposed transitions. We generated high-resolution (single cell level) Stereo-seq data from three additional adult adrenal glands + immunohistochemistry/HCR, which allowed spatial validation of three transition trajectories (revised Fig. 4, panels a, i, j, k, Supplementary Fig. 13, 15, 33):

1. From capsular fibroblast cells (CCDC80+/DCN+/FBN1+/FBLN1+ cells) to ZG (Transition 1), seen by gradual upregulation of NR5A1 but retaining DCN expression, consistent with murine models.
2. From VSMCs (ACTA2+/RGS5+) to ZF/ZR through two intermediate states (Transition 2), with gradual downregulation of ACTA2, onset of NR5A1 and STEAP4 expression, retaining RGS5 expression, and further diverging into TFIP2+ or STEAP4 states. Importantly, this transition is

supported not only by these spatial gene gradients but also from the mCA (mosaic clonal analysis) lineage tracing (revised Fig. 4g).

3. From cycling SF1+ cells (Transition 3), potentially contributing to all zones, which we validated not only through Stereoseq on three individuals (revised Supplementary Fig. 33) but also through immunostaining (revised Extended Data Fig. 6).

Although functional lineage tracing is not feasible in humans, these data strongly support the existence of multiple renewal pathways in adult adrenal cortex. Our new results are described in the revised manuscript (please see revised section "*Mechanisms of renewal of steroidogenic cells in the human adult adrenal cortex*") and we provide a disclaimer concerning the fact that our findings are derived from single cell and spatial analysis without functional *in vivo* validations in humans in the Discussion section:

"Importantly, we recognize that our proposed model of adrenocortical renewal, centered around several inferred transitions, is inherently limited by the observational nature of human transcriptomic data including RNA velocity and directional gene expression changes and are not validated by experimental evidence in intact human adrenal tissue. To strengthen the validity of our inferences, we complemented our scRNA-seq analysis with multiple orthogonal approaches, including single-cell resolution spatial transcriptomics and lineage insights gained from a rare clonal mosaic case, which together support the existence of revealed mesenchymal-to-steroidogenic transitions. Even so, we invite the readers to interpret our findings with caution and emphasize that definitive evidence for these lineage relationships will require functional validation, which is currently not feasible in human tissue. We hope this study lays the groundwork for future experimental efforts that can test and refine this model."

11. NR5a1+ cells that undergo cycling should not be considered as true progenitors, if they express other steroidogenic markers and are therefore likely to be actively involved in steroidogenesis. Instead these are differentiated cells that maintain the capacity to undergo cell cycling. **Cycling of NR5A1+ cells has been reported in several studies and the statement in Line 279: "previously unidentified adrenocortical progenitors that lack mesenchymal features" is therefore incorrect.**

We thank the reviewer for pointing out the need to more precisely describe the population of cycling $NR5A1^+$ cells. In response to this comment, we revisited this population in our single-cell and spatial datasets. These $NR5A1^+$ proliferating cells are transcriptionally distinct from other cortical cells beyond the cell cycle signature and consistently cluster together in the UMAP embedding, occupying an intermediate position relative to the three major cortical zones. They are found distributed throughout the cortex in the Stereo-seq data and express specific markers such as EZH2, UBE2T, and STMN1, as well as expected proliferation markers (TOP2A, MKI67), which distinguishes them from fully differentiated steroidogenic cells. The EZH2, UBE2T, and STMN1 genes encode proteins that are involved in epigenetic regulation, protein degradation, and cytoskeletal dynamics, respectively. EZH2 (Enhancer of Zeste Homolog 2) is a histone methyltransferase, specifically a catalytic component of the Polycomb Repressive Complex 2 (PRC2). It tri-methylates lysine 27 on histone H3 (H3K27me3), which leads to transcriptional repression of target genes. EZH2 silences gene expression during development and is crucial for stem cell maintenance, cell fate decisions, and X-chromosome inactivation. Overexpression is seen in many cancers and is considered oncogenic. UBE2T (Ubiquitin-Conjugating Enzyme E2 T) is part of the ubiquitin-proteasome system, functioning as an E2 ubiquitin-conjugating enzyme. UBE2T expression often increases in proliferating or DNA-damaged cells, and overexpression has been implicated in genomic instability and cancer, including lung,

breast, and bladder cancers. STMN1 (Stathmin 1) STMN1 is a microtubule-destabilizing protein. It regulates the dynamics of the microtubule cytoskeleton by promoting depolymerization of tubulin filaments. High STMN1 expression is commonly found in dividing cells and is associated with high proliferation, metastasis, and tumor aggressiveness.

Given these characteristics, we agree that these cells likely represent a specialized, cycling adrenocortical population with the potential for multipotency and not fate-restricted to a specific zone. We have revised the text in the Results section to reflect this more nuanced interpretation and removed the phrase "previously unidentified adrenocortical progenitors that lack mesenchymal features" by *"a population of proliferating NR5A1+/EZH2+ cells that lack mesenchymal features but may represent differentiated cortical cells with cycling capacity as proposed from BrdU labelling in mouse adrenal glands[13]".*

Additionally, we performed immunofluorescent experiments and co-staining for KI67 and SF1, which placed proliferating cells within steroidogenic rosettes (ZG) or columns within the ZF, but showed a discrepancy when it comes to SF1 protein levels, with very rare occurrence of KI67+/SF1+ cells.

"Cycling NR5A1+ cells (referred to as progenitors – PROG) were found distributed throughout the cortex (Supplementary Fig. 33), supported by immunofluorescent detection of KI67 within steroidogenic structures but rare co-detection with SF1 (encoded by NR5A1 gene) in ZG (Extended Data Fig. 6)."

The relevant reference (Chang et al., 2013) has also been added.

12. The authors present evidence for 3 transitions which as far as I can see are primarily based on the arrangement of cells on the UMAP (connection between clusters). While this is indicative it is not formal proof that the cells transit. Moreover, velocity analysis appears to be highly variable between different samples thus questioning the usability of this analysis. If no additional data can be provided, the claims of transition between the different clusters need to be greatly toned down.

We agree that UMAP-based proximity alone is not sufficient proof of cell state transitions. However, directional gene expression changes between connected clusters in the UMAP embedding is evidence of transitions (Supplementary Figs. 26-27). Also, although RNA velocity and pseudotime analysis show some variability in humans, it agrees on the directions of major transitions (Supplementary Figs. 28-32). Finally, we are using the distribution of a mutated clone in the individual with chromosomal alterations (Fig. 4g) to gain insight in which clusters are sharing precursors within the lineage. Still, we understand that in human intact tissue it is impossible to obtain the experimental evidence of transitions (similar to lineage tracing experiments in mice), and hope that the reviewer will understand this major limitation. We also respect the reviewer's opinion on this matter, and we decided to add an explicit disclaimer to the Discussion section (see below) emphasizing that the three proposed transition trajectories are inferred from integrative descriptive evidence and not functionally tested in experiment (as those are human tissues).

"Importantly, we recognize that our proposed model of adrenocortical renewal, centered around several inferred transitions, is inherently limited by the observational nature of human transcriptomic data including RNA velocity and directional gene expression changes and are not validated by experimental evidence in intact human adrenal tissue. To strengthen the validity of our inferences, we complemented our scRNA-seq analysis with multiple orthogonal approaches, including single-cell resolution spatial transcriptomics and

lineage insights gained from a rare clonal mosaic case, which together support the existence of revealed mesenchymal-to-steroidogenic transitions. Even so, we invite the readers to interpret our findings with caution and emphasize that definitive evidence for these lineage relationships will require functional validation, which is currently not feasible in human tissue. “

That said, we also performed additional spatial analysis using our newly generated Stereo-seq data, which provides independent, single-cell resolution support for the proposed trajectories. Specifically, we observed spatial gradients of transitional gene expression (DCN, RGS5, TFPI2, STEAP4, etc.) that correspond to the inferred trajectories in scRNA-seq embeddings (updated Fig. 4i–k, Supplementary Figs. 26-27). This spatial validation adds a layer of confidence to the interpretation of transition states.

Furthermore, we leveraged a rare case of mosaic chromosomal alterations (mCAs) to assess clonal architecture (Fig. 4g, Supplementary Fig. 31). The distribution of clonally aberrant and normal cells across adrenal zones supports a model in which VSMC-derived cells contribute to cortical lineages. While not equivalent to experimental lineage tracing, this analysis provides evidence for the mesenchymal-to-cortical transition.

We have revised our claims throughout the manuscript to reflect this more cautious and evidence-based interpretation (please see below the paragraph introduced in the Discussion section), and we thank the reviewer for prompting this important clarification.

13. Reclustering focusing on progenitor cells: I do not understand why the authors excluded clusters 2 and 13 for their subclustering?

We thank the reviewer for this observation and the opportunity to clarify our subclustering approach. Clusters 2 and 13 were omitted from focused reclustering to improve local resolution of the transition from mesenchyme to steroidogenic cells (specifically involving cluster 10, which included the proliferating SF1+ cells and cells assigned to ZF). In preliminary analyses, we included these broader “parental” clusters in the subclustering, however, their inclusion introduced additional unrelated heterogeneity, which reduced the clarity of the trajectory we aimed to resolve. We would like to point out that the transition from VSMCs (cluster 2) was analyzed extensively in Supplementary Fig. 29.

Our goal was to restrict the analysis to the minimal number of relevant clusters required to capture the transition, thereby enhancing interpretability and visualization. The spatial and transcriptomic positioning of cluster 2 (composed of VSMCs) relative to the transition did not require further refinement, similarly to cluster 13 (composed of ZR cells), which was transcriptionally more distant from the transition in question.

We have now clarified the rationale of cluster subselection in the manuscript to avoid confusion:

“Next, we shifted our focus towards all proliferative populations involved in adult adrenal cortex renewal, also forming transitions from mesenchymal cells (Fig. 4a,b, Supplementary Figs. 26-27). To enhance resolution of transitional states connecting to differentiated ZG/ZF or ZR, we limited subclustering to the minimal set of adjacent clusters composed of NR5A1+ cells relevant to transitions from the proliferative mesenchyme (clusters 7,8,9,10 and 12, Fig. 4a-e).”

14. Line 278/279: The authors describe "previously unidentified adrenocortical progenitors' (SF1+ cells lacking mesenchymal features**). Proliferation of SF1 (NR5A1) positive adrenocortical cells is well established in the field. These cells should however not be considered as progenitors, but rather as differentiated cells that maintain their capacity to undergo cell cycling.**

We thank the reviewer for bringing this fact to our attention. To address this concern, we acknowledged the existence of established cortical dividing cells by adding the relevant reference of the work by Cheng and colleagues to Results and Discussion.

We also edited the manuscript and removed any claims around novelty of the proliferating SF1 population (please also see our response to point 11). In the revised study, we instead place the emphasis on the novel important markers we identified for the SF1+ proliferating cells (such as EZH2, STMN1 among others), as well as the comparative analysis of the SF1+ proliferating cells in fetal versus adult adrenal cortex (revised Fig. 5). Lastly, we validated their existence and spatial distribution using the newly generated spatial data (see Supplementary Fig. 33) and immunofluorescence on adrenal gland sections (revised Extended Data Fig. 6).

Importantly, we are pointing out in the Results and Discussion that these cells may be biased to a specific cortical zone/fate:

"...and 3) a population of proliferating NR5A1(SF1)+/EZH2+ cells that lack mesenchymal features but may represent differentiated cortical cells with cycling capacity as proposed from BrdU labelling in mouse adrenal glands13 (Fig. 4a, Supplementary Figs. 26-27, Extended Data Fig. 5)."

Lines xx-xxx: *"These SF1+/EZH2+ progenitors (now transcriptionally characterized) have a unique gene expression signature beyond proliferation genes, which renders them different from the rest of the surrounding adrenocortical cells. At the same time, being dispersed through the human cortex, they might be potentially semi-differentiated or pre-committed to give rise to the cells of the local cortical zones where they reside. Previously, proliferative cortical progenitors were already established in mouse adrenals via BrdU pulse experiments13. They are likely homologous to SF1+/EZH2+ human cells. Additional future studies with lineage tracing in human organoids are required to resolve this question."*

The proposed transition of ACTA2+/RGS5+/MYH11+ VSMCs to the zF and ZR is somewhat provocative as it contradicts studies in mice. I do not dispute the fact that cell renewal might be different in the two species, but the authors need to provide better evidence that there is a real transition into steroidogenic cells.

We agree that the proposed transition of $ACTA2^{+}/RGS5^{+}/MYH11^{+}$ vascular smooth muscle cells (VSMCs) into steroidogenic cells - particularly of the ZF and ZR – diverges from existing models of adrenocortical renewal derived from murine studies, which primarily support capsular progenitor-derived centripetal differentiation. To support this possibility, we present several independent lines of evidence from our results:

1. Transcriptomic Continuity: our scRNA-seq data show a gradual shift in gene expression from $ACTA2^{+}/RGS5^{+}$ VSMC-like cells to intermediate states (e.g., $RGS5^{+}/TFPI2^{+}/STEAP4^{+}$) and onward to steroidogenic clusters expressing STAR and CYP11B1. This continuity is visible in UMAP space and supported by marker gene co-expression (Supplementary Figs. 26-27, Supplementary Figs. 29-30).

2. Spatial Validation: in our newly generated Stereo-seq data, these intermediate states were spatially mapped along the cortex, with RGS5⁺/STEAP4⁺ cells enriched in the ZR, decreasing in the ZF, and largely absent in the ZG (Fig. 4i–k). This spatial gradient is consistent with a differentiation trajectory along the centripetal axis, albeit distinct from the capsular path.
3. Clonal Evidence: a unique case of mosaic chromosomal alterations (mCAs) offered lineage-like information (Fig. 4g, Supplementary Fig. 31). The aberrant clone, lacking representation in the ZR but present in ZG and ZF, supports a model where the ZR arises from a lineage separate from the canonical capsular-derived cells, consistent with a VSMC-derived route contributing to ZR.

We have revised the manuscript to clarify that this proposed VSMC-to-cortex transition is human-specific (we refer to this as a hypothesized transition) and emphasize the need for functional studies to validate this model in humans (lines 330-339):

"Furthermore, the transition from VSMCs to ZF/ZR through the transient RGS5 state was enriched in ZR, decreasing in ZF (Fig. 4i,j, Supplementary Fig. 15). These findings suggest a new model of human adrenocortical turnover that goes beyond the classical centripetal model from ZG to ZF and subsequently ZR (Fig. 4k). Even though we lack data on the precise role of these populations in the adrenal cortex and functional validations in iPSC-derived models will be necessary, these observations open many directions for further research."

15. Concerning CCDC80: Line 284: According to the literature (https://doi.org/10.1210/me.2014-1060), Ccdc80 is a WNT dependent gene that is strongly expressed in zG cells, at least in the mouse. Is the expression in humans different? Line 287: Why does the expression of the "CCDC80+ gene, nearly exclusive to RSPO3+ fibroblasts, confirm that specifically capsular fibroblasts are involved in this transition" ?

Indeed, in human adrenal cortex, we find CCDC80 expressed in higher levels in the capsule and slightly less in ZG, as confirmed by RNAscope in situ hybridization (please see figure below). We rephrased the relevant passage in the revised manuscript:

"…cells of cluster 1 (capsule, CCDC80+/FBN1+/FBLN1+/DCN+/NR5A1- cells mapped through Visium and subsequently Stereo-seq to the outer surface of the gland (Fig. 4h).."

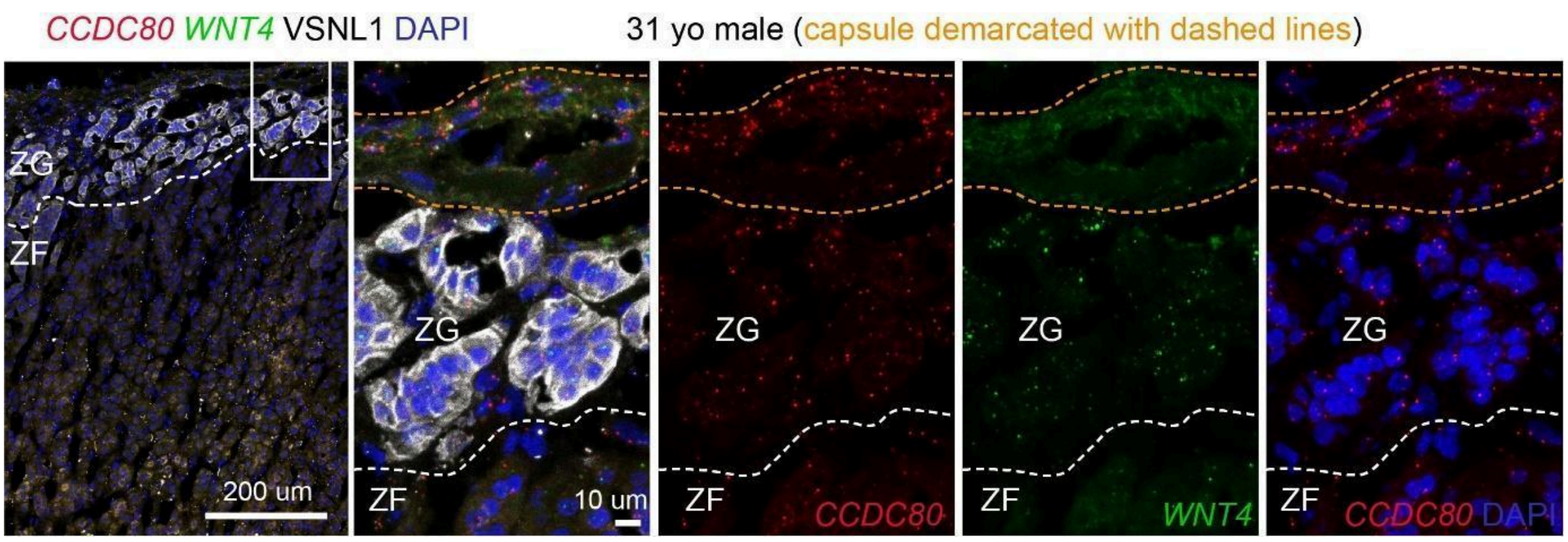

16. Line 700: "Finally, we identified unique transitions from-mesenchyme-to-cortex in adult adrenal as compared to fetal" . Some of these differences may stem from artefacts due to data integration

We thank the reviewer for raising this question. Notably, our comparison of the mesenchymal populations revealed key differences in marker gene expression levels or percentage of cells were clearly observed (revised Fig. 5e), as well as extensive heterogeneity within the fetal mesenchyme, reflected in four subclusters rather than the main two of the adult (VSMCs and capsular fibroblasts), and the predicted WT1high cluster that is evident in the fetal and only represented by very rare cells in the adult (Fig. 4e).

During the revision we re-analyzed the combined fetal and adult dataset (revised Fig. 5, Extended Data Fig. 8) explicitly comparing the gene expression of key steroidogenic populations making up the two. During re-analysis, we subselected ZG/ZF of adult with definitive zone (DZ) (both glucocorticoid-producing) and ZR with fetal zone (androgen-producing). While the integration of cells derived from developing and adult adrenal cortex is validated as smooth intermingling of the single cell transcriptomes to a great extent on the combined embedding, we observe adult-specific clusters of cells which persist when plotting key marker genes of populations (Extended Data Fig. 8g-i, k-m). Specifically, the adult ZG-DCN population is unique to the adult (Extended Data Fig. 8j), and in our inferred model it gives rise to ZG cells. Similarly, a ZR subcluster appeared unique to the adult cortex, characterized by expression of CD74, HLA-DRA, NUPR1 and SLC40A1, pointing to a pro-inflammatory phenotype (Extended Data Fig. 8l).

We further detected the CPB1+ potentially transitional cluster (Extended Data Fig. 8e) and run a more thorough analysis of the SF1+ proliferating cells and their transcriptome (revised Fig. 5 d-g)

Thus, we are inclined to answer that our observations seem to be independent of artefacts and would like to point out that collectively our observations would be very informative to build upon existing iPSC-derived adrenocortical organoid protocols to model the adult adrenal cortex in vitro.

17. Figure 4b: This graph is very difficult to understand, without additional explanation. What characteristics were used to define fetal progenitors? The authors suggest that the transitions from progenitors to steroidogenic cells are profoundly different between developmental and adult samples. However, the overlap of data sets after data integration (shown in suppl. Fig 24) does not seem to be particularly strong. What is the evidence that the data normalization of the different data sets worked well? This is important for drawing firm conclusions about differences between fetal and adult adrenal biology.

To address the reviewer's concerns, we revised Fig. 4 (now Fig. 5) to improve clarity and now provide a more focused analysis. Specifically, we limited the comparison to a well-defined subset of $SF1^{+}$ cells enriched for mitotic markers (*MKI67*, *PCNA*, and others; see Supplementary Fig. 27b). These proliferating progenitor cells were identifiable in both fetal and adult single-cell transcriptomic datasets and correspond to the red cluster in Fig. 5a.

In the revised analysis (Fig. 5d–g), we focused on the mentioned fetal and adult progenitor subsets to examine their transcriptional similarity more directly. The integration revealed that fetal and adult progenitors cluster closely together (Fig. 5c), suggesting a shared core identity. To further validate this, we performed differential gene expression analysis, which showed a set of 47 shared genes (excluding cell cycle-related genes), highlighting a robust progenitor signature across developmental stages. Despite this overall similarity, we observed notable differences in specific transcriptional regulators in these subsets, so they are not entirely identical. Fetal progenitors showed higher expression of *NR5A1*, the master regulator of steroidogenic cell fate, while the adult progenitors exhibited relatively elevated expression of *EZH2*, a chromatin remodeling factor. These consistent

distinctions may reflect developmental stage-specific regulatory programs and could inform strategies for *in vitro* reprogramming.

Regarding integration quality, we employed batch correction methods for single-cell data (described in the Methods section) and confirmed the success of dataset alignment through both visual inspection (Supplementary Fig. 37) and preservation of known biological relationships (e.g., progenitor proximity across datasets in reduced dimensions). While complete overlap is not expected given biological differences between fetal and adult tissues, the observed clustering and gene expression coherence support the validity of our integrative approach.

We have revised the figure and accompanying text to better guide the reader through the analysis and clarify our conclusions (please see revised section *"Comparison of adult and fetal human adrenocortical heterogeneity and transitions"*).

18. Extended data Fig7a and Suppl. Fig.24 g: I am not sure I interpret this map correctly, but it seems to me that mesenchymal and steroidogenic cells are not connected. If this is the case, how can the authors draw conclusion about progenitor cell -> steroidogenic cell transitions during development?

In this analysis, we integrated only later timepoints of fetal adult cortex (from week 7 of gestation onwards – around CS16-17). According to the literature (Cheng et al, Sci Adv 2022), we do not necessarily expect mesenchyme-to-cortex transition to take place after CS12-13 stages, since the WT1+;KRT19+;NR5A1+;GATA4- adrenergic coelomic epithelium that transits to adrenogenic mesenchyme becomes already fate restricted. Thus, at the stages we investigated here, mesenchymal cells probably do not give rise to adrenocortical cells, which later start to increase their numbers through proliferation (all consistent with the following papers in human in vivo and in vitro systems: Spencer et al., JCEM 1999, Cheng et al., Sci Adv 2022, Sakata et al., Dev Cell 2022).

In the revised manuscript, we mention this in the Results:

"Next, adult renewal appears to involve adrenal capsule-derived progenitors, contrasting with the fetal cortex at 6-7 weeks post-conception and onwards -a time during which a direct mesenchyme-to-steroidogenic transition is not evident, and a bridging population between the two is not observed in data (Extended data Fig. 7a). This is in agreement with studies recapitulating human fetal cortex development and the subsequent expansion of the fetal cortex through proliferation, rather than mesenchymal-to-steroidogenic transitions[5,39,40]*. "*

An alternative could be that mesenchyme-to-cortex transitions we described in the adult data take place in considered fetal stages, but with dramatically lower frequency and representation. In such a case, transitions could be absent ("broken") on the embedding of fetal-only samples, but appear in the integration of fetal and adult samples, being supported by adult data. However, we didn't find any evidence of fetal mesenchyme-to-cortex transitions (from week 7 of gestation onwards) even being integrated with adults (Fig. 5c, Extended Data Fig. 8ab). This means that even if a rare fetal mesenchyme-to-cortex transition takes place (not captured by our fetal-only analysis), we can confidently say that it is different from described adult transitions.

Mouse adult data:
19. The proposed transition of cells from the mesenchyme to both zG and zF is interesting as it would

suggest that cells are able to be directly recruited to the zF (without prior zG differentiation). This would be supporting data by Freedman et al., 2013 (10.1016/j.devcel.2013.07.016). This should be discussed.

We thank the reviewer for this insightful comment and highlighting the relevance of the Freedman et al. (2013) study. We agree that our observation of a mesenchymal transition pathway contributing directly to ZF, bypassing ZG, is consistent with the ZG-independent differentiation mechanism previously demonstrated in mice. As suggested, we have revised the manuscript to incorporate this point and cited the Freedman et al. study (Dev. Cell 2013) as experimental evidence supporting this model. Specifically, we now highlight a subset of $Wt1^{+}/Gata4^{+}$ mesenchymal cells contributing directly to the ZF in our dataset (Supplementary Fig. 55), paralleling the mouse data in which ZF differentiation occurred in the absence of ZG-derived cells.
This addition strengthens the evolutionary relevance of our findings and supports the idea that multiple, parallel progenitor-to-cortex trajectories may exist in both species.

"Previous studies also revealed $Wt1^{+}/Gata4^{+}$ mesenchymal stem cells within the murine capsule giving rise to ZF cells[15,48], bypassing the ZG, through a transient state unveiled in our data (cluster 5:Transition-Wt1) (Supplementary Fig. 55a-d, Fig. 7k), for which we uncovered novel marker genes (Serpina5, Folr1, Amhr2, Bpifb4 and others) (Supplementary Fig. 55)."

"The second stem cell source in mice, marked by $Wt1^{+}/Gata4^{+}$ expression, has been shown to contribute directly to the ZF. Freedman et al. demonstrated this pathway experimentally by showing intact ZF formation even upon targeted deletion of Nr5a1 (gene coding for SF1) in ZG cells[48]. Our human data suggest a comparable trajectory, indicating that ZG-independent ZF formation may be conserved across species."

20. Grabek et al. (2019), suggested recruitment to be higher in females than in males. Are female cells 'enriched' in transitional clusters? According to extended Fig10 transitional clusters 4 & 5 appear to be enriched in males – which is the opposite of what I would expect).

We thank the reviewer for this question. Formally, none of the clusters in this analysis showed statistically significant compositional differences between males and females (Supplementary Fig. 49c). The mesenchymal cluster 1: Mesenchyme-Sparcl1 (*Gli1+/Pthc1+* cells, source of transitions) was overrepresented in females before p-values adjustment on multiple comparisons (Supplementary Fig. 49c), but this still can not be considered as a significant difference. Cluster-free analysis also didn't give any significant compositional differences in renewal-related clusters (Supplementary Fig. 49f). Furthermore, during the revision, we analyzed the numbers of proliferating SF1+ progenitors (cluster 4) cells in the single cell transcriptomic data sets and newly generated Visium HD data sets and found no significant differences between male and female mice (see revised Fig. 8b).

However, we found that although the proliferating SF1+ progenitor cell numbers do not differ, female cells may proliferate more actively, as shown by their higher enrichment in genes related to proliferation (i.e. cell cycle and DNA replication KEGG pathways) as compared to the situation in males (upper Fig. 8c, Supplementary Fig. 49h). This finding was also supported by VisiumHD spatial transcriptomic analysis (lower Fig. 8c).

We are referring to these findings in the revised section of Results:

"Interestingly, analysis of numbers of proliferating NR5A1+ progenitor cells did not reveal a significant difference between males and females in both mouse and human, in single cell or spatial data (Fig. 8b). Despite this fact, further exploration of mouse sexual dimorphism led to the observation of enrichment of cell cycle and DNA replication pathways in female-derived proliferating NR5A1+ progenitor cells (cluster 4:PROG) (Supplementary Fig. 49g-i, Fig. 8c). This finding was supported by Visium HD spatial transcriptomics data, whereby analysis of mitotic signature in mouse male versus female cortex as a function of a) cellular subtype and b) zone of enrichment, confirmed more extensive proliferation within the progenitors in female mice (Fig. 8c, Supplementary Fig. 58). Thus, the previously described increased rate of adrenal cortex turnover in female mice[14] likely relies not on a larger number of progenitors but on an increased rate of their proliferation. In a similar analysis comparing proliferation activity between human male and female progenitors, statistical significance was not achieved (Fig. 8b,c)."

21. Supplementary data Figure 28: The expression profile does not well reflect well the known zonated expression of genes. For example Axin2 (b-catenin signaling responsive gene) was shown to be highly expressed in zG but only at low levels in the zF. Is this a problem of representing intensity on the UMAPs?

We thank the reviewer for highlighting this issue. Upon re-evaluation, we identified that the apparent discrepancy in *AXIN2* expression was primarily due to suboptimal color scaling in the original UMAP plots. In the revised version of Supplementary Fig. 54, we have adjusted the color palette (from yellow to red, representing low to high expression) to better reflect true expression intensity across clusters.

Additionally, to provide a clearer overview of canonical zonated marker genes - including *AXIN2* and others - we now include a new supplementary figure (revised Supplementary Fig. 47) showing the expression of well-established ZG and ZF markers on both single-cell UMAPs and spatial transcriptomics data (VisiumHD), separately for male and female samples. This update confirms expected patterns of zonation and supports the reliability of our spatial and single-cell data integration.

22. The discussion is rather long, at times highly speculative and should be shortened.

We agree with this comment and we made substantial changes during the revision to reflect future perspectives and recap our main findings, along with shortening the text down to a bit over 3 pages from 5 pages of the original submission.

Given the amount of new data and requests from other referees, we hope this shortening will be satisfactory.

Minor points:
Line 67: the coelomic epithelium is lateral plate and not intermediate mesoderm derived

We are thankful for pointing out this mistake, which we corrected in the revised version of the manuscript (line 76).

A previous manuscript has suggested that in mice nestin+ cells can contribute to the steroidogenic lineage. Is there any evidence from the single cell data for this transition (in both mice and human)?

During the revision, we took another look at the Nestin expression and found it expressed by few tightly subclustered mesenchymal cells both in human and mouse (cluster 3) (revised Fig. 7l). It might be possible that this population is kept in small numbers under normal conditions, and is activated upon external stressors. Unfortunately, we cannot address this directly in our data, because the cell numbers of these Nestin+ cells are low, and the entire mesenchymal cluster is entering the transition to adrenal cortex, most likely engaging these cells. In sum, our data do not contradict the idea that Nestin+ cells can contribute to steroidogenic lineage, and they also do not provide a direct support beyond the fact that these mesenchymal Nestin+ cells are a part of bigger cluster entering such a transition. We decided not to engage into discussing this point because of this and the lack of space provided by the journal (we are beyond the word limit).

Extended figure 6: LGR4 often substitutes for LGR5: please provide data for the expression of this gene

We thank the reviewer for pointing out the redundancy between the two genes. We show their expression both in the scRNA-seq data set and spatial of our mouse data sets in Supplementary Fig. 47 (bottom). We observe negligible expression of Lgr4, showing that at least in the mouse adrenal gland its expression is not noteworthy.

Fig 3f: Correct "Strergidogenesis" -> 'Steroidogenesis"

This is corrected. Again, we want to thank the reviewer for meticulously going through the manuscript and figures. We addressed this mistake in all mentions in the revised version of the manuscript.

References

Baker, J. E., Plaska, S. W., Qin, Z., Liu, C.-J., Rege, J., Rainey, W. E., & Udager, A. M. (2021). Targeted RNA sequencing of adrenal zones using immunohistochemistry-guided capture of formalin-fixed paraffin-embedded tissue. *Molecular and Cellular Endocrinology*, *530*, 111296. https://doi.org/10.1016/j.mce.2021.111296

Chang, S.-P., Morrison, H. D., Nilsson, F., Kenyon, C. J., West, J. D., & Morley, S. D. (2013). Cell Proliferation, Movement and Differentiation during Maintenance of the Adult Mouse Adrenal Cortex. *PLOS ONE*, *8*(12), e81865. https://doi.org/10.1371/journal.pone.0081865

Cheng, K., Seita, Y., Moriwaki, T., Noshiro, K., Sakata, Y., Hwang, Y. S., Torigoe, T., Saitou, M., Tsuchiya, H., Iwatani, C., Hosaka, M., Ohkouchi, T., Watari, H., Umazume, T., & Sasaki, K. (2022). The developmental origin and the specification of the adrenal cortex in humans and cynomolgus monkeys. *Science Advances*, *8*(16), eabn8485. https://doi.org/10.1126/sciadv.abn8485

Freedman, B. D., Kempna, P. B., Carlone, D. L., Shah, M., Guagliardo, N. A., Barrett, P. Q., Gomez-Sanchez, C. E., Majzoub, J. A., & Breault, D. T. (2013). Adrenocortical zonation results from lineage conversion of differentiated zona glomerulosa cells. *Developmental Cell*, *26*(6), 666–673. https://doi.org/10.1016/j.devcel.2013.07.016

Gao, T., Kastriti, M. E., Ljungström, V., Heinzel, A., Tischler, A. S., Oberbauer, R., Loh, P.-R., Adameyko, I., Park, P. J., & Kharchenko, P. V. (2023). A pan-tissue survey of mosaic chromosomal alterations in 948 individuals. *Nature Genetics*, *55*(11), 1901–1911. https://doi.org/10.1038/s41588-023-01537-1

Han, W., Gao, S., Barrett, D., Ahmed, M., Han, D., Macoska, J. A., He, H. H., & Cai, C. (2018). Reactivation of androgen receptor-regulated lipid biosynthesis drives the progression of castration-resistant prostate cancer. *Oncogene*, *37*(6), 710–721. https://doi.org/10.1038/onc.2017.385

Huang, C.-C. J., Miyagawa, S., Matsumaru, D., Parker, K. L., & Yao, H. H.-C. (2010). Progenitor cell expansion and organ size of mouse adrenal is regulated by sonic hedgehog. *Endocrinology*, *151*(3), 1119–1128. https://doi.org/10.1210/en.2009-0814

Kameneva, P., Artemov, A. V., Kastriti, M. E., Faure, L., Olsen, T. K., Otte, J., Erickson, A., Semsch, B., Andersson, E. R., Ratz, M., Frisén, J., Tischler, A. S., de Krijger, R. R., Bouderlique, T., Akkuratova, N., Vorontsova, M., Gusev, O., Fried, K., Sundström, E., … Adameyko, I. (2021). Single-cell transcriptomics of human embryos identifies multiple sympathoblast lineages with potential implications for neuroblastoma origin. *Nature Genetics*, *53*(5), 694–706. https://doi.org/10.1038/s41588-021-00818-x

King, P., Paul, A., & Laufer, E. (2009). Shh signaling regulates adrenocortical development and identifies progenitors of steroidogenic lineages. *Proceedings of the National Academy of Sciences*, *106*(50), 21185–21190. https://doi.org/10.1073/pnas.0909471106

Krycer, J. R., & Brown, A. J. (2011). Cross-talk between the Androgen Receptor and the Liver X Receptor: IMPLICATIONS FOR CHOLESTEROL HOMEOSTASIS. *Journal of Biological Chemistry*, *286*(23), 20637–20647. https://doi.org/10.1074/jbc.M111.227082

Liu, J., Kahri, A. I., Heikkilä, P., Ilvesmäki, V., & Voutilainen, R. (1995). H19 and insulin-like growth factor-II gene expression in adrenal tumors and cultured adrenal cells. *The Journal of Clinical Endocrinology & Metabolism*, *80*(2), 492–496. https://doi.org/10.1210/jcem.80.2.7531713

Lonsdale, J., Thomas, J., Salvatore, M., Phillips, R., Lo, E., Shad, S., Hasz, R., Walters, G., Garcia, F., Young, N., Foster, B., Moser, M., Karasik, E., Gillard, B., Ramsey, K., Sullivan, S., Bridge, J., Magazine, H., Syron, J., … Moore, H. F. (2013). The Genotype-Tissue Expression (GTEx) project. *Nature Genetics*, *45*(6), 580–585. https://doi.org/10.1038/ng.2653

Lyraki, R., & Schedl, A. (2021). Adrenal cortex renewal in health and disease. *Nature Reviews Endocrinology*, *17*(7), Article 7. https://doi.org/10.1038/s41574-021-00491-4

Mayama, M., Whelan, E. C., Sato, T., Stouffer, D. G., Leu, A. N., Strauss, J. F., Auchus, R. J., & Sasaki, K. (2025). *Reconstitution of adrenocortical functional zonation from human pluripotent stem cells* (p. 2025.05.24.655925). bioRxiv. https://doi.org/10.1101/2025.05.24.655925

Sakata, Y., Cheng, K., Mayama, M., Seita, Y., Detlefsen, A. J., Mesaros, C. A., Penning, T. M., Shishikura, K., Yang, W., Auchus, R. J., Strauss, J. F., & Sasaki, K. (2022). Reconstitution of human adrenocortical specification and steroidogenesis using induced pluripotent stem cells. *Developmental Cell*, *57*(22), 2566-2583.e8. https://doi.org/10.1016/j.devcel.2022.10.010

Yuefeng, Y., Zhiqi, L., Yi, C., Keyu, Z., Heng, W., Yuying, W., Ningjian, W., Yuetian, Y., Xinjie, G., Yihao, Z., Yingli, L., & Fangzhen, X. (2022). Testosterone Deficiency Promotes Hypercholesteremia and Attenuates Cholesterol Liver Uptake via AR/PCSK9/LDLR Pathways. *International Journal of Endocrinology*, *2022*(1), 7989751. https://doi.org/10.1155/2022/7989751

Response to Reviewers' Comments:

Reviewer #1 (Remarks to the Author):

The revision addresses most of my major concerns with new data and a clearer narrative. The Results are now organized around four explicit questions, which greatly improves coherence, and the fetal analysis is elevated into a dedicated, data rich section (Fig. 5) instead of a brief mention in the Discussion. Orthogonal validation has been added throughout: high resolution spatial datasets (Stereo seq for human, Visium HD for mouse) anchor the single cell subclusters in tissue and help adjudicate transitional states (Figs. 2-4, 7), while RNAscope/IHC support key ZG markers (e.g., VCAN, CCN3, DLK1) and the ZG HSD3B2^high state (Fig. 3; Extended Data referenced). The renewal section is rewritten with three concrete sources (capsule→ZG via DCN^+, VSMC→ZF/ZR, and dispersed NR5A1^+/EZH2^+ progenitors) and linked to spatial evidence and a schematic (Fig. 4i-k). The interspecies comparison is now explicit (co integrated UMAP plus spatial alignment) and carefully distinguishes conserved aldosterone producing cells from human or mouse specific programs (Figs. 6-7). Sex effects are handled more systematically: no significant male-female difference in human progenitor counts/cycling, contrasted with stronger proliferative signatures in female mouse progenitors, corroborated spatially (Fig. 8b-c). Finally, the color/cluster correspondence in focused embeddings is clarified (Fig. 4 caption), the transitional labeling for prior "clusters 9-10" is justified with spatial and clonal context, and data/code access is now provided.
That said, several items remain only partially resolved.

We want to thank the reviewer for recognizing our efforts to improve the manuscript during the revision and the constructive comments they provided. We are providing a point-by-point response below. Please note that new edits in the manuscript are marked in yellow.

First, "heterogeneity" claims are still largely qualitative; consider adding a formal cross species heterogeneity metric (e.g., diversity/entropy or differential gene program dispersion) to substantiate statements beyond descriptive clustering (Figs. 2-3, 6-7).

We want to thank the reviewer for pointing out this issue. During the revision, we introduced the suggested metric (revised Supplementary Fig. 52g) as a plot with formal cross-species comparison of ZG and ZG-to-ZF (or early ZF) subclusters. Specifically, we selected a common set of variable genes (each was variable in at least two samples) and estimated ROGUE entropy metric[1] in each sample containing at least 100 cells. Our analysis showed entropy in human samples to be significantly increased ($p = 0.048$). A short description of this result has been added to the Results section:

“Lastly, as a metric of cortical cellular subpopulations, we performed a quantitative analysis of interspecies heterogeneity of ZG and ZG-to-ZF clusters, which revealed increased heterogeneity in human versus mouse adrenal cortex (Supplementary Fig. 52g), which may reflect numerous transitional states and scaling of the gland.Additionally, we reformulated parts of the manuscript that were centered around cellular heterogeneity in human and we interpret the observed trascriptomically distinct cells as cell states that occur with age (see “Steroidogenic cell states and age-related shifts in the human adrenal cortex”).”

Second, the Methods document AUC ranked markers (FindAllMarkers, "roc") but do not state the explicit AUC threshold used to accept subclusters; if an AUC > 0.8 rule guided interpretation,

please codify it and, ideally, add a brief table listing each retained subcluster and its qualifying marker(s) (Methods pp. 27-31).

Specifically, sometimes re-clustering leads to a split of a single cluster into two and only one of them has a very specific marker. For example, original 6: ZG-CYP11B2 was re-clustered into ZG-VCAN ($VCAN^{high}$/CYP11B2-) and ZG-CYP11B2 (VCAN+/CYP11B2+). For the cluster ZG-CYP11B2 gene CYP11B2 expression is absolutely exclusive (AUC > 0.95, absent in any other cluster including ZG-VCAN), while ZG-VCAN turned out to be mostly negatively defined with the best markers HOPX (AUC = 0.73) and VCAN (AUC = 0.695). We clarified the lower AUC threshold in the Methods section:

"For the focused clusters of human ZG & ZF all clusters had at least one marker gene with AUC > 0.7, which guided their following interpretation."

To avoid any misinterpretations we now provide Supplementary tables for all key embeddings with marker genes of all subclusters and their AUC values - available on gitlab (file Markers of clusters of key embeddings.xlsx) and figshare.

Third, lineage inferences in human should be phrased more cautiously: RNA velocity is explicitly "variable across samples," and the somatic CNV evidence derives from a single donor (Fig. 4g; pp. 8-9); I recommend "supports" rather than "validates," and per sample velocity maps in Supplement.

We thank the reviewer for pointing out that these claims should be softened and that the inferred nature of the lineage dynamics should be pointed out. The relevant change in wording can be found in the revised Results:

"The pattern of mCAs in this sample, composed of mostly genetically normal ZR cells (just 3 cells within ZR are aberrant) and clonally-linked aberrant VSMCs, ZG and ZF cells, supports the inferred transition from VSMCs through TFPI2+/GNG11+ cells differentiating towards cells of the ZF and ZG, albeit pending biological validation."

Individual sample velocity can be found in revised Supplementary Figure 52, on the eleven human adrenal cortex samples from which we have obtained single cell transcriptomic data.

Fourth, the ZG HSD3B2^high "direct cortisol" interpretation is compelling transcriptionally/spatially but remains inferred (p. 7; Fig. 3); please soften to "transcriptionally consistent with," pending functional assays.

We thank the reviewer for this suggestion. During the revision, we performed additional experimental stainings, improving upon the previous approach, using immunodetection against HSD3B2 and a validated monoclonal antibody against CYP11B2 combined with in situ for CYP11B1 (revised Extended Data Fig 4 and Supplementary Fig 11). These new validations provide a more direct assessment of the enzyme content of cells and uncovered the presence of these cells in ZG of older (older than 40 years) donors.

However, this finding is yet to directly prove cortisol production (experimentally) and we revised the text as follows:

"These cells lack CYP11B2 required for aldosterone synthesis despite expressing CYP17A1+/CYP11B1+ (Fig. 3b), suggesting direct cortisol production within the ZG, as supported by a previous proteomic study[33]. This surprising finding of classical ZF marker genes CYP17A1 and CYP11B1 in ZG has been reported to reflect the effect of aging on the human adrenal gland.

Since hormone measurement in our fixed samples was not possible, we performed combined RNAscope in situ hybridization and immunofluorescence, confirming spatial co-expression of HSD3B2 and CYP21A2 and HSD3B2+/CYP11B1+/CYP11B2- cells within classical rosettes within of ZG exclusively in older donors (Extended Data Fig. 4, Supplementary Fig. 11). These findings are consistent with the spatial transcriptional data but functional validations for cortisol production from ZG are pending."

Finally, the "cholesterol balance" is a gene set-based residual model (Fig. 3g-h); renaming it "inferred cholesterol balance activity" would avoid implying flux measurements and adding per donor age (and rare WT1^+ cell) quantification would pre empt concerns about confounding and detection bias (Fig. 4e-f; Fig. 8b).

We are grateful for the insightful suggestions. We adapted the wording around the cholesterol metabolism analysis accordingly in revised Figure 3e, g, j and corresponding parts all throughout the text.

Furthermore, compositional contributions along with donor sex and age are available on revised Supplementary Figs. 1-3, while total number of WT1+ cells (only 4 cells in the total data set, reflecting their rare nature in the adult adrenal cortex, as opposed to the fetal gland) are now shown in revised Fig. 4e-f.

Reviewer #1 (Remarks on code availability):

README need to be improved with better guidance on how to reproduce the result.

We thank the reviewer for this important comment. To accommodate it, we now included the README link on a table of all key R-objects (as .rds files) (Supplementary Table 1) with codification of relevant notebooks and analysis, which can guide any attempt to reproduce our results. Also, all R-objects (.rds files) are available upon the request.

Reviewer #2 (Remarks to the Author):

I do not have further comments.

Reviewer #3 (Remarks to the Author):

The sheer amount of data in this manuscript is impressive and the authors have included a significant amount of additional data including high resolution spatial analysis. Saying this, I still have a number of problems with the manuscript, as I feel that the authors often overinterpret bioinformatic data. I realise that they included a statement (caution) at the end of their study to highlight the fact that many of their interpretations are primarily based on bioinformatic analysis. However, publishing models that are not fully corroborated may lead to the establishment of wrong dogmas, especially when published in a prestigious journal such as Nature Genetics.

Given the large amount of data (8 figures, 10 extended figures, 59 supplementary figures), verifying every claim in this manuscript is almost impossible for a reviewer. Below you will find a list of some of my concerns.

We want to thank the reviewer for recognizing our efforts and expressing their concerns around data interpretation. Please see below our response and approach we took to clarify all concerns. Changes in the manuscript are marked in yellow.

Cellular complexity:

One of the key claims of the manuscript is a very high complexity of the adrenal cortex with - for example - the presence of at least 6 different zG clusters as defined by single cell seq analysis. Depending on settings, single cell analysis can yield a very high number of clusters that represent relatively subtle alterations between cells of the same type rather than truly independent cell types.

We thank the reviewer for raising this concern. We agree on the fact that some of the observed clusters correspond to subtle transcriptomic alterations as opposed to more major differences (i.e. aldosterone- versus cortisol-producing cells, morphology etc). Nevertheless, those clusters emerged during unbiased analysis and are characterized by specific marker genes, which can not be achieved by technical high resolution re-clustering. In addition, several of those markers, like HSD3B2, CCN3 and DLK1, are established in the adrenal gland research field and closely related to adrenal biology. Thus, marker genes that arose in this analysis are not due to technical aspects of cell dissociation.

Overall, we do not see any reason to exclude any of the described clusters from the analysis or interpretation and we hope that the provided results, including high-resolution spatial mapping of novel cell states, will be helpful for future research.

However, we understand that the heterogeneity claim needed further experimental support and rephrasing. During the second round of revision, we made an effort to further validate our observations on cell states with robust molecular differences within the ZG, by performing additional stainings for VCAN, DLK1, CCN3, HSD3B2, CYP11B1 and CYP11B2 (revised Extended Data Fig 3,4, Supplementary Fig 10, 11). These revealed CCN3 immunoreactivity in high levels in ZG with a gradient in ZF, DLK1 immunoreactivity (co-expressed with CCN3 in some cells in ZG and ZF) mainly in ZF, and marker gene shifts from ZF to ZG with age, in particular the notable CYP11B1 expression in more apical locations of the cortex, often within the ZG in all individuals older than 40 years of age (Extended Data Fig 4, Supplementary Fig 10), along with increased DLK1 immunoreactivity within the VSNL1+ ZG in older donors (Extended Data Fig3, panel d). However, we need to emphasize that the observed discrepancy in some cases between transcriptomic data and immunofluorescent validations can underly differences in dynamics of mRNA synthesis-to-protein translation. Based on these observations we renamed clusters with high CCN3, DLK1 and HSD3B2 expression to ZG/ZF rather than clear ZG populations.

Furthermore, to avoid misinterpretation we use the terminology “cell state” rather than “cell type” in Fig. 3 and manuscript and we are now putting the focus on cortical cell transcriptomic states rather than claiming novel cell types and heterogeneity, which was understandably met with concern.

For this, the subheading of the relevant section of the manuscript was changed ("Steroidogenic cell states and age-related shifts in the human adrenal cortex") and the relevant results are described more cautiously:

"…we reclustered ZG and ZF populations, identifying six ZG and three ZF transcriptionally-distinct subclusters (Fig. 3a,b). ZG cells were divided in the following clusters with varying expression levels of various marker genes: a) canonical aldosterone-producing VCAN+/CYP11B2+ cells (ZG-CYP11B2), b) VCANhigh/CYP11B2- cells (ZG-VCAN) corresponding to immature CYP11B2-/CYP17A1-/CYP11B1- cells in ZG17, c) a HSD3B2high cluster (named ZG/ZF based on HSD3B2 expression in both zones and some cells of this cluster being positive for ZF marker genes CYP11B1 and CYP17A1), d) a DLK1high cluster, e) a CCN3high cluster and f) a cluster enriched in a gene set involved in cholesterol metabolism (ZG-CHOL) (Fig. 3a,b, Supplementary Fig. 8)."

"Overall, our computational analysis inferred the presence of various transcriptional cell states within ZG, with a capsule-to-ZG DCNhigh transitional population, classical androsterone-producing CYP11B2+ population marked by variable levels of VCAN expression possibly reflecting ECM remodeling , putative intermediate transitional cells marked by DLK1 or CCN3 expression and a steroidogenic HSD3B2^{high}/CYP21A2/CYP11B1 population in older individuals highlighting the possibility of direct cortisol production upon aging rather than aldosterone synthesis. We further observe the hybrid nature of some of the identified cellular subclusters (DLK1+ and HSD3B2+), found in both ZG and ZF according to our biological validations and spatial transcriptomics."

<u>zG-VCAN population:</u>

In the revised manuscript the authors aimed to corroborate there bioinformatic findings using RNA-Scope data. While the expression of VCAN (Supplementary fig10) is indeed nicely restricted to the zG, I am not sure that they form a truly independent cluster. When looking at sup Fig. 10e it seems that the vast majority (if not all) of the zG cells are VCAN positive (the occasional DAPI+ VCAN- cell could be endothelial or immune).

We want to thank the reviewer for their suggestion to look further into this finding. In our transcriptomic data set, the ZG-VCAN state is mostly negatively defined based on lower mRNA levels of several steroidogenic markers. It is transcriptionally highly similar to ZG-CYP11B2 and shares specific marker genes, including HOPX and VSNL1 (see Fig. 3b), but is CYP11B2 negative on the transcriptomic level. However, in our effort to validate this state during the revision we performed additional in situ hybridizations for VCAN combined with ZG markers VSNL1, KCNJ5, HSD3B2 and CYP11B2 in male and female donors ranging from 20 to 53 years of age (Supplementary Fig 10). VCAN mRNA correlates strongly with ZG markers, and is co-expressed with CYP11B2 (detected through a monoclonal antibody), which is not in agreement with the transcriptomics results and can be explained by a mismatch between mRNA and protein content in cells, which can sometimes happen because the proteins can accumulate and be much more persisting even after mRNA is gone. This is, for example, a well-known phenomenon in cell cycle regulation. That led us to soften our previous claim and we are suggesting that VCAN can be an additional marker gene of ZG, supported by a previous study of extracellular matrix proteins of the adrenal gland[2], and the variability in VCAN levels may reflect ECM remodelling (possibly during vertical migration of ZG cells).

We describe concisely this finding in Results:

"Importantly, the two VCAN+ clusters (VCAN+/CYP11B2+ or ZG-CYP11B2+ and ZG-VCAN) shared a core of marker genes (i.e. VSNL1, HOPX) but were set apart based on CYP11B2 expression. However, VCAN detection through in situ hybridization revealed its expression in ZG in KCNJ5+ or CYP11B2+ steroidogenic rosettes, in almost negligible levels in upper HSD3B2+ ZF. This discrepancy between transcriptomic data and the histological detection can reflect differences between dynamics of mRNA transcription/degradation and protein synthesis (Supplementary Fig. 10)."

<u>ZG-HSD3B2 (CYP11B2 negative) population:</u>

I am puzzled by supplemental Figure.11: The signal for the CYP11B2 probe seems to be present in both zG and ZF (strong staining in the zF – which should be negative for Cyp11B2)– are you sure the probe does not also detect CYP11B1? If this was the case, the argument of CYP11B2 negative cells in the zG producing glucocorticoids is not valid, as these cells would be also negative for CYP11B1. When looking at the high resolution spatial data (Fig. 3D) the ZG-HSD3B2 population seems to be located more in the zF than the zG, at least for the F20 years and M13 years samples.

We want to thank the reviewer for correctly pointing out that the CYP11B2 probe is not be specific. To circumvent this, we now used a CYP11B1-specific RNAscope probe combined with a validated monoclonal antibody against CYP11B2, combined with HSD3B2 immunodetection. We consistently observed HSD3B2 expression in both ZG and ZF in all samples examined (please see Extended Data Fig 4, Supplementary Fig 10, 11). We agree with the reviewer that in younger individuals *HSD3B2*^{high} cells are preferentially located in ZF when looking at the mRNA (Fig. 7a), while in aged samples it is enriched in ZG (Supplementary Fig. 13d, sample M40 and low resolution Visium, Fig. 2d, Supplementary Fig. 5d). It is important to note that in our analysis of high-resolution Stereo-seq data *HSD3B2*^{high} cells were found in both ZG and ZF, but were completely absent in ZR in all three samples (Supplementary Fig. 13d). In addition, on our scRNA-seq embedding and hierarchical clustering shows the *HSD3B2*^{high} state to be closely related to ZG-CYP11B2 and ZG-VCAN, which are undoubtfully ZG clusters (Fig. 3a,b).

Combining these with our immunofluorescent experiments, we can confidently say that HSD3B2 is a marker of both ZG and ZF and we would cautiously hypothesise that this transcriptomic state corresponds to a transitional state between aldosterone- and cortisol-producing cells.

Additionally, we validate CYP11B1+/CYP11B2-/HSD3B2+ cells within ZG, especially detected within the outermost cortex of older donors, further supported by an earlier observation of ZF marker expansion at the expense of classical CYP11B2+ ZG with age[3] and our own observations through immunostainings for ZG marker VSNL1. *Thus,* we can further hypothesize that in older individuals the accumulation of *HSD3B2*^{high} cells in ZG may occur due to the age-related decrease in efficiency of ZG to ZF lineage conversion.

To address these nuances related to spatial distribution of cells with *HSD3B2*^{high} transcriptomic state we now renamed this cluster from ZG-HSD3B2 to ZG/ZF-HSD3B2 on all figures and clarified putative age-related spatial variability in the main text:

"ZG cells were divided in the following clusters with varying expression levels of various marker genes:.... a HSD3B2^{high} *cluster (named ZG/ZF based on HSD3B2 expression in both zones and some cells of this cluster being positive for ZF marker genes CYP11B1 and CYP17A1)...."*

"….we identified a steroidogenic population distinguished by higher expression levels of HSD3B2 compared to other ZG and ZF cells[21–23] (cluster 5, Fig. 1a, cluster ZG-HSD3B2, Fig. 3a,b). To investigate the functional identity of this cluster, we analyzed the expression of key enzymes involved in glucocorticoid biosynthesis and identified co-expression of HSD3B2, CYP21A2, and CYP11B1. Based on their transcriptional profile, we hypothesize their involvement in pregnenolone (17OH-pregnenolone) to progesterone (17-OH progesterone) conversion via HSD3B2 enzymatic activity and progesterone (17-OH progesterone) to 11-deoxycorticosterone (11-deoxycortisol) via CYP21A2 (expressed in all cortical zones but in higher levels in the HSD3B2+ cluster) (Fig. 2b-d). These cells lack CYP11B2 required for aldosterone synthesis despite expressing CYP17A1+/CYP11B1+ (Fig. 3b), suggesting direct cortisol production within the ZG, as supported by a previous proteomic study[33]. This surprising finding of classical ZF marker genes CYP17A1 and CYP11B1 in ZG has been reported to reflect the effect of aging on the human adrenal gland. Since hormone measurement in our fixed samples was not possible, we performed combined RNAscope in situ hybridization and immunofluorescence, confirming spatial co-expression of HSD3B2 and CYP21A2 and HSD3B2+/CYP11B1+/CYP11B2- cells within classical rosettes within of ZG exclusively in older donors (Extended Data Fig. 4, Supplementary Fig. 11). These findings are consistent with the spatial transcriptional data but functional validations for cortisol production from ZG are pending."

<u>ZG-DLK1 population (lines 187-197):</u>

The bioinformatic analysis further suggests the existence of a DLK1+/CYP11B1+/CYP11B2- transitional population, which is grouped together with the zG (labelled as zG-). However, the DLK1 antibody staining as well as the slide seq data places these cells within the zF (extended data Fig3d; Suppl. Fig14).

Firstly, it is important to note again that the ZG was annotated based on marker genes from a previous study[4], those being CYP11B2, KCNJ5, WNT4, VSNL1 and others, but not CYP11B1 (please see Methods). The DLK1^{high} state in our scRNA-seq data analysis was co-clustered with ZG-CCN3 (importantly, cluster 6: ZG/ZF-CCN3 on Fig.2a,c was composed of ZG-CCN3 and ZG-DLK1 following focused re-clustering - see Supplementary Fig.8 a-d, Fig. 3b), while CCN3 is a consistent ZG transcriptomic marker (supported by spatial transcriptomics Visium, Supplementary Fig 8, panel d, Stereo-seq, Supplementary Fig 13, panel c, Supplementary Fig 14c and RNAScope validation, Extended Data Fig 3a). In the high-resolution Stereo-seq spatial data set, the DLK1^{high} state is also present in both ZG and ZF, but not ZR, with preferential location in ZF of younger samples and ZG of aged sample (Supplementary Fig. 13d). The increased DLK1 expression in ZG specifically in older individuals is supported by our low resolution Visisum data (Supplementary Fig. 8f) and our new immunodetection experiments we added during the revision (revised Extended Data Fig 3, panels c, d).

To address nuances related to spatial distribution of the DLK1^{high} transcriptomic state we renamed this state from ZG-DLK1 into ZG/ZF-DLK1 on all figures and clarified putative age-related spatial variability in the main text:

"DLK1 and CCN3 immunostaining revealed DLK1 enrichment within ZF in young donors, shifted towards ZG in older (>40 years old) individuals and several cells positive for both proteins, which led us to consider DLK1+ cells as another ZG/ZF cluster (Extended Data Fig. 3d,e)."

"To better understand the spatial arrangement of these transcriptionally distinct ZG populations and to contextualize their potential functional roles we turned to high-resolution spatial

transcriptomics..... Most apical populations within ZG were VCAN^{high} and CCN3^{high}, CYP11B2^{high}, while the DLK1^{high} population expanded much further basally in young donors and appeared more restricted to ZG in the older individuals... (Fig. 3d, Supplementary Figs. 13-14)."

"We further observe the hybrid nature of some of the identified cellular subclusters (DLK1+ and HSD3B2+), found in both ZG and ZF according to our biological validations and spatial transcriptomics."

The statement: "This pattern of presence of CCN3 and DLK1 within the CYP17A1low/CYP11B1+ population (potentially cells slowly differentiating towards ZF) reflects transcriptional states of an intermediate steroidogenic population towards maturation" appears too strong without a direct lineage confirmation.

We understand the concern of the reviewer and we rephrased this sentence to express that this is a non-validated hypothesis, based on the low levels of classical ZF markers CYP17A1 and CYP11B1:

"Importantly, DLK1^{high} and CCN3^{high} clusters were characterized by CYP11B2-/CYP17A1^{low}/CYP11B1^{low} signature, expressing those at a lower level than confidently ZF-assigned clusters, suggesting they may represent ZG to early ZF transition, pending lineage tracing experiments (Fig. 3a,b, Supplementary Fig. 8)."

More generally, why attributing the name "zG" to a cell cluster expressing CYP11B1 (and not CYP11B2)? I am not surprised then that DLK1+ cells are found within the zF in immunostaining and in slide-seq analysis.

We would like to clarify that our initial annotation approach utilized ZG and ZR markers from Baker and colleagues. ZG cells were annotated based on established marker genes *CYP11B2, KCNJ5, LGR5, WNT4, CCN3, VSNL1*, while ZR cells were annotated as *CYB5A*-, *SLC13A5*-, *SLC27A2*-expressing cells (please see Methods). The remaining cells were annotated to ZF, and where largely *CYP11B1*^{high}, while *CYP11B1* levels of expression were lower in ZG cells (UMAP and Visium, Supplementary Figure 21, panel d), even though not completely negligible, and higher in the HSD3B2+ and CHOL^{high} clusters (dotplot, Supplementary Fig 14b), which we have interpreted as a mix of ZG and ZF.

Additionally, based on our latest validations of CYP11B1/CYP11B2/HSD3B2 stainings we confidently report CYP11B1-expressing cells within ZG in older individuals, in line with a previous spatial transcriptomic study on the aged human adrenal. Thus, CYP11B1 transcriptomics analysis shows that this marker gene is not as clear cut in the ever-regenerating human adrenal cortex upon aging. During the revision, we performed stainings against VSNL1 and KCJN5, and observed their specific localization in high levels in ZG, while in a few cases, in a gradient with very low levels in upper ZF, so we can confidently consider them robust ZG marker genes. It is very likely that these gradients reflect reported ZG-to-ZF differentiation established by rodent models and the centripetal model of differentiation, and the onset of *CYP11B1* expression in those cells undergoing this transition can also occur in healthy younger individuals.

To clarify this rather interesting finding of *CYP11B1* expression we included the following phrases in the manuscript:

"This surprising finding of classical ZF marker genes CYP17A1 and CYP11B1 in ZG has been reported to reflect the effect of aging on the human adrenal gland[24]. ....we performed combined RNAscope in situ hybridization and immunofluorescence, confirming spatial co-expression of HSD3B2 and CYP21A2 and HSD3B2+/CYP11B1+/CYP11B2- cells within classical rosettes within of ZG exclusively in older donors (Extended Data Fig. 4, Supplementary Fig. 11), offering evidence for a more complex CYP11B1 expression pattern, not exclusive to the ZF, as opposed to what is considered common knowledge (represented in Fig. 2e)."

Taken together, the claim that the study revealed " previously unrecognized cell populations, especially in zona glomerulosa" (abstract) seems not 100% validated.

We hope that our new validations of the identified states during the revision and the clearer explanation of our findings support that several of the transcriptomics-identified subgroups within ZG may reflect states that correspond to altered signaling (as in the example of DLK1) or transitions between ZG and ZF.

However we completely understand the concern of the reviewer and reformulated the relevant phrase in the Abstract as shown below:

"In the adult human adrenal, we revealed novel cell states which differ upon aging, especially in zona glomerulosa..."

<u>Lineages (lines 282 onwards):</u>

<u>Transition 1:</u>

This transition is in agreement with capsular recruitment to the zG and the position of the cells (spatial mapping). The gap between cluster 1 and 3 and the sudden switch of expression profiles (gene program transitions in suppl. Fig28j) is surprising and raises the question to what extent this transition occurs in adults.

We thank the reviewer for this observation. Indeed, the apparent gap between clusters 1 and 3 and the abrupt transcriptional switch are fully consistent with the underlying biology of this process. We do not expect extensive recruitment of capsular mesenchymal cells within healthy adult adrenal tissue, supported by the astonishingly few WT1+ cells in our data set, but rather, only under stress or injury.

This is also evident in our mouse adult adrenal data set, where direct transitions from mesenchymal cells to cortical populations are similarly rare (e.g., 1: Mesenchyme–Sparcl1 to 4: ProlCS-proliferating cortical cells and 1: Mesenchyme–Sparcl1 to 5: Transition–Wt1; Fig. 6a).

In both species, our data indicate that the dominant source of daily adrenocortical turnover in adulthood is the population of more differentiated, proliferative NR5A1+/EZH2+ cortical cells, while mesenchymal-to-cortex transitions appear to represent a slow or conditionally activated pathway rather than a major homeostatic mechanism.

Thus, the sparse representation of this transition in the adult human UMAP is biologically plausible and aligns with both spatial mapping and our cross-species observations.

The under-representation of capsule-to-cortex transitions in our data set of human adult adrenal glands is briefly mentioned in the revised manuscript:

"Inferred transition 1 corresponds to the canonical transition described in mice from GLI1+/PTCH1+ mesenchymal stem cells of the adrenal capsule towards ZG cells[14–16] (Extended Data Fig. 5), characterized by Wt1 expression in rodents. Of note, WT1+ cells are found in very low numbers in the adult human cortex (Fig. 4c-f)."

"When examining this transition … we did not find intermediate transitory states with exclusive markers, suggesting fast direct differentiation of mesenchyme into cortex and that this mechanism of new cell generation in the cortex is not extensively utilized in the healthy human adult gland (Supplementary Fig. 28)."

<u>Transition 2:</u>
The authors suggest the existence of VSMC+ progenitor cells throughout the adrenal cortex that can transition towards the steroidogenic fate. This is potentially very interesting, but formal proof for this transition is in my opinion still missing.

We completely understand the reviewers point, and we agree. Unfortunately, since this transition is human-specific, we cannot use mouse lineage tracing to validate it and such experiments aren't feasible in adult human tissue nor in current organoid models of human adrenal cortex, which model fetal tissue but not adult.

Also, although adult cortical tissue itself may be modelled in the future – introducing vasculature and VSMCs can be tricky and represents a common problem for organoid development.

Recognizing these limitations, we have taken steps toward orthogonal support where possible. Our adult human mosaic clone (mCA) analysis, although dependent on rare clinical samples, provides an independent line of evidence for mesenchymal-derived cells contributing to cortical lineages. We plan to continue refining organoid models in collaboration with other groups and to analyze additional adrenal samples with informative clonal patterns that may further illuminate this putative transition.

While we cannot yet provide definitive lineage proof, we believe that the convergence of our transcriptomic, spatial, and clonal data supports the plausibility of this transition, and we now emphasize its inferred nature in all places we refer to hypothesized transitions based on our single cell transcriptomic data set and our non-validated hypotheses more clearly in several places in the revised manuscript:

"Our systematic analysis of cell states bridging cortical and mesenchymal clusters, and analysis of proliferation signatures, suggests three potential sources of newly generated steroidogenic cells…."

"Our findings suggest a new proposed model of human adrenocortical turnover…. Even though we lack experimental data on the precise role of these populations in the adrenal cortex and functional validations in iPSC-derived models will be necessary, these observations open many directions for further research, as they offer a working hypothesis informing functional experiments at the time they will become possible in in vivo or in vitro systems."

"Additionally, we … proposed a working hypothesis of renewal and dynamics in human adrenal cortex…"

Also I am not sure I completely follow the arguments based on mCA transmission. In line 318,

the authors link VSCMs with the zG (suppl. Fig31.b&c), but in Fig.4K this link is not represented? On the other hand, the authors claim a strong link of VSMCs to the zR, but while the majority of VSMCs appear to carry the rearrangement (11/18), only 7 out of 473 ZR cells are aberrant. Wouldn't you expect the ZR to have a much higher rate of rearranged cells?

Our single cell transcriptomics analysis of the combined data set of all 11 tissue donors indicates that the VSMC-derived transition is bidirectional and cluster 5:TRANS-TFPI2-USP53 contains two transcriptionally distinct directions: 1) a TFPI2^{+}/GNG11^{+} branch towards ZG/ZF, and 2) an USP53^{+} branch towards ZR (Supplementary Fig. 27a and Supplementary Fig. 31d).

However, only the VSMC cluster, the TFPI2^{+}/GNG11^{+} transition and ZG and ZF contain aberrant cells, while very few aberrant cells are found in ZR (3 cells in total) (Fig. 4g, Supplementary Fig. 31c). The USP53^{+} branch, which we hypothesize to connect VSMCs to ZR, contains no aberrant cells in this sample. Thus, the low number of aberrant ZR cells is consistent with the absence of aberrant cells in the hypothesized transitional USP53^{+} subpopulation that may give rise to ZR.

The most likely explanation is sampling limitation: the USP53^{+} branch is represented by very few cells overall (similarly to BRIDGE cells and left-side TRANS-TFPI2-USP53), making it statistically unlikely to capture mCA-carrying cells within that small fraction.

An alternative, non–mutually exclusive interpretation is that transitions toward ZF/ZG and ZR may involve shared early intermediate states (e.g., TRANS-RGS5, BRIDGE) but occur in distinct spatial microenvironments, with partially overlapping transcriptomic signatures. In this case, the apparent bifurcation in the embedding may not represent a literal lineage split, but rather parallel transitions with independent spatial origins.

We clarified this logic in the revised manuscript and added explicit text explaining why the mCA data support the ZG/ZF-contribution but do not allow us to draw conclusions about the ZR-contribution:

"The pattern of mCAs in this sample, composed of mostly genetically normal ZR cells (only 3 cells within ZR are aberrant) and aberrant VSMCs, ZG and ZF cells, supports the inferred transition from VSMCs through TFPI2+/GNG11+ cells differentiating towards cells of the ZF and ZG, albeit pending biological validation. The VSMC-to-intermediate USP53+ state predicted from our single cell analysis to give rise to ZR cells does not contain any genetically aberrant cells, thus not providing evidence supporting this transition, which requires further experimental validation in systems that will allow lineage tracing."

<u>Transition 3:</u>

The authors further propose the existence of an NR5A1+EZH2+ progenitor cell population that can give rise to all three steroidogenic zones. As outlined in my previous review I am not convinced that these cells represent true progenitors. Indeed TOP2A, UBE2T, SMNT and to a lesser extent also EZH2 are all strongly linked to cell cycle progression. Although the authors have now 'nuanced 'the text, the cluster is still named as Prog (for progenitors) and are displayed (Figure 4K) as an independent cell population that has one-way differentiation capacity towards zG, zF, zR. The confusing Velocity analysis in different samples (suppl Fig.32) does not help in this context. (incidentally, I would have expected EZH2 antibody staining to be nuclear (Extended Fig. 6)

We are thankful about this comment and understand that naming proliferating cells progenitors is raising concerns. We initially used that terminology to be consistent with those accepted within the adrenal cortex renewal research field. Specifically, when it comes to mouse model studies proliferating Sf1+/Shh+/Cyp11b2- adrenocortical cells are titled "progenitors" (for example, https://www.nature.com/articles/s41574-021-00491-4 , Figure 1), despite the fact that Shh expression is widespread in mouse ZG and Cyp11b2 is characteristic feature of very late differentiated aldosterone-producing cell lineage. In our cross-species comparison human proliferating cortical cells co-integrate with mouse proliferating cortical cells (Fig. 6c) and partially shared transcriptomic signature (Fig. 8d,e,g). Thus, our cross-species comparison led us the use of the term "adrenocortical progenitors".

Nevertheless, we agree with the reviewer and provided data are not sufficient to consider proliferating cortical cells as progenitors. We edited the manuscript and figures and are now referring to cycling SF1+ cells as "proliferating cortical state (ProlCS) cells" in all places we refer to these, and revised the suggested schematic in Fig. 4k to include all possible lineage directions, which cannot be conclusively proven with this manuscript, but we are hopeful that they will be investigated by future lineage tracing experiments in organoids.

In regards to the EZH2 staining, we screened multiple anti-EZH2 antibodies, and now included the new staining in revised Extended Data Fig6, panel d and the new antibody information in the supplementary notes and online reporting summary.

The model in Figure 4K tries to conceptualize the complex cellular dependencies and lineages, but without additional data it may mislead the scientific community. I realise that obtaining functional data from human material is difficult, but I believe this would be required to confirm these concepts. Since the attempted in vitro work did not yield conclusive results, I think the authors need to be much more careful in their statements.

We understand that our previously suggested model is raising concerns because it is based on inference and lack of experimental evidence. We carefully edited the heading in Fig 4k to "scRNA-seq analysis-based putative cortex renewal model" and redesigned the schematic to show that the direct lineage transitions and progenitors that we are suggesting are not definite and require further experimental validations and, which may be achieved in the future with state-of-the-art adrenocortical organoids derived from human iPSCs.

We also made additional points in the text when referring to the scheme in Fig 4k panel and its inferred unvalidated nature:

Results section, relevant to Fig. 4k:

"This model remains to be experimentally validated at the level of lineage tracing and functional tests. Even though we currently lack experimental data on the precise role of these populations in the human adrenal cortex, and the functional validations in iPSC-derived models will be necessary, these observations open many directions for further research, as they offer a working hypothesis informing functional experiments at the time they will become possible in in vivo or in vitro systems."

Discussion section:

"In summary, our results ***suggest a working hypothesis*** *of the mechanisms of cortical self-renewal shared between adult murine and human adrenals, which include two non-cortical*

*sources of cells giving rise to cortical subpopulations. First, capsular Gli1/2+ mesenchymal stem cells **might contribute** to ZG in both mouse and human but to a very small degree in healthy adult adrenals in human. The second stem cell source in mice, marked by Wt1 $^{+}$/Gata4 $^{+}$ expression, has been shown to contribute directly to the ZF. Freedman et al. demonstrated this pathway experimentally by showing intact ZF formation even upon targeted deletion of Nr5a1 (gene coding for SF1) in ZG cells[48]. Our **human data suggest a comparable trajectory**, indicating that ZG-independent **ZF formation may be conserved across species**. Despite the existence of WT1+ cells of the coelomic epithelium contributing to the adrenocortical primordium in early human fetal development[5], in the adult adrenals, WT1+ cells are almost completely absent. Instead, the second adrenocortical stem cell pool originates from human VSMCs, present in capsule, uppermost ZG and lowermost ZR. This transition contributes to ZF and ZR **according to the computational inference**, with previously uncharacterized intermediate states. Based on the dynamics of gene expression programs in these transitional populations we expand the centripetal model **by computationally defining** a complementary cortical renewal from scattered NR5A1+ dividing cells and VSMCs both found throughout the entire cortex."*

As the reviewer can see (in bold text), we use a very soft suggestive language and clearly point out that these are computationally inferred features requiring experimental studies.

Also, we mention the limitations here:

"Importantly, we recognize that our proposed model of adrenocortical renewal, centered around several inferred transitions, is inherently limited by the observational nature of human transcriptomic data including RNA velocity and directional gene expression changes and are not validated by experimental evidence in intact human adrenal tissue. Such evidence can be potentially obtained when human adrenocortical organoids replicating the adult human adrenal gland cellular composition and physiology are established."

An additional point to consider is that the transcriptional landscape of a complex system does not necessarily explain one-to-one the overt cell phenotypes. Dramatically different cellular states can emerge from quantitative or qualitative changes in a relatively small number of key proteins, once these proteins begin to be synthesized or activated. As a result, a system can display smooth, graded changes at the mRNA level while exhibiting sharp, apparently discontinuous boundaries in histology and morphology. This likely explains why the transition between adrenal cortical zones appears very abrupt in terms of tissue architecture and cell morphology, whereas the underlying transcriptional changes are gradual in all available datasets, including our mouse and human single-cell and bulk RNA-seq data. In these datasets, the mRNA pools shift progressively along the medio-lateral adrenal axis, but once critical regulators cross certain thresholds at the protein level - or become post-translationally modified - the resulting changes in steroidogenic machinery, cell polarity, or extracellular matrix interactions can be highly discrete. In other words, the transcriptional continuum can be funneled by nonlinear regulatory events into distinct phenotypic "attractors" that correspond to well-defined zones. More broadly, cell phenotypes are shaped not only by the composition of the mRNA pool but also by multiple additional layers of regulation that we did not address here, including translational control, protein stability and turnover, subcellular localization, post-translational modifications, epigenetic state, and local signaling environment. Disentangling how these regulatory tiers interact with transcriptional gradients to produce the sharply demarcated adrenal zones observed in vivo remains an important direction for future work.

We added the following shortened passage to the Discussion about this:

"A noteworthy point is that transcriptional landscapes might not directly explain cell phenotypes. Markedly different cellular states can arise from changes in a small set of key proteins, so mRNA profiles may vary smoothly while histological and morphological boundaries remain sharp. This likely explains why adrenal cortical zones show abrupt transitions in tissue architecture, even though our mouse and human transcriptomic data reveal gradual shifts in gene expression. Cell identity is shaped not only by mRNA levels but also by additional regulatory layers - translation, protein turnover, modifications, and local signaling - that we did not examine here and that warrant future investigation."

Finally, we would like to note that we have softened many of our conclusions and more explicitly emphasized the need for functional validation throughout the manuscript. **We share the reviewer's concern about possible misinterpretation or over-interpretation of our data and agree that, in several instances, our inferred analyses and corresponding results should not be taken as definitive / validated ground truths.** We introduced the necessary statements and self-critical disclaimers to make sure this is clear. We hope the reviewer still appreciated the importance of provided data and corresponding analyses, including new additions during this revision, which shall provide a foundation for hypothesis selection and further experimental work on human adrenal glands.

This is a final line in our manuscript clearly phrasing this statement:

"Even so, we emphasize that definitive evidence for these lineage relationships will require functional validation, which is currently not feasible in human tissue. Thus, we would encourage the readers to interpret the suggested transitions and self-renewal mechanisms in human adrenals as a working hypothesis requiring further experimental exploration."

References

1. Liu, B. *et al.* An entropy-based metric for assessing the purity of single cell populations. *Nat Commun* **11**, 3155 (2020).

2. Kremer, J. L. *et al.* Proteomic profiling of the extracellular matrix in the human adrenal cortex. *Matrix Biol Plus* **23**, 100158 (2024).

3. Iwahashi, N. *et al.* Single-cell and spatial transcriptomics analysis of human adrenal aging. *Molecular Metabolism* **84**, 101954 (2024).

4. Baker, J. E. *et al.* Targeted RNA sequencing of adrenal zones using immunohistochemistry-guided capture of formalin-fixed paraffin-embedded tissue. *Molecular and Cellular Endocrinology* **530**, 111296 (2021).
